# Fluorophore-Labeled Pyrrolones Targeting the Intracellular Allosteric Binding Site of the Chemokine Receptor CCR1

**DOI:** 10.1021/acsptsci.4c00182

**Published:** 2024-06-21

**Authors:** Lara Toy, Max E. Huber, Minhee Lee, Ana Alonso Bartolomé, Natalia V. Ortiz Zacarías, Sherif Nasser, Stephan Scholl, Darius P. Zlotos, Yasmine M. Mandour, Laura H. Heitman, Martyna Szpakowska, Andy Chevigné, Matthias Schiedel

**Affiliations:** † Department of Chemistry and Pharmacy, Medicinal Chemistry, Friedrich-Alexander-University Erlangen-Nürnberg, Nikolaus-Fiebiger-Straße 10, Erlangen 91058, Germany; ‡ Institute of Medicinal and Pharmaceutical Chemistry, Technische Universität Braunschweig, Beethovenstraße 55, Braunschweig 38106, Germany; § Immuno-Pharmacology and Interactomics, Department of Infection and Immunity, Luxembourg Institute of Health, Rue Henri Koch 29, Esch-sur-Alzette L-4354, Luxembourg; ∥ Faculty of Science, Technology and Medicine, University of Luxembourg, 2 Avenue de l’Université, Esch-sur-Alzette L-4365, Luxembourg; ⊥ Leiden Academic Centre for Drug Research (LACDR), Division of Chemistry, Leiden University, Leiden 2333 CC, Netherlands; # Department of Pharmaceutical Chemistry, Faculty of Pharmacy and Biotechnology, the German University in Cairo, New Cairo City 11835, Cairo, Egypt; ¶ Institute for Chemical and Thermal Process Engineering (ICTV), Technische Universität Braunschweig, Langer Kamp 7, Braunschweig 38106, Germany; ∇ School of Life and Medical Sciences, University of Hertfordshire Hosted by Global Academic Foundation, New Administrative Capital, Cairo 11578, Egypt; ○ Oncode Institute, Leiden University, Leiden 2333 CC, Netherlands

**Keywords:** drug discovery, fluorescent probes, GPCRs, click chemistry, medicinal chemistry, target engagement

## Abstract

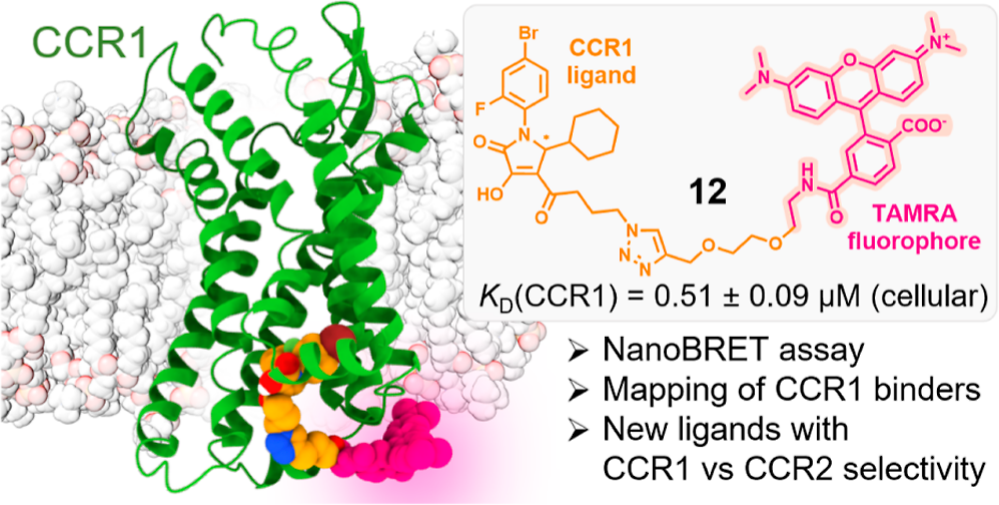

In this study, we describe the structure-based development of the first fluorescent ligands targeting the intracellular allosteric binding site (IABS) of the CC chemokine receptor type 1 (CCR1), a G protein-coupled receptor (GPCR) that has been pursued as a drug target in inflammation and immune diseases. Starting from previously reported intracellular allosteric modulators of CCR1, tetramethylrhodamine (TAMRA)-labeled ligands were designed, synthesized, and tested for their suitability as fluorescent tracers to probe binding to the IABS of CCR1. In the course of these studies, we developed LT166 (**12**) as a highly versatile fluorescent CCR1 ligand, enabling cell-free as well as cellular NanoBRET-based binding studies in a nonradioactive and high-throughput manner. Besides the detection of intracellular allosteric ligands by direct competition with **12**, we were also able to monitor the binding of extracellular antagonists due to their positive cooperative binding with **12**. Thereby, we provide a straightforward and nonradioactive method to easily distinguish between ligands binding to the IABS of CCR1 and extracellular negative modulators. Further, we applied **12** for the identification of novel chemotypes for intracellular CCR1 inhibition that feature high binding selectivity for CCR1 over CCR2. For one of the newly identified intracellular CCR1 ligands (*i.e.*, **23**), we were able to show CCR1 over CCR2 selectivity also on a functional level and demonstrated that this compound inhibits basal β-arrestin recruitment to CCR1, thereby acting as an inverse agonist. Thus, our fluorescent CCR1 ligand **12** represents a highly promising tool for future studies of CCR1-targeted pharmacology and drug discovery.

The CC chemokine receptor type 1 (CCR1) is a G protein-coupled receptor (GPCR) that belongs to a family of more than 20 chemokine receptors, which have emerged as attractive targets for drug discovery. Approximately 50 endogenous protein-based ligands, so-called chemokines, interact with these receptors, thereby mediating basal and inflammatory leukocyte trafficking.^1^ CCR1 is expressed on different immune cells, including monocytes, macrophages, neutrophils, T-lymphocytes, basophils, and dendritic cells.^1^ At least nine different chemokines (*i.e.*, CCL3, CCL5, CCL7, CCL8, CCL13-16, and CCL23) are able to bind to and activate CCR1,^2−4^ thus highlighting the high promiscuity of this receptor regarding its extracellular chemokine binding site. Recently, reported cryogenic-electron microscopy (cryo-EM) structures of the CCR1–G_i_ complex bound to different CCL15 truncations provided the first structural insights into CCR1 activation by endogenous chemokines.^5^ Since the early 2000s, CCR1 is known to play an important role in rheumatoid arthritis (RA), multiple sclerosis (MS), Alzheimer’s disease (AD), and multiple myeloma (MM).^6−10^ More recent publications also suggest CCR1 as a potential drug target for the treatment of systemic fungal infections, obesity-associated cancer, Behcet’s disease, and COVID-19.^11−15^ This high therapeutic potential has prompted intense efforts in the development of small-molecule antagonists of CCR1.^7,10,16−28^ Due to highly promising results from preclinical studies, several small-molecule CCR1 antagonists, including BX-471 (**1**, also referred to as BAY 86-5047 or ZK-811752) and BMS-817399 (**2**) entered clinical trials (see Figure 1). However, none of the clinically evaluated CCR1 antagonists have reached market approval so far, which is mainly attributed to their limited therapeutic efficacy.^29,30^ For example, BX-471 (**1**) failed to show efficacy in a phase II clinical trial in patients with relapsing remitting MS.^31^ One possible explanation for the limited therapeutic efficacy of **1** might be that this compound is unable to inhibit basal β-arrestin-2 recruitment to CCR1, which leads to an insufficient blockage of constitutive receptor internalization.^32^ Similarly, BMS-817399 (**2**) did not evoke statistically significant differences compared to placebo in phase II trials in patients with RA, despite achieving excellent coverage of the receptor.^22^ Due to these disappointing results from clinical studies, the development of other CCR1 antagonists, like the phase I clinical candidate BI-639667 (**3**), was halted.^26^ This highlights the urgent need for novel approaches to target CCR1.

**Figure 1:**
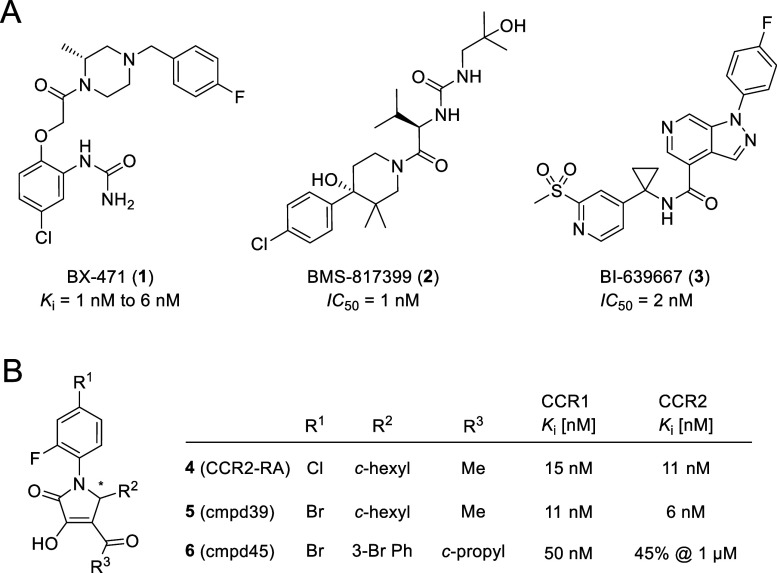
Chemical structures and biological activities of the selected CCR1 antagonists. (A) Previous phase II clinical candidates BX-471 (**1**)^16^ and BMS-817399 (**2**),^22^ as well as the previous phase I clinical candidate BI-639667 (**3**).^26^ (B) CCR2-RA (**4**),^40,41^ an intracellular allosteric antagonist of CCR1 and CCR2, as well as its analogues **5**–**6**.^33^

**Scheme 1:**
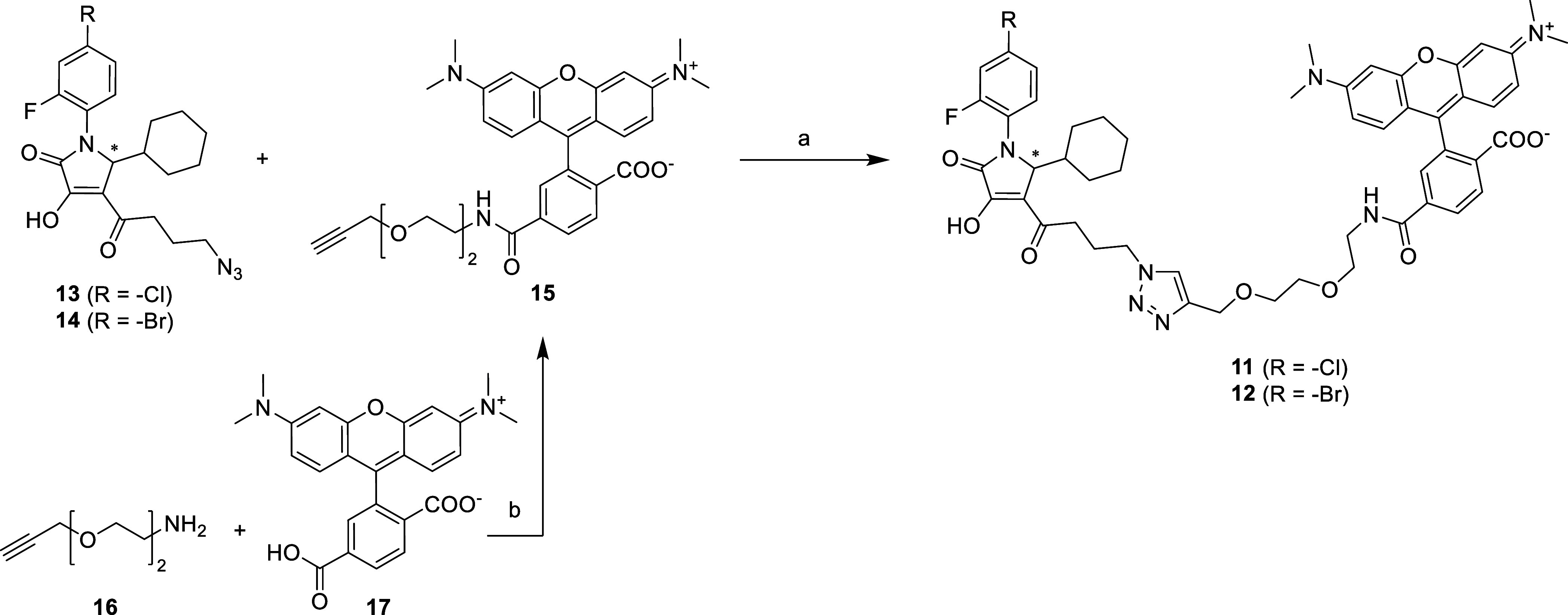
Synthesis of the Fluorescent CCR1 Probes **11**–**12** Reagents and conditions: (a) CuSO_4_·5 H_2_O, sodium ascorbate, TBTA, water/*tert*-BuOH/DMF mixture (1:1:1 (v/v/v)), rt, 1 h, 24–25% yield; (b) **17**, TBTU, DMF, DIPEA, 0 °C, 15 min, then **16**, rt, 2 h, 63% yield.

Highly interesting in this respect was a discovery by Ortiz Zacarías *et al.*(33) In their study, the authors showed that the CCR2-targeted and CCR2-RA (**4**)-derived radioligand [^3^H]CCR2-RA-[*R*] also binds to CCR1 with affinities similar to those reported for CCR2. This is especially intriguing since CCR2-RA (**4**, Figure 1B) has previously been identified as an intracellular allosteric CCR2 antagonist *via* X-ray cocrystallography.^34^ With this, the authors provided the first evidence for the existence of a druggable intracellular binding site (IABS) at CCR1.^33^ In a structure-affinity relationship (SAR) study, several pyrrolone-based derivatives, such as **5**, were identified that showed dual inhibition of CCR1 and CCR2. Additionally, also preferential CCR1 antagonists exemplified by **6** were discovered, thus indicating that CCR1 over CCR2 selectivity can be achieved by targeting the IABS of CCR1.

In general, a druggable IABS that allows the binding of small molecule antagonists was recently identified by X-ray cocrystallography for several other GPCRs, including the chemokine receptors CXCR2,^35^ CCR7,^36^ CCR9,^37^ as well as the beta-2 adrenergic receptor (β_2_AR).^38^ In addition, a druggable IABS has been suggested for several other GPCRs.^39^ Compared to antagonists that bind to an orthosteric site that is located within the helical bundle and accessible from the extracellular environment, ligands targeting the IABS feature a new dual mechanism of specific GPCR modulation, which is characterized by (i) a stabilization of the inactive receptor conformation resulting in a negative cooperativity with the orthosteric agonist and (ii) a direct steric blockage of intracellular transducer (G protein and/or β-arrestin) binding.^34,40,41^ Taking advantage of this new mode of GPCR inhibition is especially attractive for GPCRs, for which the development of orthosteric antagonists has shown only limited therapeutic success, such as CCR1. In general, targeting an allosteric site at GPCRs has the potential to result in highly selective receptor modulation, as exemplified by the CCR9-targeted vercirnon,^42^ since allosteric binding pockets tend to be less conserved than orthosteric binding sites.^43^ Intracellular allosteric GPCR antagonists are of special interest, as they are noncompetitive toward the orthosteric agonists,^40,44,45^ allowing for an efficient signaling blockade even in the presence of very high agonist concentrations. In the case of CCR1, IABS-targeted ligands were even shown to inhibit basal G protein activation in addition to agonist-induced G protein activation, thereby acting as inverse agonists.^33^ All of these features highlight the immense potential of intracellular allosteric GPCR inhibitors, thus providing a highly promising alternative to orthosteric antagonists for inhibiting GPCR-mediated signaling in a therapeutic setup.

For the discovery of novel lead structures for intracellular allosteric CCR1 inhibition, the availability of molecular tools that allow straightforward ligand identification and characterization is of utmost importance. With the radioligand [^3^H]CCR-RA-[*R*],^33^ Ortiz Zacarías *et al.* have therefore reported a highly valuable molecular tool that can be utilized to detect binding to the IABS of CCR1. However, radioligand binding assays are accompanied by some disadvantages, such as high infrastructure requirements according to radiation protection measures, the production of radioactive waste, and often laborious (heterogeneous) assay protocols, including washing steps for removing the unbound radioligand prior to the assay readout. The latter is also an important reason why radioligands are often not well-suited for continuous assay readouts, the detection of low-affinity binders, and cellular target engagement studies investigating their binding to intracellular target sites.^46^ Recently, we and others reported the development of fluorescent tracers targeting the IABS of CCR2, CCR9, and CXCR2.^44,45,47,48^ These molecular tools were successfully applied for cell-free and cellular binding studies using the nonradioactive NanoBRET technology. In the course of the development of our biarylsulfonamide-based CCR2 tracer **7** (Figure 2), which showed three digit nanomolar binding to CCR2 in a membrane-based (*K*_D_(CCR2) = 266 nM) and cellular setup (*K*_D_(CCR2) = 114 nM), respectively, but no relevant binding to CCR1, we have already described the pyrrolone-based fluorescent probes **8** and **9** (Figure 2).^47^ Due to their only moderate CCR2 affinities, these pyrrolone-based ligands (**8**–**9**) were not considered for further characterization as fluorescent probes for CCR2. However, with respect to potential applications as molecular tools for CCR1, these pyrrolone-based fluorescent ligands came back into our focus.

**Figure 2:**
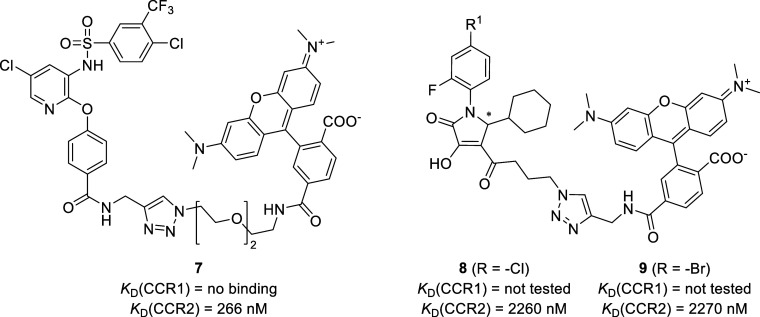
Chemical structures and binding affinities of previously reported biarylsulfonamide- or pyrrolone-based fluorescent probes **7**–**9**.^47^

Herein, we aimed at developing a fluorescently labeled intracellular CCR1 ligand to provide a nonradioactive molecular tool that allows us to directly study ligand binding to the IABS of CCR1 both in a cell-free and a cellular environment.

## Results and Discussion

**Figure 3:**
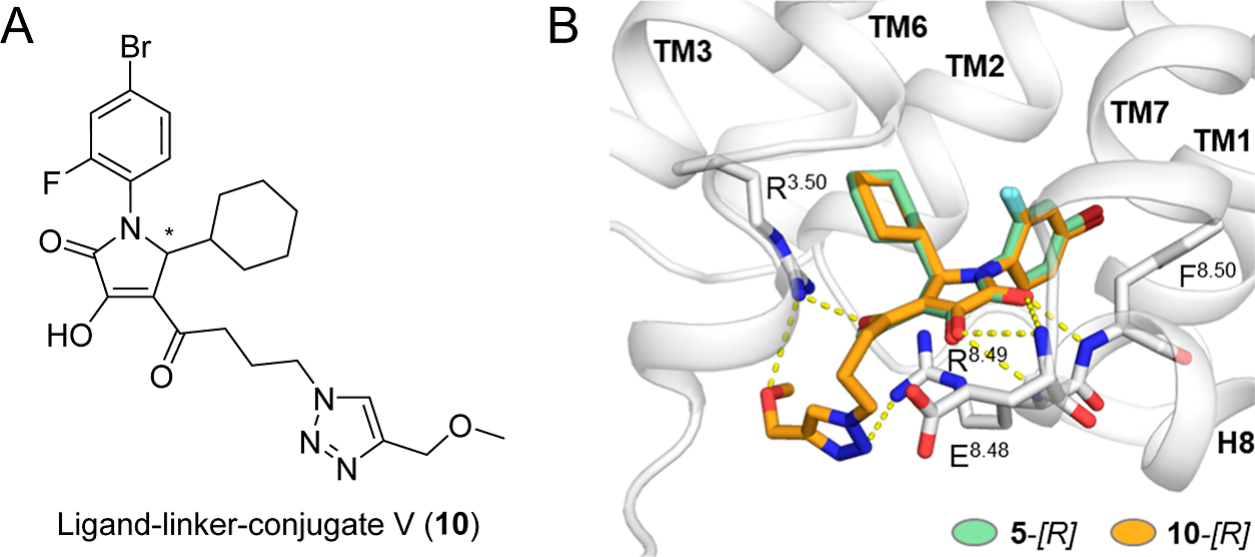
Design of fluorescent ligands targeting the intracellular allosteric binding site of CCR1. (A) Chemical structure of the CCR1 ligand-linker conjugate V (**10**), identified by molecular docking as a suitable template for the design of fluorescent CCR1 ligands. (B) Overlay of the predicted binding modes of the parental intracellular CCR1 inhibitor **5**-[*R*] with the ligand-linker conjugate V (**10**-[*R*]). Docking scores (Vina): **5**-[*R*] = –10.4 and **10**-[*R*] = –9.9.

The design of our fluorescent probes targeting the IABS of CCR1 was based on the pyrrolone scaffold of the previously reported intracellular dual CCR1/CCR2 inhibitors **4**–**5** (Figure 1).^33^ We selected these pyrrolone-based CCR1/CCR2 inhibitors as a starting point for our studies for the following two reasons (i) **4** and **5** are high-affinity ligands for the IABS of CCR1;^33^ (ii) the cocrystal structure of CCR2 in complex with **4** (PDB 5T1A)^34^ enables rationalizing the binding of pyrrolone-based ligands to the IABS of the closely related CCR1 by using a homology model. Based on a previously reported CCR1 homology model,^33^ we performed molecular docking studies with pyrrolone-based ligand–linker conjugates (Figures 3 and S1). Given the similar docking scores that were obtained for the (*R*)-enantiomers of the ligand–linker conjugate V (**10**) and the high affinity intracellular CCR1/CCR2 ligands **4** and **5**, respectively, we identified the C_α_-position of the acetyl groups of **4** and **5** to be suitable for the installation of a linker while retaining CCR1 affinity. These results are consistent with the docking studies that were performed in the course of the development of our CCR2 fluorescent tracers.^47^

In addition to the already synthesized fluorescently labeled pyrrolones **8** and **9**,^47^ we aimed for analogues **11**–**12** with a longer polyethylene glycol (PEG)-based linker between the CCR1-targeted pyrrolone core and the fluorophore, in order to provide initial insights into linker length influences on CCR1 affinity. As our docking studies indicated that the triazole moieties of linker-ligand conjugates are able to interact with a polar amino acid at the entrance of the intracellular allosteric binding pocket of CCR1 (*i.e.*, R^8.49^, see Figure 3), we were able to apply our previously established click chemistry-based protocol for the conjugation of azido-functionalized pyrrolones with a cell-permeable TAMRA-based fluorophore.^47^ For the synthesis of new fluorescently labeled pyrrolones **11**–**12** (Scheme 1), we used the previously reported azido-functionalized pyrrolones **13**–**14** and conjugated them with 6-TAMRA-PEG_2_-alkyne **15** by means of straightforward Cu(I)-catalyzed azide–alkyne cycloaddition (CuAAC).^49−51^ The clickable 6-TAMRA-PEG_2_-alkyne **15** was obtained by amide bond formation between H_2_N-PEG_2_-alkyne (**16**) and 6-carboxy-tetramethylrhodamin (**17**) using TBTU as a coupling reagent.

**Figure 4:**
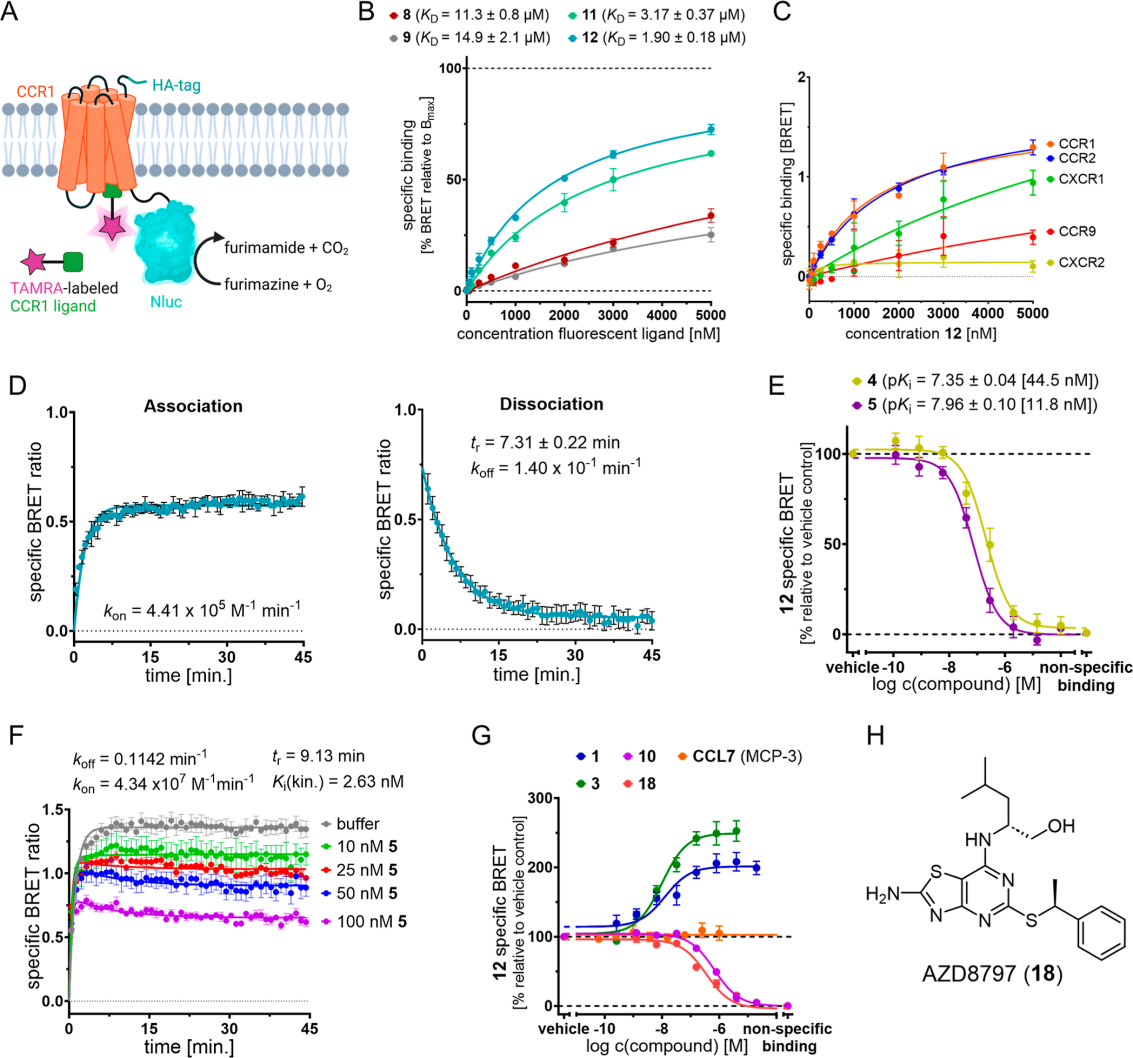
Application of fluorescent ligands for cell-free NanoBRET binding studies targeting the IABS of CCR1. (A) Cartoon representation of the NanoBRET strategy to detect binding to the IABS of CCR1. (B) Specific saturation binding curve of fluorescent ligands **8**–**9** and **11**–**12** in a NanoBRET-based assay using CCR1_Nluc membranes (mean ± SEM, triplicate measurement, *n* ≥ 3). See Figure S2E–H for curves for total and nonspecific binding. (C) Comparison of representative specific binding curves of the fluorescent ligand **12** binding to membrane preparations from HEK293T cells expressing the respective C-terminally Nluc-tagged chemokine receptors CCR1, CCR2, CCR9, CXCR1, and CXCR2. The experiments were performed in triplicate (*n* ≥ 3). See Figure S5 for representative curves for total, specific, and nonspecific binding of **12**. (D) Representative association and dissociation curves with **12** (1000 nM) using CCR1_Nluc membranes. Further information about kinetic binding studies is provided in Table S1. (E) Competition binding curves and detected p*K*_i_ values (mean ± SEM, triplicate measurement, *n* = 4) for the known intracellular CCR1 inhibitors **4** (dark yellow) and **5** (dark purple), obtained with **12** (2000 nM) and CCR1_Nluc membranes. The *K*_i_ values are given in square brackets. (F) Representative kinetic competition binding curves (mean ± SEM, triplicate measurement, *n* = 4) for **5**, obtained with **12** (1000 nM) and CCR1_Nluc membranes. (G) Binding curves of the allosteric extracellular CCR1 inhibitor BX-471^16^ (**1**, dark blue, *n* = 5), the CCR1 antagonist BI-639667^26^ (**3**, dark green, *n* = 3) with a previously unknown binding site, the extracellular orthosteric agonist CCL7 (orange, *n* = 4), the ligand-linker conjugate V (**10**, purple, *n* = 3), and the CX3CR1 antagonist AZD8797 (**18**, salmon, *n* = 3)^54^ with reported off-target binding to CCR1, obtained with **12** (2000 nM) and CCR1_Nluc membranes (mean ± SEM, triplicate measurement). (H) Chemical structure of AZD8797 (**18**).

To evaluate the CCR1 affinity of previously reported (**8**–**9**) and new fluorescently labeled pyrrolones (**11**–**12**), we developed a NanoBRET-based binding assay (Figures 4A–H and S2A–J). To this end, we used a previously reported CCR1 construct (hereafter referred to as CCR1_Nluc) with a small and bright luciferase variant (nanoluciferase,^52^ Nluc), fused to the intracellular C-terminus of CCR1 (Figures 4A and S2A).^47^ In saturation binding experiments using membranes from HEK293T cells transiently expressing CCR1_Nluc, **12** showed the highest affinity with a *K*_D_ value of 1.90 ± 0.18 μM (Figures 4B and S2E–H). A slightly lower affinity was detected for its chloro analogue **11** (*K*_D_ = 3.17 ± 0.37 μM), which is consistent with the slightly weaker affinity reported for the chlorinated parent ligand **4**, compared to **5** (Figure 1).^33^ For both fluorescent probes with short linkers (**8**–**9**), only weak affinities with *K*_D_ values in the two-digit micromolar range were detected (for **8**: *K*_D_ = 11.3 ± 0.8 μM; for **9**: *K*_D_ = 14.9 ± 2.1 μM), thereby indicating that a longer linker contributes to CCR1 affinity, possibly by avoiding steric clashes of the TAMRA fluorophore with amino acid residues at the entrance of the binding pocket (Figure S3). Initial selectivity studies with **11** and **12** revealed that they also bind with similar affinities to the closely related CCR2 (for **11**: *K*_D_(CCR2) = 1.98 ± 0.27 μM; for **12**: *K*_D_(CCR2) = 1.69 ± 0.31 μM; see Figure S4). This was expected, given the very low CCR2/CCR1 selectivity of the parental ligands **4** and **5** (Figure 1).^33^ For further studies, we selected **12** due to its highest CCR1 affinity. In a broader selectivity screening, **12** also showed binding to CXCR1 (*K*_D_(CXCR1) = 12.30 ± 0.30 μM). Binding signals of **12** for other chemokine receptors with a previously identified druggable IABS (*e.g.*, CCR9 and CXCR2)^35,37^ were much weaker (Figures 4C and S5), thus indicating significantly lower affinities of **12** for these receptors.

Kinetic binding studies with **12** revealed a fast association of the ligand–receptor complex by exhibiting a rate constant of *k*_on_ = 4.41 ± 0.43 × 10^5^ M^–1^ min^–1^ (Figure 4D, Table S1). With a rate constant of *k*_off_ = 1.40 ± 0.04 × 10^–1^ min^–1^ and a residence time of *t*_r_ = 7.31 ± 0.22 min, the dissociation was observed to be fast as well. The 6-fold difference between the resulting kinetic *K*_D_ value (*K*_D(kin.)_ = 0.317 ± 0.032 μM) and the equilibrium *K*_D_ value (*K*_D(eq.)_ = 1.90 ± 0.18 μM) might be rationalized by the fact that the *B*_max_ value for the equilibrium *K*_D_ determination could only be extrapolated due to assay interferences at higher tracer concentrations.

Next, we investigated the suitability of **12** as a molecular tool to study the binding of nonfluorescent ligands to the IABS of CCR1. To this end, we set up membrane-based competition experiments with **12** and the known intracellular CCR1 antagonists **5** and **4** (Figures 4E and S2I). In very good agreement with the previously reported affinity of **5** [p*K*_i_ = 7.98 ± 0.04 (11 nM)] from a radioligand binding assay,^33^ we detected full displacement of the fluorescent probe and a p*K*_i_ value of 7.96 ± 0.10 (11.8 nM). A similar but slightly lower affinity was detected for **4** (p*K*_i_ = 7.35 ± 0.04 (44.5 nM), which is consistent with literature data.^33^ Higher affinities for **5** and **4** were detected by applying **12** in a kinetic competition setup [for **5**: p*K*_i(kin.)_ = 8.58 ± 0.08 (2.63 nM), *k*_on_ = 4.34 ± 1.02 × 10^7^ M^–1^ min^–1^, *k*_off_ = 0.1142 ± 0.0062 min^–1^, *t*_r_ = 9.1 ± 0.5 min, Figure 4F; for **4**: p*K*_i(kin.)_ = 8.56 ± 0.09 (2.78 nM), *k*_on_ = 4.76 ± 1.30 × 10^7^ M^–1^ min^–1^, *k*_off_ = 0.1324 ± 0.0065 min^–1^, *t*_r_ = 15.4 ± 0.3 min, Figure S6]. Interestingly, when comparing the kinetic data of the unlabeled CCR1 ligand **5** with the **5**-derived fluorescent probe **12**, it becomes obvious that the attachment of a fluorophore mainly slows down association, whereas dissociation is only affected to a minor extent. To rationalize the design of our fluorescent CCR1 ligand **12** in a retrospective manner, we synthesized and tested the ligand–linker conjugate V (**10**, Scheme S1, Figures 3, 4G, and S2J). Using our equilibrium competition assay, we detected a two-digit nanomolar CCR1 affinity for **10** [p*K*_i_ = 7.01 ± 0.01 (97.6 nM)], thus corroborating the design of **12**. The orthosteric CCR1 agonist CCL7, also referred to as MCP-3, showed no competition with **12** (Figures 4G and S2J), thereby confirming the previously reported noncompetitive binding mode of intracellular allosteric chemokine receptor antagonists.^40,44,45^ Next, we were interested to see if **12** is a suitable tool to distinguish between intracellular allosteric inhibitors binding to the IABS of CCR1 and CCR1 antagonists binding to other binding sites of the receptor. To this end, we tested the CCR1 antagonist BX-471 (**1**),^16^ which is known to bind to an extracellular allosteric ligand binding site of CCR1^53^ and thus should not compete with **12** for receptor binding. As expected, **1** was not able to displace **12** from its intracellular binding site; on the contrary, **1** significantly enhanced the CCR1 binding of **12** by approximately 100% (Figures 4G and S2J). The observed positive cooperativity between **1** and pyrrolone-based intracellular CCR1 antagonists is consistent with reports by Ortiz Zacarías *et al.*, which show that **1** significantly enhances the CCR1 binding of the radioligand [^3^H]-CCR2-RA-[*R*].^33^ For BI-639667 (**3**),^26^ a high-affinity CCR1 antagonist and phase I clinical candidate with an unknown binding site, we observed a very similar behavior (∼150% enhancement of probe binding, Figures 4G and S2J). These results clearly indicate that **3** does not bind to the IABS of CCR1 and suggest an extracellular binding site of **3**. The mechanistic similarity between **3** and the extracellular allosteric antagonist **1**(16,53) was further confirmed by the fact that for both ligands, the positive cooperativity with **12** can also be observed in the presence of the orthosteric agonist CCL7 (200 nM, Figure S7A), thus suggesting no competition with CCL7 and eventually an extracellular allosteric binding site for **3**, as reported for **1**. While massively increasing *B*_max_ of **12**, the presence of **3** (10 μM) has only a minor impact on the binding affinity of **12** as well as on the *K*_i_ values detected by means of our **12**-based NanoBRET competition binding assay (Figure S7B–D). These observations are in full agreement with the noncompetitive relationship between **3** and **12**. For the CX3CR1-targeted phase I clinical candidate AZD8797 (**18**, Figure 4H),^54,55^ pharmacological studies by Cederblad *et al.* suggested an intracellular binding site on the basis of a direct competition with G protein binding.^56^ Because **18** was reported to bind to CCR1 as well,^54^ we were curious to elucidate if this off-target affinity is mediated by the IABS of CCR1. By means of our NanoBRET competition assay, we clearly show that **18** displaces **12** from the IABS of CCR1 [p*K*_i_ = 7.14 ± 0.09 (75.6 nM), Figure 4G], thus highlighting **18** as an intracellular allosteric chemokine receptor antagonist. This further strengthens the hypothesis by Cederbald *et al.*(56) that **18** also binds to the IABS of its target receptor CX3CR1.

**Figure 5:**
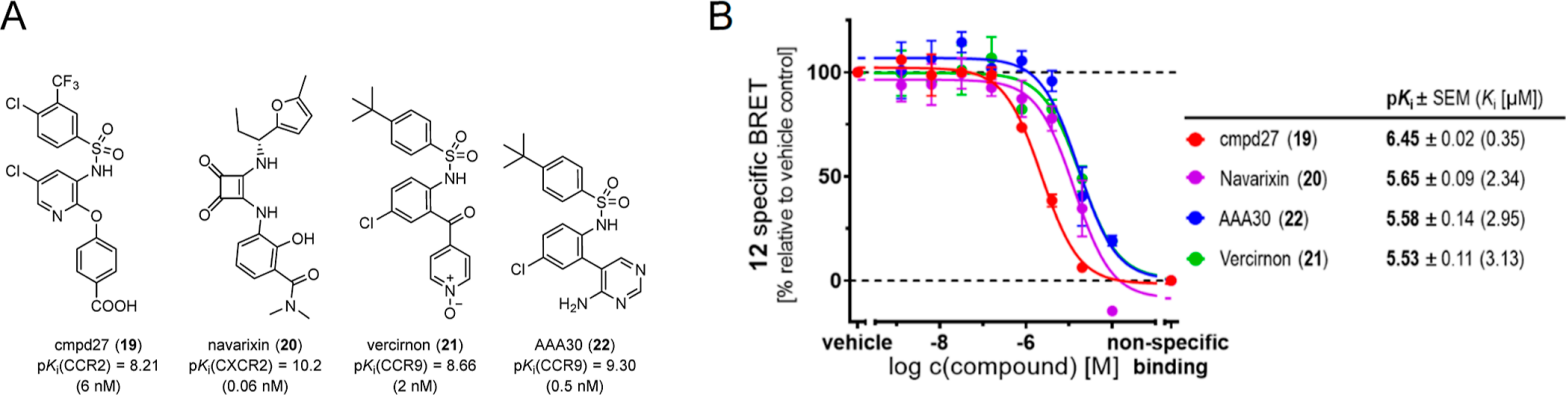
(A) Chemical structures and reported p*K*_i_ values (*K*_i_ values and targets given in brackets) of known intracellular chemokine receptor antagonists **19**–**22**.^44,45,47,57^ (B) Competition binding curves and p*K*_i_ values (mean ± SEM, triplicate measurement, *n* ≥ 3) for **19**–**22**, obtained with **12** (2000 nM) and CCR1_Nluc membranes. For representative competition binding curves of the single experiments, see Figure S8.

After having shown that **12** is a highly valuable tool for mapping CCR1 antagonists to specific binding sites at CCR1, we wanted to study the promiscuity of CCR1’s IABS, especially since the orthosteric chemokine binding site of CCR1 is known to be quite promiscuous. To this end, we tested a selection of known intracellular allosteric antagonists targeting other chemokine receptors for their competition with **12** (Figure 5). All these ligands, including the CCR2-targeted cmpd27 (**19**),^47^ the CXCR2-targeted navarixin (**20**),^44,57^ as well as the CCR9-targeted vercirnon (**21**)^42,45^ and AAA30 (**22**),^45^ which were reported as antagonists with subnanomolar to single-digit nanomolar affinities for their targeted receptor, showed strongly reduced binding to CCR1. Among the tested intracellular chemokine receptor antagonists, the CCR2-targeted biarylsulfonamide cmpd27 (**19**)^47^ showed the highest CCR1 affinity [p*K*_i_ = 6.45 ± 0.02, (354 nM)], which is not surprising since structurally related biarylsulfonamide-based CCR2 antagonists were already reported to bind to CCR1 as well.^33^ In general, these results indicate the potential of the IABS of CCR1 as a target site for the development of selective drugs but also highlight the challenge of achieving CCR1 over CCR2 selectivity and vice versa by targeting the IABS of these receptors.

**Figure 6:**
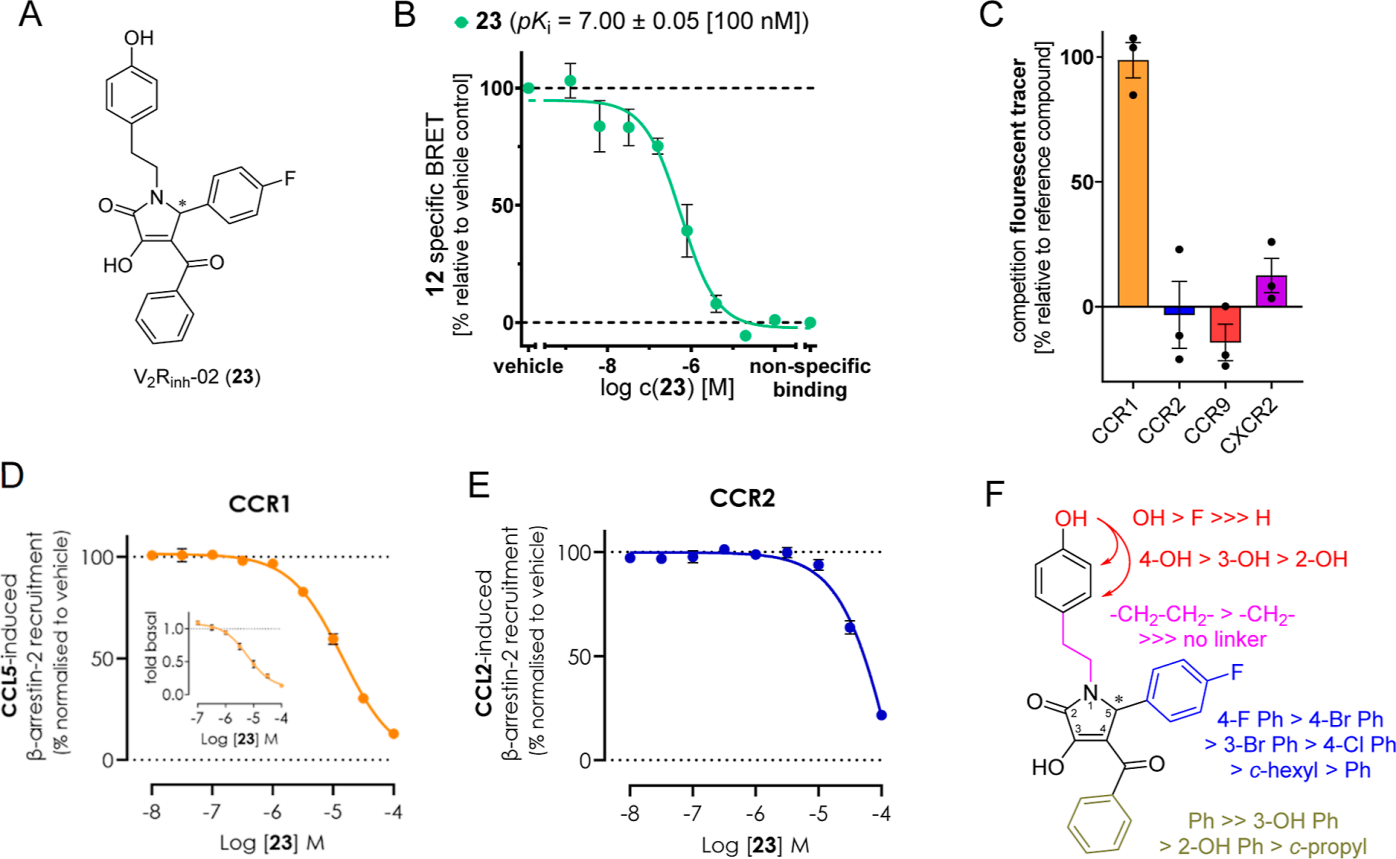
Identification of **23** as a new ligand for selective intracellular CCR1 over CCR2 inhibition. (A) Chemical structure of **23**. (B) Competition binding curve and p*K*_i_ value (mean ± SEM, triplicate measurement, *n* = 4) for **23**, obtained with **12** (2000 nM) and CCR1_Nluc membranes. For a representative competition binding curve from a single experiment, see Figure S9. (C) Selectivity studies with **23** and the chemokine receptors CCR1, CCR2, CCR9, and CXCR2. Percentual inhibition of tracer binding occurred in the presence of **23** (20 μM). For the binding studies with CCR2, CCR9, and CXCR2, we used our recently reported methods to detect ligand binding to the IABS of the respective chemokine receptor.^44,45,47^ Experiments were performed in triplicate (*n* ≥ 3). (D) Concentration–response curve from a cellular CCR1 NanoBiT β-arrestin recruitment assay with **23** in the presence and absence (inset) of CCR1 agonist CCL5 (3 nM). pIC_50_ values (mean ± SEM, *n* = 3). A concentration–response curve for CCL5-mediated CCR1 activation is shown in Figure S10A. (E) Concentration–response curve from a cellular CCR2 NanoBiT β-arrestin recruitment assay with **23** in the presence of CCR2 agonist CCL2 (10 nM). pIC_50_ value (mean ± SEM, *n* = 3). A concentration–response curve for CCL2-mediated CCR2 activation is shown in Figure S10B. (F) Schematic illustration of the SAR model for **23**.

With **6** (Figure 1), a first lead structure for intracellular CCR1 inhibition that features an approximately 20-fold CCR1 over CCR2 selectivity was previously discovered by Ortiz Zacarías *et al.*(33) In order to discover new chemotypes for selective CCR1 over CCR2 inhibition, we aimed to apply our novel NanoBRET competition binding assay for CCR1 in combination with our recently published CCR2 competition binding assay based on **7**.^47^ In our first attempt, we screened a small in-house library enriched with intracellular GPCR ligands and structural analogues thereof. In the course of this screening, we identified compound V_2_R_inh_-02 (**23**), which was originally developed as an antagonist of the vasopressin-2 receptor (V_2_R, *K*_i_ ∼ 70 nM),^58^ as an intracellular CCR1 ligand (Figures 6A and S9A). Not surprisingly, this compound also shares the pyrrolone core of the known intracellular CCR1/CCR2 inhibitors **4**–**5** but features a different substitution pattern. With a p*K*_i_ value of 7.00 ± 0.05 [100 nM] for CCR1 and no significant binding (inh. −3% @20 μM) to CCR2, **23** has a remarkable CCR1 over CCR2 binding selectivity of at least 172-fold (Table 1, Figures 6B,C, S9A and S11B) and a very good solubility (*S*_kin_ = 442 ± 27 μM, Figure S9B). Additionally, we tested **23** for binding to the IABS of CCR9 and CXCR2 using our recently reported NanoBRET-based assay platform (Figure 6C).^44,45^ In the course of these studies, we detected no binding of **23** to CCR9 and CXCR2 up to a concentration of 20 μM. Since cell lineage-dependent effects have been observed for CCR1 antagonists,^59^ we studied the CCR1 over CCR2 selectivity of **23** by using a previously reported radioligand binding assay with membrane preparations from osteosarcoma (U2OS) cells stably expressing human CCR1 or CCR2.^33^ Also under these orthogonal conditions, **23** has a remarkable CCR1 (p*K*_i_ = 6.9 ± 0.05 [117 nM]) over CCR2 (p*K*_i_ = 5.4 ± 0.1 [3821 nM]) binding selectivity of 33-fold (Figure S9C,D). As Ortiz Zacarías *et al.* showed that the binding selectivity of their most CCR1 over CCR2 selective compound **6** (∼20-fold CCR1 over CCR2 selectivity, see Figure 1) did not result in significant selectivity on a functional level (pIC_50_(CCR1) = 5.07 ± 0.05 [8.64 μM], pIC_50_(CCR2) = 5.06 ± 0.05 [8.77 μM]),^33^ we were keen to see whether or not **23** is able to evoke a CCR1 over CCR2 selective inhibition in a functional assay. In agreement with these previous reports,^33^ we observed a reduction in CCR1 over CCR2 selectivity when moving from membrane-based binding assays to cell-based functional assays. Nonetheless, in our cellular NanoBiT β-arrestin recruitment assay, **23** still shows a more that 10-fold CCR1 over CCR2 selectivity (pIC_50_(CCR1) = 4.85 ± 0.01 [14.2 μM], pIC_50_(CCR2) = 3.71 ± 0.01 [186 μM], Figure 6D,E). In addition to agonist-induced β-arrestin recruitment, **23** was also able to inhibit basal β-arrestin recruitment at CCR1 (pIC_50_(CCR1) = 5.21 ± 0.15 [6.00 μM]), thus acting as an inverse CCR1 agonist with respect to β-arrestin recruitment. This special feature of **23** is of particular interest because CCR1 has a high constitutive activity, which is suggested to lead to G protein-independent β-arrestin-mediated internalization.^32^ These results are highly consistent with reports by Ortiz Zacarías *et al.* that characterized their pyrrolone-based intracellular CCR1 ligands **4**–**6** as inverse CCR1 agonists by using a G protein activation assay.^33^ To investigate a potential signaling bias of **23**, we tested the compound by means of a NanoBiT miniGi recruitment assay.^60^ Here, we detected a pIC_50_ value of 4.17 ± 0.14 [68.9 μM], which is in a similar range to the data from the β-arrestin recruitment assay. Thus, this indicated no strong signaling bias of **23** (Figures S9E and S10C). To elucidate the SAR of this new scaffold for selective CCR1 over CCR2 inhibition, we synthesized several analogues of **23** (**24**–**40**) and tested them by means of our NanoBRET competition binding assays (Schemes S2 and S3, Table 1, Figures 6F and S11). First, we aimed to examine the impact of the stereochemistry of **23** on CCR1 affinity. As for the structurally related pyrrolone **4**, large differences in CCR2 affinity between the eutomer **4**-[*R*] and the distomer **4**-[*S*] were described.^61^ The (+)- and (−)-enantiomers of **23**, which were obtained by stereoselective synthesis (Scheme S3), also differed in their CCR1 affinities, with p*K*_i_ values of 7.18 ± 0.01 (65.7 nM) for (+)**-23** and 6.68 ± 0.08 (216 nM) for (−)**-23**, respectively. Furthermore, we identified the 4-hydroxyphenethyl group at position 1 of the pyrrolone scaffold as highly important for CCR1 affinity and selectivity. Its presence seems to block CCR2 binding, also when combined with the structural features of the high-affinity CCR2 ligand **5**, as exemplified by **24**. Especially the 4-hydroxy group at this moiety is essential for CCR1 binding (see **25** and **26**). Shifting this hydroxy group from position 4 to positions 3 and 2, respectively, led to decreased affinities (see **27**, **28**). A shortening of the ethylene bridge by one methylene group was tolerated (see **29**), while a complete removal resulted in an inactive compound (see **30**). In position 5 of the pyrrolone, several aromatic and aliphatic substituents were tolerated (see **31**–**35**). Finally, the benzoyl moiety in position 4 of the pyrrolone was identified to be essential for CCR1 affinity as well because of a replacement by cyclopropanecarbonyl, 4-hydroxybenzoyl, and 3-hydroxybenzoyl massively impaired CCR1 binding (see **36**–**39**).

**Table 1:**
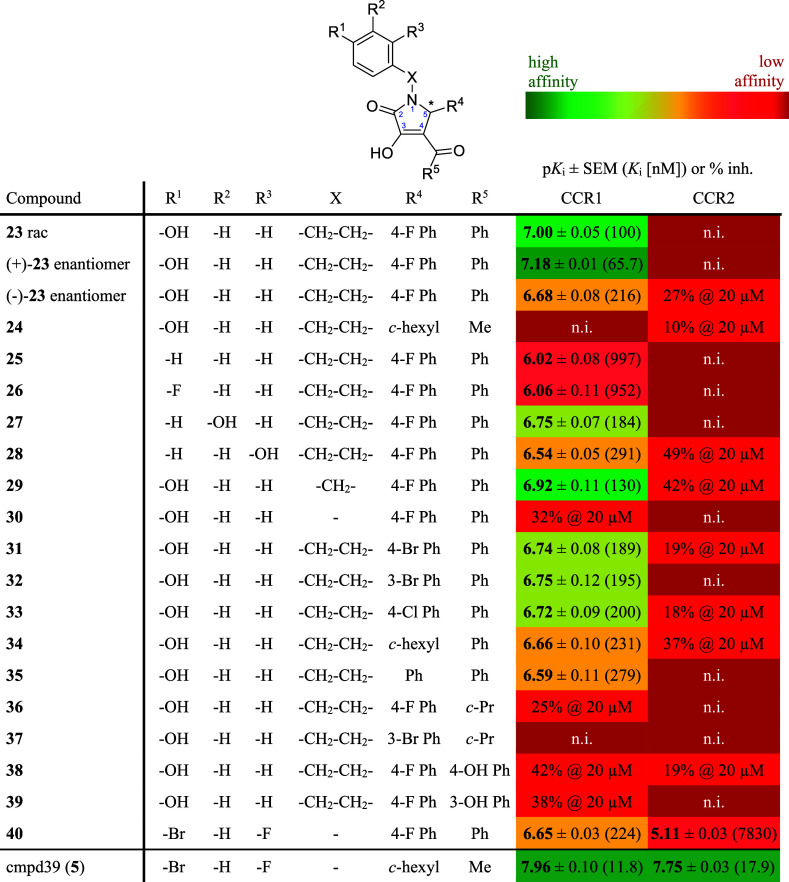
CCR1 and CCR2 Affinity Data for **23** and Its Analogues **24**–**40**^a^

a CCR1 affinity data was obtained by means of our NanoBRET competition binding assay using **12** (2000 nM) and CCR1_Nluc membranes. CCR2 affinity data was obtained by using a NanoBRET competition binding assay, as previously reported.^47^ Experiments were performed in triplicate (*n* = 3). For competition binding curves, see Figure S11. Compound **5** is shown as a reference for a high affinity dual CCR1/CCR2 inhibitor. n.i.: no inhibition (percentual inhibition at 20 μM < 10%). To illustrate the affinity data, a traffic light representation is used; a color scale is given above.

**Figure 7:**
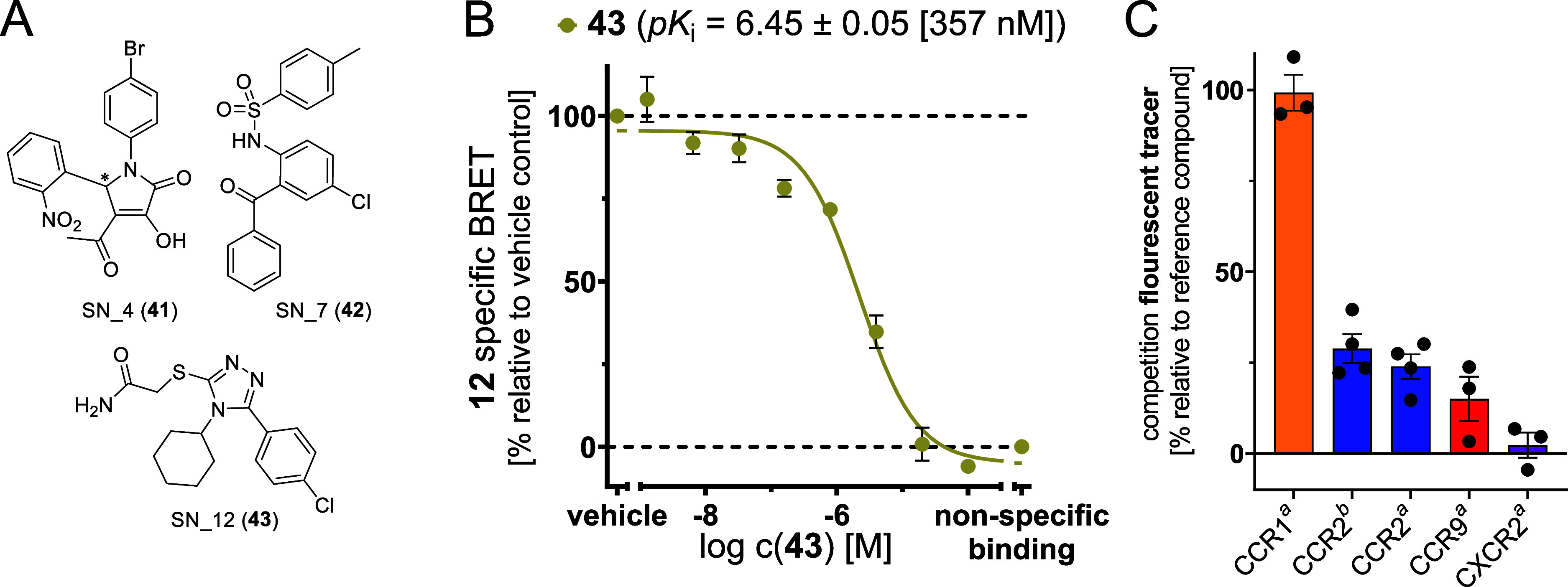
Identification of SN12 (**43**) as a new lead structure for selective intracellular CCR1 over CCR2 inhibition. (A) Chemical structure of hits (**41**–**43**) from a virtual screening campaign. (B) Competition binding curve and p*K*_i_ value (mean ± SEM, triplicate measurement, *n* = 3) for **43**, obtained with **12** (2000 nM) and CCR1_Nluc membranes. For a representative competition binding curve from single experiments, see Figure S15. (C) Selectivity studies with **43** and the chemokine receptors CCR1, CCR2, CCR9, and CXCR2. Percentual inhibition of tracer binding was observed in the presence of **43** (*a* = 20 μM; *b* = 100 μM). For the binding studies with CCR2, CCR9, and CXCR2, we used our recently reported methods to detect ligand binding to the IABS of the respective chemokine receptor.^44,45,47^ Experiments were performed at least in duplicate (*n* ≥ 3).

In a second approach for identifying novel intracellular CCR1 antagonists, we performed a multistep virtual screening, including pharmacophore screening, molecular docking, and protein–ligand interaction fingerprint postdocking filtration. To this end, we adapted a recently published protocol that enabled the discovery of novel CCR5 antagonists.^62^ More detailed information on the virtual screening is given in the Supporting Information. In an initial testing of the 24 hit compounds from the virtual screening campaign (Figures 7 and S12), three compounds [SN_4 (**41**), SN_7 (**42**), and SN_12 (**43**)] showed an inhibition ≥50% at a concentration of 20 μM. Whereas the pyrrolone-based **41** and biarylsulfonamide **42** are structurally related to already known intracellular CCR1/CCR2 antagonists,^33,41^ the 1,2,4-triazole **43** represents a completely novel chemotype for intracellular CCR1 inhibition. Since **43** also evoked the strongest CCR1 inhibition among the virtual screening hits and the predicted docking pose of **43** could nicely be corroborated by molecular dynamics (MD) simulations (Figures S13 and S14), we focused on this compound for further characterization. For this compound, we detected a p*K*_i_ value of 6.45 ± 0.05 (357 nM) for CCR1 and a weaker binding to CCR2 (29% inh. @100 μM), CCR9 (15% inh. @20 μM), and CXCR2 (2% inh. @20 μM), thus indicating selective binding of **43** to the IABS of CCR1.

**Figure 8:**
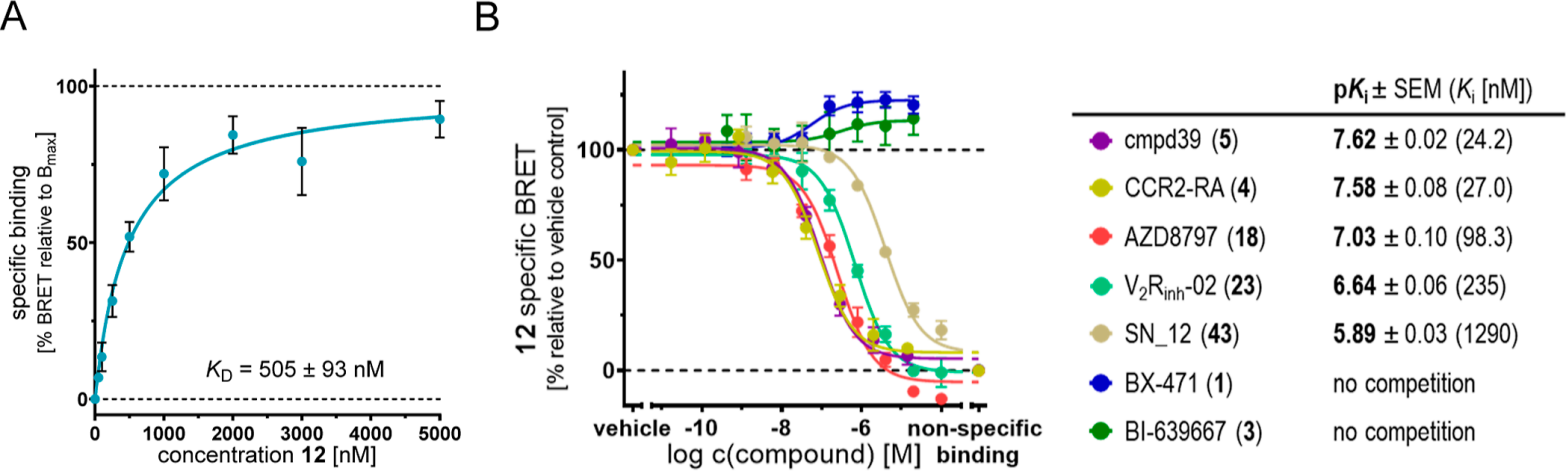
Application of **12** as a fluorescent tracer for a cellular NanoBRET-based CCR1 binding assay using live HEK293T cells expressing CCR1_Nluc. (A) Saturation binding curve (specific binding) of **12** in a cellular NanoBRET-based experiment (mean ± SEM, quadruplicate measurement, *n* = 3). See Figure S16A,B for more detailed information. (B) Competition binding curves and p*K*_i_ values (mean ± SEM, quadruplicate measurement) for BX-471 (**1**, dark blue, *n* = 4), BI-639667 (**3**, dark green, *n* = 4), CCR2-RA (**4**, dark yellow, *n* = 3), cmpd39 (**5**, purple, *n* = 3), AZD8797 (**18**, salmon, *n* = 3), V_2_R_inh_-02 (**23**, sea green, *n* = 4), and SN_12 (**43**, khaki, *n* = 3) obtained with **12** (1000 nM). The *K*_i_ values are given in square brackets. Representative competition binding curves for the single experiments are shown in Figure S16C,D.

Having shown that our fluorescent CCR1 ligand **12** is a suitable molecular tool to study the binding of high- to low-affinity binders to the IABS of CCR1 under cell-free conditions, we aimed to transfer our NanoBRET binding assay to a live cell environment. In general, the assessment of the interactions between a drug and its target protein in a cellular environment is a critical step in preclinical drug discovery.^63^ This step, also called cellular target engagement, is especially relevant in the case of intracellular binding sites, such as the IABS of GPCRs, since compounds need to pass the cell membrane to be suitable candidates for further cellular and *in vivo* evaluation. To our best knowledge, no small-molecule tracer has been developed so far that allows cellular binding assays for the IABS of CCR1. For establishing our cell-based CCR1 binding assay, we used live HEK293T cells transiently expressing CCR1_Nluc. With this cell-based assay setup, we determined a *K*_D_ value of 505 ± 93 nM for **12** (Figures 8A and S16A,B). This demonstrates that **12** is able to pass through the cell membrane and bind to CCR1 at the intracellular face of the receptor. By applying **12** in a cell-based competition binding assay, we determined p*K*_i_ values of 7.62 ± 0.02 (24.2 nM) and 7.58 ± 0.08 (27.0 nM) for the literature-known intracellular CCR1 antagonists **5** and **4**, respectively, which is in good agreement with the *K*_i_ value detected in our cell-free setup. For compounds AZD8797 [**18**, p*K*_i_ = 7.03 ± 0.10 (98.3 nM)], **23** [p*K*_i_ = 6.64 ± 0.06 (235 nM)], and **43** [p*K*_i_ = 5.89 ± 0.03 (1290 nM)], which were identified as IABS-targeted CCR1 ligands in the course of this study, we were able to detect target engagement in a live cell environment as well. Using our cell-based assay, we also detected a positive cooperativity between **12** and the extracellular CCR1 antagonists BX-471 (**1**) and BI-639667 (**3**); however, this was much less pronounced compared to the data from cell-free studies. This might be explained by different levels of active versus inactive state conformations of CCR1 when comparing cell-free and cell-based conditions. Overall, the results from live cell NanoBRET and membrane-based experiments are in very good agreement with each other, thus highlighting the suitability of **12** as a molecular tracer to determine cellular target engagement for the IABS of CCR1.

In summary, starting from the pyrrolone-based CCR1/CCR2 inhibitors **4** and **5**, we developed the first small-molecule-based fluorescent probes targeting the IABS of CCR1. In combination with a cell-free NanoBRET-based setup, our fluorescent CCR1 probe **12** (*K*_D(eq.)_ = 1.90 ± 0.18 μM; *K*_D(kin.)=_ 0.317 ± 0.032 μM) enabled equilibrium as well as kinetic binding studies. Selectivity studies with other chemokine receptors, which are known to feature a druggable IABS (*i.e.*, CCR2, CCR9, CXCR1, and CXCR2), indicated that **12** binds to CCR2 and CXCR1 but shows much weaker binding to CCR9 and CXCR2. Applying **12** as a molecular tool to study the binding of nonfluorescent compounds, we were able to (i) reproduce the reported affinity data for the reference ligand **5**; (ii) map known CCR1 antagonists with an unknown mode of action to specific binding sites of the receptor; (iii) identify novel chemotypes for selective intracellular CCR1 over CCR2 inhibition. For our new pyrrolone-based lead structure **23** that showed remarkable CCR1 over CCR2 binding selectivity, we used our novel CCR1 NanoBRET assay to establish an initial SAR model, highlighting the importance of the 4-hydroxyphenethyl group in position 1 of the pyrrolone scaffold for CCR1 affinity and selectivity. In functional assays on β-arrestin recruitment, we observed a strong reduction in CCR1 over CCR2 selectivity for **23**, as compared with binding assays. Nevertheless, **23** still shows more than 10-fold CCR1 over CCR2 selectivity in the β-arrestin recruitment assay in living cells and is therefore, to our best knowledge, the first intracellular CCR1 ligand that features statistically significant CCR1 over CCR2 selectivity on a functional level. Additionally, our functional assays revealed that **23** inhibits basal β-arrestin recruitment to CCR1 and thus can be considered as an inverse agonist with respect to β-arrestin recruitment. This is especially interesting because CCR1 is known to be constitutively active, which is proposed to lead to G protein-independent, β-arrestin-mediated internalization.^32^ However, when using **23** as a tool compound for selective CCR1 over CCR2 inhibition, it should be noted that the observed effects might also be mediated *via* an inhibition of V_2_R (*K*_i_ ∼ 70 nM),^58^ if this receptor is present under the applied test conditions. A removal of the V_2_R affinity of **23** while retaining its ability to evoke a selective CCR1 over CCR2 inhibition will be part of future studies. Finally, we show that our fluorescent CCR1 probe **12** can be utilized to study cellular target engagement for the IABS of CCR1 *via* a cell-based NanoBRET assay. Using this setup, we were also able to demonstrate cellular target engagement for newly discovered CCR1-targeted chemotypes **23** and **43**. As the IABS of CCR1 is a highly promising target site for the development of anti-inflammatory and immune modulatory drugs, respectively, our fluorescent tracer **12** represents a very important tool compound for future studies investigating the therapeutic potential of intracellular CCR1 ligands.

## Data Availability

All data analyzed during this study are included within the manuscript (and its Supporting Information). Data deposition does not apply to the current study.

## Supporting Information Available

Experimental procedures for compound synthesis, identification of suitable linker fragments and linker lengths for the design of fluorescently labeled CCR1 ligands based on the scaffolds of CCR2-RA (**4**) and cmpd39 (**5**), development of a cell-free NanoBRET-based binding assay for CCR1, selectivity studies, MD simulation studies, structure–activity relationship studies, synthesis of the ligand–linker conjugate V (**10**), synthesis of analogues **24**–**40** derived from the newly discovered intracellular CCR1 antagonist **23**, stereoselective synthesis, kinetic parameters detected for the interaction between **12** and CCR1 by using our membrane-based NanoBRET assay, NMR spectra, and HPLC chromatograms (PDF)

## Abbreviations

AD: Alzheimer’s disease
BRET: bioluminescence resonance energy transfer
CCL: chemokine (C–C motif) ligand
CCR: CC chemokine receptor
COVID-19: coronavirus disease 2019
cryo-EM: cryogenic-electron microscopy
CuAAC: Cu(I)-catalyzed azide-alkyne cycloaddition
CXCR: CXC chemokine receptor
CX3CR: CX3C motif chemokine receptor
DIPEA: *N*,*N*-diisopropylethylamine
GPCRs: G protein-coupled receptors
HEK: human embryonic kidney
IABS: intracellular allosteric binding site
MCP-3: monocyte chemotactic protein 3
MD: molecular dynamics
MM: multiple myeloma
MS: multiple sclerosis
NanoBiT: NanoLuc binary technology
Nluc: NanoLuc luciferase
PDB: protein data bank
PEG: polyethylene glycol
RA: rheumatoid arthritis
SAR: structure-affinity relationship
SEM: standard error of the mean
TAMRA: tetramethylrhodamine
TBTU: 2-(1*H*-benzotriazole-1-yl)-1,1,3,3-tetramethylaminium tetrafluoroborate
TBTA: tris(benzyltriazolylmethyl)amine
*t*_r_: residence time
U2OS: human osteosarcoma cells
V_2_R: vasopressin-2 receptor
β_2_AR: beta-2 adrenergic receptor

## References

[ref1] Griffith J. W.; Sokol C. L.; Luster A. D. Chemokines and chemokine receptors: positioning cells for host defense and immunity. Annu. Rev. Immunol. 2014, 32, 659–702. 10.1146/annurev-immunol-032713-120145. 24655300

[ref2] Stone M. J.; Hayward J. A.; Huang C.; Huma Z. E.; Sanchez J. Mechanisms of regulation of the chemokine-receptor network. Int. J. Mol. Sci. 2017, 18, 342 10.3390/ijms18020342. 28178200 PMC5343877

[ref3] Murphy P. M.; Baggiolini M.; Charo I. F.; Hebert C. A.; Horuk R.; Matsushima K.; Miller L. H.; Oppenheim J. J.; Power C. A. International union of pharmacology. XXII. Nomenclature for chemokine receptors. Pharmacol. Rev. 2000, 52, 145–176. 10699158

[ref4] Schall T. J.; Proudfoot A. E. Overcoming hurdles in developing successful drugs targeting chemokine receptors. Nat. Rev. Immunol. 2011, 11, 355–363. 10.1038/nri2972. 21494268

[ref5] Shao Z.; Shen Q.; Yao B.; Mao C.; Chen L. N.; Zhang H.; Shen D. D.; Zhang C.; Li W.; Du X.; Li F.; Ma H.; Chen Z. H.; Xu H. E.; Ying S.; Zhang Y.; Shen H. Identification and mechanism of G protein-biased ligands for chemokine receptor CCR1. Nat. Chem. Biol. 2022, 18, 264–271. 10.1038/s41589-021-00918-z. 34949837 PMC8885419

[ref6] Haringman J. J.; Kraan M. C.; Smeets T. J. M.; Zwinderman K. H.; Tak P. P. Chemokine blockade and chronic inflammatory disease: proof of concept in patients with rheumatoid arthritis. Ann. Rheum. Dis. 2003, 62, 715–721. 10.1136/ard.62.8.715. 12860725 PMC1754636

[ref7] Amat M.; Benjamim C. F.; Williams L. M.; Prats N.; Terricabras E.; Beleta J.; Kunkel S. L.; Godessart N. Pharmacological blockade of CCR1 ameliorates murine arthritis and alters cytokine networks in vivo. Br. J. Pharmacol. 2006, 149, 666–675. 10.1038/sj.bjp.0706912. 17016504 PMC2014657

[ref8] Mahad D. J.; Trebst C.; Kivisakk P.; Staugaitis S. M.; Tucky B.; Wei T.; Lucchinetti C. F.; Lassmann H.; Ransohoff R. M. Expression of chemokine receptors CCR1 and CCR5 reflects differential activation of mononuclear phagocytes in pattern II and pattern III multiple sclerosis lesions. J. Neuropathol. Exp. Neurol. 2004, 63, 262–273. 10.1093/jnen/63.3.262. 15055450

[ref9] Halks-Miller M.; Schroeder M. L.; Haroutunian V.; Moenning U.; Rossi M.; Achim C.; Purohit D.; Mahmoudi M.; Horuk R. CCR1 is an early and specific marker of Alzheimer’s disease. Ann. Neurol. 2003, 54, 638–646. 10.1002/ana.10733. 14595653

[ref10] Vallet S.; Raje N.; Ishitsuka K.; Hideshima T.; Podar K.; Chhetri S.; Pozzi S.; Breitkreutz I.; Kiziltepe T.; Yasui H.; Ocio E. M.; Shiraishi N.; Jin J.; Okawa Y.; Ikeda H.; Mukherjee S.; Vaghela N.; Cirstea D.; Ladetto M.; Boccadoro M.; Anderson K. C. MLN3897, a novel CCR1 inhibitor, impairs osteoclastogenesis and inhibits the interaction of multiple myeloma cells and osteoclasts. Blood 2007, 110, 3744–3752. 10.1182/blood-2007-05-093294. 17715391 PMC2077320

[ref11] Lionakis M. S.; Albert N. D.; Swamydas M.; Lee C. R.; Loetscher P.; Kontoyiannis D. P. Pharmacological blockade of the chemokine receptor CCR1 protects mice from systemic candidiasis of hematogenous origin. Antimicrob. Agents Chemother. 2017, 61, e02365–16 10.1128/AAC.02365-16. 27993850 PMC5328547

[ref12] Conroy M. J.; Galvin K. C.; Kavanagh M. E.; Mongan A. M.; Doyle S. L.; Gilmartin N.; O’Farrelly C.; Reynolds J. V.; Lysaght J. CCR1 antagonism attenuates T cell trafficking to omentum and liver in obesity-associated cancer. Immunol. Cell Biol. 2016, 94, 531–537. 10.1038/icb.2016.26. 27046081

[ref13] Gilchrist A.; Echeverria S. L. Targeting chemokine receptor CCR1 as a potential therapeutic approach for multiple myeloma. Front. Endocrinol. (Lausanne) 2022, 13, 846310 10.3389/fendo.2022.846310. 35399952 PMC8991687

[ref14] Sayeed H. M.; Lee E. S.; Byun H. O.; Sohn S. The role of CCR1 and therapeutic effects of anti-CCL3 antibody in herpes simplex virus-induced Behcet’s disease mouse model. Immunology 2019, 158, 206–218. 10.1111/imm.13102. 31393598 PMC6797864

[ref15] Chua R. L.; Lukassen S.; Trump S.; Hennig B. P.; Wendisch D.; Pott F.; Debnath O.; Thurmann L.; Kurth F.; Volker M. T.; Kazmierski J.; Timmermann B.; Twardziok S.; Schneider S.; Machleidt F.; Muller-Redetzky H.; Maier M.; Krannich A.; Schmidt S.; Balzer F.; Liebig J.; Loske J.; Suttorp N.; Eils J.; Ishaque N.; Liebert U. G.; von Kalle C.; Hocke A.; Witzenrath M.; Goffinet C.; Drosten C.; Laudi S.; Lehmann I.; Conrad C.; Sander L. E.; Eils R. COVID-19 severity correlates with airway epithelium-immune cell interactions identified by single-cell analysis. Nat. Biotechnol. 2020, 38, 970–979. 10.1038/s41587-020-0602-4. 32591762

[ref16] Liang M.; Mallari C.; Rosser M.; Ng H. P.; May K.; Monahan S.; Bauman J. G.; Islam I.; Ghannam A.; Buckman B.; Shaw K.; Wei G. P.; Xu W.; Zhao Z.; Ho E.; Shen J.; Oanh H.; Subramanyam B.; Vergona R.; Taub D.; Dunning L.; Harvey S.; Snider R. M.; Hesselgesser J.; Morrissey M. M.; Perez H. D.; et al. Identification and characterization of a potent, selective, and orally active antagonist of the CC chemokine receptor-1. J. Biol. Chem. 2000, 275, 19000–19008. 10.1074/jbc.M001222200. 10748002

[ref17] Naya A.; Sagara Y.; Ohwaki K.; Saeki T.; Ichikawa D.; Iwasawa Y.; Noguchi K.; Ohtake N. Design, synthesis, and discovery of a novel CCR1 antagonist. J. Med. Chem. 2001, 44, 1429–1435. 10.1021/jm0004244. 11311066

[ref18] Revesz L.; Bollbuck B.; Buhl T.; Eder J.; Esser R.; Feifel R.; Heng R.; Hiestand P.; Jachez-Demange B.; Loetscher P.; Sparrer H.; Schlapbach A.; Waelchli R. Novel CCR1 antagonists with oral activity in the mouse collagen induced arthritis. Bioorg. Med. Chem. Lett. 2005, 15, 5160–5164. 10.1016/j.bmcl.2005.08.057. 16198561

[ref19] Horuk R.; Shurey S.; Ng H. P.; May K.; Bauman J. G.; Islam I.; Ghannam A.; Buckman B.; Wei G. P.; Xu W.; Liang M.; Rosser M.; Dunning L.; Hesselgesser J.; Snider R. M.; Morrissey M. M.; Perez H. D.; Green C. CCR1-specific non-peptide antagonist: efficacy in a rabbit allograft rejection model. Immunol. Lett. 2001, 76, 193–201. 10.1016/S0165-2478(01)00172-9. 11306147

[ref20] Gladue R. P.; Cole S. H.; Roach M. L.; Tylaska L. A.; Nelson R. T.; Shepard R. M.; McNeish J. D.; Ogborne K. T.; Neote K. S. The human specific CCR1 antagonist CP-481,715 inhibits cell infiltration and inflammatory responses in human CCR1 transgenic mice. J. Immunol. 2006, 176, 3141–3148. 10.4049/jimmunol.176.5.3141. 16493073

[ref21] Zhang P.; Pennell A. M. K.; Wright J. K. J.; Chen W.; Leleti M. R.; Li Y.; Li L.; Xu Y.; Gleason M. M.; Zeng Y.; Greenman K. L. Preparation of pyrazolopyridylacetylpiperazinylbenzenes as CCR1 chemokine receptor antagonists. WO 2008147822 A1, 2008.

[ref22] Santella J. B. 3rd; Gardner D. S.; Duncia J. V.; Wu H.; Dhar M.; Cavallaro C.; Tebben A. J.; Carter P. H.; Barrish J. C.; Yarde M.; Briceno S. W.; Cvijic M. E.; Grafstrom R. R.; Liu R.; Patel S. R.; Watson A. J.; Yang G.; Rose A. V.; Vickery R. D.; Caceres-Cortes J.; Caporuscio C.; Camac D. M.; Khan J. A.; An Y.; Foster W. R.; Davies P.; Hynes J. Jr. Discovery of the CCR1 antagonist, BMS-817399, for the treatment of rheumatoid arthritis. J. Med. Chem. 2014, 57, 7550–7564. 10.1021/jm5003167. 25101488

[ref23] Pennell A. M.; Aggen J. B.; Sen S.; Chen W.; Xu Y.; Sullivan E.; Li L.; Greenman K.; Charvat T.; Hansen D.; Dairaghi D. J.; Wright J. K.; Zhang P. 1-(4-Phenylpiperazin-1-yl)-2-(1H-pyrazol-1-yl)ethanones as novel CCR1 antagonists. Bioorg. Med. Chem. Lett. 2013, 23, 1228–1231. 10.1016/j.bmcl.2013.01.005. 23374868

[ref24] Hossain N.; Mensonides-Harsema M.; Cooper M. E.; Eriksson T.; Ivanova S.; Bergstrom L. Structure activity relationships of fused bicyclic and urea derivatives of spirocyclic compounds as potent CCR1 antagonists. Bioorg. Med. Chem. Lett. 2014, 24, 108–112. 10.1016/j.bmcl.2013.11.062. 24332486

[ref25] Zhang P.; Dairaghi D. J.; Jaen J. C.; Powers J. P. Recent advances in the discovery and development of CCR1 antagonists. Annu. Rep. Med. Chem. 2013, 48, 133–147. 10.1016/B978-0-12-417150-3.00010-7.

[ref26] Harcken C.; Kuzmich D.; Cook B.; Mao C.; Disalvo D.; Razavi H.; Swinamer A.; Liu P.; Zhang Q.; Kukulka A.; Skow D.; Patel M.; Patel M.; Fletcher K.; Sherry T.; Joseph D.; Smith D.; Canfield M.; Souza D.; Bogdanffy M.; Berg K.; Brown M. Identification of novel azaindazole CCR1 antagonist clinical candidates. Bioorg. Med. Chem. Lett. 2019, 29, 441–448. 10.1016/j.bmcl.2018.12.024. 30595446

[ref27] Harcken C.; Sarko C.; Mao C.; Lord J.; Raudenbush B.; Razavi H.; Liu P.; Swinamer A.; Disalvo D.; Lee T.; Lin S.; Kukulka A.; Grbic H.; Patel M.; Patel M.; Fletcher K.; Joseph D.; White D.; Amodeo L.; Berg K.; Brown M.; Thomson D. S. Discovery and optimization of pyrazole amides as antagonists of CCR1. Bioorg. Med. Chem. Lett. 2019, 29, 435–440. 10.1016/j.bmcl.2018.11.015. 30455146

[ref28] Norman P. AZD-4818, a chemokine CCR1 antagonist: WO 2008103126 and WO2009011653. Expert Opin. Ther. Pat. 2009, 19, 1629–1633. 10.1517/13543770903118996. 19586423

[ref29] P Gladue R.; F Brown M.; H Zwillich S. CCR1 antagonists: what have we learned from clinical trials. Curr. Top. Med. Chem. 2010, 10, 1268–1277. 10.2174/156802610791561237. 20536425

[ref30] Szekanecz Z.; Koch A. E. Successes and failures of chemokine-pathway targeting in rheumatoid arthritis. Nat. Rev. Rheumatol. 2016, 12, 5–13. 10.1038/nrrheum.2015.157. 26607389

[ref31] Zipp F.; Hartung H. P.; Hillert J.; Schimrigk S.; Trebst C.; Stangel M.; Infante-Duarte C.; Jakobs P.; Wolf C.; Sandbrink R.; Pohl C.; Filippi M. Blockade of chemokine signaling in patients with multiple sclerosis. Neurology 2006, 67, 1880–1883. 10.1212/01.wnl.0000244420.68037.86. 17130431

[ref32] Gilliland C. T.; Salanga C. L.; Kawamura T.; Trejo J.; Handel T. M. The Chemokine Receptor CCR1 Is Constitutively Active, Which Leads to G Protein-independent, β-Arrestin-mediated Internalization. J. Biol. Chem. 2013, 288, 32194–32210. 10.1074/jbc.M113.503797. 24056371 PMC3820859

[ref33] Ortiz Zacarías N. V.; van Veldhoven J. P. D.; Portner L.; van Spronsen E.; Ullo S.; Veenhuizen M.; van der Velden W. J. C.; Zweemer A. J. M.; Kreekel R. M.; Oenema K.; Lenselink E. B.; Heitman L. H.; Ijzerman A. P. Pyrrolone derivatives as intracellular allosteric modulators for chemokine receptors: selective and dual-targeting inhibitors of CC chemokine receptors 1 and 2. J. Med. Chem. 2018, 61, 9146–9161. 10.1021/acs.jmedchem.8b00605. 30256641 PMC6328288

[ref34] Zheng Y.; Qin L.; Zacarias N. V.; de Vries H.; Han G. W.; Gustavsson M.; Dabros M.; Zhao C.; Cherney R. J.; Carter P.; Stamos D.; Abagyan R.; Cherezov V.; Stevens R. C.; Ijzerman A. P.; Heitman L. H.; Tebben A.; Kufareva I.; Handel T. M. Structure of CC chemokine receptor 2 with orthosteric and allosteric antagonists. Nature 2016, 540, 458–461. 10.1038/nature20605. 27926736 PMC5159191

[ref35] Liu K.; Wu L.; Yuan S.; Wu M.; Xu Y.; Sun Q.; Li S.; Zhao S.; Hua T.; Liu Z. J. Structural basis of CXC chemokine receptor 2 activation and signalling. Nature 2020, 585, 135–140. 10.1038/s41586-020-2492-5. 32610344

[ref36] Jaeger K.; Bruenle S.; Weinert T.; Guba W.; Muehle J.; Miyazaki T.; Weber M.; Furrer A.; Haenggi N.; Tetaz T.; Huang C. Y.; Mattle D.; Vonach J. M.; Gast A.; Kuglstatter A.; Rudolph M. G.; Nogly P.; Benz J.; Dawson R. J. P.; Standfuss J. Structural basis for allosteric ligand recognition in the human CC chemokine receptor 7. Cell 2019, 178, 1222–1230.e10. 10.1016/j.cell.2019.07.028. 31442409 PMC6709783

[ref37] Oswald C.; Rappas M.; Kean J.; Dore A. S.; Errey J. C.; Bennett K.; Deflorian F.; Christopher J. A.; Jazayeri A.; Mason J. S.; Congreve M.; Cooke R. M.; Marshall F. H. Intracellular allosteric antagonism of the CCR9 receptor. Nature 2016, 540, 462–465. 10.1038/nature20606. 27926729

[ref38] Liu X.; Ahn S.; Kahsai A. W.; Meng K. C.; Latorraca N. R.; Pani B.; Venkatakrishnan A. J.; Masoudi A.; Weis W. I.; Dror R. O.; Chen X.; Lefkowitz R. J.; Kobilka B. K. Mechanism of intracellular allosteric β2AR antagonist revealed by X-ray crystal structure. Nature 2017, 548, 480–484. 10.1038/nature23652. 28813418 PMC5818265

[ref39] Ortiz Zacarias N. V.; Lenselink E. B.; Ijzerman A. P.; Handel T. M.; Heitman L. H. Intracellular receptor modulation: novel approach to target GPCRs. Trends Pharmacol. Sci. 2018, 39, 547–559. 10.1016/j.tips.2018.03.002. 29653834 PMC7048003

[ref40] Zweemer A. J.; Nederpelt I.; Vrieling H.; Hafith S.; Doornbos M. L.; de Vries H.; Abt J.; Gross R.; Stamos D.; Saunders J.; Smit M. J.; Ijzerman A. P.; Heitman L. H. Multiple binding sites for small-molecule antagonists at the CC chemokine receptor 2. Mol. Pharmacol. 2013, 84, 551–561. 10.1124/mol.113.086850. 23877010

[ref41] Zweemer A. J. M.; Bunnik J.; Veenhuizen M.; Miraglia F.; Lenselink E. B.; Vilums M.; de Vries H.; Gibert A.; Thiele S.; Rosenkilde M. M.; Ijzerman A. P.; Heitman L. H. Discovery and mapping of an intracellular antagonist binding site at the chemokine receptor CCR2. Mol. Pharmacol. 2014, 86, 358–368. 10.1124/mol.114.093328. 25024169

[ref42] Walters M. J.; Wang Y.; Lai N.; Baumgart T.; Zhao B. N.; Dairaghi D. J.; Bekker P.; Ertl L. S.; Penfold M. E.; Jaen J. C.; Keshav S.; Wendt E.; Pennell A.; Ungashe S.; Wei Z.; Wright J. J.; Schall T. J. Characterization of CCX282-B, an orally bioavailable antagonist of the CCR9 chemokine receptor, for treatment of inflammatory bowel disease. J. Pharmacol. Exp. Ther. 2010, 335, 61–69. 10.1124/jpet.110.169714. 20660125

[ref43] Burford N. T.; Watson J.; Bertekap R.; Alt A. Strategies for the identification of allosteric modulators of G-protein-coupled receptors. Biochem. Pharmacol. 2011, 81, 691–702. 10.1016/j.bcp.2010.12.012. 21184747

[ref44] Huber M. E.; Wurnig S.; Toy L.; Weiler C.; Merten N.; Kostenis E.; Hansen F. K.; Schiedel M. Fluorescent ligands enable target engagement studies for the intracellular allosteric binding site of the chemokine receptor CXCR2. J. Med. Chem. 2023, 66, 9916–9933. 10.1021/acs.jmedchem.3c00769. 37463496 PMC10388362

[ref45] Huber M. E.; Toy L.; Schmidt M. F.; Vogt H.; Budzinski J.; Wiefhoff M. F. J.; Merten N.; Kostenis E.; Weikert D.; Schiedel M. A chemical biology toolbox targeting the intracellular binding site of CCR9: fluorescent ligands, new drug leads and PROTACs. Angew. Chem., Int. Ed. 2022, 61, e202116782 10.1002/anie.202116782. PMC9306553 34936714

[ref46] Huber M. E.; Toy L.; Schmidt M. F.; Weikert D.; Schiedel M. Small molecule tools to study cellular target engagement for the intracellular allosteric binding site of GPCRs. Chem.—Eur. J. 2023, 29, e202202565 10.1002/chem.202202565. 36193681 PMC10100284

[ref47] Toy L.; Huber M. E.; Schmidt M. F.; Weikert D.; Schiedel M. Fluorescent ligands targeting the intracellular allosteric binding site of the chemokine receptor CCR2. ACS Chem. Biol. 2022, 17, 2142–2152. 10.1021/acschembio.2c00263. 35838163

[ref48] Casella B. M.; Farmer J. P.; Nesheva D. N.; Williams H. E. L.; Charlton S. J.; Holliday N. D.; Laughton C. A.; Mistry S. N. Design, synthesis, and application of fluorescent ligands targeting the intracellular allosteric binding site of the CXC chemokine receptor 2. J. Med. Chem. 2023, 66, 12911–12930. 10.1021/acs.jmedchem.3c00849. 37523859 PMC10544029

[ref49] Huisgen R. 1,3-Dipolar cycloadditions. Proc. Chem. Soc. London, 1961; pp 357–396.

[ref50] Tornoe C. W.; Christensen C.; Meldal M. Peptidotriazoles on solid phase: [1,2,3]-triazoles by regiospecific copper(i)-catalyzed 1,3-dipolar cycloadditions of terminal alkynes to azides. J. Org. Chem. 2002, 67, 3057–3064. 10.1021/jo011148j. 11975567

[ref51] Rostovtsev V. V.; Green L. G.; Fokin V. V.; Sharpless K. B. A stepwise huisgen cycloaddition process: copper(I)-catalyzed regioselective ″ligation″ of azides and terminal alkynes. Angew. Chem., Int. Ed. 2002, 41, 2596–2599. 10.1002/1521-3773(20020715)41:14<2596::AID-ANIE2596>3.0.CO;2-4. 12203546

[ref52] Hall M. P.; Unch J.; Binkowski B. F.; Valley M. P.; Butler B. L.; Wood M. G.; Otto P.; Zimmerman K.; Vidugiris G.; Machleidt T.; Robers M. B.; Benink H. A.; Eggers C. T.; Slater M. R.; Meisenheimer P. L.; Klaubert D. H.; Fan F.; Encell L. P.; Wood K. V. Engineered luciferase reporter from a deep sea shrimp utilizing a novel imidazopyrazinone substrate. ACS Chem. Biol. 2012, 7, 1848–1857. 10.1021/cb3002478. 22894855 PMC3501149

[ref53] Vaidehi N.; Schlyer S.; Trabanino R. J.; Floriano W. B.; Abrol R.; Sharma S.; Kochanny M.; Koovakat S.; Dunning L.; Liang M.; Fox J. M.; de Mendonca F. L.; Pease J. E.; Goddard W. A. 3rd; Horuk R. Predictions of CCR1 chemokine receptor structure and BX 471 antagonist binding followed by experimental validation. J. Biol. Chem. 2006, 281, 27613–27620. 10.1074/jbc.M601389200. 16837468

[ref54] Karlström S.; Nordvall G.; Sohn D.; Hettman A.; Turek D.; Ahlin K.; Kers A.; Claesson M.; Slivo C.; Lo-Alfredsson Y.; Petersson C.; Bessidskaia G.; Svensson P. H.; Rein T.; Jerning E.; Malmberg A.; Ahlgen C.; Ray C.; Vares L.; Ivanov V.; Johansson R. Substituted 7-amino-5-thio-thiazolo[4,5-d]pyrimidines as potent and selective antagonists of the fractalkine receptor (CX3CR1). J. Med. Chem. 2013, 56, 3177–3190. 10.1021/jm3012273. 23516963

[ref55] Lu M.; Zhao W.; Han S.; Lin X.; Xu T.; Tan Q.; Wang M.; Yi C.; Chu X.; Yang W.; Zhu Y.; Wu B.; Zhao Q. Activation of the human chemokine receptor CX3CR1 regulated by cholesterol. Sci. Adv. 2022, 8, eabn8048 10.1126/sciadv.abn8048. 35767622 PMC9242592

[ref56] Cederblad L.; Rosengren B.; Ryberg E.; Hermansson N. O. AZD8797 is an allosteric non-competitive modulator of the human CX3CR1 receptor. Biochem. J. 2016, 473, 641–649. 10.1042/BJ20150520. 26656484 PMC4764977

[ref57] Dwyer M. P.; Yu Y.; Chao J.; Aki C.; Chao J.; Biju P.; Girijavallabhan V.; Rindgen D.; Bond R.; Mayer-Ezel R.; Jakway J.; Hipkin R. W.; Fossetta J.; Gonsiorek W.; Bian H.; Fan X.; Terminelli C.; Fine J.; Lundell D.; Merritt J. R.; Rokosz L. L.; Kaiser B.; Li G.; Wang W.; Stauffer T.; Ozgur L.; Baldwin J.; Taveras A. G. Discovery of 2-hydroxy-N,N-dimethyl-3-{2-[[(R)-1-(5-methylfuran-2-yl)propyl]amino]-3,4-dioxocyclobut-1-enylamino}benzamide (SCH 527123): a potent, orally bioavailable CXCR2/CXCR1 receptor antagonist. J. Med. Chem. 2006, 49, 7603–7606. 10.1021/jm0609622. 17181143

[ref58] Yangthara B.; Mills A.; Chatsudthipong V.; Tradtrantip L.; Verkman A. S. Small-molecule vasopressin-2 receptor antagonist identified by a g-protein coupled receptor ″pathway″ screen. Mol. Pharmacol. 2007, 72, 86–94. 10.1124/mol.107.034496. 17435162

[ref59] Gilchrist A.; Gauntner T. D.; Fazzini A.; Alley K. M.; Pyen D. S.; Ahn J.; Ha S. J.; Willett A.; Sansom S. E.; Yarfi J. L.; Bachovchin K. A.; Mazzoni M. R.; Merritt J. R. Identifying bias in CCR1 antagonists using radiolabelled binding, receptor internalization, β-arrestin translocation and chemotaxis assays. Br. J. Pharmacol. 2014, 171, 5127–5138. 10.1111/bph.12835. 24990525 PMC4253460

[ref60] Nehme R.; Carpenter B.; Singhal A.; Strege A.; Edwards P. C.; White C. F.; Du H.; Grisshammer R.; Tate C. G. Mini-G proteins: Novel tools for studying GPCRs in their active conformation. PLoS One 2017, 12, e0175642 10.1371/journal.pone.0175642. 28426733 PMC5398546

[ref61] Bhangoo S.; Ren D.; Miller R. J.; Henry K. J.; Lineswala J.; Hamdouchi C.; Li B.; Monahan P. E.; Chan D. M.; Ripsch M. S.; White F. A. Delayed functional expression of neuronal chemokine receptors following focal nerve demyelination in the rat: a mechanism for the development of chronic sensitization of peripheral nociceptors. Mol. Pain 2007, 3, 38 10.1186/1744-8069-3-38. 18076762 PMC2228278

[ref62] El-Zohairy M. A.; Zlotos D. P.; Berger M. R.; Adwan H. H.; Mandour Y. M. Discovery of novel CCR5 ligands as anticolorectal cancer agents by sequential virtual screening. ACS Omega 2021, 6, 10921–10935. 10.1021/acsomega.1c00681. 34056245 PMC8153923

[ref63] Stefaniak J.; Huber K. V. M. Importance of quantifying drug-target engagement in cells. ACS Med. Chem. Lett. 2020, 11, 403–406. 10.1021/acsmedchemlett.9b00570. 32292539 PMC7153009

