# Supplementary material for: Fluorophore-Labeled Pyrrolones Targeting the Intracellular Allosteric Binding Site of the Chemokine Receptor CCR1
Source: ACS Pharmacol Transl Sci. 2024 Jun 21;7(7):2080–92. doi: 10.1021/acsptsci.4c00182 (PMC11249626; doi:10.1021/acsptsci.4c00182)
Supplement: Supplementary file 1 — pt4c00182_si_001.pdf [file pt4c00182_si_001.pdf]

Supporting Information

Fluorophore-labeled Pyrrolones Targeting the Intracellular Allosteric Binding Site of the Chemokine Receptor CCR1

Lara Toy,[Δ,‡] *Max E. Huber,*[Δ,‡] *Minhee Lee,*[§] *Ana Alonso Bartolomé,*[□,⌂] *Natalia V. Ortiz Zacarías,*[◊] *Sherif Nasser,*[₸] *Stephan Scholl,*[°] *Darius P. Zlotos,*[₸] *Yasmine M. Mandour,*[‖] *Laura H. Heitman,*[◊,ʃ] *Martyna Szpakowska,*[□] *Andy Chevigné,*[□] *Matthias Schiedel,*[§,Δ,*]

[Δ] Department of Chemistry and Pharmacy, Medicinal Chemistry, Friedrich-Alexander-University Erlangen-Nürnberg, Nikolaus-Fiebiger-Straße 10, 91058 Erlangen, Germany

[§] Institute of Medicinal and Pharmaceutical Chemistry, Technische Universität Braunschweig, Beethovenstraße 55, 38106 Braunschweig, Germany

[□] Immuno-Pharmacology and Interactomics, Department of Infection and Immunity, Luxembourg Institute of Health, rue Henri Koch 29, L-4354 Esch-sur-Alzette, Luxembourg

[⌂] Faculty of Science, Technology and Medicine, University of Luxembourg, 2 Avenue de l'Université, L-4365 Esch-sur-Alzette, Luxembourg

[◊] Leiden Academic Centre for Drug Research (LACDR), Division of Chemistry, Leiden University, 2333 CC Leiden, Netherlands

[₸] Department of Pharmaceutical Chemistry, Faculty of Pharmacy and Biotechnology, The German University in Cairo, 11835 New Cairo City, Cairo, Egypt

[°] Institute for Chemical and Thermal Process Engineering (ICTV), Technische Universität Braunschweig, Langer Kamp 7, 38106, Braunschweig, Germany

[‖] School of Life and Medical Sciences, University of Hertfordshire hosted by Global Academic Foundation, New Administrative Capital, 11578 Cairo, Egypt

[ʃ] Oncode Institute, Leiden University, 2333 CC Leiden, Netherlands

*Correspondence: Prof. Dr. Matthias Schiedel, Institute of Medicinal and Pharmaceutical Chemistry, Technische Universität Braunschweig, Beethovenstraße 55, 38106 Braunschweig, Germany, Email: matthias.schiedel@tu-braunschweig.de

Table of Contents

Page	Contents
**S3**	**Experimental Procedures**
**S3**	**Synthesis**
S3	General remarks
S4	Synthesis and compound characterization
**S20**	**Biological tests**
S20	Cell culture
S20	Transient transfection using polyethylenimine
S20	Membrane preparation
S20	cDNA constructs
S20	ELISA
S21	Emission and excitation spectra of the fluorescent ligands
S21	Emission spectra of Nluc-labeled CCR1 (CCR1_Nluc) protein
S21	NanoBRET binding assays
S22	Kinetic solubility assay
S23	Membrane-based radioligand binding assays
S23	Cellular NanoBiT β-arrestin recruitment assays
S23	Cellular NanoBiT miniGi recruitment assays
**S23**	**Computational Methods**
S23	Virtual screening
S23	Molecular docking
S24	Molecular dynamics (MD) simulation
**S25**	**Supplementary Figures**
**S41**	**Supplementary Schemes**
**S44**	**Supplementary Tables**
**S45**	**Supplementary NMR Spectra**
**S119**	**Supplementary HPLC Chromatograms**
**S145**	**Supplementary References**

Experimental Procedures

Synthesis

General remarks: Starting materials (chemicals) were purchased from commercial suppliers (Abcr, Acros, Alfa Aesar, BLDpharm, Sigma Aldrich, TCI) and used without any further purification. Solvents were used in p.a. quality and dried according to common procedures, if necessary. Literature known compounds were either purchased from commercial suppliers (MedChemExpress (BX-471 (**1**), AZD8797 (**18**), navarixin (**20**)), kindly provided by the opnMe program of Boehringer Ingelheim (BI 639667 (**3**)), or synthesized according to previously published procedures (cmpd39 (**5**),[1] **8**,[1] **9**,[1] **13**,[1] **14**,[1] 6-carboxy-tetramethylrhodamine (**17**),[2] cmpd27 (**19**),[1] vercirnon (**21**),[3] AAA30 (**22**).[3] The 24 hit compounds from the virtual screening campaign (SN_1 - SN_24) were ordered from Specs (Zoetermeer, Netherlands). Thin-layer chromatography (TLC) for reaction monitoring was performed with alumina plates coated with Merck silica gel 60 F_{254} (layer thickness: 0.2 mm) or Merck silica gel 60 RP-18 F_{254} (layer thickness: 0.2 mm) and analyzed under UV-light (254 nm). As an alternative method for reaction monitoring, we used high performance liquid chromatography mass spectrometry (HPLC-MS). HPLC-MS analyses were performed using a Thermo Scientific Dionex UltiMate 3000 HPLC system in combination with a DAD detector (220/230/254 nm) and an Agilent ZORBAX ECLIPSE, XDB-C8 column (3.0 mm x 100 mm, 3.5 µm). Elution was performed at room temperature under gradient conditions. Eluent A was water containing 0.1% (v/v) formic acid; eluent B was methanol. Linear gradient conditions were as follows: 0‑0.2 min: A=75%, B=25%; 0.2‑6.0 min: linear increase to B=100%; 6.0‑8.5 min: B=100%; 8.5‑9.0 min: linear decrease to A=75%, B=25%; 9.0-12.0 min: A=75%, B=25%. A flow rate of 0.4 mL·min^{-1} was maintained during the entire elution. Mass detection was performed with a BRUKER amaZon SL mass spectrometer using ESI as ionization source. As an alternative method for low resolution electrospray ionization mass spectrometry (LRMS-ESI), we used an Advion expression® compact mass spectrometer (CMS) coupled with an automated TLC plate reader Plate Express® (Advion) and an atmospheric pressure chemical ionization (APCI) device. High-resolution mass spectrometry (HRMS) was performed on an AB Sciex Triple TOF660 Sciex, on a Bruker maXis MS, a Bruke rtimsTOF Pro, or a LTQ-Orbitrap Velos spectrometer using an electrospray ionization (ESI) or atmospheric pressure photoionization (APPI) source. Flash column chromatography was performed with hand packed Silica Columns 60M (0.040-0.063 mm, 230-400 mesh) as a stationary phase on a Biotage Selekt automated flash purification system with UV-Vis detector. Optical rotation was measured with a JASCO C-2000 polarimeter (cylindrical glass cuvette with a path of 100 mm and a volume of 1 mL). Yields were not optimized. NMR spectra were recorded using either a Bruker Avance 400 (^{1}H: 400 MHz, ^{13}C: 101 MHz), Bruker Avance 500 (^{1}H: 500 MHz, ^{13}C: 126 MHz), or a Bruker Avance 600 (^{1}H: 600 MHz, ^{13}C: 151 MHz) instrument. The spectra are referenced against the NMR solvent or tetramethylsilane (TMS) and are reported as follows: ^{1}H: chemical shift δ (ppm), multiplicity (s = singlet, d = doublet, dd = doublet of doublets, t = triplet, m = multiplet, b = broad), integration, coupling constant (*J* in Hz). ^{13}C: chemical shift δ (ppm), abbreviations: carbons that could not be found in ^{13}C spectra (DEPTQ) but in HMBC or HSQC are additionally marked with an asterisk (*). Signals that are partially overlaid by a solvent signal are marked with a hashtag (#). The assignment resulted from HMBC and HSQC experiments. High resolution mass spectra were measured with a timsTOF Pro Mass Spectrometer from Bruker Daltonics using ESI as ionization source. Purity was determined for all tested compounds by HPLC and UV detection and was >95%. HPLC analyses were performed using an Agilent 1200 series HPLC system employing a diode array detector (DAD, detection at 200, 220, 254 or 560 nm) and a ZORBAX ECLIPSE, XDB-C8 column (4.6 mm x 150 mm, 5 µm) with a flow rate of 0.5 mL·min^{-1}. If not stated otherwise, the indicated purity was determined at a wavelength of 254 nm. As solvent systems the following binary solvent systems were used. M1: Elution was performed at room temperature under gradient conditions. Eluent A was water containing 0.1% (v/v) TFA; eluent B was acetonitrile. Linear gradient conditions were as follows: 0‑3.0 min: A=90%, B=10%; 3.0-18.0 min: linear increase to A=5%, B=95%; 18.0-24.0 min: A=5%, B=95%; 24.0‑27.0 min: linear decrease to A=90%, B=10%; 27.0-30.0 min: A=90%, B=10%. M2: Elution was performed at room temperature under gradient conditions. Eluent A was water containing 0.1% (v/v) formic acid; eluent B was methanol. Linear gradient conditions were as follows: 0‑3.0 min: A=90%, B=10%; 3.0-18.0 min: linear increase to B=100%; 18.0-24.0 min: B=100%; 24.0-27.0 min: linear decrease to A=90%, B=10%; 27.0-30.0 min: A=90%, B=10%. M3: Elution was performed at room temperature under gradient conditions. Eluent A was water containing 0.1% (v/v) formic acid; eluent B was methanol. Linear gradient conditions were as follows: 0‑3.0 min: A=90%, B=10%; 3.0-48.0 min: linear increase to B=100%; 48.0-54.0 min: B=100%; 54.0-57.0 min: linear decrease to A=90%, B=10%; 57.0-60.0 min: A=90%, B=10%.

Synthesis and compound characterization:

1-(4-Bromo-2-fluorophenyl)-5-cyclohexyl-3-hydroxy-4-(4-(4-(methoxymethyl)-1*H*-1,2,3-triazol-1-yl)butanoyl)-1,5-dihydro-2*H*-pyrrol-2-one (**10**)

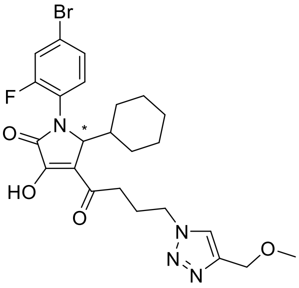

4-(4-Azidobutanoyl)-1-(4-bromo-2-fluorophenyl)-5-cyclohexyl-3-hydroxy-1,5-dihydro-2*H*-pyrrol-2-one (**14**, 10.0 mg, 21.5 µmol, 1.0 eq), 3-methoxyprop-1-yne (1.81 mg, 2.20 µL, 25.8 µmol, 1.2 eq), and tris(benzyltriazolylmethyl)amine (TBTA, 1.14 mg, 2.15 µmol, 0.1 eq) were dissolved in a water/*tert*-BuOH/DMF mixture (1.8 mL, 1:1:1). An aqueous $CuSO_4$ solution (21.8 µL, 0.1 M, 0.1 eq) and an aqueous solution of sodium ascorbate (42.9 µL, 0.1 M, 0.2 eq) were added in that order. The resulting reaction mixture was stirred for 1 h at room temperature under nitrogen atmosphere. After completion, volatiles were removed under reduced pressure. The residue was purified by preparative HPLC (acetonitrile/water (0.1% TFA): gradient 30-58%) to obtain the TFA salt of the title compound as a colourless solid (8.0 mg, 57%). ^{1}H NMR (600 MHz, DMSO-d_6 δ [ppm]): 12.37 (bs, 1H, pyrrolone -OH), 8.11 (s, 1H, triazole H-5), 7.75 (dd, *J* = 10.2, 1.9 Hz, 1H, phenyl H-6), 7.60 – 7.48 (m, 2H, phenyl H-3,5), 4.91 (d, *J* = 1.9 Hz, 1H, pyrrolone H-5), 4.44 (s, 2H, -triazole-C<u>H</u>$_2$-O-CH_3), 4.39 (t, *J* = 7.1 Hz, 2H,-CO-CH_2-CH_2-C<u>H</u>$_2$-triazole-), 3.25 (s, 3H, -CH_3), 2.90 – 2.76 (m, 2H, -CO-C<u>H</u>$_2$-CH_2-CH_2-triazole-), 2.16 – 2.05 (m, 2H, -CO-CH_2-C<u>H</u>$_2$-CH_2-triazole-), 1.95 – 1.85 (m, 1H, cyclohexyl H-1), 1.64 – 1.30 (m, 5H, cyclohexyl -CH_2-), 1.06 – 0.73 (m, 4H, cyclohexyl -CH_2-), 0.54 – 0.41 (m, 1H, cyclohexyl -CH_2-);^{13}C NMR (DEPTQ, 151 MHz, DMSO-d_6 δ [ppm]): 194.44* q (C=O), 164.55 q (pyrrolone C-3), 157.66 q (q, *J* = 30.2 Hz, $^-$OO<u>C</u>-CF_3), 156.66 q (d, *J* = 254.6 Hz, phenyl C-2), 152.62* q (pyrrolone C-2), 143.81 q (triazole C-4), 129.82 (phenyl C-5), 128.18 (d, *J* = 3.0 Hz, phenyl C-6), 124.98 q (d, *J* = 10.9 Hz, phenyl C-1), 123.74 (triazole C-5), 120.42 (d, *J* = 9.3 Hz, phenyl C-4), 120.07 (d, *J* = 23.5 Hz, phenyl C-3), 118.85* q (pyrrolone C-4), 64.97 (-triazole-<u>C</u>H_2-O-CH_3), 62.59 (pyrrolone C-5), 57.23 (-CH_3), 48.72 (-CO-CH_2-CH_2-<u>C</u>H_2-triazole-), 39.66$^{\#*}$ (cyclohexyl C-1), 38.81 (-CO-<u>C</u>H_2-CH_2-CH_2-triazole-), 30.28, 26.45, 25.95, 25.84, 25.55 (cyclohexyl C-2,3,4,5,6), 24.20 (-CO-CH_2-<u>C</u>H_2-CH_2-triazole-); LRMS (ESI$^+$): *m/z* 535 $[M+H]^+$; HRMS (ESI$^+$): *m/z* calcd for $C_{24}H_{29}BrFN_4O_4^+$: 535.1351 $[M+H]^+$, found: 535.1351 $[M+H]^+$; HPLC retention time: 18.33 min, 99% (M1).

4-((2-(2-((1-(4-(1-(4-Chloro-2-fluorophenyl)-2-cyclohexyl-4-hydroxy-5-oxo-2,5-dihydro-1*H*-pyrrol-3-yl)-4-oxobutyl)-1*H*-1,2,3-triazol-4-yl)methoxy)ethoxy)ethyl)carbamoyl)-2-(6-(dimethylamino)-3-(dimethyliminio)-3*H*-xanthen-9-yl)benzoate (**11**)

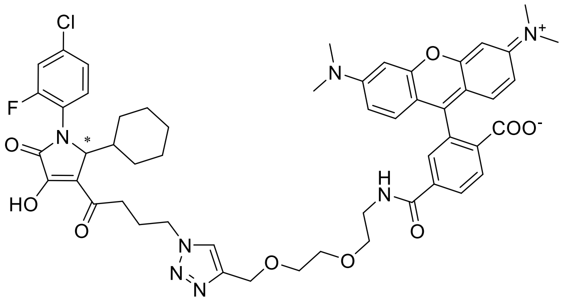

4-Acetyl-1-(4-chloro-2-fluorophenyl)-5-cyclohexyl-3-hydroxy-1,5-dihydro-2*H*-pyrrol-2-one (**13**, 19.0 mg, 45.1 µmol, 1.0 eq), 2-(6-(dimethylamino)-3-(dimethyliminio)-3*H*-xanthen-9-yl)-4-((2-(2-(prop-2-yn-1-yloxy)ethoxy)ethyl)carbamoyl)benzoate (**15**, 25.1 mg, 45.1 µmol, 1.0 eq), and tris(benzyltriazolylmethyl)amine (TBTA, 2.40 mg, 4.51 µmol, 0.1 eq) were dissolved in a water/*tert*-BuOH/DMF mixture (1.5 mL, 1:1:1). An aqueous $CuSO_4$ solution (45.1 µL, 0.1 M, 0.1 eq) and an aqueous solution of sodium ascorbate (90.2 µL, 0.1 M, 0.2 eq) were added in that order. The resulting reaction mixture was stirred for 1 h at room temperature under nitrogen atmosphere. After completion, volatiles were removed under reduced pressure. The residue was purified by preparative HPLC (acetonitrile/water (0.1% TFA): gradient 30-52%) to obtain the TFA salt of title compound as a colorless solid (12 mg, 25%). ^{1}H NMR (600 MHz, DMSO-d_6 δ [ppm]): 13.34 (bs, 1H, -COOH), 12.34 (bs, 1H, pyrrolone -OH), 8.82 (t, *J* = 5.6 Hz, 1H, -N<u>H</u>CO-), 8.32 – 8.19 (m, 2H, carbamoyl benzoate H-5,6), 8.06 (s, 1H triazole H-5), 7.86 (s, 1H, carbamoyl benzoate H-3), 7.66 – 7.59 (m, 2H, phenyl H-3,6), 7.39 (dd, *J* = 8.7, 2.3 Hz, 1H, phenyl H-5), 7.10 – 6.81 (m, 6H, 3*H*-xanthene H-1,2,4,5,7,8), 4.91 (d, *J* = 2.0

Hz, 1, pyrrolone H-5), 4.48 (s, 2H, -triazole-C$\underline{H}_2$-O-), 4.37 (t, *J* = 7.1 Hz, 2H, -CH$_2$-C$\underline{H}_2$-triazole-), 3.55 – 3.51 (m, 6H, -triazole-CH$_2$-O-C$\underline{H}_2$-C$\underline{H}_2$-O-C$\underline{H}_2$-), 3.43 – 3.40 $^{\#}$ (m, 2H, -C$\underline{H}_2$-NHCO-), 3.27 – 3.18 (m, 12H, -N(CH$_3$)$_2$ & =N$^+$(CH$_3$)$_2$), 2.90 – 2.78 (m, 2H,-CO-C$\underline{H}_2$-CH$_2$-), 2.13 – 2.04 (m, *J* = 7.0, 2.5 Hz, 2H, -CO-CH$_2$-C$\underline{H}_2$-), 1.94 – 1.85 (m, 1H, cyclohexyl H-1), 1.63 – 1.30 (m, 5H, cyclohexyl -CH$_2$-), 1.04 – 0.74 (m, 4H, cyclohexyl -CH$_2$-), 0.52 – 0.43 (m, 1H, cyclohexyl -CH$_2$-); ^{13}C NMR (DEPTQ, 151 MHz, DMSO-*d$_6$* δ [ppm]): 194.54 q (-$\underline{C}$O-CH$_2$-), 164.54 q (-NH$\underline{C}$O-), 164.38 q (pyrrolone C-3), 157.65 q (q, *J* = 30.7 Hz, F$_3$C-$\underline{C}$OO$^-$), 156.60 q (d, *J* = 253.6 Hz, phenyl C-2), 152.40 q (pyrrolone C-2), 143.89 q (triazole C-4), 132.58 q (d, *J* = 9.9 Hz, phenyl C-4), 130.48 (*3H*-xanthene C-1,8), 129.48 (phenyl C-6), 128.98 (carbamoyl benzoate C-5), 125.25 (d, *J* = 3.4 Hz, phenyl C-5), 124.47 q (d, *J* = 12.0 Hz, phenyl C-1), 123.67 (triazole C-5), 119.19 q (pyrrolone C-4), 117.30 (d, *J* = 23.9 Hz, phenyl C-3), 96.38 (3*H*-xanthene C-4,5), 69.47 (-O-$\underline{C}$H$_2$-CH$_2$-NHCO-), 68.80, 68.71 (-O-$\underline{C}$H$_2$-$\underline{C}$H$_2$-O-), 63.48 (-triazole-$\underline{C}$H$_2$-O-), 62.62 (pyrrolone C-5), 48.67 (-CO-CH$_2$-CH$_2$-$\underline{C}$H$_2$-triazole-), 40.42 (-N(CH$_3$)$_2$ & =N$^+$(CH$_3$)$_2$, 39.75$^{\#}$ (cyclohexyl C-1), 39.72$^{\#}$ (-O-CH$_2$-$\underline{C}$H$_2$-NHCO-), 38.88 (-CO-$\underline{C}$H$_2$-CH$_2$-CH$_2$-triazole-), 30.22 (cyclohexyl -CH$_2$-), 26.43 (cyclohexyl -CH$_2$-), 25.91 (cyclohexyl -CH$_2$-), 25.81 (cyclohexyl -CH$_2$-), 25.53 (cyclohexyl -CH$_2$-), 24.13 (-CO-CH$_2$-$\underline{C}$H$_2$-CH$_2$-triazole-); UV-Vis: $\lambda_{max(Ex)}$ = 554 nm, $\lambda_{max(Em)}$ = 612 nm; LRMS (ESI$^+$) *m/z*: 976 [M+H]$^+$; HRMS(ESI$^+$): *m/z* calcd for $C_{52}H_{56}ClFN_7O_9^+$: 976.3807 [M+H]$^+$, found: 976.3810; HPLC retention time: 17.65 min, > 99% (M1).

a) the ^{13}C NMR signals for $^-$OOC-$\underline{C}$F$_3$, -COOH, carbamoyl benzoate C-1,2,3,4,6, 3*H*-xanthene C-2,3,4a,6,7,8a,9,9a,10a could not be detected.

4-((2-(2-((1-(4-(1-(4-Bromo-2-fluorophenyl)-2-cyclohexyl-4-hydroxy-5-oxo-2,5-dihydro-1*H*-pyrrol-3-yl)-4-oxobutyl)-1*H*-1,2,3-triazol-4-yl)methoxy)ethoxy)ethyl)carbamoyl)-2-(6-(dimethylamino)-3-(dimethyliminio)-3*H*-xanthen-9-yl)benzoate (**12**, LT166):

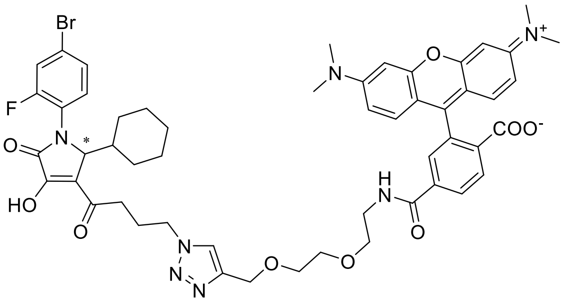

4-(4-Azidobutanoyl)-1-(4-bromo-2-fluorophenyl)-5-cyclohexyl-3-hydroxy-1,5-dihydro-2*H*-pyrrol-2-one (**14**, 15.4 mg, 32.2 µmol, 1.0 eq), 2-(6-(dimethylamino)-3-(dimethyliminio)-3H-xanthen-9-yl)-4-((2-(2-(prop-2-yn-1-yloxy)ethoxy)ethyl)carbamoyl)benzoate (**15**, 17.9 mg, 32.2 µmol , 1.0 eq), and tris(benzyltriazolylmethyl)amine (TBTA, 1.71 mg, 3.22 µmol, 0.1 eq) were dissolved in a water/*tert*-BuOH/DMF mixture (1.8 mL, 1:1:1). An aqueous $CuSO_4$ solution (32.2 µL, 0.1 M, 0.1 eq) and an aqueous solution of sodium ascorbate (64.4 µL, 0.1 M, 0.2 eq) were added in that order. The resulting reaction mixture was stirred for 1 h at room temperature under nitrogen atmosphere. After completion, volatiles were removed under reduced pressure. The residue was purified by preparative HPLC (acetonitrile/water (0.1% TFA): gradient 30-62%) to obtain the TFA salt of title compound as a pink solid (9 mg, 24%). ^{1}H NMR (600 MHz, DMSO-*d$_6$* δ [ppm]): 13.28 (bs, 1H, Ar-COOH), 12.39 (bs, 1H, pyrrolone C-2), 8.81 (t, *J* = 5.6 Hz, 1H, -N$\underline{H}$CO-), 8.28 – 8.19 (m, 2H, carbamoyl benzoate H-5,6), 8.06 (s, 1H, triazole H-5), 7.84 (s, 1H, carbamoyl benzoate H-3), 7.76 (d, *J* = 10.2, 2.0 Hz, 1H, phenyl H-5), 7.58 – 7.49 (m, 2H, phenyl H-3,6), 7.01 – 6.81 (m, 6H, *3H*-xanthene H-1,2,4,5,7,8), 4.91 (s, 1H, pyrrolone H-5), 4.47 (s, 2H, -triazole-C$\underline{H}_2$-O-), 4.37 (t, *J* = 6.8 Hz, 2H, -CH$_2$-C$\underline{H}_2$-triazole-), 3.54 – 3.50$^{\#}$ (m, 6H, -triazole-CH$_2$-O-C$\underline{H}_2$-C$\underline{H}_2$-O-C$\underline{H}_2$-), 3.42 – 3.39$^{\#}$ (m, 2H, -C$\underline{H}_2$-NHCO-), 3.23 – 3.12 (m, 12H, -N(CH$_3$)$_2$ & =N$^+$(CH$_3$)$_2$), 2.90 – 2.78 (m, 2H,-CO-C$\underline{H}_2$-CH$_2$-), 2.13 – 2.04 (m, 2H, -CO-CH$_2$-C$\underline{H}_2$-), 1.93 – 1.85 (m, 1H, cyclohexyl H-1), 1.62 – 1.31 (m, 5H, cyclohexyl -CH$_2$-), 1.05 – 0.74 (m, 4H, cyclohexyl -CH$_2$-), 0.52 – 0.42 (m, 1H, cyclohexyl -CH$_2$-); ^{13}C NMR (DEPTQ, 151 MHz, DMSO-d$_6$ δ [ppm]): 194.53 q (-$\underline{C}$O-CH$_2$-), 164.55 q (-NH$\underline{C}$O-), 164.38 q (pyrrolone C-2), 157.31 q (q, *J* = 36.2 Hz, F$_3$C-$\underline{C}$OO$^-$), 156.62 q (d, *J* = 254.9 Hz, phenyl C-2), 152.54 q (pyrrolone C-2), 143.90 q (triazole C-4), 130.25 (*3H*-xanthene C-1,8), 129.78 (phenyl C-5), 129.01 (carbamoyl benzoate C-5), 128.17 (d, *J* = 2.5 Hz, phenyl C-6), 124.88 q (d, *J* = 12.6 Hz, phenyl C-1), 123.67 (triazole C-5), 120.45 q (d, *J* = 9.7 Hz, phenyl C-4), 120.06 (d, *J* = 23.4 Hz, phenyl C-3), 119.11 q (pyrrolone C-4), 96.61 (3*H*-xanthene C-4,5), 69.45 (-O-$\underline{C}$H$_2$-CH$_2$-NHCO-), 68.79, 68.70 (-O-$\underline{C}$H$_2$-$\underline{C}$H$_2$-O-), 63.47 (-triazole-$\underline{C}$H$_2$-O-), 62.58 (pyrrolone C-5), 48.67 (-CO-CH$_2$-CH$_2$-$\underline{C}$H$_2$-triazole-), 40.32 (-N(CH$_3$)$_2$ & =N$^+$(CH$_3$)$_2$, 39.75$^{\#}$ (cyclohexyl C-1), 39.47$^{*\#}$ (-O-CH$_2$-$\underline{C}$H$_2$-NHCO-), 38.86 (-CO-$\underline{C}$H$_2$-CH$_2$-CH$_2$-triazole-), 30.22 (cyclohexyl -CH$_2$-), 26.41 (cyclohexyl -CH$_2$-), 25.91 (cyclohexyl -CH$_2$-), 25.80 (cyclohexyl -CH$_2$-), 25.52 (cyclohexyl -CH$_2$-), 24.14 (-CO-CH$_2$-$\underline{C}$H$_2$-CH$_2$-triazole-); UV-Vis: $\lambda_{max(Ex)}$ = 558 nm, $\lambda_{max(Em)}$ = 612 nm; LRMS (ESI+) *m/z*: 1020 [M+H]$^+$; HRMS(ESI+): *m/z* calcd for $C_{52}H_{56}BrFN_7O_9^+$: 1020.3301 [M+H]$^+$,found: 1020.3300 [M+H]$^+$; HPLC retention time: 15.30 min, 97% (M1).

a) the ^{13}C NMR signals for $^-$OOC-$\underline{C}$F$_3$, -COOH, carbamoyl benzoate C-1,2,3,4,6, *3H*-xanthene C-2,3,4a,6,7,8a,9,9a,10a could not be detected.

2-(6-(Dimethylamino)-3-(dimethyliminio)-3*H*-xanthen-9-yl)-4-((2-(2-(prop-2-yn-1-yloxy)ethoxy)ethyl)carbamoyl)benzoate (**15**, 6-TAMRA-PEG$_2$-alkine)

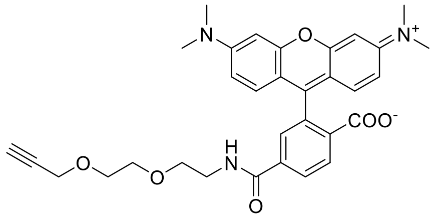

6-Carboxy-tetramethylrhodamine (**17**, 120 mg, 279 µmol, 1.0 eq) was dissolved in DMF (1 mL) and cooled to 0°C. TBTU (134 mg, 418 µmol, 1.5 eq) was dissolved in DMF (0.5 mL), DIPEA was added (54 mg, 418 µmol, 1.5 eq) and the mixture was added dropwise. The reaction mixture was stirred for 15 min at 0°C before 2-[2-(2-propynyloxy)ethoxy]ethylamine (H_2N-PEG$_2$-alkyne (**16**), 43.9 mg, 307 µmol, 1.1 eq) was added. The reaction mixture was stirred for further 2 h at ambient temperature. After evaporation of the volatile components, the crude product was purified by automated flash chromatography (methanol (+ 0.1% NEt_3)/CH_2Cl_2: gradient 1 - 10%) to obtain the title compound (98 mg, 63%) as a pink solid; ^{1}H NMR (400 MHz, DMSO-d_6 δ [ppm]): 8.78 (t, *J* = 5.6 Hz, 1H, -NHCO-), 8.16 (dd, *J* = 8.0, 1.4 Hz, 1H, carbamoylbenzoate H-5), 8.06 (d, *J* = 8.0, 1H, carbamoylbenzoate H-6), 7.68 – 7.59 (m, 1H, carbamoylbenzoate H-3), 6.58 – 6.43 (m, 6H, *3H*-xanthene H-1,2,4,5,7,8), 4.05 (d, *J* = 2.4 Hz, 2H, HC≡C-CH_2-O-), 3.51 – 3.48 (m, 4H, -O-CH_2-CH_2-O-), 3.46 (t, *J* = 5.9, 2H, -O-CH_2-CH_2-NHCO-), 3.38 (t, *J* = 2.4 Hz, 1H, -O-CH_2-CH_2-NHCO-), 3.37 – 3.32 (m, 2H, -O-CH_2-CH_2-NHCO-), 2.94 (s, 12H, -N(CH_3)$_2$ & =N$^+$(CH_3)$_2$); ^{13}C NMR (DEPTQ, 151 MHz, DMSO-d_6 δ [ppm]): 168.23 q (-COO$^-$), 164.63 q (-NHCO-), 152.85 q (carbamoyl benzoate C-4), 152.13 q (3*H*-xanthene C-4a,10a), 151.94 q (3*H*-xanthene C-3,6), 140.38 q (carbamoylbenzoate C-2), 129.20 (carbamoylbenzoate C-5), 128.55 q (carbamoylbenzoate C-1), 128.47 (3*H*-xanthene C-1,8), 124.66 (carbamoylbenzoate C-6), 122.25 (carbamoylbenzoate C-3), 109.06 (3*H*-xanthene C-2,7), 105.62 q (3*H*-xanthene C-8a,9a), 97.95 (3*H*-xanthene C-4,5), 84.71 q (-C≡CH), 77.03 (-C≡CH), 69.18, 68.59 (-O-CH_2-CH_2-O-), 68.36 (-O-CH_2-CH_2-NHCO-), 57.40 (-O-CH_2-C≡CH), 39.78$^\#$ (-N(CH_3)$_2$ & =N$^+$(CH_3)$_2$), 39.24$^\#$ (-O-CH_2-CH_2-NHCO-)**;** LRMS (ESI$^+$) *m/z* (%): 556 [M+H]$^+$. The attached ^{1}H and ^{13}C NMR spectra contain signals of residual DIPEA.

4-Benzoyl-5-(4-fluorophenyl)-3-hydroxy-1-(4-hydroxyphenethyl)-1,5-dihydro-2*H*-pyrrol-2-one (**23**, V$_2$Ri$_{nh}$-02)[4]

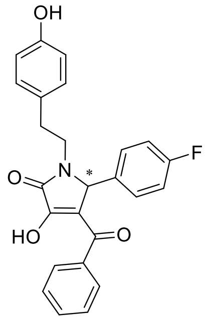

4-Fluorobenzaldehyde (33.6 mg, 271 µmol, 1.0 eq), ethyl 2,4-dioxo-4-phenylbutanoate (**44**, 50.0 mg, 271 µmol, 1.0 eq), and 4-(2-aminoethyl)phenol (37.2 mg, 271 µmol, 1.0 eq) were placed in a microwave tube and dissolved in acetic acid (1.0 mL). The reaction mixture was heated to 90 °C and stirred for 18 h. After complete conversion of the starting materials monitored by LC-MS, the colorless precipitate was separated by filtration and washed with Et_2O, then purified by preparative HPLC (acetonitrile/water (0.1% TFA): gradient 30-65%) to obtain the title compound as a colorless solid (44 mg, 38%). ^{1}H NMR (400 MHz, DMSO-d_6 δ [ppm]): 11.80 (bs, 1H, pyrrolone -OH), 9.24 (s, 1H, 4-hydroxyphenethyl -OH), 7.70 – 7.63 (m, 2H, benzoyl H-2,6), 7.58 – 7.50 (m, 1H, benzoyl H-4), 7.46 – 7.40 (m, 2H, benzoyl H-3,5), 7.37 – 7.29 (m, 2H, 4-fluorophenyl H-2,6), 7.18 – 7.10 (m, 2H, 4-fluorophenyl H-3,5), 6.94 – 6.88 (m, 2H, 4-hydroxyphenethyl H-2,6), 6.69 – 6.62 (m, 2H, 4-hydroxyphenethyl H-3,5), 5.32 (s, 1H, pyrrolone H-5), 3.83 – 3.62 (m, 1H, one H of Ar-CH_2-CH_2-), 2.80 – 2.63 (m, 2H, one H of Ar-CH_2-CH_2- and one H of Ar-CH_2-CH_2-), 2.60 – 2.53$^\#$ (m, 1H, one H of Ar-CH_2-CH_2-); ^{13}C NMR (DEPTQ, 101 MHz, DMSO-d_6, δ [ppm]): 189.04 q (benzoyl C=O), 164.79 q (pyrrolone C-2), 161.89 q (d, *J* = 244.3 Hz, 4-fluorophenyl C-4), 155.86 q (4-hydroxyphenethyl C-4), 151.44 q (pyrrolone C-3), 138.02 q (benzoyl C-1), 132.54 (benzoyl C-4), 132.33 q (d, *J* = 2.8 Hz, 4-fluorophenyl C-1), 129.88 (d, *J* = 8.4 Hz, 4-fluorophenyl C-2,6), 129.49 (4-hydroxyphenethyl C-2,6), 128.70 (benzoyl C-2,6), 128.55 q (4-hydroxyphenethyl C-1), 128.14 (benzoyl C-3,5), 119.13 q (pyrrolone C-4), 115.50 (d, *J* = 21.5 Hz, 4-fluorophenyl C-3,5), 115.25 (4-hydroxyphenethyl C-3,5), 60.17 (pyrrolone C-5), 41.92 (-CH_2-CH_2-Ar), 32.80 (-CH_2-CH_2-Ar); LRMS (ESI$^+$) *m/z*: 418 [M+H]$^+$, HRMS (ESI$^+$): *m/z* calcd for $C_{25}H_{21}FNO_4^+$:

418.1449 [M+H]+, found: 418.1447. HPLC retention time 19.32 min, > 99% (M2). The obtained analytical data are in good agreement with literature values.[4]

The enantiomer (+)-**23** was obtained in a two-step procedure from the diastereomer **50a** (see Scheme S3). In brief, **50a** (65 mg, 112 µM,1.0 eq) and lithium chloride (28.5 mg, 673 µM, 6.0 eq) were diluted in a microwave tube with 1,3-dimethyl-2-imidazolidinone (1.5 mL) and heated at 80 °C for 24 h. After cooling down to room temperature, the reaction mixture was quenched with water and acidified with 1N HCl. The mixture was extracted with toluene (3 x 10 mL). The combined organic layer was washed with saturated brine (30 mL), dried over magnesium sulfate, and concentrated under reduced pressure. The obtained residue was purified by automated column chromatography (EtOH/DCM, gradient 2-100%) to obtain the *O*-methyl analogue of (+)-**23** as colorless solid (25.0 mg, 51%). R_f = 0.35 (MeOH/DCM, 1:9); LRMS (ESI+) *m/z*: 432 [M+H]+. *O*-Demethylation was achieved in a second step by suspending the *O*-methyl analogue of (+)-**23** (25.0 mg, 58.0 µM, 1.0 eq) in dry CH_2Cl_2 (0.5 mL) and cooling down to 0°C. Then, BBr_3 (1M in CH_2Cl_2, 10 eq) was added dropwise. After the addition of BBr_3, the reaction mixture was stirred at room temperature overnight. Then, the reaction mixture was quenched with water and neutralized with $NaHCO_3$ while cooling on an ice-bath. The organic and aqueous layers were separated, and the aqueous layer was extracted with EtOAc (3 x 10 mL). The combined organic layer was dried over $MgSO_4$, filtered, and concentrated under reduced pressure. The crude residue was purified by trituration with Et_2O to obtain the title compound as a colorless solid (4 mg, 17%). LRMS (ESI+) *m/z*: 418 [M+H]+; HPLC retention time: 19.19 min, 99% (M2); $[\alpha]_D^{24}$: + 2.15° (c = 0.11, DMSO-d_6).

The enantiomer (-)-**23** was obtained in a two-step procedure from the diastereomer **50b** (see Scheme S3). In brief, **50b** (40 mg, 68.9 µM,1.0 eq) and lithium chloride (17.5 mg, 414 µM, 6.0 eq) were diluted in a microwave tube with 1,3-dimethyl-2-imidazolidinone (1.5 mL) and heated at 80 °C for 24 h. After cooling down to room temperature, the reaction mixture was quenched with water and acidified with 1N HCl. The mixture was extracted with toluene (3 x 10 mL). The combined organic layer was washed with saturated brine (30 mL), dried over magnesium sulfate, and concentrated under reduced pressure. The obtained residue was purified by automated column chromatography (EtOH/DCM, gradient 2-100%) to obtain the *O*-methyl analogue of (-)-**23** as pale yellow solid (17.0 mg, 57%). R_f = 0.35 (MeOH/DCM, 1:9); LRMS (ESI+) *m/z*: 432 [M+H]+. *O*-Demethylation was achieved in a second step by suspending the *O*-methyl analogue of (-)-**23** (17.0 mg, 39.4 µM, 1.0 eq) in dry CH_2Cl_2 (0.5 mL) and cooling down to 0°C. Then, BBr_3 (1M in CH_2Cl_2, 10 eq) was added dropwise. After the addition of BBr_3, the reaction mixture was stirred at room temperature overnight. Then, the reaction mixture was quenched with water and neutralized with $NaHCO_3$ while cooling on an ice-bath. The organic and aqueous layers were separated, and the aqueous layer was extracted with EtOAc (3 x 10 mL). The combined organic layer was dried over $MgSO_4$, filtered, and concentrated under reduced pressure. The crude residue was purified by trituration with Et_2O to obtain the title compound as a colorless solid (6 mg, 37%). LRMS (ESI+) *m/z*: 418 [M+H]+; HPLC retention time: 19.24 min, 97% (M2); $[\alpha]_D^{24}$: - 2.23° (c = 0.12, DMSO-d_6).

4-Acetyl-5-cyclohexyl-3-hydroxy-1-(4-hydroxyphenethyl)-1,5-dihydro-2*H*-pyrrol-2-one (**24**)

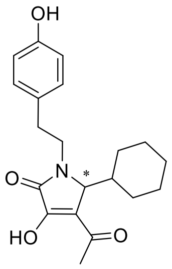

Cyclohexane carbaldehyde (21.2 mg, 189 µmol, 1.0 eq), ethyl 2,4-dioxopentanoate (50.0 mg, 189 µmol, 1.0 eq), and 4-(2-aminoethyl)phenol (25.9 mg, 189 µmol, 1.0 eq) were placed in a microwave tube and dissolved in acetic acid (1.0 mL). The reaction mixture was heated to 80 °C and stirred for 16 h. After complete conversion of the starting materials monitored by LC-MS, the acetic acid was removed under reduced pressure and the residue was purified by preparative HPLC (acetonitrile/water (0.1% TFA): gradient 30-56%) to obtain the colorless solid (14 mg, 40.8 µmol, 21%). ^{1}H NMR (400 MHz, DMSO-d_6 δ [ppm]): 11.90 (bs, 1H, pyrrolone -OH), 9.21 (s, 1H, 4-hydroxyphenethyl -OH), 7.00 – 6.88 (m, 2H, 4-hydroxyphenethyl H-2,6), 6.75 – 6.54 (m, 2H, 4-hydroxyphenethyl H-3,5), 4.07 (s, 1H, pyrrolone H-5), 3.94 (dt, *J* = 14.6, 7.6 Hz, 1H, one H of Ar-CH_2-$\underline{CH_2}$-), 3.29 – 3.21# (m, 1H, one H of Ar-CH_2-$\underline{CH_2}$-), 2.85 – 2.74 (m, 1H, one H of Ar-$\underline{CH_2}$-CH_2-), 2.70 – 2.61 (m, 1H, one H of Ar-$\underline{CH_2}$-CH_2-), 2.34 (s, 3H, -CO-$\underline{CH_3}$), 1.94 – 1.81 (m, 1H, cyclohexyl H-1), 1.73 – 1.45 (m, 4H, cyclohexyl -CH_2-), 1.36 – 0.81 (m, 6H, cyclohexyl -CH_2-); ^{13}C NMR (DEPTQ, 101 MHz, DMSO-d_6 δ [ppm]): 192.95 q (-$\underline{C}$O-CH_3), 165.32 q (pyrrolone C-2), 155.79 q (4-hydroxyphenethyl C-4), 153.83 q (pyrrolone C-3), 129.45 (4-hydroxyphenethyl C-2,6), 128.57 q (4-hydroxyphenethyl C-1), 118.74 q (pyrrolone C-4), 115.20 (4-hydroxyphenethyl C-3,5), 60.31 (pyrrolone C-5), 43.14 (-$\underline{C}H_2$-CH_2-Ar), 38.98*# (cyclohexyl C-1), 32.45 (-CH_2-$\underline{C}H_2$-Ar), 30.45 (-CO-$\underline{C}H_3$), 29.29 26.98, 26.53, 26.20, 26.04 (cyclohexyl -CH_2-); LRMS (ESI+) *m/z*: 344 [M+H]+, HRMS (ESI+): *m/z* calcd for $C_{20}H_{26}NO_4^+$: 344.1856 [M+H]+, found 344.1856; HPLC retention time: 19.68 min, > 99% (M2).

4-Benzoyl-5-(4-fluorophenyl)-3-hydroxy-1-phenethyl-1,5-dihydro-2*H*-pyrrol-2-one (**25**)

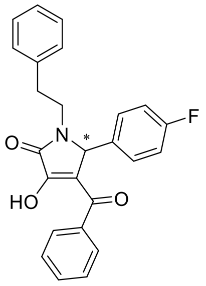

4-Fluorobenzaldehyde (84.5 mg, 681 µmol, 1.0 eq), ethyl 2,4-dioxo-4-phenylbutanoate (**44**, 150 mg, 681 µmol, 1.0 eq), and 2-phenylethyl amine (82.5 mg, 681 µmol, 1.0 eq) were placed in a microwave tube and dissolved in acetic acid (1.0 mL). The reaction mixture was heated to 90 °C and stirred overnight. After complete conversion of the starting materials monitored by LC-MS, the colorless precipitate was separated by filtration and then washed with Et_2O. This step was performed several times to obtain the title compound as a colorless solid (153 mg, 56%). ^{1}H NMR (400 MHz, DMSO-d_6 δ [ppm]): 11.79 (bs, 1H, pyrrolone -OH), 7.69 – 7.64 (m, 2H, benzoyl H-2,6), 7.59 – 7.50 (m, 1H, benzoyl H-4), 7.47 – 7.40 (m, 2H, benzoyl H-3,5), 7.36 – 7.25 (m, 4H, 4-fluorophenyl H-2,6 and phenethyl H-2,6), 7.25 – 7.18 (m, 1H, phenethyl H-4), 7.18 – 7.08 (m, 4H, 4-fluorophenyl H-3,5 and phenethyl H-3,5), 5.35 (s, 1H, pyrrolone H-5), 3.90 – 3.73 (m, 1H, one H of Ar-CH_2-$C\underline{H}_2$-), 2.91 – 2.75 (m, 2H, one H of Ar-CH_2-$C\underline{H}_2$- and one H of Ar-$C\underline{H}_2$-CH_2-), 2.76 – 2.61 (m, 1H, one H of Ar-$C\underline{H}_2$-CH_2-); ^{13}C NMR (DEPTQ, 101 MHz, DMSO-d_6 δ [ppm]): 189.03 q (benzoyl C=O), 164.86 q (pyrrolone C-2), 161.91 q (d, J = 244.3 Hz, 4-fluorophenyl C4), 151.39 q (pyrrolone C-3), 138.62 q (phenethyl C-1), 138.01 q (benzoyl C-1), 132.55 (benzoyl C-4), 132.29 q (d, J =2.8 Hz, 4-fluorophenyl C-1), 129.89 (d, J =8.3 Hz, 4-fluorophenyl C-2,6), 128.70 (benzoyl C-2,6), 128.60 (phenethyl C-3,5), 128.48 (phenethyl C-2,6), 128.14 (benzoyl C-3,5), 126.42 (phenethyl C-4), 119.16 q (pyrrolone C-4), 115.52 (d, J = 21.5 Hz, 4-fluorophenyl C-3,5), 60.13 (pyrrolone C-5), 41.62 (-$\underline{C}H_2$-CH_2-Ar), 33.59 (-CH_2-$\underline{C}H_2$-Ar); LRMS (ESI$^+$) *m/z*: 402 $[M+H]^+$. HRMS (ESI$^+$): *m/z* calcd for $C_{25}H_{21}FNO_3^+$: 402.1500 $[M+H]^+$, found: 402.1498; HPLC retention time: 18.56 min, >99% (M1).

4-Benzoyl-1-(4-fluorophenethyl)-5-(4-fluorophenyl)-3-hydroxy-1,5-dihydro-2*H*-pyrrol-2-one (**26**)

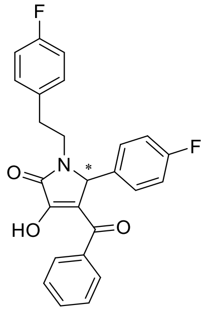

4-Fluorobenzaldehyde (56.4 mg, 454 µmol, 1.0 eq), ethyl 2,4-dioxo-4-phenylbutanoate (**44**, 100 mg, 454 µmol, 1.0 eq), and 2-(4-fluorophenyl)ethan-1-amine (63.2 mg, 0.454 mmol, 1.0 eq) were placed in a microwave tube and dissolved in acetic acid (1.0 mL). The reaction mixture was heated to 95 °C and stirred overnight. After complete conversion of the starting materials monitored by LC-MS a colorless precipitate the colorless precipitate was separated by filtration and then washed with Et_2O. This step was performed several times to obtain the title compound as a yellowish solid (110 mg, 58%). ^{1}H NMR (400 MHz, DMSO-d_6 δ [ppm]): 11.76 (bs, 1H, pyrrolone -OH), 7.72 – 7.61 (m, 2H, benzoyl H-2,6), 7.58 – 7.50 (m, 1H, benzoyl H-4), 7.49 – 7.40 (m, 2H, benzoyl H-3,5), 7.40 – 7.32 (m, 2H, 4-fluorophenyl H-2,6), 7.25 – 6.94 (m, 6H, 4-fluorophenyl H-3,5 and 4-fluorophenethyl H-2,3,5,6), 5.40 (s, 1H, pyrrolone H-5), 3.90 – 3.71 (m, 1H, one H of -$C\underline{H}_2$-CH_2-Ar), 2.88 – 2.76 (m, 2H, one H of -$C\underline{H}_2$-CH_2-Ar and one H of -CH_2-$C\underline{H}_2$-Ar), 2.76 – 2.64 (m, 1H, one H of -CH_2-$C\underline{H}_2$-Ar); ^{13}C NMR (DEPTQ, 151 MHz, DMSO-d_6 δ [ppm]): 188.99 q (benzoyl C=O), 164.87 q (pyrrolone C-2), 161.87 q (d, J = 244.6 Hz, 4-fluorophenyl C-4), 160.93 q (d, J = 243.1 Hz, 4-fluorophenethyl C-4), 151.30 q (pyrrolone C-3), 137.98 q (benzoyl C-1), 134.75 q (d, J = 3.0 Hz, 4-fluorophenethyl C-1), 132.48 (benzoyl C-4), 132.27 q (d, J = 2.8 Hz, 4-fluorophenyl C-1), 130.39 (d, J = 8.0 Hz, 4-fluorophenethyl C-2,6), 129.83 (d, J = 8.3 Hz, 4-fluorophenyl C-2,6), 128.64 (benzoyl C-2,6), 128.09 (benzoyl C-3,5), 119.15 q (pyrrolone C-4), 115.47 (d, J = 21.6 Hz, 4-fluorophenyl C-3,5), 115.10 (d, J = 21.1 Hz, 4-fluorophenethyl C-3,5), 60.03 (pyrrolone C-5), 41.58 (-$\underline{C}H_2$-$\underline{C}H_2$-Ar), 32.62 (-CH_2-$\underline{C}H_2$-Ar); LRMS (ESI$^+$) *m/z*: 420 $[M+H]^+$. HRMS (ESI$^+$): *m/z* calcd for $C_{25}H_{20}F_2NO_3^+$: 420.1406 $[M+H]^+$, found 420.1406; HPLC retention time: 18.60 min, > 99% (M1).

4-Benzoyl-5-(4-fluorophenyl)-3-hydroxy-1-(3-hydroxyphenethyl)-1,5-dihydro-2*H*-pyrrol-2-one (**27**)

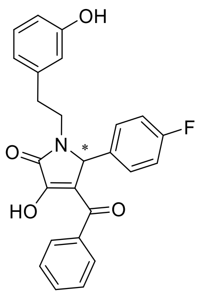

4-Fluorobenzaldehyde (56.4 mg, 454 µmol, 1.0 eq), ethyl 2,4-dioxo-4-phenylbutanoate (**44**, 100 mg, 454 µmol, 1.0 eq), and 3-(2-methoxyphenyl)ethanamine (68.7 mg, 454 µmol, 1.0 eq) were placed in a microwave tube and dissolved in acetic acid (2.0 mL). The reaction mixture was heated to 90 °C and stirred for 18 h. After complete conversion of the starting materials, the reaction mixture was cooled to room temperature and concentrated under reduced pressure. The crude intermediate 4-benzoyl-5-(4-fluorophenyl)-3-hydroxy-1-(3-methoxyphenethyl)-1,5-dihydro-2*H*-pyrrol-2-one (**47**) was dissolved under nitrogen atmosphere in dry CH_2Cl_2 (2.0 mL). Boron tribromide (314 mg, 1.25 mmol, 2.8 eq) was added to the solution. The reaction mixture was stirred for 3 h at room temperature. After complete conversion of starting materials, water (3 mL) was added to quench the reaction. The mixture was stirred for further 10 min at room temperature and extracted with a mixture of methanol/CH_2Cl_2 (1:9). The organic layer was combined, dried over sodium sulfate, and concentrated under reduced pressure. The crude product was dissolved in glacial acetic acid (1 mL) and diethyl ether (10 mL) was added to the solution. The colorless precipitate was separated by filtration and washed with Et_2O, then purified by preparative HPLC (acetonitrile/water (0.1% TFA): gradient 25-55%) to obtain the title compound as a colorless solid (12 mg, 6%).^{1}H NMR (400 MHz, DMSO-d_6 δ [ppm]): 11.78 (bs, 1H, pyrrolone -OH), 9.32 (s, 1H, 3-hydroxyphenethyl -OH), 7.76 – 7.61 (m, 2H, benzoyl H-2,6), 7.60 – 7.50 (m, 1H, benzoyl H-4), 7.47 – 7.39 (m, 2H, benzoyl H-3,5), 7.37 – 7.30 (m, 2H, 4-fluorophenyl H-2,6), 7.18 – 7.11 (m, 2H, 4-fluorophenyl H-3,5), 7.07 (dd, J = 7.8, 7.8 Hz, 1H, 3-hydroxyphenethyl H-5), 6.63 – 6.58 (m, 1H, 3-hydroxyphenethyl H-4), 6.56 – 6.51 (m, 2H, 3-hydroxyphenethyl H-2,6), 5.34 (s, 1H, pyrrolone H-5), 3.82 – 3.73 (m, 1H, one H of Ar-CH_2-$C\underline{H}_2$-), 2.84 – 2.70 (m, 2H, one H of Ar-CH_2-$C\underline{H}_2$- and one H of Ar-$C\underline{H}_2$-CH_2-), 2.61 – 2.53 (m, 1H, one H of Ar-$C\underline{H}_2$-CH_2-); ^{13}C NMR (DEPTQ, 101 MHz, DMSO-d_6 δ [ppm]): 164.89 q (pyrrolone C-2), 161.89 q (d, J = 244.4 Hz, 4-fluorophenyl C-4), 157.42 q (3-hydroxyphenethyl C-3), 139.97 q (3-hydroxyphenethyl C-1), 138.23 q (benzoyl C-1), 132.48 (benzoyl C-4), 129.88 (d, 4-fluorophenyl C-2,6), 129.45 (3-hydroxyphenethyl C-5), 128.70 (benzoyl C-2,6), 128.12 (benzoyl C-3,5), 119.14 (3-hydroxyphenethyl C-2 or C-6), 119.03 q (pyrrolone C-4), 115.49 (d, J = 21.4 Hz, 4-fluorophenyl C-3,5), 115.43 (3-hydroxyphenethyl C-2 or C-6), 113.41 (3-hydroxyphenethyl C-4), 60.19 (pyrrolone C-5), 41.65 (-$\underline{C}H_2$-CH_2-Ar), 33.65 (-CH_2-$\underline{C}H_2$-Ar); LRMS (ESI$^+$) *m/z*: 418 [M+H]$^+$, HRMS (ESI$^+$): *m/z* calcd for $C_{25}H_{21}FNO_4^+$: 418.1449 [M+H]$^+$, found: 418.1450; HPLC retention time 17.04 min, 99% (M1).

4-Benzoyl-5-(4-fluorophenyl)-3-hydroxy-1-(2-hydroxyphenethyl)-1,5-dihydro-2*H*-pyrrol-2-one (**28**)

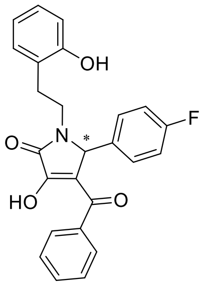

4-Fluorobenzaldehyde (56.36 mg, 454 µmol, 1.0 eq), ethyl 2,4-dioxo-4-phenylbutanoate (**44**, 100.0 mg, 454 µmol, 1.0 eq), and 2-(2-aminoethyl)phenol (62.29 mg, 454 µmol, 1.0 eq) were placed in a microwave tube and dissolved in acetic acid (2.0 mL). The reaction mixture was heated to 90 °C and stirred for 24 h. After complete conversion of the starting materials, the reaction mixture was cooled down to room temperature and Et_2O was added. The colorless precipitate was separated by filtration and washed with Et_2O, to obtain the title compound as a colorless solid (18.90 mg, 10%). ^{1}H NMR (500 MHz, DMSO-d_6 δ [ppm]): 11.73 (bs, 1H, pyrrolone -OH), 9.36 (s, 1H, 2-hydroxyphenethyl -OH), 7.68 – 7.63 (m, 2H, benzoyl H-2,6), 7.56 – 7.51 (m, 1H, benzoyl H-4), 7.45 – 7.39 (m, 2H, benzoyl H-3,5), 7.28 – 7.21 (m, 2H, 4-fluorophenyl H-2,6), 7.15 – 7.08 (m, 2H, 4-fluorophenyl H-3,5), 7.04 (td, J = 7.7, 1.7 Hz, 1H, 2-hydroxyphenethyl H-4), 6.97 (dd, J = 7.7, 1.7 Hz, 1H, 2-hydroxyphenethyl H-6), 6.78 (dd, J = 7.7, 1.2 Hz, 1H, 2-hydroxyphenethyl H-3), 6.70 (td, J = 7.7, 1.2 Hz, 1H, 2-hydroxyphenethyl H-5), 5.30 (s, 1H, pyrrolone H-5), 3.87 – 3.78 (m, 1H, one H of Ar-CH_2-$C\underline{H}_2$-), 2.86 – 2.73 (m, 2H, one

H of Ar-C<u>H</u>$_2$-CH$_2$- and one H of Ar-CH$_2$-C<u>H</u>$_2$-), 2.71 – 2.62 (m, 1H, one H of Ar-C<u>H</u>$_2$-CH$_2$-); ^{13}C NMR (126 MHz, DMSO-*d*$_6$, δ [ppm]): 188.80 q (benzoyl C=O), 164.72 q (pyrrolone C-2), 161.68 q (d, *J* = 244.2 Hz, 4-fluorophenyl C-4), 155.29 q (2-hydroxyphenethyl C-2), 151.53 q (pyrrolone C-3), 137.98 q (benzoyl C-1), 132.28 (benzoyl C-4 and 4-fluorophenyl C-1), 130.11 (2-hydroxyphenethyl C-6), 129.65 (d, *J* = 8.2 Hz, 4-fluorophenyl C-2,6), 128.50 (benzoyl C-2,6), 127.95 (benzoyl C-3,5), 127.48 (2-hydroxyphenethyl C-4), 124.60 q (2-hydroxyphenethyl C-1), 119.01 q (pyrrolone C-4), 118.84 (2-hydroxyphenethyl C-5), 115.24 (d, *J* = 21.5 Hz, 4-fluorophenyl C-3,5), 114.76 (2-hydroxyphenethyl C-3), 60.13 (pyrrolone C-5), 39.84 (-<u>C</u>H$_2$-CH$_2$-Ar), 28.58 (-CH$_2$-<u>C</u>H$_2$-Ar);LRMS (APCI$^-$) *m/z*: 416 [M-H]$^-$; HRMS (ESI$^+$): *m/z* calcd for $C_{25}H_{20}FNO_4Na^+$: 440.1269 [M+Na]$^+$, found: 440.1269; HPLC retention time 17.27 min, > 99% (M1).

4-Benzoyl-5-(4-fluorophenyl)-3-hydroxy-1-(4-hydroxybenzyl)-1,5-dihydro-2*H*-pyrrol-2-one (**29**)

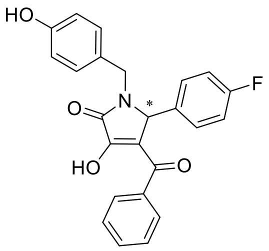

4-Fluorobenzaldehyde (56.4 mg, 454 µmol, 1.0 eq), ethyl 2,4-dioxo-4-phenylbutanoate (**44**, 100 mg, 454 µmol, 1.0 eq), and 4-hydroxybenzamine (55.9 mg, 454 µmol, 1.0 eq) were placed in a microwave tube and dissolved in acetic acid (1.0 mL). The reaction mixture was heated to 95 °C and stirred for 23 h. The colorless precipitate was separated by filtration and then washed with Et$_2$O. This step was performed several times to obtain the title compound as a colorless solid (66 mg, 36%). ^{1}H NMR (400 MHz, DMSO-*d*$_6$ δ [ppm]): 11.93 (bs, 1H, pyrrolone -OH), 9.43 (s, 1H, 4-hydroxybenzyl -OH), 7.72 – 7.63 (m, 2H, benzoyl H-2,6), 7.60 – 7.50 (m, 1H, benzoyl H-4), 7.47 – 7.38 (m, 2H, benzoyl H-3,5), 7.32 – 7.25 (m, 2H, 4-fluorophenyl H-2,6), 7.16 – 7.09 (m, 2H, 4-fluorophenyl H-3,5), 6.95 – 6.85 (m, 2H, 4-hydroxybenzyl H-2,6), 6.73 – 6.65 (m, 2H, 4-hydroxybenzyl H-3,5), 5.18 (s, 1H, pyrrolone H-5), 4.78 (d, *J* = 14.9 Hz, 1H, one H of -CH$_2$-), 3.60 (d, *J* = 14.9 Hz, 1H, one H of -CH$_2$-); ^{13}C NMR (DEPTQ, 151 MHz, DMSO-*d*$_6$ δ [ppm]): 188.92 q (benzoyl C=O), 165.03 q (pyrrolone C-2), 161.84 q (d, *J* = 244.3 Hz, 4-fluorophenyl C-4), 156.72 q (4-hydroxybenzyl C-4), 151.23 q (pyrrolone C-3),137.91 q (benzoyl C-1), 132.51 (benzoyl C-4), 132.04 (4-fluorophenyl C-1), 129.86 (d, *J* = 8.4 Hz, 4-fluorophenyl C-2,6), 129.21 (4-hydroxybenzyl C-2,6), 128.68 (benzoyl C-2,6), 128.08 (benzoyl C-2,6), 126.62 q (4-hydroxybenzyl C-1), 119.23 q (pyrrolone C-4), 115.45 (d, *J* = 21.6 Hz, 4-fluorophenyl C-3,5), 115.33 (4-hydroxybenzyl C-3,5), 59.66 (pyrrolone C-5), 43.40 (-<u>C</u>H$_2$-). LRMS (ESI$^+$) *m/z*: 404 [M+H]$^+$. HRMS (ESI$^+$): *m/z* calcd for $C_{24}H_{19}FNO_4^+$: 404.1293 [M+H]$^+$, found: 404.1293 ; HPLC retention time: 15.52 min, 99% (M1).

4-Benzoyl-5-(4-fluorophenyl)-3-hydroxy-1-(4-hydroxyphenyl)-1,5-dihydro-2*H*-pyrrol-2-one (**30**)

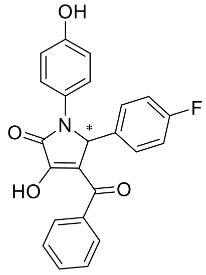

4-Fluorobenzaldehyde (56.4 mg, 454 µmol, 1.0 eq), ethyl 2,4-dioxo-4-phenylbutanoate (**44**, 100 mg, 454 µmol, 1.0 eq), and 4-aminophenol (49.5 mg, 0.454 mmol, 1.0 eq) were placed in a microwave tube and dissolved in acetic acid (1.0 mL). The reaction mixture was heated to 95 °C and stirred overnight. After complete conversion of the starting materials monitored by LC-MS, the precipitate was separated by filtration and then washed with Et$_2$O. This step was performed several times to obtain the title compound as a yellowish solid (63 mg, 36%). ^{1}H NMR (400 MHz, DMSO-*d*$_6$ δ [ppm]): 11.84 (bs, 1H, pyrrolone -OH), 9.46 (s, 1H, 4-hydroxyphenyl -OH), 7.74 – 7.67 (m, 2H, benzoyl H-2,6), 7.62 – 7.51 (m, 1H, benzoyl H-4), 7.48 – 7.42 (m, 2H, benzoyl H-3,5), 7.42 – 7.36 (m, 2H, 4-fluorophenyl H-2,6), 7.36 – 7.30 (m, 2H, 4-fluorophenyl H-3,5), 7.08 – 6.97 (m, 2H, 4-hydroxyphenyl H-2,6), 6.76 – 6.64 (m, 2H, 4-hydroxyphenyl H-3,5), 6.19 (s, 1H, pyrrolone H-5); ^{13}C NMR (DEPTQ, 101 MHz, DMSO-*d*$_6$ δ [ppm]): 189.23 q (benzoyl C=O), 164.06 q (pyrrolone C-2), 161.54 q (d, *J* = 243.9 Hz, 4-fluorophenyl C-4), 155.26 q (4-hydroxyphenyl C-4), 150.62 q (pyrrolone C-3), 138.00 q (benzoyl C-1), 132.82 q (d, *J* = 2.7 Hz, 4-fluorophenyl C-1), 132.65 (benzoyl C-4), 129.85 (d, *J* = 8.3 Hz, 4-fluorophenyl C-2,6), 128.75 (benzoyl C-3,5), 128.19 (benzoyl C-2,6), 127.57 (4-hydroxyphenyl C-1), 124.87 (4-hydroxyphenyl C-2,6), 119.41q (pyrrolone C-4), 115.23 (4-hydroxyphenol C-3,5), 115.13 (d, *J* = 21.2 Hz, 4-fluorophenyl C-3,5), 61.04 (pyrrolone

C-5); LRMS (ESI$^+$) *m/z*: 390 [M+H]$^+$; HRMS (ESI$^+$): *m/z* calcd for $C_{23}H_{17}FNO_4^+$: 390.1136 [M+H]$^+$, found: 390.1136; HPLC retention time: 16.29 min, > 99% (M1).

4-Benzoyl-5-(4-bromophenyl)-3-hydroxy-1-(4-hydroxyphenethyl)-1,5-dihydro-2*H*-pyrrol-2-one (**31**)

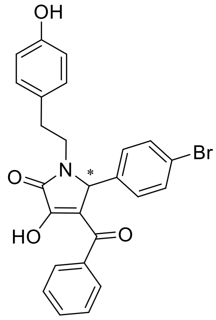

4-Bromobenzaldehyde (42.0 mg, 227 µmol, 1.0 eq), ethyl 2,4-dioxo-4-phenylbutanoate (**44**, 50.0 mg, 227 µmol, 1.0 eq), and 4-(2-aminoethyl)phenol (31.1 mg, 227 µmol, 1.0 eq) were placed in a microwave tube and dissolved in acetic acid (1.0 mL). The reaction mixture was heated to 95 °C and stirred for 20 h. After complete conversion of the starting materials monitored by LC-MS, the colorless precipitate was separated by filtration and then washed with Et_2O. This step was performed several times to obtain the title compound as a white solid (59 mg, 54%). ^{1}H NMR (400 MHz, DMSO-d_6 δ [ppm]): 11.91 (bs, 1H, pyrrolone -OH), 9.24 (s, 1H, 4-hydroxyphenethyl -OH), 7.71 – 7.59 (m, 2H, benzoyl H-2,6), 7.58 – 7.47 (m, 3H, benzoyl H-4 and 4-bromophenyl H-2,6), 7.47 – 7.35 (m, 2H, benzoyl H-3,5), 7.29 – 7.20 (m, 2H, 4-bromophenyl H-3,5), 6.94 – 6.87 (m, 2H, 4-hydroxyphenethyl H-2,6), 6.76 – 6.49 (m, 2H, 4-hydroxyphenethyl H-3,5), 5.28 (s, 1H, pyrrolone H-5), 3.94 – 3.69 (m, 1H, one H of Ar-CH$_2$-C$\underline{H}_2$-), 2.87 – 2.65 (m, 2H, one H of Ar-CH$_2$-C$\underline{H}_2$- and one H of Ar-C$\underline{H}_2$-CH$_2$-), 2.64 – 2.52 (m, 1H, one H of Ar-C$\underline{H}_2$-CH$_2$-); ^{13}C NMR (DEPTQ, 151 MHz, DMSO-d_6 δ [ppm]): 188.87 q (benzoyl C=O), 164.91 q (pyrrolone C-2), 155.83 q (4-hydroxyphenethyl C-4), 151.63 q (pyrrolone C-3), 138.03 q (benzoyl C-1), 135.80 q (4-bromophenyl C-4), 132.43 (benzoyl C-4), 131.52 (4-bromophenyl C-2,6 or C-3,5), 129.96 (4-bromophenyl C-2,6 or C-3,5), 129.44 (4-hydroxyphenethyl C-2,6), 128.61 (benzoyl C-2,6), 128.50 q (4-hydroxyphenethyl C-1), 128.06 (benzoyl C-3,5), 121.34 q (4-bromophenyl C-1), 118.85 (pyrrolone C-4), 115.23 (4-hydroxyphenethyl C-3,5), 60.25 (pyrrolone C-5), 41.94 (-$\underline{C}$H$_2$-CH$_2$-Ar), 32.74 (-CH$_2$-$\underline{C}$H$_2$-Ar); LRMS (ESI$^+$) *m/z*: 480 [M(^{81}Br)+H]$^+$; HRMS (ESI$^+$): *m/z* calcd for $C_{25}H_{21}BrNO_4^+$: 478.0648 [M(^{79}Br)+H]$^+$, Found: 478.0649; HPLC retention time: 17.52 min, > 99% (M1).

4-Benzoyl-5-(3-bromophenyl)-3-hydroxy-1-(4-hydroxyphenethyl)-1,5-dihydro-2*H*-pyrrol-2-one (**32**)

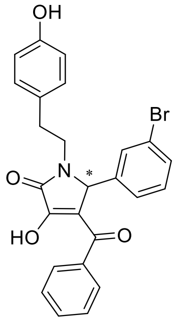

3-Bromobenzaldehyde (42.0 mg, 227 µmol, 1.0 eq), ethyl 2,4-dioxo-4-phenylbutanoate (**44**, 50.0 mg, 227 µmol, 1.0 eq), and 4-hydroxyphenethylamine (31.1 mg, 227 µmol, 1.0 eq) were placed in a microwave tube and dissolved in acetic acid (1.0 mL). The reaction mixture was heated to 90 °C and stirred overnight. After complete conversion of the starting materials monitored by LC-MS, the colorless precipitate was separated by filtration and then washed with Et_2O. This step was performed several times to obtain the title compound as a colorless solid (55 mg, 51%). ^{1}H NMR (400 MHz, DMSO-d_6 δ [ppm]: 11.91 (bs, 1H, pyrrolone -OH), 9.25 (s, 1H, 4-hydroxyphenethyl -OH), 7.69 – 7.64 (m, 2H, benzoyl H-2,6), 7.58 – 7.52 (m, 1H, benzoyl H-4), 7.50 – 7.40 (m, 4H, benzoyl H-3,5, 3-bromophenyl H-2,4), 7.28 – 7.11 (m, 2H, 3-bromophenyl H-5,6), 6.96 – 6.88 (m, 2H, 4-hydroxyphenethyl H-2,6), 6.70 – 6.65 (m, 2H, 4-hydroxyphenethyl H-3,5), 5.31 (s, 1H, pyrrolone H-5), 3.83 – 3.74 (m, 1H, one H of Ar-CH$_2$-C$\underline{H}_2$-), 2.80 – 2.70 (m, 2H, one H of Ar-CH$_2$-C$\underline{H}_2$- and one H of Ar-C$\underline{H}_2$-CH$_2$-), 2.62 – 2.54 (m, 1H, one H of Ar-C$\underline{H}_2$-CH$_2$-); ^{13}C NMR (DEPTQ, 101 MHz, DMSO-d_6 δ [ppm]): 188.94 q (benzoyl C=O), 164.87 q (pyrrolone C-2), 155.87 q (4-hydroxyphenethyl C-4), 151.77 q (pyrrolone C-3), 139.16 q (3-bromophenyl C-1), 138.01 q (benzoyl C-1), 132.54 (benzoyl C-4), 131.24 (3-bromophenyl C-2 or C-4 or C-5), 130.91 (3-bromophenyl C-2 or C-4 or C-5), 130.88 (3-bromophenyl C-2 or C-4 or C-5), 129.52 (4-hydroxyphenethyl C-2,6), 128.69

(benzoyl C-2,6), 128.56 (4-hydroxyphenethyl C-1), 128.14 (benzoyl C-3,5), 126.48 (3-bromophenyl C-6), 121.69 q (3-bromophenyl C-3), 118.70 q (pyrrolone C-4), 115.25 (4-hydroxyphenethyl C-3,5), 60.28 (pyrrolone C-5), 42.02 (Ar-CH_2-$\underline{C}H_2$-), 32.80 (Ar-$\underline{C}H_2$-CH_2-); LRMS (ESI$^+$) *m/z*: 480 $[M+H]^+$; HRMS (ESI$^+$): *m/z* calcd for $C_{25}H_{21}BrNO_4^+$: 478.0649 $[M+H]^+$; found: 478.0643; HPLC retention time: 17.30 min, > 99% (M1). The attached ^{1}H and ^{13}C NMR spectra contains residual solvent signals (CH_2Cl_2).

4-Benzoyl-5-(4-chlorophenyl)-3-hydroxy-1-(4-hydroxyphenethyl)-1,5-dihydro-2*H*-pyrrol-2-one (**33**)

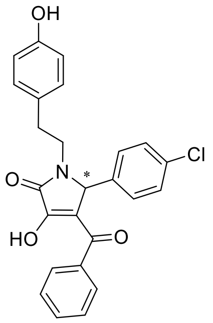

4-Chlorobenzaldehyde (31.9 mg, 227 µmol, 1.0 eq), ethyl 2,4-dioxo-4-phenylbutanoate (**44**, 50.0 mg, 227 µmol, 1.0 eq), and 4-hydroxyphenethylamine (31.1 mg, 227 µmol, 1.0 eq) were placed in a microwave tube and dissolved in acetic acid (1.0 mL). The reaction mixture was heated to 95 °C and stirred overnight. After complete conversion of the starting materials monitored by LC-MS, the colorless precipitate was separated by filtration and then washed with Et_2O. This step was performed several times to obtain the title compound as a white solid (9 mg, 9%). ^{1}H NMR (400 MHz, DMSO-d_6 δ [ppm]): 11.88 (bs, 1H, pyrrolone -OH), 9.24 (s, 1H, 4-hydroxyphenethyl -OH), 7.71 – 7.59 (m, 2H, benzoyl H-2,6), 7.57 – 7.46 (m, 1H, benzoyl H-4), 7.44 – 7.34 (m, 4H, benzoyl H-3,5 and 4-chlorophenyl H-2,6), 7.33 – 7.21 (m, 2H, 4-chlorophenyl H-3,5), 6.95 – 6.88 (m, 2H, 4-hydroxyphenethyl H-2,6), 6.69 – 6.64 (m, 2H, 4-hydroxyphenethyl H-3,5), 5.28 (s, 1H, pyrrolone H-5), 3.82 – 3.69 (m, 1H, one H of Ar-CH_2-$C\underline{H}_2$-), 2.79 – 2.65 (m, 2H, one H of Ar-CH_2-$C\underline{H}_2$- and one H of Ar-$C\underline{H}_2$-CH_2-), 2.60 – 2.53$^\#$ (m, 1H, one H of Ar-CH_2-$C\underline{H}_2$-); ^{13}C NMR (151 MHz, DMSO-d_6 δ [ppm]): 188.34 q (benzoyl C=O), 165.43 q (pyrrolone C-2), 155.83 q (4-hydroxyphenethyl C-4), 153.08* q (pyrrolone C-3), 138.41 q (benzoyl C-1), 136.00 q (4-chlorophenyl C-1), 132.59 q (4-chlorophenyl C-4), 132.10 (benzoyl C-4), 129.62 (4-chlorophenyl C-2,6), 129.45 (4-hydroxyphenethyl C-2,6), 128.63 (benzoyl C-2,6), 128.58 q (4-hydroxyphenethyl C-1), 128.53 (4-chlorophenyl C-3,5), 127.95 (benzoyl C-3,5), 118.15 q (pyrrolone C-4), 115.25 (4-hydroxyphenethyl C-3,5), 60.19 (pyrrolone C-5), 41.94 (-$\underline{C}H_2$-CH_2-Ar), 32.75 (-CH_2-$\underline{C}H_2$-Ar); LRMS (ESI$^+$) *m/z*: 434 $[M+H]^+$; HRMS (ESI$^+$): *m/z* calcd for $C_{25}H_{21}ClNO_4^+$: 434.1154 $[M+H]^+$; found: 434.1155; HPLC retention time: 17.36 min, 99% (M1).

4-Benzoyl-5-cyclohexyl-3-hydroxy-1-(4-hydroxyphenethyl)-1,5-dihydro-2*H*-pyrrol-2-one (**34**)

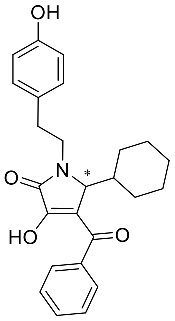

Cyclohexanecarbaldehyde (25.5 mg, 227 µmol, 1.0 eq), ethyl 2,4-dioxo-4-phenylbutanoate (**44**, 50.0 mg, 227 µmol, 1.0 eq), and 4-(2-aminoethyl)phenol (31.1 mg, 227 µmol, 1.0 eq) were placed in a microwave tube and dissolved in acetic acid (1.0 mL). The reaction mixture was heated to 80 °C and stirred for 16 h. After complete conversion of the starting materials monitored by LC-MS, the colorless precipitate was separated by filtration and washed with Et_2O. This step was performed several times to obtain the title compound as a colorless solid (24 mg, 26%). ^{1}H NMR (400 MHz, DMSO-d_6 δ [ppm]): 11.16 (bs, 1H, pyrrolone -OH), 9.23 (s, 1H, 4-hdroxyphenyl -OH), 7.77 – 7.72 (m, 2H, benzoyl H-2,6), 7.65 – 7.58 (m, 1H, benzoyl H-4), 7.55 – 7.48 (m, 2H, benzoyl H-3,5), 7.06 – 6.99 (m, 2H, 4-hydroxyphenethyl H-2,6), 6.72 – 6.63 (m, 2H, hydroxyphenethyl H-3,5), 4.42 (d, J = 2.6 Hz, 1H, pyrrolone H-5), 3.96 (dt, J = 14.5, 7.5 Hz, 1H, one H of Ar-CH_2-$C\underline{H}_2$-), 3.29 – 3.20$^\#$ (m, 1H, one H of Ar-CH_2-$C\underline{H}_2$-), 2.89 – 2.64 (m, 2H, Ar-$C\underline{H}_2$-CH_2-), 1.95 – 1.79 (m, 1H, cyclohexyl H-1), 1.70

– 1.35 (m, 5H, cyclohexyl -CH_2-), 1.28 – 0.46 (m, 6H, cyclohexyl -CH_2-); ^{13}C NMR (DEPTQ, 101 MHz, DMSO-d_6 δ [ppm]): 190.37 q (benzoyl C=O), 164.87 q (pyrrolone C-2), 155.82 q (4-hydroxylphenyl C-4),150.41 q (pyrrolone C-3), 137.57 q (benzoyl C-1), 132.95 (benzoyl C-4), 129.52 (4-hydroxyphenethyl C-2,6), 129.11 (benzoyl C-2,6), 128.70 q (4-hydroxyphenethyl C-1), 128.46 (benzoyl C-3,5), 118.10 q (pyrrolone C-4), 115.20 (4-hydroxyphenethyl C-3,5), 60.89 (pyrrolone C-5), 42.04 (-$\underline{C}H_2$-CH_2-Ar), 38.73 (cyclohexyl C-1), 32.76 (-CH_2-$\underline{C}H_2$-Ar), 29.82, 26.62, 26.30, 26.14, 25.81 (cyclohexyl C-2,3,4,5,6); LRMS (ESI$^+$) *m/z*: 406 [M+H]$^+$; HRMS (ESI$^+$): *m/z* calcd for $C_{25}H_{28}NO_4^+$: 406.2013 [M+H]$^+$, found 406.2014 ; HPLC retention time: 17.70 min, 96% (M1). The attached ^1H and ^{13}C NMR spectra contain residual solvent signals (MeOH).

4-Benzoyl-3-hydroxy-1-(4-hydroxyphenethyl)-5-phenyl-1,5-dihydro-2H-pyrrol-2-one (**35**)

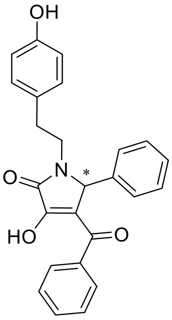

Benzaldehyde (48.2 mg, 454 µmol, 1.0 eq), ethyl 2,4-dioxo-4-phenylbutanoate (**44**, 100 mg, 454 µmol, 1.0 eq), and 4-(2-aminoethyl)phenol (62.3 mg, 0.454 mmol, 1.0 eq) were placed in a microwave tube and dissolved in acetic acid (1.0 mL). The reaction mixture was heated to 95 °C and stirred for 20 h. After cooling to room temperature, the colorless precipitate was separated by filtration and then washed with Et_2O. This step was performed several times to obtain the title compound as a yellowish solid (70 mg, 39%). ^1H NMR (400 MHz, DMSO-d_6 δ [ppm]): 11.76 (bs, 1H, pyrrolone -OH), 9.24 (s, 1H, 4-hydroxyphenethyl -OH), 7.68 – 7.63 (m, 2H, benzoyl H-2,6), 7.57 – 7.51 (m, 1H, benzoyl H-4), 7.46 – 7.40 (m, 2H, benzoyl H-3,5), 7.35 – 7.24 (m, 5H, phenyl H-2,3,4,5,6), 6.93 – 6.88 (m, 2H, 4-hydroxyphenethyl H-2,6), 6.70 – 6.64 (m, 2H, 4-hydroxyphenethyl H-3,5), 5.29 (s, 1H, pyrrolone H-5), 3.81 – 3.70 (m, 1H, one H of Ar-CH_2-$C\underline{H}_2$-), 2.80 – 2.65 (m, 2H, one H of Ar-CH_2-$\underline{C}H_2$- and one H of Ar-$C\underline{H}_2$-CH_2-), 2.58 – 2.52* (m, J = 7.9 Hz, 1H, one H of Ar-$C\underline{H}_2$-CH_2-); ^{13}C NMR (DEPTQ, 151 MHz, DMSO-d_6 δ [ppm]): 188.99 q (benzoyl C=O), 164.82 q (pyrrolone C-2), 155.81 q (4-hydroxylphenyl C-4), 151.30 q (pyrrolone C-3), 138.00 q (benzoyl C-1), 136.10 q (phenyl C-1), 132.44 (benzoyl C-4), 129.41 (4-hydroxyphenethyl C-2,6), 128.63 (benzoyl C-2,6), 128.61 (phenyl C-2,6 or C-3,5), 128.54 q (4-hydroxyphenethyl C-1), 128.27 (benzene C-4), 128.08 (benzoyl C-3,5), 127.68 (benzene C-2,6 or C-3,5), 119.28 q (pyrrolone C-4), 115.22 (4-hydroxyphenethyl C-3,5), 60.97 (pyrrolone C-5), 41.93 (Ar-CH_2-$\underline{C}H_2$-), 32.76 (Ar-$\underline{C}H_2$-CH_2-); LRMS (ESI$^+$) *m/z*: 400 [M+H]$^+$; HRMS (ESI$^+$): *m/z* calcd for $C_{25}H_{22}NO_4^+$: 400.1543 [M+H]$^+$, found: 400.1543; HPLC retention time: 15.39 min, > 99% (M1). The attached ^1H and ^{13}C NMR spectra contain residual solvent signals (MeOH).

4-(Cyclopropanecarbonyl)-5-(4-fluorophenyl)-3-hydroxy-1-(4-hydroxyphenethyl)-1,5-dihydro-2*H*-pyrrol-2-one (**36**)

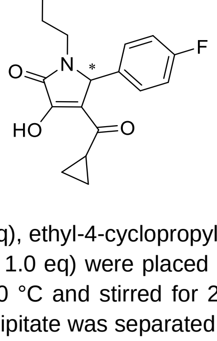

4-Fluorobenzaldehyde (33.6 mg, 271 µmol, 1.0 eq), ethyl-4-cyclopropyl-2,4-dioxobutanoate (50.0 mg, 271 µmol, 1.0 eq), and 4-(2-aminoethyl)phenol (37.2 mg, 271 µmol, 1.0 eq) were placed in a microwave tube and dissolved in acetic acid (1.0 mL). The reaction mixture was heated to 90 °C and stirred for 2 days. After complete conversion of the starting materials monitored by LC-MS, the colorless precipitate was separated by filtration and washed with Et_2O. This step was performed several times to obtain the title compound as a colorless solid (13 mg, 13%). ^1H NMR (400 MHz, DMSO-d_6 δ

[ppm]): 12.32 (bs, 1H, pyrrolone -OH), 9.22 (s, 1H, 4-hydroxyphenethyl -OH), 7.21 – 7.07 (m, 4H, 4-fluorophenyl H-2,3,5,6), 6.91 – 6.84 (m, 2H, 4-hydroxyphenethyl H-2,6), 6.68 – 6.60 (m, 2H, 4-hydroxyphenethyl H-3,5), 5.06 (s, 1H, pyrrolone H-5), 3.76 – 3.64 (m, 1H, one H of Ar-CH$_2$-C$\underline{H}_2$-), 2.80 (tt, *J* = 7.7, 4.7 Hz, 1H, cyclopropyl H-1), 2.73 – 2.59 (m, 2H, one H of Ar-CH$_2$-C$\underline{H}_2$- & one H of Ar-C$\underline{H}_2$-CH$_2$-), 2.55 – 2.43* (m, 1H, one H of Ar-C$\underline{H}_2$-CH$_2$-), 0.92 – 0.62 (m, 4H, cyclopropyl H-2,3); ^{13}C NMR (DEPTQ, 101 MHz, DMSO-*d*$_6$ δ [ppm]): 193.95 q (C=O), 165.00 q (pyrrolone C-2), 161.69 q (d, *J* = 243.8 Hz, 4-fluorophenyl C-4), 155.82 q (4-hydroxyphenethyl C-4), 154.20 q (pyrrolone C-3), 133.01 q (d, *J* = 2.9 Hz, 4-fluorophenyl C-1), 129.60 (d, *J* = 8.4 Hz, 4-fluorophenyl C-2,6), 129.45 (4-hydroxyphenethyl C-2,6), 128.54 q (4-hydroxyphenethyl C-1), 119.79 q (pyrrolone C-4), 115.33 (d, *J* = 21.1 Hz, 4-fluorophenyl C-3,5), 115.23 (4-hydroxyphenethyl C-3,5), 59.50 (pyrrolone C-5), 41.63 (Ar-CH$_2$-$\underline{C}$H$_2$-), 32.70 (Ar-$\underline{C}$H$_2$-CH$_2$-), 19.12 (cyclopropyl C-1), 10.82 (cyclopropyl C-2/3), 10.17 (cyclopropyl C-2/3); LRMS (ESI$^+$) *m/z*: 382 [M+H]$^+$; HRMS (ESI$^+$): *m/z* calcd for $C_{22}H_{21}FNO_4^+$: 382.1449 [M+H]$^+$, found 382.1443; HPLC retention time: 16.28 min, > 99% (M1).

5-(3-Bromophenyl)-4-(cyclopropanecarbonyl)-3-hydroxy-1-(4-hydroxyphenethyl)-1,5-dihydro-*2H*-pyrrol-2-one (**37**)

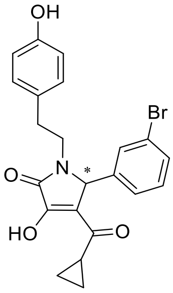

3-Bromobenzaldehyde (50.2 mg, 271 µmol, 1.0 eq), ethyl-4-cyclopropyl-2,4-dioxobutanoate (50.0 mg, 271 µmol, 1.0 eq), and 4-(2-aminoethyl)phenol (37.2 mg, 271 µmol, 1.0 eq) were placed in a microwave tube and dissolved in acetic acid (1.0 mL). The reaction mixture was heated to 90 °C and stirred for 5 h, afterwards the reaction mixture was cooled to room temperature and stirred overnight. After complete conversion of the starting materials monitored by LC-MS, the acetic acid was removed under reduced pressure. Diethyl ether was added to the crude residue and stirred for 5 min. The mixture was filtered, and the filter cake was washed with diethyl ether several times to obtain the colorless title compound in sufficient purity. This step was performed several times to obtain the title compound as a colorless solid (15 mg, 13%). ^{1}H NMR (400 MHz, DMSO-*d*$_6$ δ [ppm]): 12.49 (bs, 1H, pyrrolone -OH), 9.22 (s, 1H, 4-hydroxylphenethyl -OH), 7.48 (ddd, *J* = 7.9, 1.9, 1.0 Hz, 1H, 3-bromophenyl H-6), 7.35 (t, *J* = 1.9 Hz, 1H, 3-bromophenyl H-2), 7.27 (t, *J* = 7.9 Hz, 1H, 3-bromophenyl H-5), 7.13 – 7.06 (m, 1H, 3-bromophenyl H-4), 6.93 – 6.86 (m, 2H, 4-hydroxylphenethyl H-2,6), 6.68 – 6.61 (m, 2H, 4-hydroxylphenethyl H-3,5), 5.05 (s, 1H, pyrrolone H-5), 3.72 (ddd, *J* = 11.2, 5.2, 5.2 Hz, 1H, one H of Ar-CH$_2$-C$\underline{H}_2$-), 2.81 (tt, *J* = 7.8, 4.7 Hz, 1H, cyclopropyl H-1), 2.71 – 2.60 (m, 2H, one H of Ar-CH$_2$-C$\underline{H}_2$- and one H of Ar-C$\underline{H}_2$-CH$_2$-), 2.56 – 2.44$^{\#}$ (m, 1H, one H of Ar-C$\underline{H}_2$-CH$_2$-), 0.93 – 0.65 (m, 4H, cyclopropyl H-2,3); ^{13}C NMR (DEPTQ, 101 MHz, DMSO-*d*$_6$ δ [ppm]): 193.95 q (C=O), 165.02 q (pyrrolone C-2), 155.83 q (4-hydroxylphenyl C-4), 154.30 q (pyrrolone C-3),139.81 q (3-bromophenyl C-1), 130.95 (3-bromophenyl C-2 or C-4 or C-5), 130.75 (C-2 or C-4 or C-5), 130.68 (C-2 or C-4 or C-5), 129.48 (4-hydroxyphenethyl C-2,6), 128.53 q (4-hydroxyphenethyl C-1), 126.28 (3-bromophenyl C-6), 121.54 q (3-bromophenyl C-3), 119.48 q (pyrrolone C-4), 115.22 (4-hydroxyphenethyl C-3,5), 59.61 (pyrrolone C-5), 41.72 (Ar-CH$_2$-$\underline{C}$H$_2$-), 32.69 (Ar-$\underline{C}$H$_2$-CH$_2$-), 19.12 (cyclopropyl C-1), 10.92 (cyclopropyl C-2/3), 10.25 (cyclopropyl C-2/3); LRMS (ESI$^+$) *m/z*: 442 [M+H]$^+$; HRMS (ESI$^+$): *m/z* calcd for $C_{22}H_{21}BrNO_4^+$: 442.0648 [M+H]$^+$, found: 442.0642; HPLC retention time 19.58 min, 99% (M2).

5-(4-Fluorophenyl)-3-hydroxy-4-(4-hydroxybenzoyl)-1-(4-hydroxyphenethyl)-1,5-dihydro-2*H*-pyrrol-2-one (**38**)

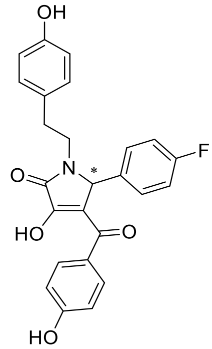

4-Fluorobenzaldehyde (52.5 mg, 423 µmol, 1.0 eq), ethyl 4-(4-hydroxyphenyl)-2,4-dioxobutanoate (**46**, 100.0 mg, 423 µmol, 1.0 eq), and 4-(2-aminoethyl)phenol (58.07 mg, 423 µmol, 1.0 eq) were placed in a microwave tube and dissolved in acetic acid (1.0 mL). The reaction mixture was heated to 90 °C and stirred for 18 h. After complete conversion of the starting materials, the reaction mixture was cooled down to room temperature and Et_2O was added. The brown precipitate was separated by filtration and washed with Et_2O, to obtain the title compound as a brown solid (31.0 mg, 17%). ^{1}H NMR (500 MHz, DMSO-d_6 δ [ppm]): 11.48 (bs, 1H, pyrrolone -OH), 10.22 (bs, 1H, 4-hydroxybenzoyl -OH), 9.21 (bs, 1H, 4-hydroxyphenethyl -OH), 7.62 – 7.57 (m, 2H, 4-hydroxybenzoyl H-2,6), 7.31 – 7.24 (m, 2H, 4-fluorophenyl H-2,6), 7.15 – 7.08 (m, 2H, 4-fluorophenyl H-3,5), 6.94 – 6.88 (m, 2H, 4-hydroxyphenethyl H-2,6), 6.78 – 6.73 (m, 2H, 4-hydroxybenzoyl H-3,5), 6.69 – 6.64 (m, 2H, 4-hydroxyphenethyl H-3,5), 5.30 (s, 1H, pyrrolone H-5), 3.79 – 3.70 (m, 1H, one H of Ar-CH$_2$-C<u>H</u>$_2$-), 2.79 – 2.67 (m, 2H, one H of Ar-C<u>H</u>$_2$-CH$_2$- and one H of Ar-CH$_2$-C<u>H</u>$_2$-), 2.58 – 2.51$^{\#}$ (m, 1H, one H of Ar-C<u>H</u>$_2$-CH$_2$-);^{13}C NMR (126 MHz, DMSO-d_6, δ [ppm]): 187.01 q (benzoyl C=O), 165.10 q (pyrrolone C-2), 161.68 q (d, J = 244.4 Hz, 4-fluorophenyl C-4), 161.61 q (4-hydroxybenzoyl C-4), 155.69 q (4-hydroxyphenethyl C-4), 150.10* q (pyrrolone C-3), 132.45 q (d, J = 3.8 Hz, 4-fluorophenyl C-1), 131.39 (4-hydroxybenzoyl C-2,6), 129.64 (d, J = 8.3 Hz, 4-fluorophenyl C-2,6), 129.30 (4-hydroxyphenethyl C-2,6), 129.08 q (4-hydroxybenzoyl C-1), 128.47 q (4-hydroxyphenethyl C-1), 119.50 q (pyrrolone C-4), 115.27 (d, J = 21.4 Hz, 4-fluorophenyl C-3,5), 115.10 (4-hydroxyphenethyl C-3,5), 114.58 (4-hydroxybenzoyl C-3,5), 60.21 (pyrrolone C-5), 41.72 (-<u>C</u>H$_2$-CH$_2$-Ar), 32.70 (-CH$_2$-<u>C</u>H$_2$-Ar); LRMS (ESI$^-$) *m/z*: 432 [M-H]$^-$; HRMS (ESI$^+$): *m/z* calcd for $C_{25}H_{21}FNNaO_5^+$: 456.1218 [M+Na]$^+$, found: 456.1221; HPLC retention time 18.08 min, 98% (M2). The attached ^{1}H and ^{13}C NMR spectra contain residual solvent signals (Et_2O, AcOH).

5-(4-Fluorophenyl)-3-hydroxy-4-(3-hydroxybenzoyl)-1-(4-hydroxyphenethyl)-1,5-dihydro-2*H*-pyrrol-2-one (**39**)

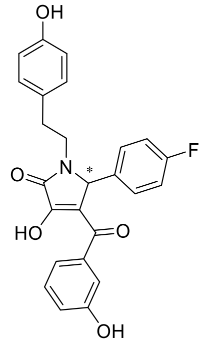

5-(4-Fluorophenyl)-3-hydroxy-1-(4-hydroxyphenethyl)-4-(3-methoxybenzoyl)-1,5-dihydro-2*H*-pyrrol-2-one (**48**, 25.0 mg, 55.9 µmol, 1.0 eq) was dissolved in dry CH_2Cl_2 (1.5 mL). To this solution, boron tribromide (84.0 mg, 335 µmol, 6.0 eq) was added and stirred at room temperature for 4 h. After complete conversion of the starting materials, water (3 mL) was added to quench the reaction and the mixture was stirred for 10 min at room temperature. The mixture was extracted with diethyl ether. The combined organic layer was dried over sodium sulfate and concentrated under reduced pressure. The crude product was purified by preparative HPLC (acetonitrile/water (0.1% TFA): gradient 25-50%) to obtain the title compound as a colorless solid (6 mg, 25%).^{1}H NMR (600 MHz, DMSO-d_6 δ [ppm]): 11.69 (bs, 1H, pyrrolone -OH), 9.61 (bs, 1H, 3-hydroxybenzoyl -OH), 9.23 (s, 1H, 4-hydroxyphenethyl -OH), 7.30 – 7.25 (m, 2H, 4-fluorophenyl H-2,6), 7.21 (t, J = 7.9 Hz, 1H, 3-hydroxybenzoyl H-5), 7.16 – 7.12 (m, 2H, 4-fluorophenyl H-3,5), 7.12 – 7.09 (m, 1H, 3-hydroxybenzoyl H-4 or H-6), 7.03 – 7.00 (m, 1H, 3-hydroxybenzoyl H-2), 6.92 – 6.88 (m, 3H, 4-hydroxyphenethyl H-2,6, 3-hydroxybenzoyl H-4 or H-6), 6.68 – 6.64 (m, 2H, 4-hydroxyphenethyl H-3,5), 5.29 (s, 1H, pyrrolone H-5), 3.78 – 3.71 (m, 1H, one H of Ar-CH$_2$-C<u>H</u>$_2$-), 2.79 – 2.67 (m, 2H, one H of Ar-C<u>H</u>$_2$-CH$_2$- and one H of Ar-CH$_2$-C<u>H</u>$_2$-), 2.59 – 2.51 (m, 1H, one H of Ar-C<u>H</u>$_2$-

CH_2);^{13}C NMR[a] (DEPTQ, 151 MHz, DMSO-d_6 δ [ppm]): 161.81 q (d, J = 244.5 Hz, 4-fluorophenyl C-4), 157.01 q (3-hydroxybenzoyl C-3), 155.83 q (4-hydroxyphenethyl C-4), 129.70 (d, J = 8.3 Hz, 4-fluorophenethyl C-2,6), 129.44 (4-hydroxyphenethyl C-2,6), 129.07 (3-hydroxybenzoyl C-6), 128.56 q (4-hydroxyphenethyl C-1), 119.57 (3-hydroxybenzoyl C-4), 118.41 q (pyrrolone C-4), 115.43 (d, J = 21.3 Hz, 4-fluorophenyl C-3,5), 115.23 (4-hydroxyphenethyl C-3,5), 115.00 (3-hydroxybenzoyl C-2), 60.17 (pyrrolone C-5), 41.89 (Ar-CH_2-$\underline{C}H_2$-), 32.77 (Ar-$\underline{C}H_2$-CH_2-).The attached ^1H and ^{13}C NMR spectra contain residual solvent signals (MeOH). LRMS (ESI^+) *m/z*: 434 $[M+H]^+$; HRMS (ESI^+): *m/z* calcd for $C_{25}H_{21}FNO_5^+$: 434.1398 $[M+H]^+$, found: 434.1400; HPLC retention time 15.64 min, 98% (M1).

[a] the ^{13}C NMR signals for the carbons of 3-hydroxybenzoyl C=O, pyrrolone C-2,3, 4-fluorophenyl C-1, and 3-hydroxybenzoyl C-1,5 could not be detected.

4-Benzoyl-1-(4-bromo-2-fluorophenyl)-5-(4-fluorophenyl)-3-hydroxy-1,5-dihydro-2H-pyrrol-2-one (**40**)

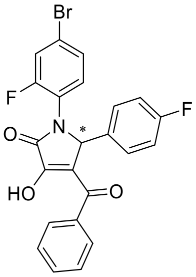

4-Bromo-2-fluoroaniline (43.1 mg, 227 µmol, 1.0 eq), ethyl 2,4-dioxo-4-phenylbutanoate (**44**, 50.0 mg, 227 µmol, 1.0 eq), and 4-fluorobenzaldehyde (28.2 mg, 227 µmol, 1.0 eq) were placed in a microwave tube and dissolved in acetic acid (1.0 mL). The reaction mixture was heated to 80 °C and stirred for 16h. After complete conversion of the starting materials monitored by LC-MS, the colorless precipitate formed was separated by filtration and washed with Et_2O. This step was performed several times to obtain the title compound as a colorless solid (21 mg, 20%). ^1H NMR (400 MHz, DMSO-d_6 δ [ppm]): 12.11 (bs, 1H, pyrrolone -OH), 7.80 – 7.73 (m, 2H, benzoyl H-2,6), 7.65 – 7.53 (m, 3H, 4-bromo-2-fluorophenyl H-3,5,6), 7.50 – 7.42 (m, 3H, benzoyl H-3,4,5), 7.41 – 7.34 (m, 2H, 4-fluorophenyl H-2,6), 7.07 – 6.99 (m, 2H, 4-fluorophenyl H-3,5), 6.17 (s, 1H, pyrrolone H-5); ^{13}C NMR (DEPTQ, 101 MHz, DMSO-d_6 δ [ppm]): 189.30 q (benzoyl C=O), 164.13 q (pyrrolone C-2), 161.84 q (d, J = 244.7 Hz, 4-fluorophenyl C-4), 156.65 q (d, J = 255.4 Hz, 4-bromo-2-fluorophenyl C-2), 150.15 q (pyrrolone C-3), 137.75 q (benzoyl C-1), 132.86 (benzoyl C-4), 131.86 q (d, J = 2.7 Hz,4-fluorophenyl C-1), 129.87 (4-bromo-2-fluorophenyl C-5), 129.83 (d, J = 8.5 Hz, 4-fluorophenyl C-2,6), 128.87 (benzoyl C-2,6), 128.28 (benzoyl C-3,5), 128.05 (d, J = 3.4 Hz, 4-bromo-2-fluorophenyl C-6), 123.04 q (d, J = 12.3 Hz, 4-bromo-2-fluorophenyl C-1), 120.64 q (pyrrolone C-4), 120.52 q (d, J = 9.1 Hz, 4-bromo-2-fluorophenyl C-4), 119.85 (d, J = 23.2 Hz, 4-bromo-2-fluorophenyl C-3), 115.41 (d, J = 21.6 Hz, 4-fluorophenyl C-3,5), 61.78 (pyrrolone C-5); LRMS (ESI^+) *m/z*: 472 $[M(^{81}Br)+H]^+$; HRMS (ESI^+): *m/z* calcd for $C_{23}H_{15}BrF_2NO_3^+$: 470.0198 $[M(^{79}Br)+H]^+$, found: 470.0199; HPLC retention time: 18.77 min, 99% (M1).

Ethyl 2,4-dioxo-4-phenylbutanoate (**44**)[5]

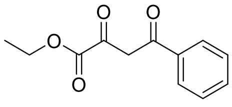

To a NaH suspension (60% w/w dispersion in mineral oil, 1.33 g, 33.3 mmol, 4.0 eq) together with dry THF at 0°C was added diethyl oxalate (3.65 g, 25.0 mmol, 3.0 eq) under virguous stirring. After 15 minutes stirring at room temperature, acetophenone (1.0 g, 8.32 mmol, 1.0 eq) was added and the resulting mixture was refluxed for 1 h. The reaction was cooled down to room temperature and acidified the mixture with 2N HCl. The precipitate was filtered, washed with water and further purified by Biotage flash chromatography (EtOAc/isohexane, gradient 5–30%) to obtain the title compound as a pale-yellow oil (550 mg, 30%). R_F = 0.25 (EtOA/isohexane, 2:8); ^1H NMR (400 MHz, DMSO-d_6 δ [ppm]): 14.51 (bs, 1H, enol -O$\underline{H}$ of -COC$\underline{H}_2$CO-), 8.10 – 8.05 (m, 2H, phenyl H-2,6), 7.75 – 7.68 (m, 1H, phenyl H-4), 7.62 – 7.55 (m, 2H, phenyl H-3,5), 7.13 (s, 1H, -COC$\underline{H}_2$CO-), 4.32 (q, J = 7.1 Hz, 2H, -C$\underline{H}_2$-CH_3), 1.31 (t, J = 7.1 Hz, 3H, -CH_2-C$\underline{H}_3$); LRMS (ESI^+) *m/z*: 221 $[M+H]^+$. Dissolved in DMSO, the title compound predominantly exists in the enol form. Thus, only one proton was observed for -COC$\underline{H}_2$CO-. The obtained analytical data are very good agreement with literature values.[5]

Ethyl 4-(3-methoxyphenyl)-2,4-dioxobutanoate (**45**)[6]

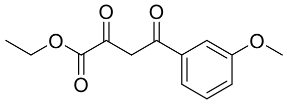

To 20% sodium ethanolate solution 1 M in ethanol (1.29 mL,16.65 mmol, 5.0 eq) was added 1-(3-methoxyphenyl)ethan-1-one (500 mg, 3.33 mmol, 1.0 eq) under nitrogen atmosphere. After 10 min stirring at room temperature, diethyl oxalate (729 mg, 3.33 mmol, 1.0 eq.) in abs. ethanol (3 mL) was added and stirred for 15 h at room temperature. After complete conversion of starting materials, the precipitated was filtrated off under vacuum and crude product was dissolve din water (200 mL). 2 M HCl was added to adjust pH 3-4. The formed precipitates were separated by filtration and dried under reduced pressure. The title compound was obtained as a white powder (0.492 g, 1.97 mmol, 59 %). ^{1}H NMR (400 MHz, DMSO-d_6 δ [ppm]): 14.62 (bs, 1H, enol -O$\underline{H}$ of -COC$\underline{H}_2$CO-), 7.70 – 7.64 (m, 1H, 3-methoxyphenyl H-6), 7.55 – 7.46 (m, 2H, 3-methoxyphenyl H-2,5), 7.31 – 7.26 (m, 1H, 3-methoxyphenyl H-4), 7.11 (s, 1H, -CO-C$\underline{H}_2$-CO-), 4.32 (q, J = 7.1 Hz, 2H, -O-C$\underline{H}_2$-CH$_3$), 3.85 (s, 3H, -OC$\underline{H}_3$), 1.32 (t, J = 7.1 Hz, 3H, -O-CH$_2$-C$\underline{H}_3$); ^{13}C NMR (101 MHz, DMSO-d_6 δ [ppm]): 189.95 q (H$_3$C-CH$_2$-O-CO-$\underline{C}$O-), 169.01 q (-CH$_2$-$\underline{C}$O-Ar), 161.54 q (H$_3$C-CH$_2$-O-$\underline{C}$O-CO-), 159.63 q (3-methoxyphenyl C-3), 135.81 q (3-methoxyphenyl C-1), 130.29 (3-hydroxyphenyl C-5), 120.41 (3-methoxyphenyl C-6 or C-4), 120.22 (3-methoxyphenyl C-4 or C-6), 112.19 (3-methoxyphenyl C-2), 98.19 (-CO-$\underline{C}$H$_2$-CO-), 62.18 (-O-$\underline{C}$H$_2$-CH$_3$), 55.42 (-O$\underline{C}$H$_3$), 13.82 (-O-CH$_2$-$\underline{C}$H$_3$); LRMS (APCI$^-$) *m/z*: 249 [M-H]$^-$. Dissolved in DMSO, the title compound predominantly exists in the enol form. Thus, only one proton was observed for -COC$\underline{H}_2$CO-. The obtained analytical data are in good agreement with literature values.[6]

Ethyl 4-(4-hydroxyphenyl)-2,4-dioxobutanoate (**46**)[7]

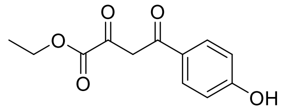

To 20% sodium ethanolate solution 1 M in ethanol (6.25 g, 18.4 mmol, 5.0 eq) was added 4-hydroxyacetophenone (500 mg, 3.67 mmol, 1.0 eq) under nitrogen atmosphere. After 10 min stirring at room temperature, diethyl oxalate (1.61 g, 11.0 mmol, 3.0 eq) was added and stirred for 2 days at room temperature. After complete conversion of starting materials, 2 M HCl was added to adjust pH 3-4. The formed precipitates were separated by filtration and dried under reduced pressure. The title compound was obtained as a yellow powder (508 mg, 2.15 mmol, 59%). ^{1}H NMR (500 MHz, DMSO-d_6 δ [ppm]): 15.46 (bs, 1H, enol -O$\underline{H}$ of -COC$\underline{H}_2$CO-), 10.69 (s, 1H, -OH), 8.01 – 7.96 (m, 2H, 4-hydroxyphenyl H-2,6), 7.04 (s, 1H, -CO-C$\underline{H}_2$-CO-), 6.94 – 6.89 (m, 2H, phenyl H-3,5), 4.31 (q, J = 7.1 Hz, 2H, -C$\underline{H}_2$-CH$_3$), 1.31 (t, J = 7.1 Hz, 3H, -CH$_2$-C$\underline{H}_3$), ^{13}C NMR (126 MHz, DMSO-d_6 δ [ppm]): 190.06 q (H$_3$C-CH$_2$-O-CO-$\underline{C}$O-), 166.97 q (-CH$_2$-$\underline{C}$O-Ar), 163.32 q (4-hydroxyphenyl C-4), 161.70 q (H$_3$C-CH$_2$-O-$\underline{C}$O-CO-), 130.74 (4-hydroxyphenyl C-2,6), 125.38 q (-hydroxyphenyl C-1), 115.80 (4-hydroxyphenyl C-3,5), 97.44 (-CO-$\underline{C}$H$_2$-CO-), 61.94 (H$_3$C-$\underline{C}$H$_2$-O-), 13.76 (H$_3\underline{C}$-CH$_2$-O-); LRMS (APCI$^-$) *m/z*: 235 [M-H]$^-$. Dissolved in DMSO, the title compound predominantly exists in the enol form. Thus, only one proton was observed for -COC$\underline{H}_2$CO-. The obtained analytical data are in good agreement with literature values.[7]

5-(4-Fluorophenyl)-3-hydroxy-1-(4-hydroxyphenethyl)-4-(3-methoxybenzoyl)-1,5-dihydro-2*H*-pyrrol-2-one (**48**)

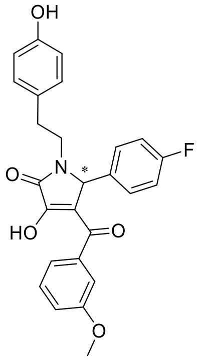

4-Fluorobenzaldehyde (99.2 mg, 799 µmol, 1.0 eq), ethyl 4-(3-methoxyphenyl)-2,4-dioxobutanoate (**45**, 200.0 mg, 799 µmol, 1.0 eq), and 4-(2-aminoethyl)phenol (109.6 mg, 799 µmol, 1.0 eq) were placed in a microwave tube and dissolved in acetic acid (2.0 mL). The reaction mixture was heated to 90 °C and stirred for 20 h. After complete conversion of the starting materials, the reaction mixture was cooled down to room temperature and Et_2O was added. The brown precipitate was separated by filtration and washed with Et_2O to obtain the title compound as a brown solid (102.5 mg, 229.1 µmol, 29%). ^{1}H NMR (500 MHz, DMSO-d_6 δ [ppm]): 11.58 (bs, 1H, pyrrolone -OH), 9.21 (s, 1H, 4-hydroxyphenethyl -OH), 7.37 – 7.24 (m, 4H, 4-fluorophenyl H-2,6, 3-methoxybenzoyl H-5,6), 7.20 – 7.08 (m, 4H, 4-fluorophenyl H-3,5, 3-methoxybenzoyl H-2,4), 6.94 -6.88 (m, 2H, 4-hydroxyphenethyl H-2,6), 6.70 – 6.64 (m, 2H, 4-hydroxyphenethyl H-3,5), 5.30 (s, 1H, pyrrolone H-5), 3.79 – 3.70 (m, 4 H, one H of -C$\underline{H}_2$-CH$_2$-Ar, -C$\underline{H}_3$), 2.81 – 2.67 (m, 2H, one H of -C$\underline{H}_2$-CH$_2$-Ar, one H of -CH$_2$-C$\underline{H}_2$-Ar), 2.58 – 2.52$^\#$ (m, 1H, one H of -CH$_2$-C$\underline{H}_2$-Ar); ^{13}C NMR (DEPTQ, 126 MHz, DMSO-d_6, δ [ppm]): 188.35* q (3-methoxybenzoyl C=O), 164.98 q (pyrrolone C-2), 161.82 q (d, *J* = 244.2 Hz, 4-fluorophenyl C-4), 158.84 q (3-methoxybenzoyl C-3), 155.81 q (4-hydroxyphenethyl C-4), 151.89* q (pyrrolone C-3), 139.42 q (3-methoxybenzoyl C-1), 132.57 q (4-fluorophenyl C-1), 129.80 (d, *J* = 8.4 Hz, 4-fluorophenyl C-2,6), 129.42 (4-hydroxyphenethy C-2,6), 129.18 (3-methoxybenzoyl C-5), 128.55 q (4-hydroxyphenethyl C-1), 121.09 (3-methoxybenzoyl C-6), 118.74 q (pyrrolone C-4), 118.13 (3-methoxybenzoyl C-4), 115.40 (d, *J* = 21.5 Hz, 4-fluorophenyl C-2,6), 115.22 (4-hydroxyphenethyl C-3,5), 113.55 (3-methoxybenzoyl C-2), 60.20 (pyrrolone C-5), 55.22 (-$\underline{C}$H$_3$), 41.90 (-$\underline{C}$H$_2$-CH$_2$-Ar), 32.76 (-CH$_2$-$\underline{C}$H$_2$-Ar); LRMS (APCI$^-$) *m/z*: 446 [M-H]$^-$. The attached ^{1}H and ^{13}C NMR spectra contain residual solvent signals (Et_2O).

4-Benzoyl-5-(4-fluorophenyl)-3-hydroxy-1-(4-methoxyphenethyl)-1,5-dihydro-2*H*-pyrrol-2-one (**49**)

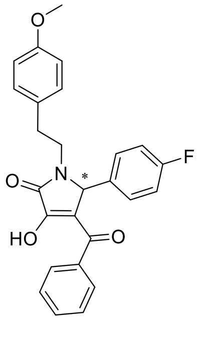

4-Fluorobenzaldehyde (164 mg, 1.32 mmol, 1.0 eq), ethyl 2,4-dioxo-4-phenylbutanoate (**44**, 200 mg, 1.32 mmol, 1.0 eq), and 2-(4-methoxyphenyl)ethan-1-amine (291 mg, 1.32 mmol, 1.0 eq) were placed in a microwave tube and dissolved in acetic acid (1.0 mL). The reaction mixture was heated to 90 °C and stirred for 18 h. After complete conversion of the starting materials monitored by LC-MS, the colorless precipitate was separated by filtration and washed with Et_2O to obtain the title compound as a colorless solid (276 mg, 49%). ^{1}H NMR (400 MHz, DMSO-d_6 δ [ppm]): 11.81 (s, 1H, pyrrolone -OH), 7.69 – 7.64 (m, 2H, benzoyl H-2,6), 7.58 – 7.52 (m, 1H, benzoyl H-4), 7.46 – 7.40 (m, 2H, benzoyl H-3,5), 7.38 – 7.32 (m, 2H, 4-fluorophenyl H-2,6), 7.18 – 7.11 (m, 2H, 4-fluorophenyl H-3,5), 7.07 – 7.02 (m, 2H, 4-methoxyphenethyl H-2,6), 6.86 – 6.81 (m, 2H, 4-methoxyphenethyl H-3,5), 5.37 (s, 1H, pyrrolone H-5), 3.83 – 3.74 (m, 1H, one H of Ar-CH$_2$-C$\underline{H}_2$-), 3.71 (s, 3H, -O-C$\underline{H}_3$), 2.83 – 2.72 (m, 2H, one H of Ar-C$\underline{H}_2$-CH$_2$- and one H of Ar-CH$_2$-C$\underline{H}_2$-), 2.68 – 2.57 (m, 1H, one H of Ar-C$\underline{H}_2$-CH$_2$-); ^{13}C NMR (DEPTQ, 101 MHz, DMSO-d_6, δ [ppm]): 189.03 q (benzoyl C=O), 164.84 q (pyrrolone C-2), 161.90 q (d, *J* = 244.3 Hz, 4-fluorophenyl C-4), 157.84 q (4-methoxyphenethyl C-4), 151.45 q (pyrrolone C-3), 138.03 q (benzoyl C-1), 132.53 (benzoyl C-4), 132.34 q (d, *J* = 2.8 Hz, 4-fluorophenyl C-1), 130.38 q (4-methoxyphenethyl C-1), 129.89 (d, *J* = 8.3 Hz, 4-fluorophenyl C-2,6), 129.58 (4-methoxyphenethyl C-2,6), 128.70 (benzoyl C-2,6), 128.13 (benzoyl C-3,5), 119.11 q (pyrrolone C-4), 115.50 (d, *J* = 21.5 Hz, 4-fluorophenyl C-3,5), 113.86 (4-methoxyphenethyl C-3,5), 60.08

(pyrrolone C-5), 54.99 (-O-C̲H3), 41.80 (-C̲H2-CH2-Ar), 32.68 (-CH2-C̲H2-Ar); HRMS (ESI$^+$): *m/z* calcd for $C_{26}H_{23}FNO_4^+$: 432.1601 [M+H]$^+$, found: 432.1607 [M+H]$^+$; HPLC retention time: 20.26 min, 97% (M2).

Methyl (*R*)-2-(((*R*)-4-benzoyl-5-(4-fluorophenyl)-1-(4-methoxyphenethyl)-2-oxo-2,5-dihydro-1*H*-pyrrol-3-yl)oxy)-2-phenylacetate and methyl (*R*)-2-(((*R*)-4-benzoyl-5-(4-fluorophenyl)-1-(4-methoxyphenethyl)-2-oxo-2,5-dihydro-1*H*-pyrrol-3-yl)oxy)-2-phenylacetate (**50a**-**b**)

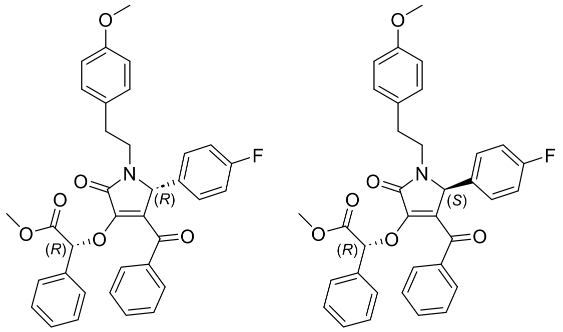

To an ice-cooled mixture of 4-benzoyl-5-(4-fluorophenyl)-3-hydroxy-1-(4-methoxyphenethyl)-1,5-dihydro-2*H*-pyrrol-2-one (**49**, 271 mg, 628 µmol, 1.0 eq), methyl (*S*)-mandelate (146 mg, 879 µmol, 1.4 eq), and triphenylphosphine (213 mg, 813 µmol, 1.3 eq) in anhydrous THF (2 mL) is added dropwise DIAD (165 mg, 813 µmol, 1.3 eq). Then, the reaction was allowed to warm to room temperature and was stirred for further 12 hours. Subsequently, the solvent was removed under reduced pressure and the yellow oily residue was purified by column chromatography (eluent: 83/17 hexanes/ethyl acetate), in order to separate the *R*,*R*- and the *R*,*S*-diastereomers from each other.

50a is the diastereomer that was eluted first from the column and was obtained as a light brown solid (71 mg, 20%). ^{1}H NMR (400 MHz, DMSO-d_6 δ [ppm]): 7.85 – 7.79 (m, 2H, benzoyl H-2,6), 7.65 – 7.59 (m, 1H, benzoyl H-4), 7.53 – 7.46 (m, 2H, benzoyl H-3,5), 7.36 – 7.18 (m, 7H, 4-fluorophenyl H-2,4,5,6, phenyl H-3,4,5), 7.09 – 7.01 (m, 4H, 4-methoxyphenethyl H-2,6, phenyl H-2,6), 6.88 – 6.83 (m, 2H, 4-methoxyphenethyl H-3,5), 6.79 (s, 1H, H3C-O-CO-CH̲(OR)-Ph), 5.41 (s, 1H, pyrrolone H-5), 3.80 – 3.74 (m, 1H, one H of Ar-CH2-CH̲2-), 3.72 (s, 3H, Ar-O-CH̲3), 3.71 (s, 3H, H̲3C-O-CO-), 2.82 – 2.73 (m, 2H, one H of Ar-CH̲2-CH2- and one H of Ar-CH2-CH̲2-), 2.68 – 2.61 (m, 1H, one H of Ar-CH̲2-CH2-); ^{13}C NMR (DEPTQ, 101 MHz, DMSO-d_6, δ [ppm]): 189.12 q (benzoyl C=O), 169.92 q (H3C-O-C̲O-CH(OR)-Ph), 163.94 q (pyrrolone C-2), 162.09 q (d, *J* = 251.5 Hz, 4-fluorophenyl C-4), 157.91 q (4-methoxyphenethyl C-4), 148.33 q (pyrrolone C-3), 137.33 q (benzoyl C-1), 134.35 q (phenyl C-1), 133.11 (benzoyl C-4), 131.09 q (d, *J* = 2.9 Hz, 4-fluorophenyl C-1), 130.27 q (4-methoxyphenethyl C-1), 129.63 (d, *J* = 8.1 Hz, 4-fluorophenyl C-2,6), 129.59 (4-methoxyphenethyl C-2,6), 129.16 (phenyl C-4), 128.90 (benzoyl C-2,6), 128.48 (benzoyl C-3,5), 128.45 (phenyl C-3,5), 127.44 (phenyl C-2,6), 125.18 q (pyrrolone C-4), 115.93 (d, *J* = 21.7 Hz, 4-fluorophenyl C-3,5), 113.93 (4-methoxyphenethyl C-3,5), 78.25 (H3C-O-CO-C̲H(OR)-Ph), 60.16 (pyrrolone C-5), 55.03 (Ar-O-C̲H3), 52.75 (H3C̲-O-CO-), 41.95 (-C̲H2-CH2-Ar), 32.59 (-CH2-C̲H2-Ar); LRMS (ESI+) *m/z*: 580 [M+H]$^+$; HPLC retention time: 39.80 min, 97% (M3). The attached ^{1}H and ^{13}C NMR spectra of **50** contain signals for the side product diisopropyl hydrazine-1,2-dicarboxylate.

50b is the diastereomer that was eluted second from the column and was obtained as a light brown solid (64 mg, 18%). ^{1}H NMR (400 MHz, DMSO-d_6 δ [ppm]): 7.63 – 7.60 (m, 2H, benzoyl H-2,6), 7.46 – 7.39 (m, 3H, 4-fluorophenyl H-2,6, benzoyl H-4), 7.34 – 7.29 (m, 1H, phenyl H-4), 7.26 – 7.14 (m, 6H, phenyl H-3,5, benzoyl H-3,5, 4-fluorophenyl H-3,5), 7.07 – 7.01 (m, 4H, 4-methoxyphenethyl H-2,6, phenyl H-2,6), 6.88 – 6.83 (m, 2H, 4-methoxyphenethyl H-3,5), 6.69 (s, 1H, H3C-O-CO-CH̲(OR)-Ph), 5.40 (s, 1H, pyrrolone H-5), 3.76 – 3.68 (m, 4H, one H of Ar-CH2-CH̲2-, Ar-O-CH̲3), 3.59 (s, 3H, H̲3C-O-CO-), 2.83 – 2.73 (m, 2H, one H of Ar-CH̲2-CH2- and one H of Ar-CH2-CH̲2-), 2.62 – 2.53 (m, 1H, one H of Ar-CH̲2-CH2-); ^{13}C NMR (DEPTQ, 101 MHz, DMSO-d_6, δ [ppm]): 189.29 q (benzoyl C=O), 169.36 q (H3C-O-C̲O-CH(OR)-Ph), 163.76 q (pyrrolone C-2), 162.09 q (d, *J* = 244.8 Hz, 4-fluorophenyl C-4), 157.89 q (4-methoxyphenethyl C-4), 148.96 q (pyrrolone C-3), 137.26 q (benzoyl C-1), 134.50 q (phenyl C-1), 132.70 (benzoyl C-4), 131.25 q (d, *J* = 2.9 Hz, 4-fluorophenyl C-1), 130.27 q (4-methoxyphenethyl C-1), 130.18 (d, *J* = 8.3 Hz, 4-fluorophenyl C-2,6), 129.55 (4-methoxyphenethyl C-2,6), 128.89 (phenyl C-4), 128.73 (benzoyl C-2,6), 128.41 (phenyl C-3,5), 128.04 (benzoyl C-3,5), 127.09 (phenyl C-2,6), 125.65 q (pyrrolone C-4), 115.61 (d, *J* = 21.6 Hz, 4-fluorophenyl C-3,5), 113.94 (4-methoxyphenethyl C-3,5), 78.25 (H3C-O-CO-C̲H(OR)-Ph), 60.23 (pyrrolone C-5), 55.02 (Ar-O-C̲H3), 52.27 (H3C̲-O-CO-), 41.93 (-C̲H2-CH2-Ar), 32.58 (-CH2-C̲H2-Ar); LRMS (ESI+) *m/z*: 580 [M+H]$^+$; HPLC retention time: 39.19 min, 99% (M3). The attached ^{1}H and ^{13}C NMR spectra of **50b** contain signals for residual triphenylphosphine oxide.

Biological tests

Cell culture: HEK293T cells were cultured as previously described.[8] In brief, HEK293T cells (gift from Chair of Physiology, Prof. Dr. Alzheimer, FAU Erlangen-Nürnberg) were grown on 10 cm culture dishes at 37 °C and 5% CO_2. As growth medium, Dulbecco's Modified Eagle Medium (DMEM)/F12 (Invitrogen) supplemented with 10% fetal bovine serum (FBS, Gibco™ Fetal Bovine Serum, qualified, Brazil), L-glutamine (final concentration: 2 mM; from Gibco™ L-glutamine 200 mM, 100X), penicillin (final concentration: 100 units/mL), and streptomycin (final concentration: 100 µg/mL; from Gibco™ Penicillin-Streptomycin 10,000 U/mL) was used. Cells were split every three to four days and regularly confirmed to be free of mycoplasma contamination using the luminescence-based MycoAlert Plus Kit (Lonza).

Transient transfection using polyethylenimine: For transfection, we used a previously published procedure.[8] In brief, HEK293T cells were plated onto culture dishes (Ø 10 cm or Ø 15 cm) and grown to a confluence of approximately 50% at 37 °C and 5% CO_2. The growth medium was renewed one-hour before transfection. The transfection mix was prepared, as described in the following. Solution A (0.4-2.2% total DNA (1-5.5 µg) in Gibco™ phosphate buffered saline, pH 7.4) and solution B (3% PEI (linear, 25 kDa, from Polysciences) solution prepared from a PEI stock solution (0.4 µg/µL) in PBS without $MgCl_2$ and $CaCl_2$) were mixed one to one, the resulting mixture was vortexed for 5 s and incubated for 30 min at room temperature. The pre-incubated transfection mix was added dropwise to the cells and cell cultivation was continued at 37 °C and 5% CO_2.

Membrane preparation: Membranes were prepared as previously reported.[8] In brief, membranes from HEK293T cells transiently expressing the respective GPCR were prepared as follows. The medium of the transfected cells was refreshed after 24 h before cells were harvested 48 h post-transfection. The growth medium was removed, the cells were carefully washed with cold phosphate buffered saline (10 mL per Ø 15 cm dish). The cells were detached with 15 mL of ice-cold Tris-EDTA buffer (10 mM Tris, 0.5 mM EDTA, 5.4 mM KCl, 140 mM NaCl, pH 7.4) and subsequently centrifuged with 218 *g* for 8 minutes. The supernatant was removed, and the cells were resuspended in 10 mL Tris-EDTA buffer. The cells were lysed with an Ultraturrax (20,000 rpm) used five times for 5 seconds with a 25-second break on ice in between. The lysate was centrifuged at 50,830 *g* for 18 min at 4 °C. The supernatant was discarded, and the pellet was homogenized in membrane buffer (50 mM Tris, 1 mM EDTA, 5 mM $MgCl_2$, 100 µg/mL bacitracin, 5 µg/mL soybean trypsin inhibitor, pH 7.4) with a glass-teflon homogenizer. Aliquots of 250 µL were shock frozen in liquid nitrogen and directly stored at -80 °C. Finally, the protein concentration was determined using the Lowry method.[9]

cDNA constructs: The CCR1-Nluc fusion construct (CCR1_Nluc) in pcDNA3.1 was generated as previously reported[1] using the Gibson Assembly[10] (New England Biolabs) method. Therefore, the sequences of the Nluc enzyme[11] (pNLF1-C, Promega) and (3xHA)-tagged CCR1 (CCR1, cdna.org, #CCR010TN00) were amplified by polymerase chain reaction and were directly fused in frame (no linker). DNA sequencing was performed to verify sequence integrity (Eurofins Genomics). Plasmids were cloned into *E.coli* DH5-alpha (New England Biolabs) and purified using a Maxiprep DNA purification kit (Invitrogen). For selectivity studies with CCR2, CCR9, CXCR1, and CXCR2, we modified the procedure described above. Instead of (3xHA)-tagged CCR1 (cdna.org, #CCR010TN00), we used (3xHA)-tagged CCR2 (cdna.org, #CCR020TN00), (3xHA)-tagged CCR9 (cdna.org, #CCR090TN00), (3xHA)-tagged CXCR1 (cdna.org, #CXCR10TN00), and (3xHA)-tagged CXCR2 (cdna.org, #CXCR20TN00), respectively. For CCR2, we inserted a GSSG linker between the C-terminus of the respective receptor and the Nluc-tag, whereas for CXCR1, CXCR2, and CCR9 no linker was used. The constructs for the Nluc-labeled CCR2, CCR9, CXCR1, and CXCR2 were already published.[1, 3, 8]

ELISA: ELISA-based experiments were performed as previously reported.[1, 3, 8] For confirmation of CCR1 expression, HEK293T cells were transfected with the plasmid encoding 3xHA-CCR1 or with the 3xHA-tagged CCR1_Nluc construct using polyethylenimine in suspension. Therefore, HEK293T cells were detached from their culture plates and diluted to a density of 3 x 10^5 cells/mL in growth medium. This cell suspension was mixed with the preformed transfection mix (PEI/DNA ratio 2.5:1) consisting of 1.2 µg of receptor cDNA plasmid and 1.2 µg of single stranded salmon sperm DNA (ssDNA, Sigma Aldrich) in phosphate buffered saline (PBS) per 2.4 mL of cell suspension. Subsequently, cells were transferred to a 48-well plate (7.5 x 10^4 cells/well), which was pretreated with poly-D-lysine (0.1 mg/mL, dissolved in water). Cells were incubated for 48 h at 37 °C and 5% CO_2. On the day of the assay, the medium was removed, and cells were incubated with 200 µL/well of ROTI®Histofix 4% fixation solution (Carl Roth) for 10 min at room temperature. Cells were washed once with 300 µL washing buffer for two minutes (150 mM NaCl, 25 mM Tris, pH 7.5) and blocked for one hour using 800 µL blocking buffer (30 g/L skim milk powder in washing buffer). After removal of the blocking solution, 200 µL/well of anti-HA rabbit IgG antibody (Sigma Aldrich, catalog # H6908, 1:4000 in blocking solution) were added. After 60 min of incubation, wells were washed twice for two minutes (300 µL/well) and blocked again for one hour at room temperature, before 200 µL/well anti-rabbit IgG-HRP antibody (Invitrogen by Thermo Fisher Scientific, catalog # G-21234, 1:1000 in blocking solution) was added. After incubation for one hour, cells were washed three times for two minutes (300 µL/well), before the substrate reaction was initiated by the addition of substrate buffer (6 mM o-phenylenediamine in 35 mM citric acid, 66 mM Na_2HPO_4, pH 5.0). After 15 minutes incubation in the dark, the reactions were terminated by addition of 1 M H_2SO_4 (200 µL/well). For each well, 2 x 150 µL of the resulting mixture were transferred to a clear, flat bottom 96-well plate and absorption was measured at 492 nm in a microplate reader. The measured absorbance values were baseline-

corrected using cells transfected with a non-tagged muscarinic receptor (M3R, cdna.org) as negative control. These baseline-corrected values were normalized to 3xHA-CCR1 expression.

Emission and excitation spectra of the fluorescent ligands: For the detection of the emission and excitation spectra of the fluorescent ligands, we used referred to a previously published procedure.[8] For the excitation spectra, the fluorescent ligands (**11-12**) were diluted to 500 µM in aqueous solution and 25 µL of these solutions were pipetted into a 384-well plate. Then, excitation spectra were measured with a CLARIOstar microplate reader. The emission spectra of the fluorescent ligands (1 mM in DMSO) were recorded using a CLARIOstar (BMG Labtech, Ortenberg, Germany) microplate reader and 480 nm as excitation wavelength.

Emission spectra of Nluc-labeled CCR1 (CCR1_Nluc) protein: The emission spectra of CCR1_Nluc has already been reported by Toy *et al*.[1] For a confirmation of the reported data with new CCR1_Nluc membrane batches, furimazine (Promega, Mannheim, Germany 1:2000) was added to the membrane preparations (2 µg protein/well). After 5 minutes incubation in the dark, the emission spectra were measured ranging from 350 to 700 nm using a CLARIOstar (BMG Labtech, Ortenberg, Germany) microplate reader.

NanoBRET binding assays: *Membrane-based NanoBRET saturation assay:* For the establishment of our NanoBRET binding assay, we referred to recently published protocols.[8, 12] The fluorescent ligands (**8**-**9** and **11**-**12**) were dissolved in DMSO (1 mM) and further diluted to varying concentrations in assay buffer (50 mM Na_2HPO_4, 50 mM KH_2PO_4, pH 7.4, 1 mg/mL saponin, 5% FBS) and 5 µL of these dilutions were pipetted to a 384-well plate. To determine total binding, 5 µL of assay buffer were added to the corresponding wells, while 5 µL of a solution of the reported non-fluorescent intracellular CCR1 inhibitor (**4** or **5**,[13] final assay concentration: 10 µM) in assay buffer were used to determine non-specific binding. Then, 20 µL of the membrane preparations diluted in assay buffer (3 µg total protein/well) were added and the plates were incubated for 90 min at 37 °C. Subsequently, 5 µL of a furimazine solution (Promega, Mannheim, Germany, final assay dilution: 1:5,000) were added to each well (final assay volume: 35 µL) before measuring luminescence with a CLARIOstar microplate reader using 620/10 nm and 475/30 nm emission filters after 5 min of incubation in the dark. Bioluminescence resonance energy transfer (BRET) was determined as the ratio of acceptor fluorescence and donor luminescence. The algorithms for one-site saturation binding from PRISM10.2.1 (GraphPad, USA) were utilized to analyze total, non-specific and specific binding. Specific binding signals were calculated as a difference of total and non-specific binding. If required, netBRET values were calculated as the difference between total BRET values and the values obtained in the absence of a fluorescent ligand. To determine the effect of **3** on the binding of the fluorescent ligand **12**, 5 µL of a solution of **3** (final assay concentration: 10 µM) or buffer were pipetted to a 384-well plate. Subsequently, 20 µL of the membrane preparations diluted in assay buffer (3 µg total protein/well) were added. After incubation for 90 minutes at 37 °C, 5 µL of varying dilutions of the fluorescent ligand **12** dissolved in assay buffer, 5 µL of buffer, or a solution of **5** (final assay concentration: 10 µM) were added. The plates were again then incubated for 90 minutes at 37 °C. Subsequently, 5 µL of a furimazine solution (Promega, Mannheim, Germany, final assay dilution: 1:5,000) were added to each well (final assay volume: 40 µL) before luminescence was measured. For selectivity studies with CCR2, CCR9, CXCR1, and CXCR2, we modified the procedure described above. For CCR2, we used membranes preparations from HEK293T cells expressing the published CCR2_GSSG_Nluc construct[1] (4 µg total protein/well) and CCR2-RA (final assay concentration: 10 µM) as a known intracellular CCR2 antagonist[13] to determine non-specific binding. For CCR9, we used membranes preparations from HEK293T cells expressing the published CCR9_Nluc construct[3] (2 µg total protein/well) and vercirnon (**21**, final assay concentration: 10 µM) as a known intracellular CCR9 antagonist[14, 15] to determine non-specific binding. For CXCR1, we used membranes preparations from HEK293T cells expressing the aforementioned CXCR1_Nluc construct[8] (4 µg total protein/well) and navarixin (**20**, final assay concentration: 10 µM) as a known intracellular CXCR1 antagonist[16] to determine non-specific binding. For CXCR2, we used membranes preparations from HEK293T cells expressing the aforementioned CXCR2_Nluc construct[8] (2 µg total protein/well) and cmpd24 (final assay concentration: 1 µM) as a known intracellular CXCR2 antagonist[16] to determine non-specific binding.

Membrane-based NanoBRET competition assay: The fluorescent ligand **12** was dissolved in assay buffer and 5 µL of this solution (final assay concentration: 2000 nM) were pipetted to a 384-well plate, followed by the addition of 5 µL of varying dilutions of the competing ligand dissolved in assay buffer. Then, 20 µL of the membrane preparation (diluted in assay buffer, 3 µg total protein/well) were added and the plates were incubated for 90 min at 37 °C. Subsequently, 5 µL of a furimazine solution (final assay dilution: 1:5,000 in assay buffer) were added to each well (final assay volume: 35 µL). Plates were read on a CLARIOstar microplate reader using 620/10 nm and 475/30 nm emission filters after 5 min of incubation in the dark. To determine the inhibition constants (K_i) of the non-labeled ligands, data were analyzed using the one site-fit K_i equation in PRISM10.2.1 (GraphPad, USA). Since the B_{max} value for the equilibrium K_D ($K_{D(eq.)}$) determination could only be extrapolated, due to assay interferences at higher tracer concentrations, we used the kinetic K_D value ($K_{D(kin.)}$) as a constant for the calculation of K_i values. For compounds that showed more than 50% competition at the highest concentration tested, we manually set a constraint for the curve fitting to approach the value detected for non-specific binding (0% specific BRET). For compounds that showed less than 50% competition at the highest competitor concentration tested, only the values for percentual inhibition of tracer binding at a given concentration are provided. To determine the effect of **3** on the affinity of **5**, **3** from the DMSO stock solution was added to the membrane preparation (final assay concentration: 10 µM).

Membrane-based NanoBRET association kinetic assay: 5 µL of a solution of the fluorescent ligand (**12**) diluted to varying concentrations (final assay concentrations: 1–3 µM) in assay buffer and 5 µL of assay buffer were transferred to a 384-well plate. For the determination of non-specific binding, we added a solution of a non-labeled competitor dissolved in assay buffer (**5**, final assay concentration: 10 µM) instead. After the addition of 5 µL of a furimazine solution (final assay dilution: 1:630 in assay buffer), plates were incubated for 3 minutes in the dark at ambient temperature. Subsequently, 20 µL of the membrane preparation (3 µg total protein/well) were added (final assay volume: 35 µL). BRET ratios were measured with a CLARIOstar microplate reader using 620/10 nm and 475/30 nm emission filters over time at ambient temperature. The obtained data were analyzed using the association kinetics (one ligand concentration) algorithm in PRISM 10.2.1 (GraphPad, USA) to determine association kinetics using a pre-determined k_{off} as a constraint.

Membrane-based NanoBRET dissociation kinetic assay: 5 µL of a solution of the fluorescent ligand (**12**) diluted to varying concentrations (final assay concentrations: 0.25–3 µM) in assay buffer and 5 µL of assay buffer were transferred to a 384-well plate. In order to determine non-specific binding, we added a solution of a non-labeled competitor dissolved in assay buffer (**5**, final assay concentration: 10 µM) instead of the 5 µL of assay buffer. Then, 20 µL of the membrane preparation (3 µg total protein/well) were added, and plates were incubated for 1 h at ambient temperature in the dark. Subsequently, 5 µL of a furimazine solution (final assay dilution: 1:630 in assay buffer) were added. Plates were incubated for further 5 minutes in the dark at ambient temperature. Thereafter, 1 µL of a solution of a non-unlabeled competitor dissolved in assay buffer (**5**, final assay concentration: 10 µM) was added. For control experiments, we added 1 µL of assay buffer instead of the competitor solution. BRET ratios were measured with a CLARIOstar microplate reader using 620/10 nm and 475/30 nm emission filters over time at ambient temperature. Specific BRET ratios were calculated as a difference of total and non-specific binding. The obtained data were analyzed using the dissociation - one phase exponential decay algorithm in PRISM10.2.1 (GraphPad, USA) to determine dissociation kinetics.

Membrane-based NanoBRET competition kinetic assay: 2.5 µL of a solution of the fluorescent ligand (**12**) diluted in assay buffer (final assay concentration: 1000 nM) and 2.5 µL of a solution of the competing non-labeled ligands (final assay concentrations: 0-100 nM) or **5** for determining non-specific binding (final assay concentration: 10 µM) in assay buffer were transferred to a 384-well plate. Next, 5 µL furimazine (final assay dilution: 1:750 in assay buffer) were added, the plate was centrifuged and incubated for 5 minutes in the dark at ambient temperature. Thereafter, 20 µL of the membrane preparation (3 µg total protein/well) were added and BRET ratios were measured with a CLARIOstar microplate reader using 620/10 nm and 475/30 nm emission filters over time. Specific BRET ratios were calculated as a difference of total and non-specific binding. The obtained data were analyzed using the kinetics of competitive binding algorithm in PRISM10.2.1 (GraphPad, USA) to determine competition binding kinetics.

Live cell NanoBRET: HEK293T cells were transfected with the plasmid (5.5 µg) for CCR1_Nluc using polyethylenimine (PEI; 7.5 µg) as transfection reagent as described above. After 24 h at 37 °C and 5% CO_2, the cells were detached with DMEM and transferred to a white F-bottom 384-well plate (10,000 cells/well), which was coated with 5 µL/well poly-D-lysine (0.1 mg/mL, dissolved in water), and incubated for further 24 h at 37 °C and 5% CO_2. Subsequently, cells were washed with phosphate-buffered saline (Gibco™ DPBS, with $CaCl_2$ and $MgCl_2$). Assay medium (Gibco™ DMEM/F-12, 15 mM HEPES, no phenol red supplemented with 5% FBS) was added and cells were incubated at 37 °C for 30 minutes. Then, 5 µL of a solution containing the fluorescent ligand (**12**), diluted in assay medium at varying concentrations, were added in case of saturation binding experiments. To determine non-specific binding, 5 µL of a solution of **5** dissolved in assay medium (final assay concentration: 10 µM) were added. For competition binding experiments, 5 µL of a solution of the fluorescent ligand (**12**) diluted in assay medium (final assay concentration: 1 µM) and 5 µL of a solution of the potential competitor (diluted from 10 mM DMSO-stock solutions with assay medium) at varying concentrations were added to the corresponding wells. After 90 min of incubation at 37 °C, 5 µL of a furimazine solution (final assay dilution: 1:2500, diluted with assay medium) were added. After a further incubation of 5 min in the dark at 37 °C, BRET ratios were measured with a CLARIOstar microplate reader using 620/10 nm and 475/30 nm emission filters. Total, non-specific and specific binding, which was calculated as a difference of total and non-specific binding, were analyzed using the algorithms for one-site saturation binding in PRISM10.2.1 (GraphPad, USA). To determine the inhibition constants (K_i) of the potential competitors, data were normalized to total and non-specific binding and analyzed using the one site-fit K_i equation in PRISM10.2.1 (GraphPad, USA).

Kinetic solubility assay: Kinetic solubility was assessed in saline phosphate buffer pH 7.25 (1.19 g $Na_2HPO_4 \cdot 12\,H_2O$, 0.095 g KH_2PO_4 and 4.0 g NaCl in 0.5 L double distilled water). The pH was adjusted to 7.25 with concentrated hydrochloric acid. The buffer was filtered through a 0.45 µM membrane filter prior to use. Kinetic aqueous solubility was determined by nephelometry. Measurements were performed on a NEPHELOstar Plus (BMG LABTECH, Ortenberg, Germany) at a wavelength of 635 nm. In brief, dilution series of the compounds in DMSO (1% v/v final DMSO concentration) were prepared to obtain at least three concentrations below and two concentrations above the precipitation point. 198 µL of saline phosphate buffer pH 7.25 were placed in a 96-well plate (Corning UV transparent flat-bottom 96-well-plate, Corning Incorporated, Kennebunk, ME, USA) and 2 µL of the compound DMSO stock solution was added and immediately mixed. After shaking (10 s, 500 rpm, double orbital), the measurement was carried out with a laser intensity of 80%, a beam focus of 2.5 mm at 25 °C. With the obtained data points, turbidity was plotted against the tested

concentrations and the intersection with the baseline (no precipitate) gave the kinetic solubility. The measurements were performed in triplicate and the mean value is reported (± SD).

Membrane-based radioligand binding assays: Assays were performed according to the published protocol.[13] Radioligand binding assays were performed in a 100 µL reaction volume containing varying concentrations (final concentrations: 0.001 – 10 µM) of compounds **23** and **5**, 6 nM [^{3}H]-CCR2-RA-[*R*] (specific activity 60.6 Ci mmol^{-1}, custom labeled by Vitrax in Placentia, CA, USA), and 15 µg membrane protein, all diluted in assay buffer (50mM Tris-HCl, 5 mM $MgCl_2$, 0.1% CHAPS, at pH 7.4). Membranes were obtained from Tango™ CCR1-*bla* or Tango™ CCR2-*bla* osteosarcoma (U2OS) cells stably expressing the human CCR1 (U2OS-CCR2) or human CCR2b (U2OS-CCR2) cells (Invitrogen, Carlsbad, CA, USA). Total binding was determined in the absence of competing ligand, while non-specific binding was determined in the presence of 10 µM CCR2-RA-[*R*]. After membrane addition, reaction mixtures were incubated for 2 h at 25 °C while shaking. After 2 h, reactions were terminated by harvesting through a 96-well GF/C filter plate using a PerkinElmer Filtermate harvester (PerkinElmer, Groningen, NL) and ice-cold wash buffer (50mM Tris-HCl, 5 mM $MgCl_2$, 0.1% CHAPS, at pH 7.4). Afterwards, filter plate was dried, and radioactivity was measured in a P-E-2450 Microbeta2 scintillation plate counter (PerkinElmer, Groningen, NL) after addition of 25 µL of Microscint scintillation cocktail (PerkinElmer, Groningen, NL).

Cellular NanoBiT β-arrestin recruitment assays: Chemokine-induced β-arrestin-2 recruitment to chemokine receptors CCR1 and CCR2 was monitored using a nanoluciferase complementation-based assay (NanoBiT, Promega).[17-19] 5 x 10^6 HEK293T cells were plated in 10 cm dishes and cultured for 24 h before transfection with vectors encoding for the human chemokine receptor CCR1 or CCR2 C-terminally fused with SmBiT and β-arrestin 2 N-terminally fused with LgBiT. 24 h after transfection cells were harvested, distributed into white 96-well plates (5 × 10^4 cells per well) and incubated for 1 h at 37 °C with compound **23** at concentrations ranging from 10 nM to 10 µM. The endogenous chemokine ligand CCL5 (3 nM, for CCR1) or CCL2 (10 nM, for CCR2) was then added, and luminescence generated upon nanoluciferase complementation in the presence of coelenterazine H was measured with a Mithras LB940 luminometer (Berthold Technologies) for 20 min.

Cellular NanoBiT miniGi recruitment assays: Chemokine-induced Gi recruitment to the chemokine receptor CCR1 was monitored using a nanoluciferase complementation-based assay (NanoBiT, Promega).[20] 5 x 10^6 HEK293T cells were plated in 10 cm dishes and cultured for 24 h before transfection with vectors encoding for the human chemokine receptor CCR1 C-terminally fused with SmBiT and miniGi (mG, engineered GTPase domain of Gα subunit) N-terminally fused with LgBiT. 24 h after transfection cells were harvested, distributed into white 96-well plates (5 × 10^4 cells per well) and incubated for 1 h at 37 °C with compound **23** at concentrations ranging from 10 nM to 100 µM. The endogenous chemokine ligand CCL5 (3 nM, for CCR1) was then added, and luminescence generated upon nanoluciferase complementation in the presence of coelenterazine H was measured with a Mithras LB940 luminometer (Berthold Technologies) for 20 min.

Computational Methods

Virtual screening: Due to the high similarity of the intracellular allosteric binding sites of CCR1 and CCR2 and much more detailed information on intracellular CCR2 ligands than intracellular CCR1 ligands, we performed our initial screening based on the crystal structure of CCR2 bound to CCR2-RA-[*R*] (PDB ID 5T1A),[21] adapting a recently reported protocol that was used for discovery of novel CCR5 antagonists.[22] Briefly, we sequentially screened SPECS database[23] using a pharmacophore model based on the crystallized coordinates of CCR2-RA-[*R*] using MOE[24] followed by docking in the intracellular allosteric binding site of CCR2 using GOLD.[25, 26] The resulting docked poses were then filtered to exclude those missing critical interactions with CCR2 using PLIF filtration in MOE. Finally, the top 5% of the identified hits were subsequently docked to a homology model of CCR1 (*vide infra*). After visual examination of the obtained binding modes 24 hit compounds were identified for further investigation.

Molecular docking: For the docking studies in the course of our virtual screening campaign, the CCR1 protein was downloaded in its inactive state from the GPCRdb.[27, 28] CCR1 structure was optimized using the Quickprep and structure preparation wizard in MOE[24] using CHARMM27 forcefield. The protein was saved in mol2 format and loaded in GOLD.[25, 26] The amino acids defining the IABS were listed in a text file to define the docking cavity. 50 GA runs were performed for docking CCR2-RA-[*R*] (**4**-[*R*]) and SN_12 (**43**), with the generated solutions clustered based on their RMSD with the threshold set to 0.75 Å using the complete linkage method. Docking runs were performed using ChemScore. Flexibility of amino acids Lys63^{ICL1}, Arg131$^{3.50}$, Leu240$^{6.36}$ and Arg307$^{8.49}$ was taken in consideration in the docking runs, with conformational sampling of their side chains being done by the rotamer libraries implemented in GOLD.[25, 26]
For the docking studies with our ligand-linker conjugates (Figure S1), molecular ligand structures were geometry optimized as neutral molecules by means of Avogadro (Version 1.2.0)[29] using the Universal Force Field (UFF) and the Steepest Decent Algorithm until convergence (ΔE = 0). A previously reported homology model of the inactive conformation of CCR1[13] was used for docking. The compounds were docked by means of AutoDock Vina 1.1.2.[30, 31] An exhaustiveness value of 8 and a search space of 22 x 22 x 22 Å³ was applied around the unliganded intracellular allosteric binding site of CCR1. Twenty docking poses were generated for each ligand and inspected manually and according to the docking score.

Molecular dynamics (MD) simulations: MD simulation was performed on SN_12 (**43**) in complex with CCR1 protein. For the membrane preparation, the membrane bilayer builder feature in CHARMM-GUI web interface was used, in order to embed CCR1 in a membrane bilayer system. For initial parameterization, CHARMM forcefield was used for the protein while OpenFF was used to generate CHARMM topology and parameter files for the ligand. A ratio of 1:1 palmitoyl-oleoyl-phosphatidylcholine (POPC) molecules in the upper and lower leaflet was used and NaCl molecules were added, in order to neutralize the system and reach a concentration of 150 mM, resembling cellular conditions, with the ions' placement method selected as Monte-Carlo method. The resulting complex, embedded in a membrane bilayer, consisted of 622 POPC molecules, 329 sodium ions, 331 chloride ions. The system was solvated with approximately 119338 water molecules in a 150.1 x 150.1 x 209.3 Å box. Finally, input files for minimization, equilibration, and production runs were generated for AMBER package, using the AMBER forcefield option in CHARMM-GUI; FF19SB for protein, Lipid21 for lipids, TIP3P for water molecules and GAFF2 for the ligand with charges being assigned using AM1-BCC method.[32] Hydrogen mass partitioning option was selected in order to accelerate the MD simulation through repartitioning atomic masses, where hydrogen atom mass increases by a factor of 3 and the increased mass is subtracted from the linked heavy atom, which allows an increase in the simulation time step up to 4 fs. MD simulations were performed using the CUDA version of PMEMD (Particle Mesh Ewald Molecular Dynamics)[33] of the AMBER Molecular Dynamics Package[34] following a standard protocol adopted of minimization, equilibration and production using the default settings produced by CHARMM-GUI. The number of residues was set to 356 (355 protein residues in addition to the ligand), whenever restraints are applied. The trajectory lengths for density equilibration and production were 1.875 ns and 100 ns, respectively. Minimization was applied with a maximum of 5000 steps using the steepest-descent steps method, with restraints applied to protein residues, with a force constant of 10 kcal/mol. Å^2, and restraints applied to the membrane, with a force constant of 2.5 kcal/mol. Å^2. Heating was employed at 303.15 K using the Langevin thermostat, while density equilibration and production were conducted at constant pressure (1 bar). Langevin dynamics were generally employed, non-bonded interactions were cut off at 9.0 Å, and the Particle-Mesh Ewald method was used to treat long range electrostatics under periodic boundary conditions. The trajectories were analyzed using CPPTraj,[35] while plots and visual inspection of the trajectories was done using XMgrace[36] and VMD,[37] respectively.

Supplementary Figures

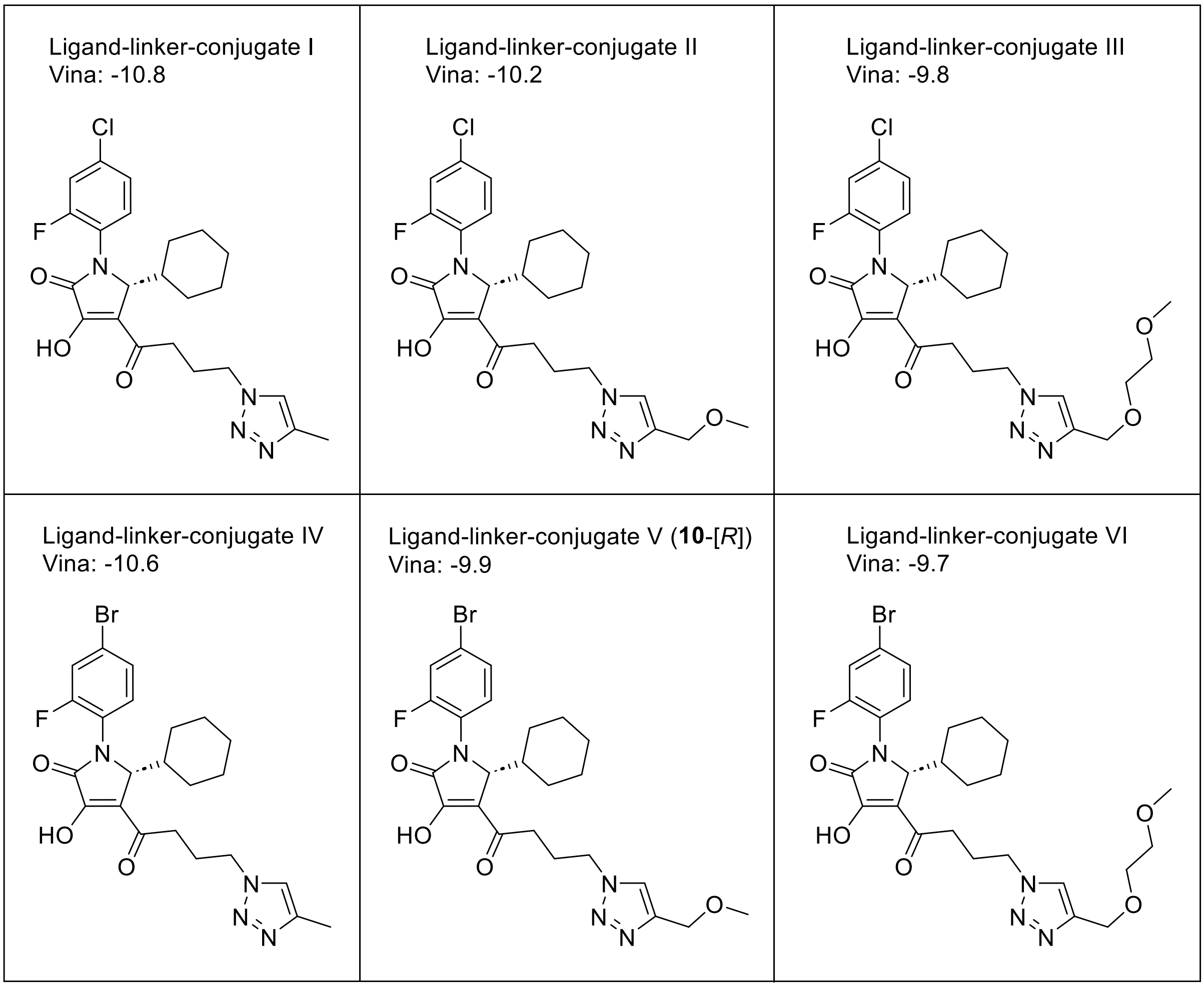

Reference compounds: (**4**-[*R*]), Vina: -10.6; **5**-[*R*], Vina: -10.4

Figure S1. Identification of suitable linker fragments and linker lengths for the design of fluorescently labeled CCR1 ligands based on the scaffolds of CCR2-RA (**4**) cmpd39 (**5**). A) Chemical structures and docking scores of designed CCR1 ligand-linker conjugates docked into the published homology model of CCR1,[13] which is based on the co-crystal structure of CCR2 and **4**-[*R*] (PDB ID: 5T1A).[21] The reported intracellular allosteric CCR1/CCR2 inhibitors **4**-[*R*] and **5**-[*R*] were used as a reference compounds for the docking study.[13] The design of ligand-linker conjugates is based on the predicted binding modes of the intracellular CCR1 antagonists,[13] available SAR data,[13] and the synthetic accessibility of the final fluorescent ligands.

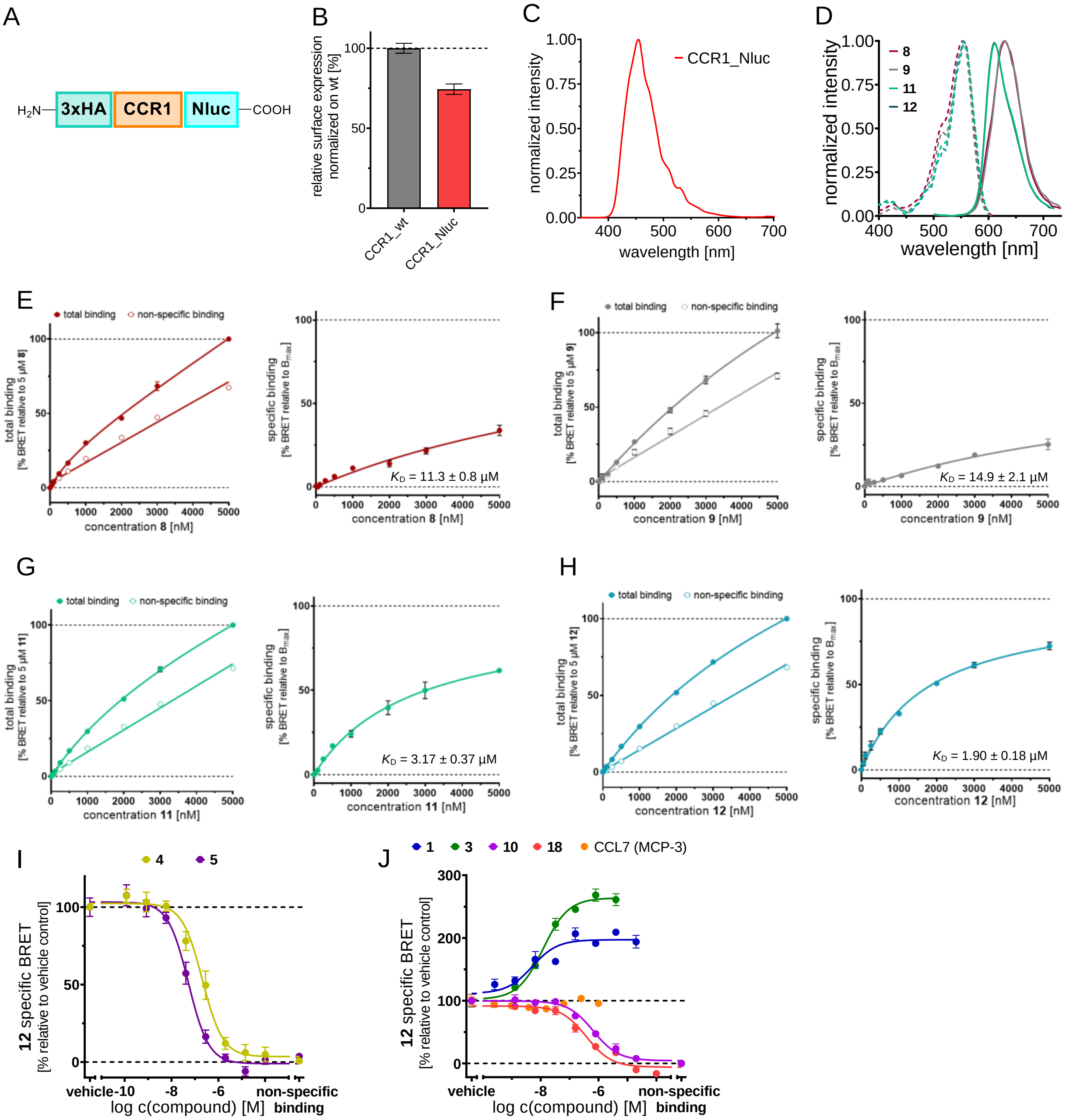

Figure S2. Development of a cell-free NanoBRET-based binding assay for CCR1. A) Schematic representation of the genetic construct of CCR1 (hereafter referred to as CCR1_Nluc)[1] used in the course of assay development. B) Expression level of CCR1_Nluc detected via ELISA normalized to the expression wild-type 3xHA-CCR1 (CCR1_wt). Bar diagram representing the mean values ± SEM (n = 3) with each test performed in quadruplicate. The experiment confirms that CCR1_Nluc is well-expressed in HEK293T cells. C) Emission spectra of CCR1_Nluc using membrane preparations from HEK293T cells expressing the respective C-terminally Nluc-labeled 3xHA-CCR1 fusion protein. D) Spectral properties of the novel fluorescent CCR1 ligands (**11**-**12**) compared to previously reported fluorescent ligands **8**-**9**.[1] Absorption and fluorescence emission spectra of the fluorescent ligands were normalized to the respective maximum signal of each sample. Excitation spectra of the fluorescent ligands are shown as dashed lines, emission spectra of the fluorescent ligands are represented as solid lines. E-H) Binding curves (total and non-specific binding on the left, specific binding on the right) with fluorescent ligands **8** (n = 3, see E), **9** (n = 3, see F), **11** (n = 4, see G), and **12** (n = 4, see H) using membrane preparations from HEK293T cells expressing the CCR1_Nluc construct. Single experiments were performed in triplicate. I) Representative competition binding curves from single experiment with the reference inhibitors CCR2-RA (**4**, dark yellow) and cmpd39 (**5**, dark purple), obtained with **12** (2000 nM) and CCR1_Nluc membranes (mean ± SEM, triplicate measurement). J) Representative competition binding curves from single experiments with the allosteric extracellular CCR1 inhibitor BX-471[38] (**1**, dark blue), the CCR1 antagonist BI-639667[39] (**3**, dark green) with a previously unknown binding site, the extracellular orthosteric agonist CCL7 (orange), the ligand-linker conjugate V (**10**, purple), and the CX3CR1 antagonist AZD8797 (**18**, salmon)[40] with reported off-target binding to CCR1, obtained with **12** (2000 nM) and CCR1_Nluc membranes (mean ± SEM, triplicate measurement).

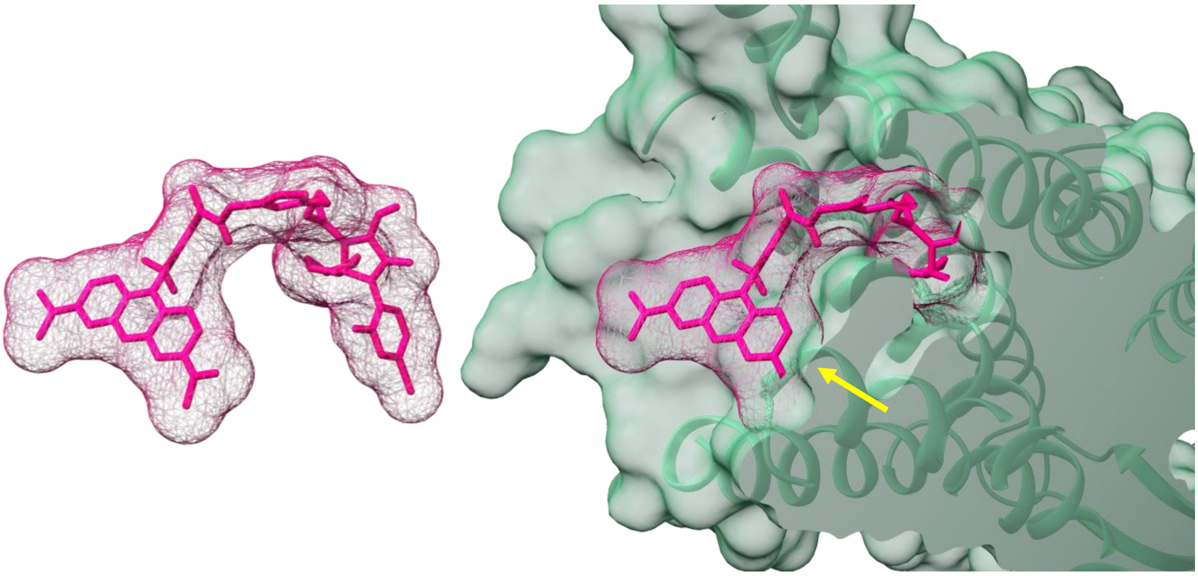

Figure S3. Predicted CCR1 binding mode of **9** (magenta, molecule surface shown as grid line) indicates the potential of steric clashes (yellow arrow) of the attached TAMRA fluorophore with amino acids at the entrance of the IABS of CCR1 (green). For comparison, the unbound **9** is shown on the left.

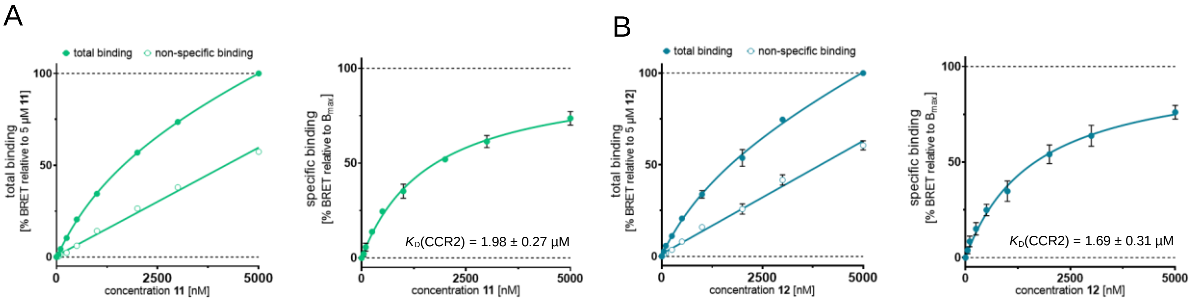

Figure S4. Selectivity studies with fluorescent CCR1 tracers **11**-**12** regarding their CCR2 affinity using a previously reported membrane based NanoBRET binding assay.[1] A) Binding curves (total and non-specific binding on the left, specific binding on the right) with fluorescent ligand **11** (n = 4) using membrane preparations from HEK293T cells expressing the CCR2_GSSG_Nluc construct. Single experiments were performed in triplicate. B) Binding curves (total and non-specific binding on the left, specific binding on the right) with fluorescent ligand **12** (n = 4) using membrane preparations from HEK293T cells expressing the CCR2_GSSG_Nluc construct. Single experiments were performed in triplicate.

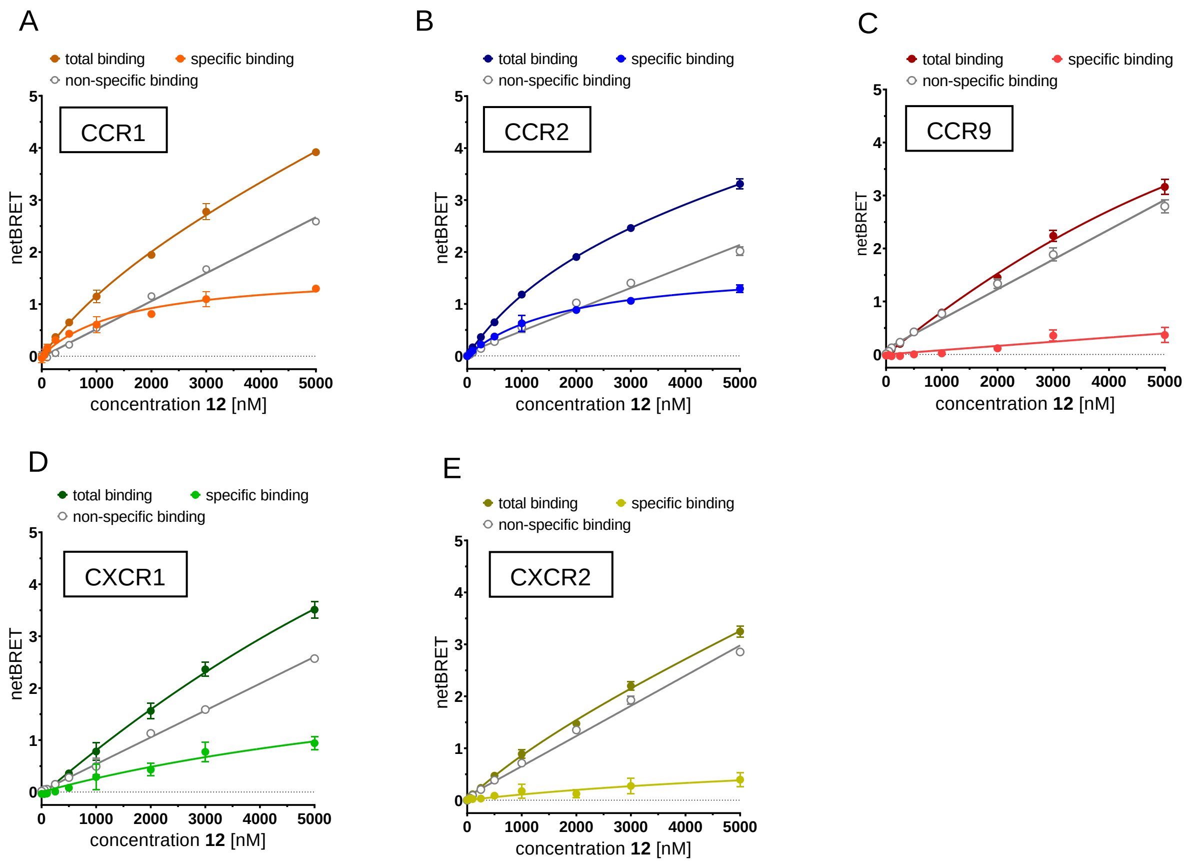

Figure S5. Selectivity studies with the fluorescent ligand **12** for further chemokine receptors. A) Representative saturation binding curves (total, specific, and non-specific binding) using membrane preparations from HEK293T cells expressing the CCR1_Nluc construct, as published by Toy *et al*.[1] The experiments were performed in triplicate (n=4). B) Representative saturation binding curves (total, specific, and non-specific binding) using membrane preparations from HEK293T cells expressing the CCR2_GSSG_Nluc construct, as published by Toy *et al*.[1] The experiments were performed in triplicate (n=4). C) Representative saturation binding curves (total, specific, and non-specific binding) using membrane preparations from HEK293T cells expressing the CCR9_Nluc construct, as published by Huber *et al*.[3] The experiments were performed in triplicate (n=3). D) Representative saturation binding curves (total, specific, and non-specific binding) using membrane preparations from HEK293T cells expressing the CXCR1_Nluc construct, as published by Huber *et al*.[8] The experiments were performed in triplicate (n=3). E) Representative saturation binding curves (total, specific, and non-specific binding) using membrane preparations from HEK293T cells expressing the CXCR2_Nluc construct, as published by Huber *et al*.[8] The experiments were performed in triplicate (n=3).

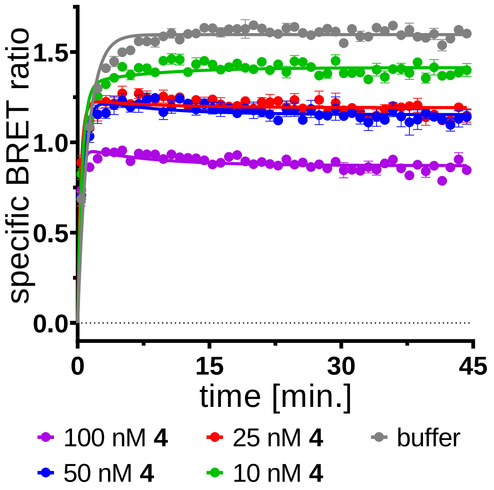

Figure S6. Representative kinetic competition binding curves (mean ± SEM, triplicate measurement, n = 4) for **4**, obtained with **12** (1000 nM) and CCR1_Nluc membranes.

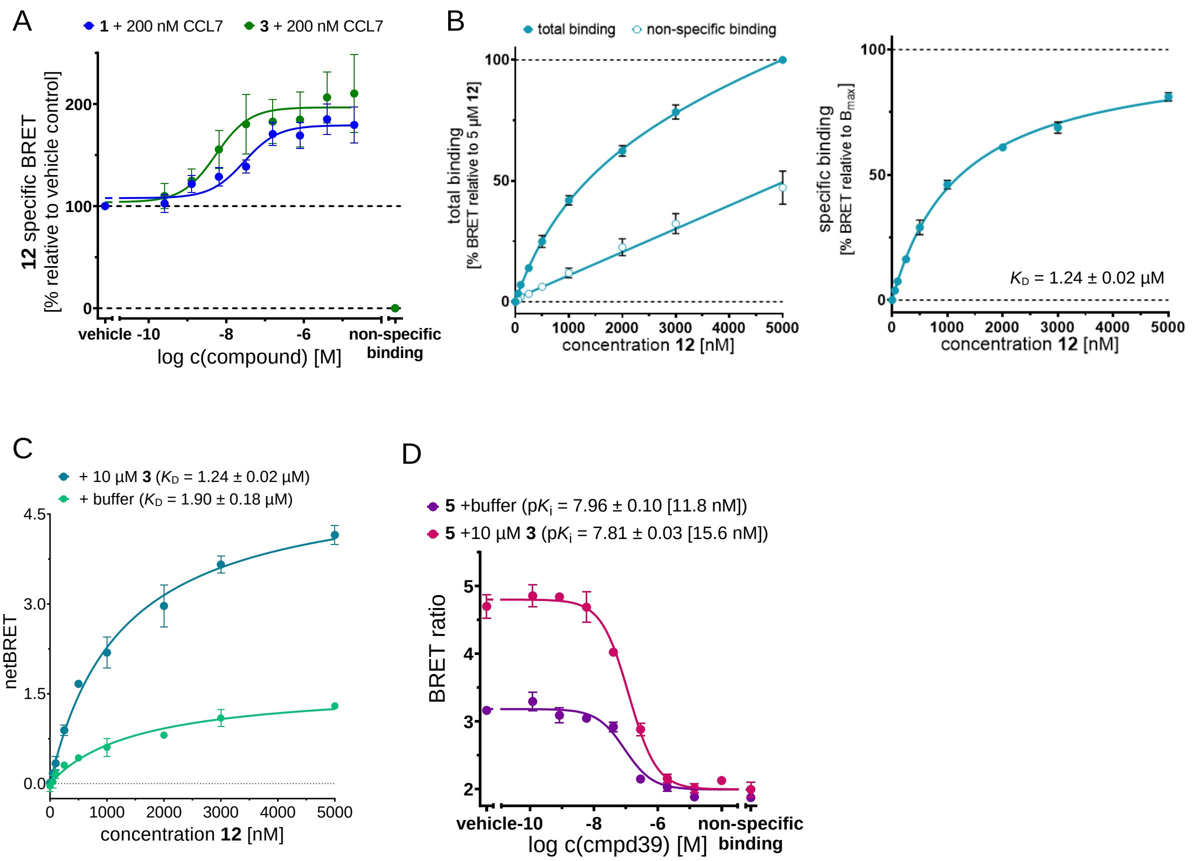

Figure S7. Application of the fluorescent CCR1 ligand **12** as a tool to mechanistically characterize CCR1 ligands with unknown binding sites. A) Competition binding curves with the allosteric extracellular CCR1 inhibitor BX-471[38] (**1**, blue, n = 3), the CCR1 antagonist BI-639667[39] (**3**, green, n = 3) in the presence of the extracellular orthosteric agonist CCL7 (200 nM). Measurements were performed in triplicates. B) Binding curves (total and non-specific binding on the left, specific binding on the right) with fluorescent ligand **12** in the presence of **3** (10 µM) using membrane preparations from HEK293T cells expressing the CCR1_Nluc construct (n = 3). Single experiments were performed in triplicate. C) Comparison of representative saturation binding curves (specific binding) with fluorescent ligand **12** in the presence (turquoise) and absence (sea green) of **3** (10 µM), using membrane preparations from HEK293T cells expressing the CCR1_Nluc construct. Experiments were performed in triplicate. D) Comparison of representative competition binding curves for **5**, obtained with fluorescent ligand **12** in the presence (pink) and absence (purple) of **3** (10 µM), using membrane preparations from HEK293T cells expressing the CCR1_Nluc construct. Experiments were performed in triplicate.

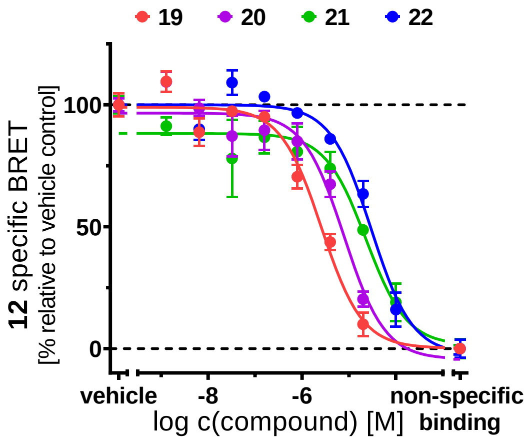

Figure S8. Representative competition binding curves from single experiments with the intracellular chemokine receptor antagonists **19**-**22**.[1, 3, 8, 16], obtained with **12** (2000 nM) and CCR1_Nluc membranes (mean ± SEM, triplicate measurement).

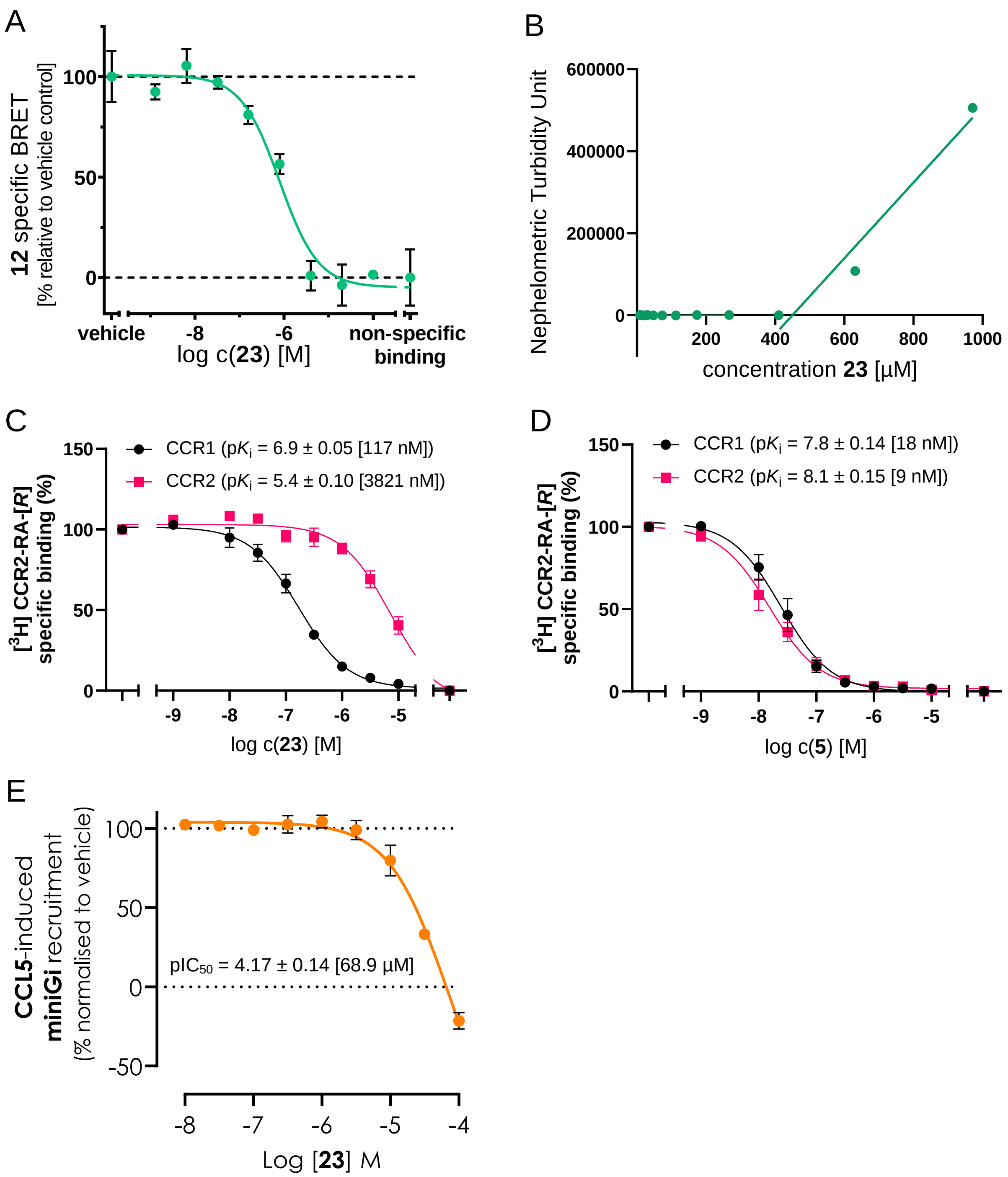

Figure S9. Compound **23** as a new intracellular CCR1 ligand with CCR1 over CCR2 selectivity. A) Representative competition binding curve from a single NanoBRET-based experiment with the intracellular chemokine receptor antagonist **23**, obtained with **12** (2000 nM) and membranes from HEK293T cells transiently expressing CCR1_Nluc (mean ± SEM, triplicate measurement). B) Representative graph (n = 3) from nephelometry to determine the kinetic aqueous solubility of **23**. C) Displacement curves for the intracellular CCR1 inhibitor **23**, obtained with the radioligand [^{3}H]CCR2-RA-[*R*] (6 nM) and membranes from U2OS cells stably expressing the human CCR1 (black) or CCR2 (pink). Experiments were performed at 25 °C (mean ± SEM, duplicate measurement, n = 3). D) Control experiments with the dual intracellular CCR1/2 inhibitor cmpd39 (**5**).[13] Displacement curves were obtained with the radioligand [^{3}H]CCR2-RA-[*R*] (6 nM) and membranes from U2OS cells stably expressing the human CCR1 (black) or CCR2 (pink). Experiments were performed at 25 °C (mean ± SEM, duplicate measurement, n = 3). E) Concentration-response curve from a cellular CCR1 NanoBiT miniGi recruitment assay with **23** in the presence of CCR1 agonist CCL5 (3 nM). pIC_{50} value (mean ± SEM, n = 3). A concentration–response curve for CCL5-mediated CCR1 activation is shown in Figure S10C.

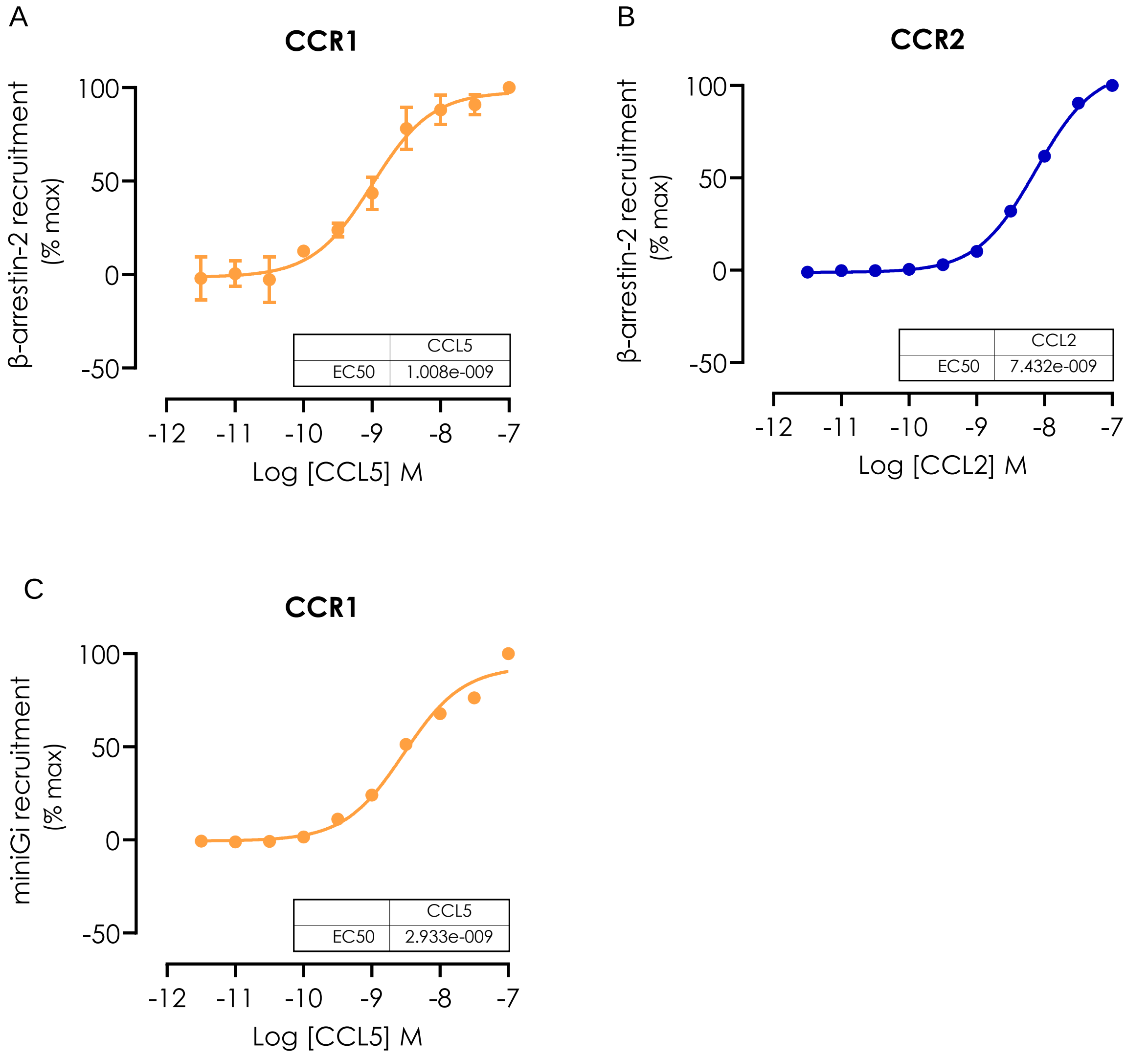

Figure S10. Concentration-dependent activation of CCR1 or CCR2 by their endogenous agonists. A) Concentration-response curve for CCL5-mediated CCR1 activation in a NanoBiT β-arrestin 2 recruitment assay (n = 3). B) Concentration-response curve for CCL2-mediated CCR2 activation (n =1) in a NanoBiT β-arrestin 2 recruitment assay. C) Concentration-response curve for CCL5-mediated CCR1 activation in a NanoBiT miniGi recruitment assay (n = 3).

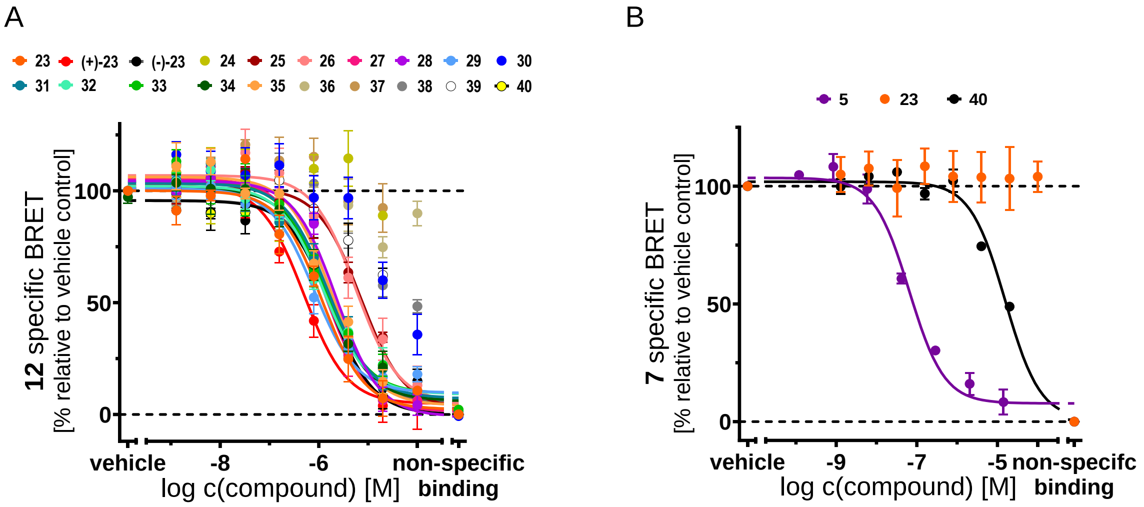

Figure S11. Structure activity-relationship studies for **23**. A) CCR1 competition binding curves for the intracellular chemokine receptor antagonist **23**, its enantiomers, and its analogues **24**-**40** obtained with **12** (2000 nM) and CCR1_Nluc membranes (mean ± SEM (n = 3), triplicate measurement). For compounds that showed less than 50% competition at 20 µM no fit was used. B) CCR2 competition binding curves for **23** and its analogue **40** obtained by using a NanoBRET competition binding assay as previously reported (mean ± SEM (n = 3), triplicate measurement).[1] Compound **5** is shown as a reference for a high affinity dual CCR1/CCR2 inhibitor.

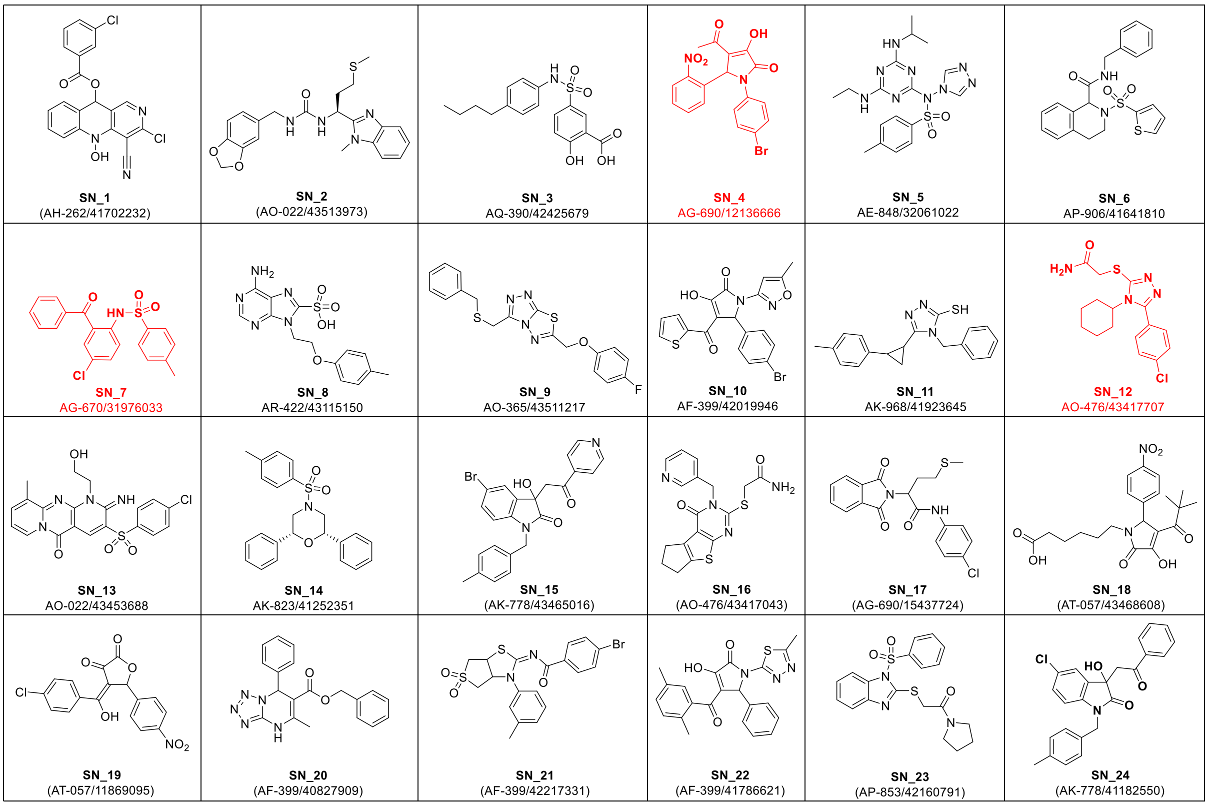

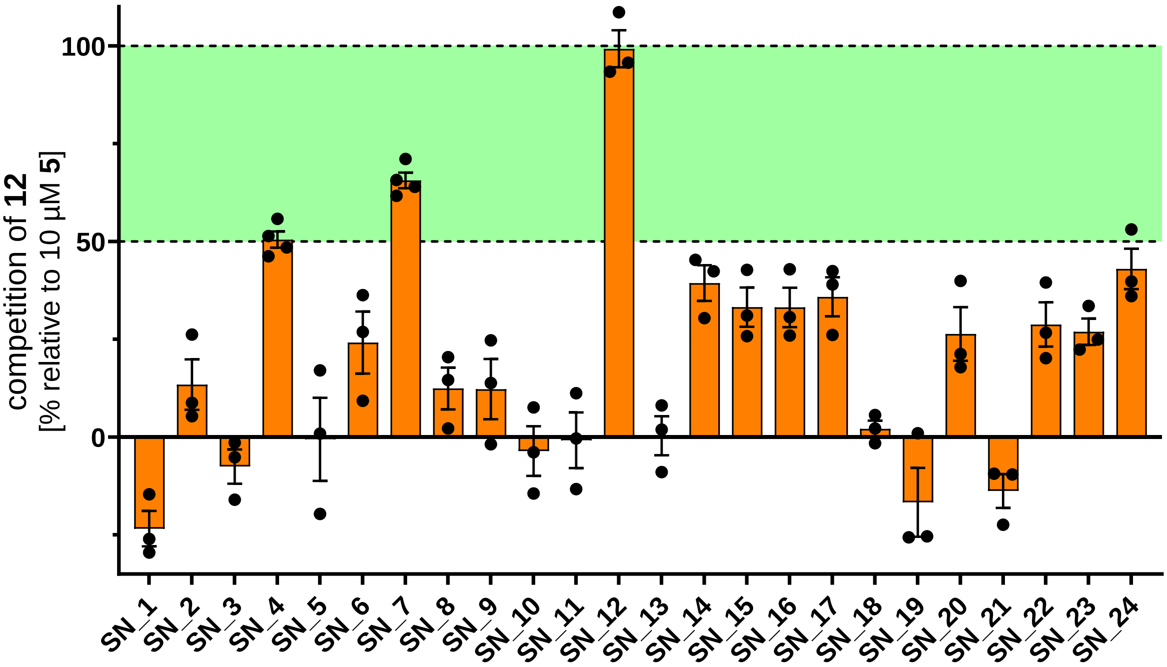

Figure S12. Chemical structures of virtual screening hits (above, Specs ID is given in brackets) and their percentual CCR1 inhibition at a compound concentration of 20 µM (below). Inhibition data obtained with **12** (2000 nM) and CCR1_Nluc membranes (mean ± SEM (n ≥ 3, at least duplicate measurement). Chemical structures of compounds that evoked ≥ 50% inhibition at 20 µM are shown in red.

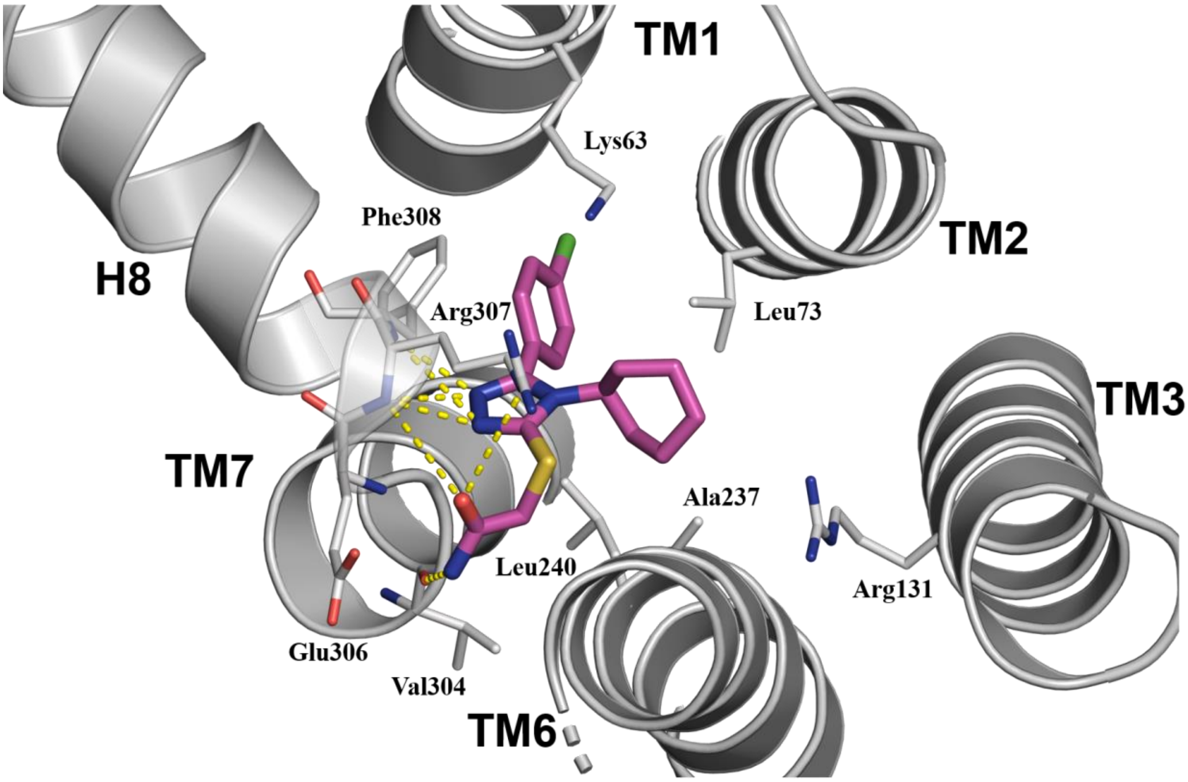

Figure S13. Suggested docking pose of SN_12 (**43**) bound to the IABS of CCR1. The 1,2,4-triazole of **43** is predicted to interact via hydrogen bonds with the backbone amide of Arg307$^{8.49}$ and Phe308$^{8.50}$, in a similar manner as reported for the pyrrolone of CCR2-RA-[*R*] in CCR2.[21] The thioacetamide moiety extends further into the outer polar region, forming hydrogen bond interactions with the backbone of Val304$^{7.56}$ and the side chain of Arg307$^{8.49}$. The 4-chlorophenyl ring of **43** is predicted to interact with Lys63^{ICL1} via a cation-π interaction. Furthermore, the cyclohexyl ring of **43** is predicted to interact with Ala237$^{6.33}$ and Leu240$^{6.36}$ via hydrophobic interactions. Figure generated by PyMOL.[41]

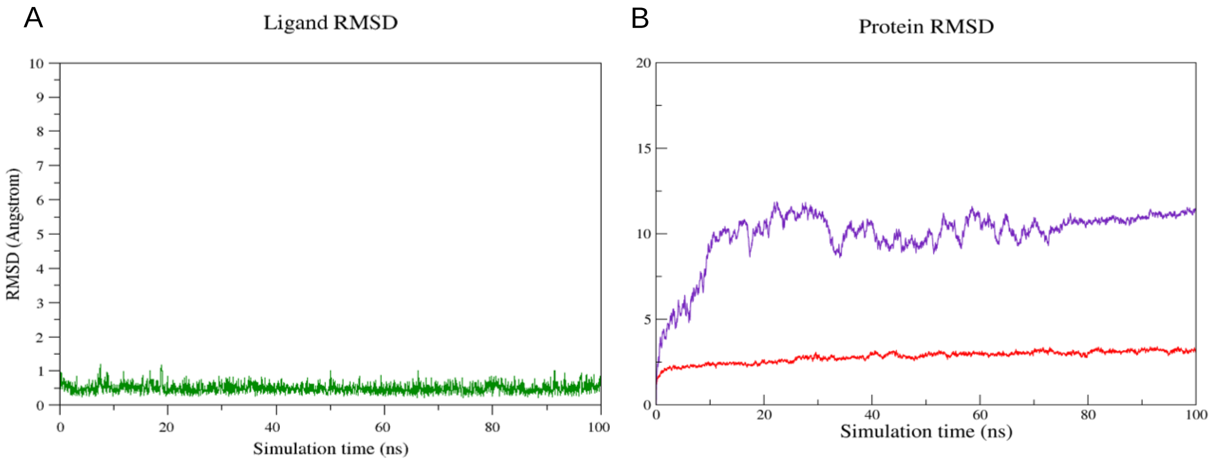

Figure S14. Molecular dynamics (MD) simulations confirm the predicted CCR1 binding mode of SN_12 (**43**). A) Plots of root mean square deviations of **43** heavy atoms along the MD simulation time. B) Plots of root mean square deviations of protein main chain atoms along the MD simulation time. **43** heavy atoms plot colored green, protein main chain atoms plot colored indigo, and protein main chain atoms plot excluding termini residues colored red. The MD simulations confirm the accuracy of the predicted binding mode with less than 1 Å ligand RMSD fluctuations relative to the initial docked pose during the entire 100 ns simulation time, highlighting the stability and reliability of the docked pose of **43** in the IABS of CCR1. The MD simulations were conducted over a simulation time of 100 ns using AMBER software.

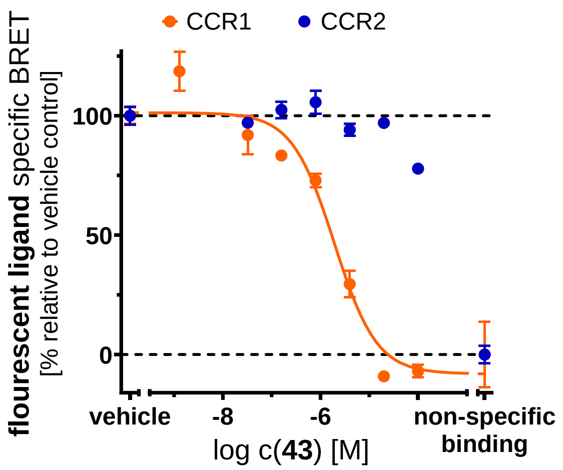

Figure S15. Characterization of SN_12 (**43**) as a selective intracellular CCR1 inhibitor. Representative competition binding curves from single experiments with **43**. CCR1 data (orange) obtained with **12** (2000 nM) and CCR1_Nluc membranes (mean ± SEM, triplicate measurement). CCR2 data (blue) obtained by using a NanoBRET competition binding assay as previously reported (mean ± SEM, triplicate measurement).[1]

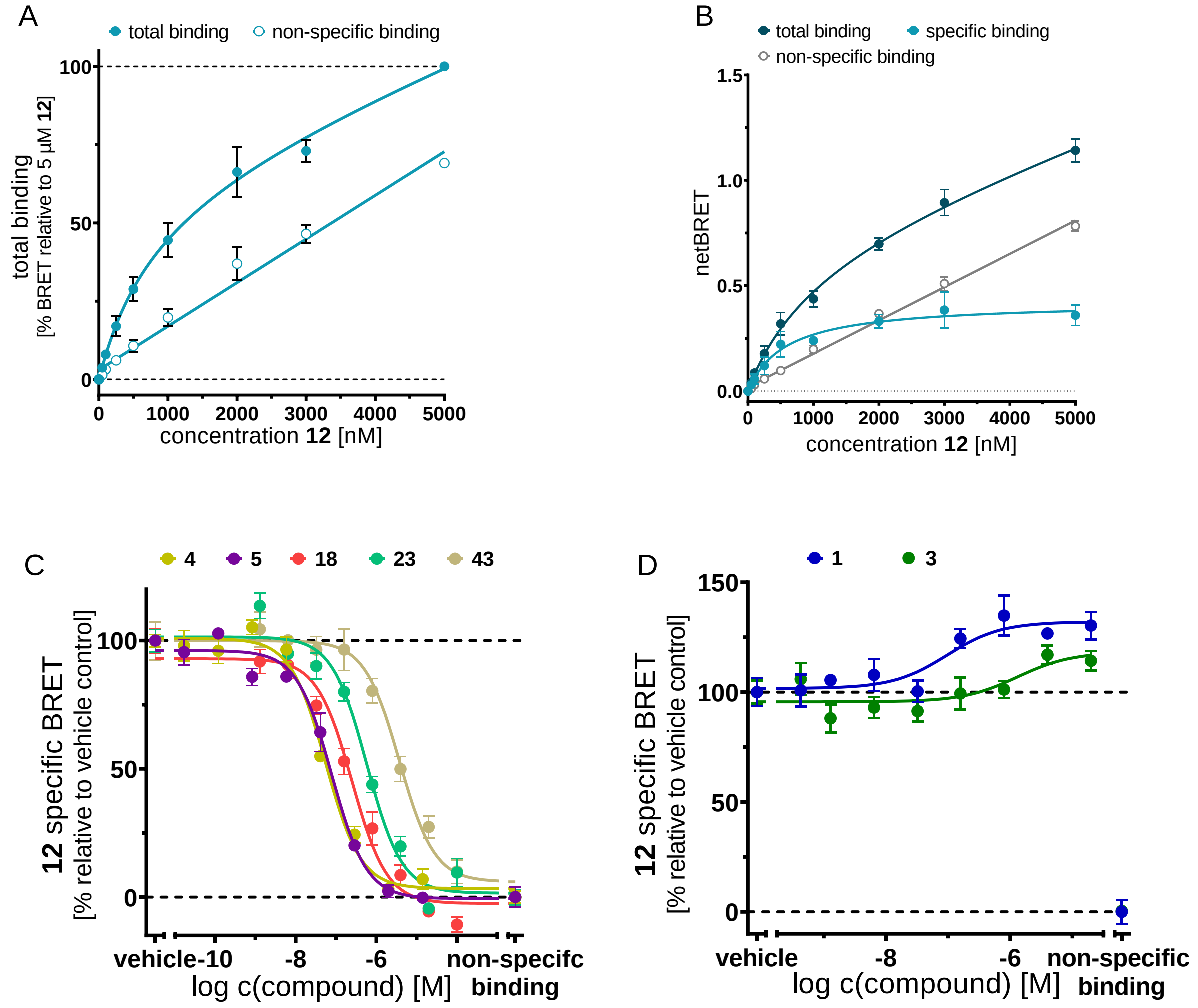

Figure S16. Application of **12** as a fluorescent tracer for a cellular NanoBRET-based CCR1 binding assay, using live HEK293T cells expressing CCR1_Nluc. A) Saturation binding curves (total and non-specific binding) of **12** in a cellular NanoBRET-based experiment (mean ± SEM, quadruplicate measurement, n = 3). B) Representative saturation binding curves from single experiments (total, specific, and non-specific binding) of **12** in a cellular NanoBRET-based experiment (mean ± SEM, quadruplicate measurement, n = 3). C) Representative competition binding curves from single experiments (mean ± SEM, quadruplicate measurement, n =4) for CCR2-RA (**4**, dark yellow), cmpd39 (**5**, purple), AZD8797 (**18**, salmon), V_2R_{inh}-02 (**23**, sea green), and SN_12 (**43**, khaki) obtained with **12** (1000 nM). D) Representative binding curves (mean ± SEM, quadruplicate measurement, n = 4) of BX-471 (**1**, dark blue), and BI-639667 (**3**, dark green) obtained with **12** (1000 nM).

Supplementary Schemes

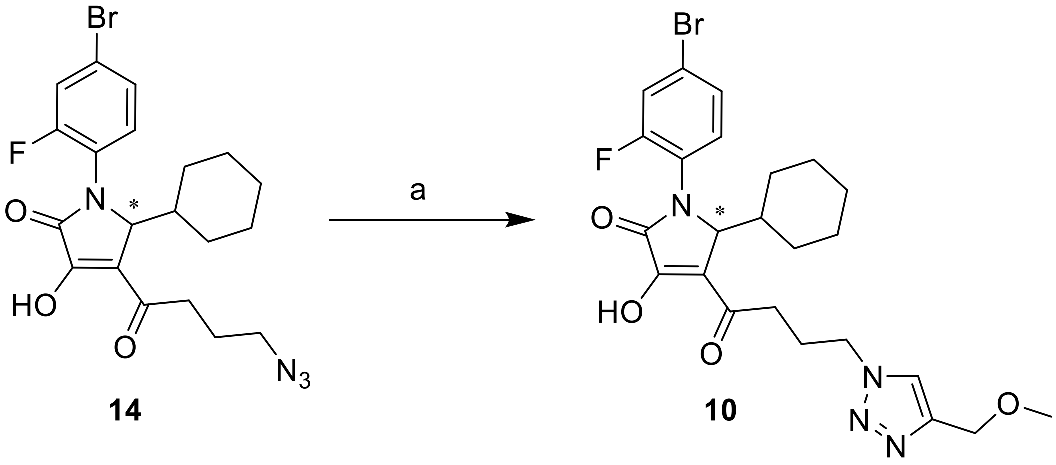

Scheme S1. Synthesis of the ligand−linker conjugate V (**10**). Reagents and conditions: a) 3-methoxyprop-1-yne, $CuSO_4 \cdot 5\ H_2O$, sodium ascorbate, TBTA, water/*tert*-BuOH/DMF mixture (1:1:1 (v/v)), rt, 1 h, 57% yield.

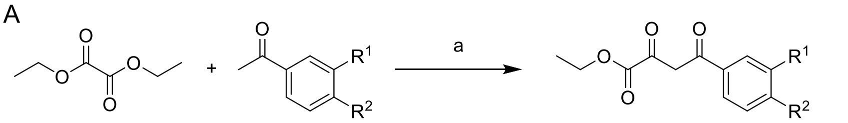

44 (R^1 = -H, R^2 = -H)
45 (R^1 = -OMe, R^2 = -H)
46 (R^1 = -H, R^2 = -OH)

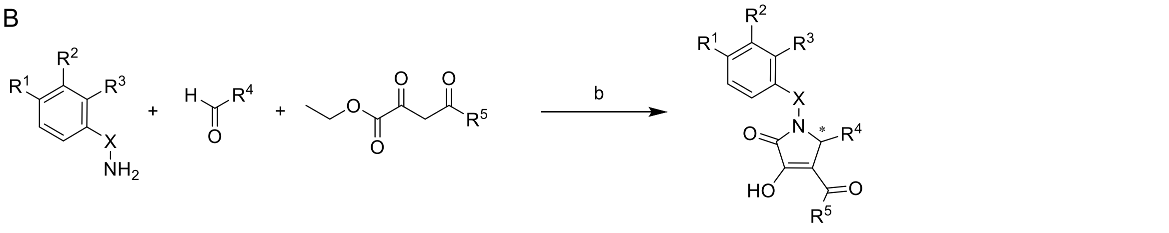

	R^1	R^2	R^3	X	R^4	R^5
**24**	-OH	-H	-H	$-CH_2-CH_2-$	*c*-hexyl	Me
**25**	-H	-H	-H	$-CH_2-CH_2-$	4-F Ph	Ph
**26**	-F	-H	-H	$-CH_2-CH_2-$	4-F Ph	Ph
**28**	-H	-H	-OH	$-CH_2-CH_2-$	4-F Ph	Ph
**29**	-OH	-H	-H	$-CH_2-$	4-F Ph	Ph
**30**	-OH	-H	-H	-	4-F Ph	Ph
**31**	-OH	-H	-H	$-CH_2-CH_2-$	4-Br Ph	Ph
**32**	-OH	-H	-H	$-CH_2-CH_2-$	3-Br Ph	Ph
**33**	-OH	-H	-H	$-CH_2-CH_2-$	4-Cl Ph	Ph
**34**	-OH	-H	-H	$-CH_2-CH_2-$	*c*-hexyl	Ph
**35**	-OH	-H	-H	$-CH_2-CH_2-$	Ph	Ph
**36**	-OH	-H	-H	$-CH_2-CH_2-$	4-F Ph	*c*-Pr
**37**	-OH	-H	-H	$-CH_2-CH_2-$	3-Br Ph	*c*-Pr
**38**	-OH	-H	-H	$-CH_2-CH_2-$	4-F Ph	4-OH Ph
**40**	-Br	-H	-F	-	4-F Ph	Ph

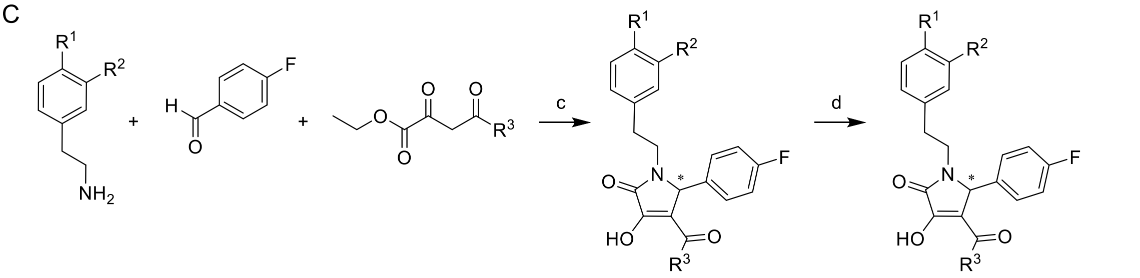

	R^1	R^2	R^3
[**47**]	-H	-OMe	Ph
**48**	-OH	-H	3-OMe Ph

	R^1	R^2	R^3
**27**	-H	-OH	Ph
**39**	-OH	-H	3-OH Ph

Scheme S2. Synthesis of analogues **24**-**40** derived from the newly discovered intracellular CCR1 antagonist **23**. A) Synthesis of the 2,4-dioxo ester building blocks. Reagents and conditions: a) for **44**: NaH, THF, 0 °C to reflux, 1 h, 30% yield; for **45** and **46**: NaOEt, EtOH, rt, 48 h, 59% yield. B) One-step procedure for the synthesis of the pyrrolones **24**-**26**, **28**-**38**, and **40**: Reagents and conditions: b) acetic acid, 80-95 °C, 5-48 h, 9-58% yield. C) Two-step procedure for the synthesis of the pyrrolones **27** and **39**. Reagents and conditions: c) acetic acid, 90 °C, 18-20 h; d) BBr_3, CH_2Cl_2, rt, 3-4 h, 6-7% yield over two steps.

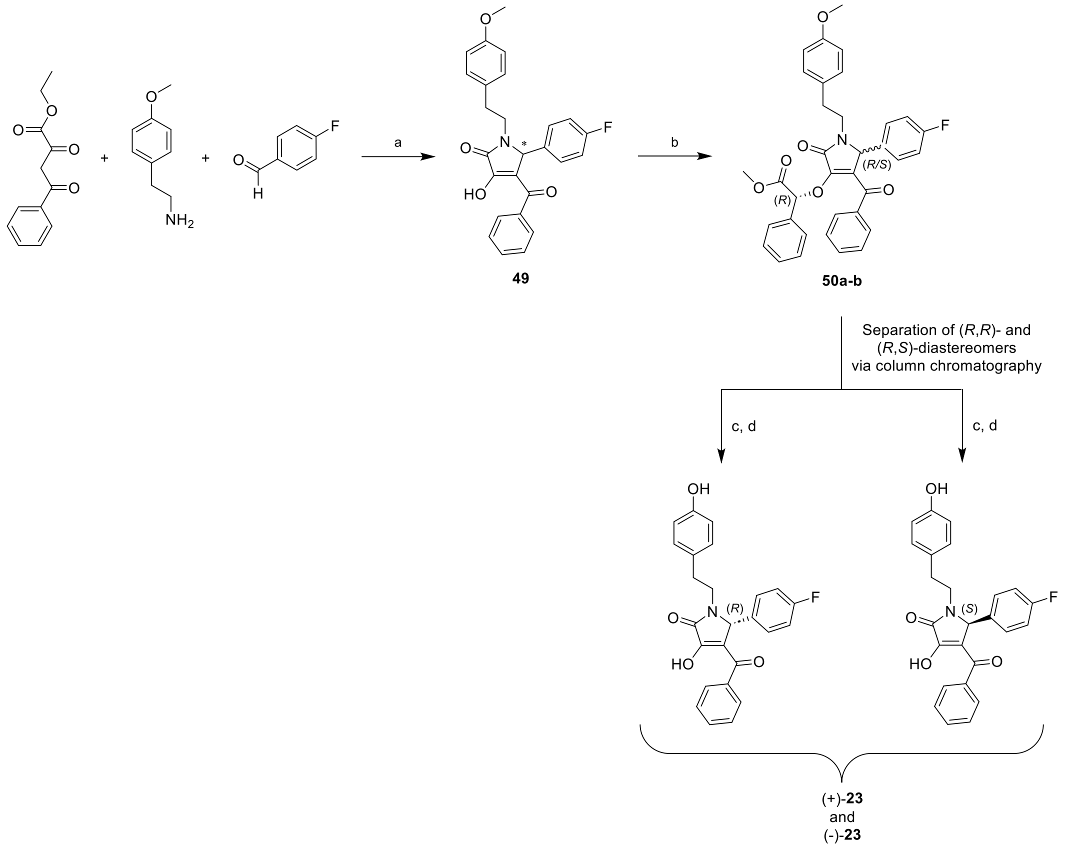

Scheme S3. Stereoselective synthesis (+)-**23** and (-)-**23**. Reagents and conditions: a) acetic acid, 90 °C, 18 h, 49% yield; b) methyl (*S*)-(+)-mandelate, PPh_3, DIAD, THF, 0 °C to rt, 12 h, separation of the diastereomers was achieved by normal phase column chromatography (EtOAc/isohexane, gradient 5–30%), 38% (combined yield for both diastereomers); c) LiCl, 1,3-dimethyl-2-imidazolidinon (DMEU), 80 °C, 24 h; d) BBr_3, CH_2Cl_2, 0 °C to rt, 16 h, 17-37% yield over two steps.

Supplementary Tables

Table S1. Kinetic parameters detected for the interaction between **12** and CCR1 by using our membrane-based NanoBRET assay and by applying the indicated conditions. A) Dissociation. B) Association. For the calculation of k_{on}, a pre-determined k_{off} of 0.1397 min^{-1} was used.

A

Dissociation at room temperature (membranes)

	k_{off} [min^{-1}]	t_R [min]
3000 nM	0.1034	9.67
	0.1266	7.90
	0.1390	7.19
	0.1552	6.44
	0.1187	8.42
	0.1188	8.42
	0.1563	6.40
	0.1612	6.20
2000 nM	0.1235	8.10
1000 nM	0.1287	7.77
	0.1387	7.21
	0.1385	7.22
	0.1406	7.11
	0.1366	7.32
	0.1476	6.78
	0.1740	5.75
	0.1692	5.91
250 nM	0.1187	8.42
	0.1067	9.37
	0.1422	7.03
	0.1477	6.77
	0.1688	5.92
	0.1691	5.91
	0.1484	6.74
	0.1147	8.72
mean	**0.1397**	**7.31**
SEM	0.0041	0.22

B

Association at room temperature (membranes)

	k_{on} [M^{-1} min^{-1}]
3000 nM	318264
	213374
	384190
	297524
	440003
	370730
	337773
2000 nM	249782
1000 nM	620193
	367336
	231762
	478819
	220044
	432188
	557225
	297545
	525954
	494479
250 nM	737710
	971395
	722175
mean	**441355**
SEM	42736

Supplementary NMR Spectra

NMR spectra for compounds **10**-**12, 15, 23**-**40, 44**-**46,** and **48** can be found on the following pages.

^{1}H NMR (600 MHz, DMSO-d_6) for compound **10**

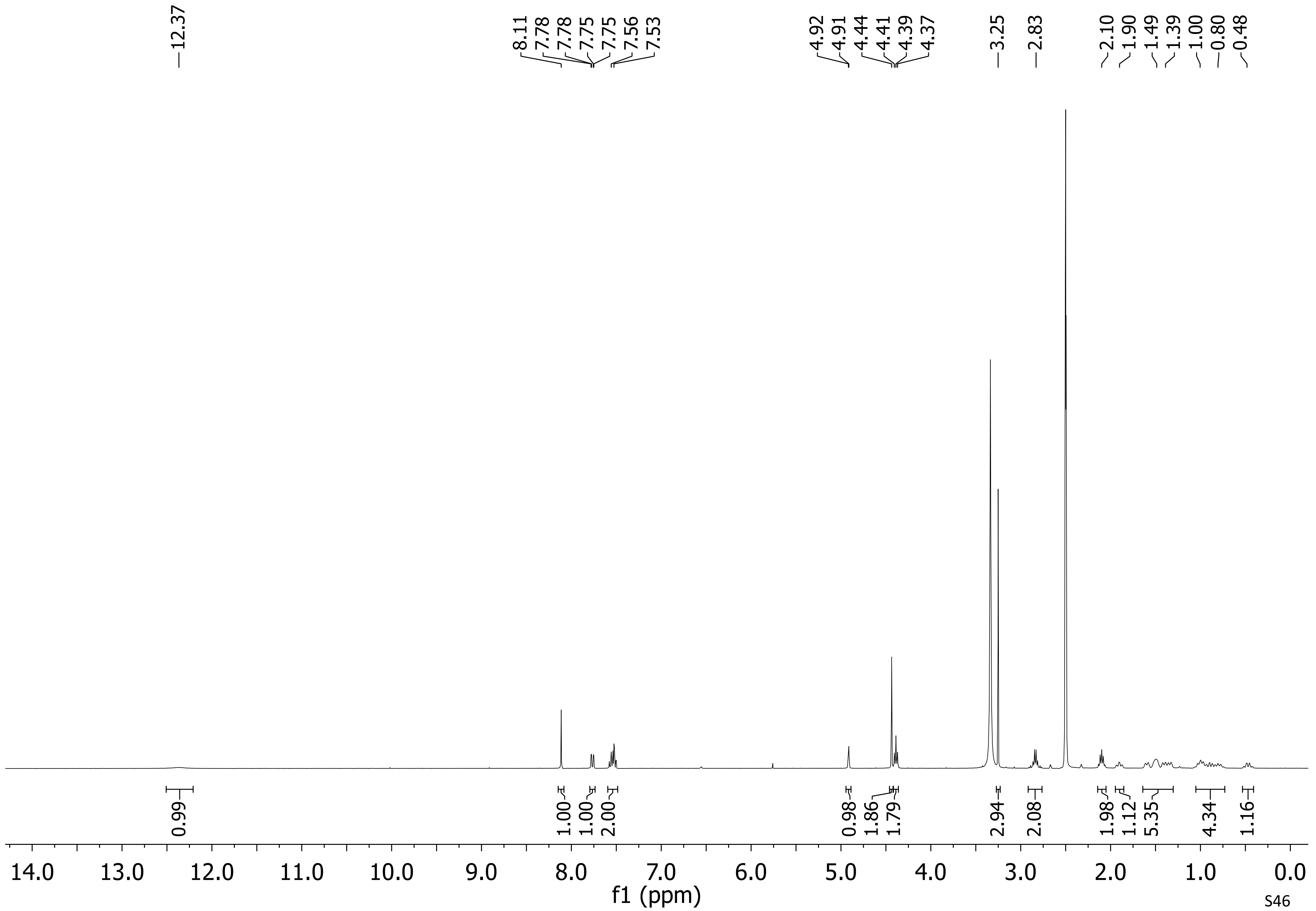

DEPTQ ^{13}C NMR (151 MHz, DMSO-d_6) for compound **10**

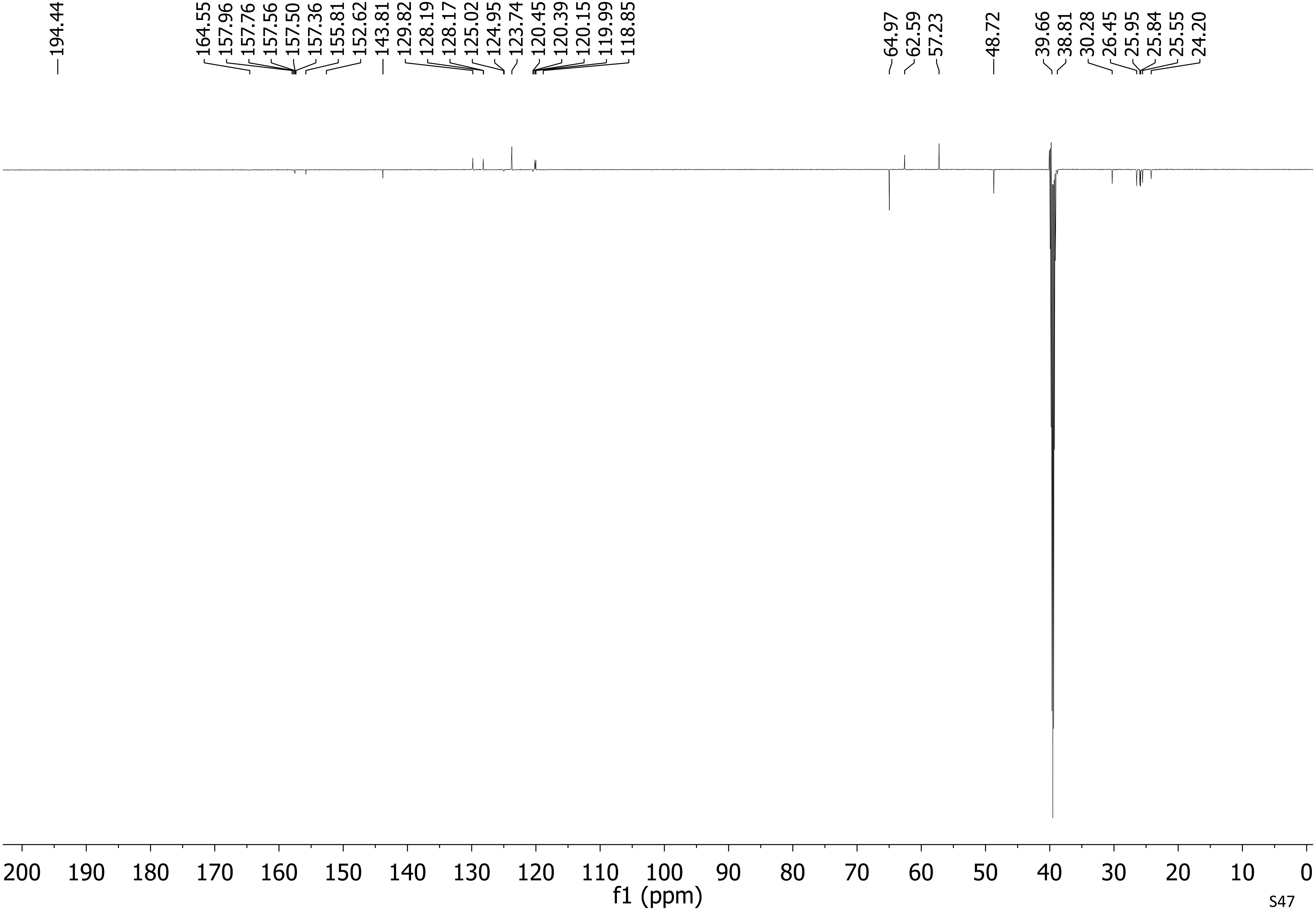

DEPTQ ^{13}C NMR (151 MHz, DMSO-d_6) for compound **10** (zoomed-in view)

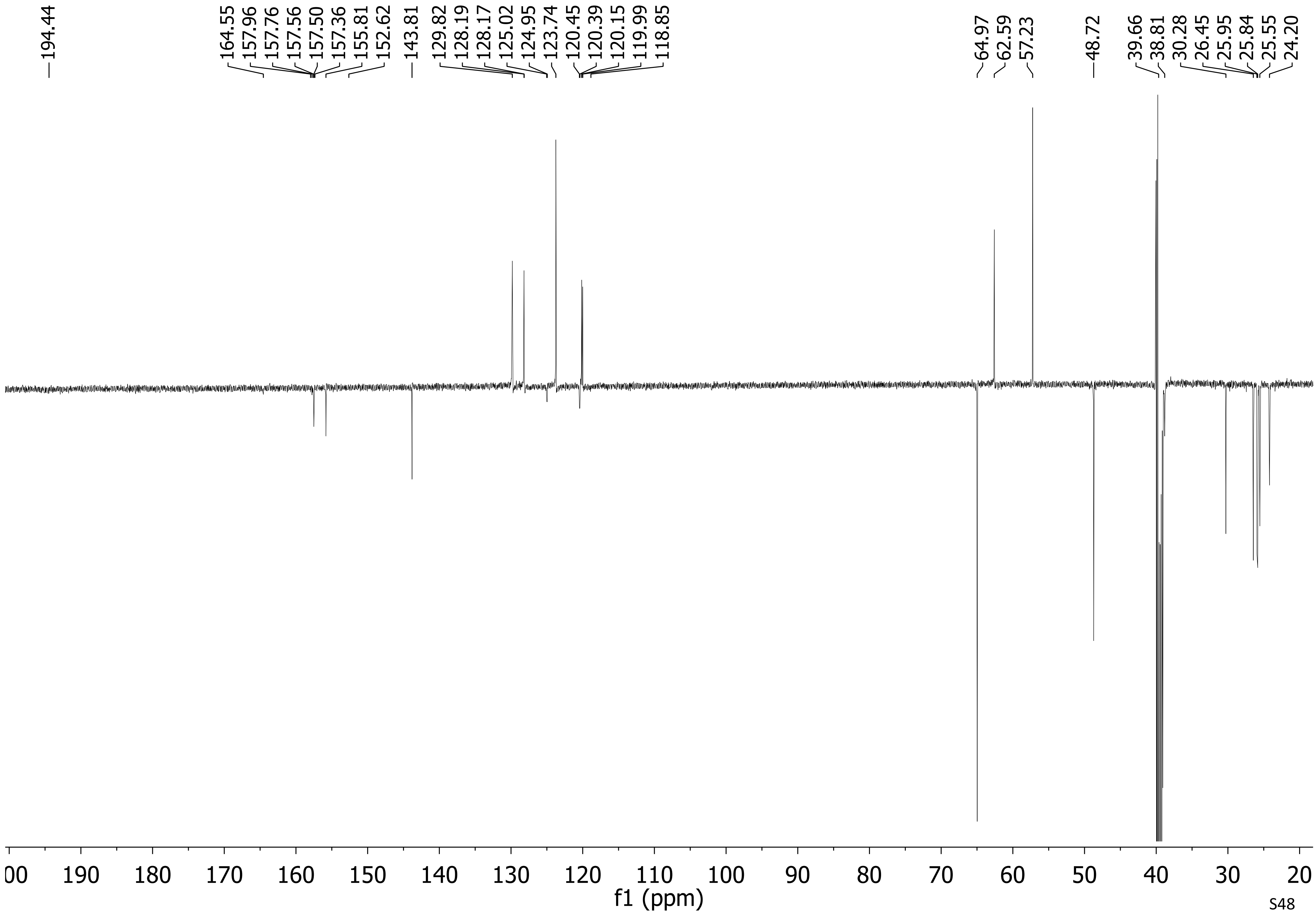

^{1}H NMR (600 MHz, DMSO-d_6) for compound **11**

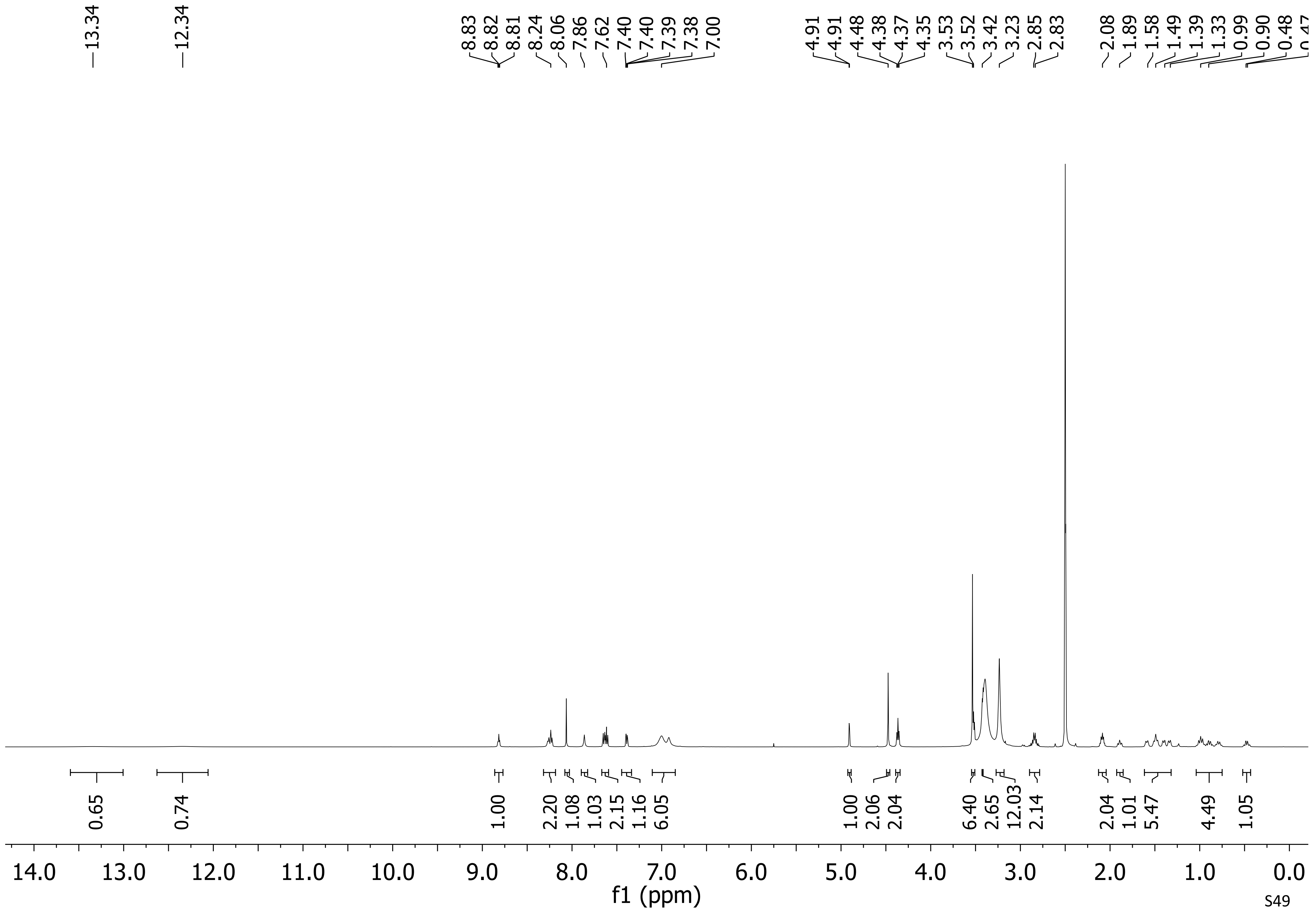

^{1}H NMR (600 MHz, DMSO-d_6) for compound **11** (zoomed-in view)

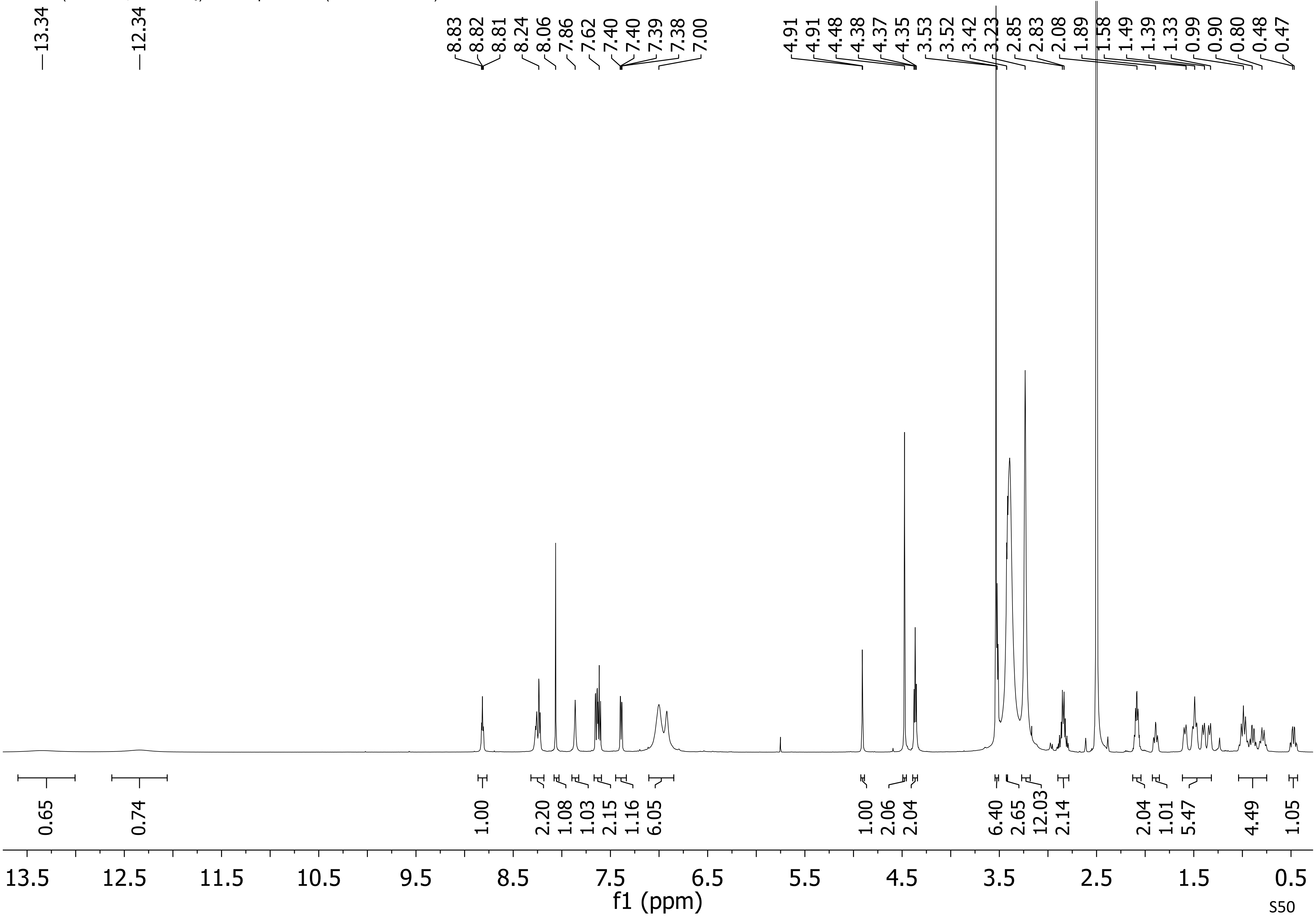

DEPTQ ^{13}C NMR (151 MHz, DMSO-d_6) for compound **11**

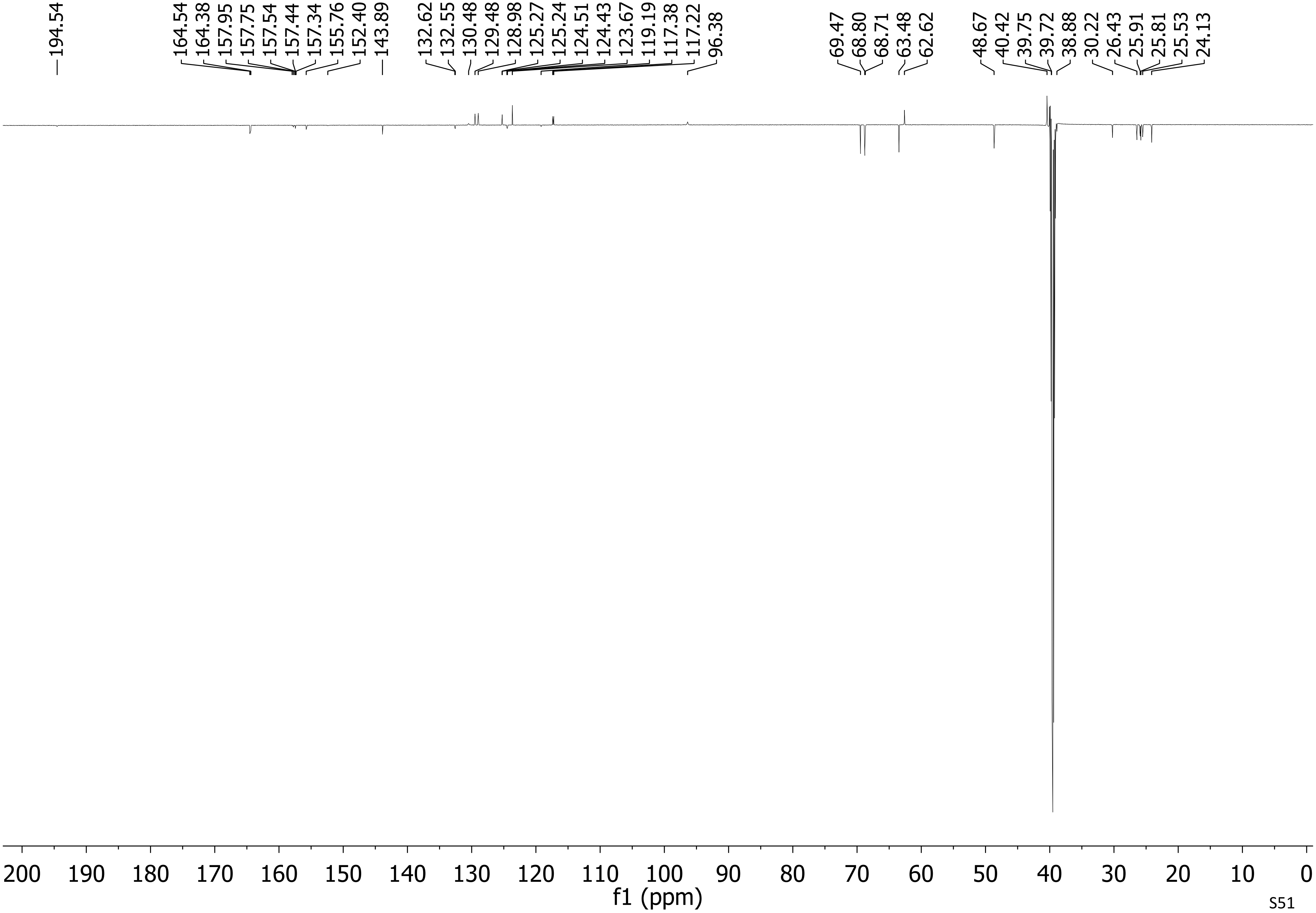

DEPTQ ^{13}C NMR (151 MHz, DMSO-d_6) for compound **11** (zoomed-in view)

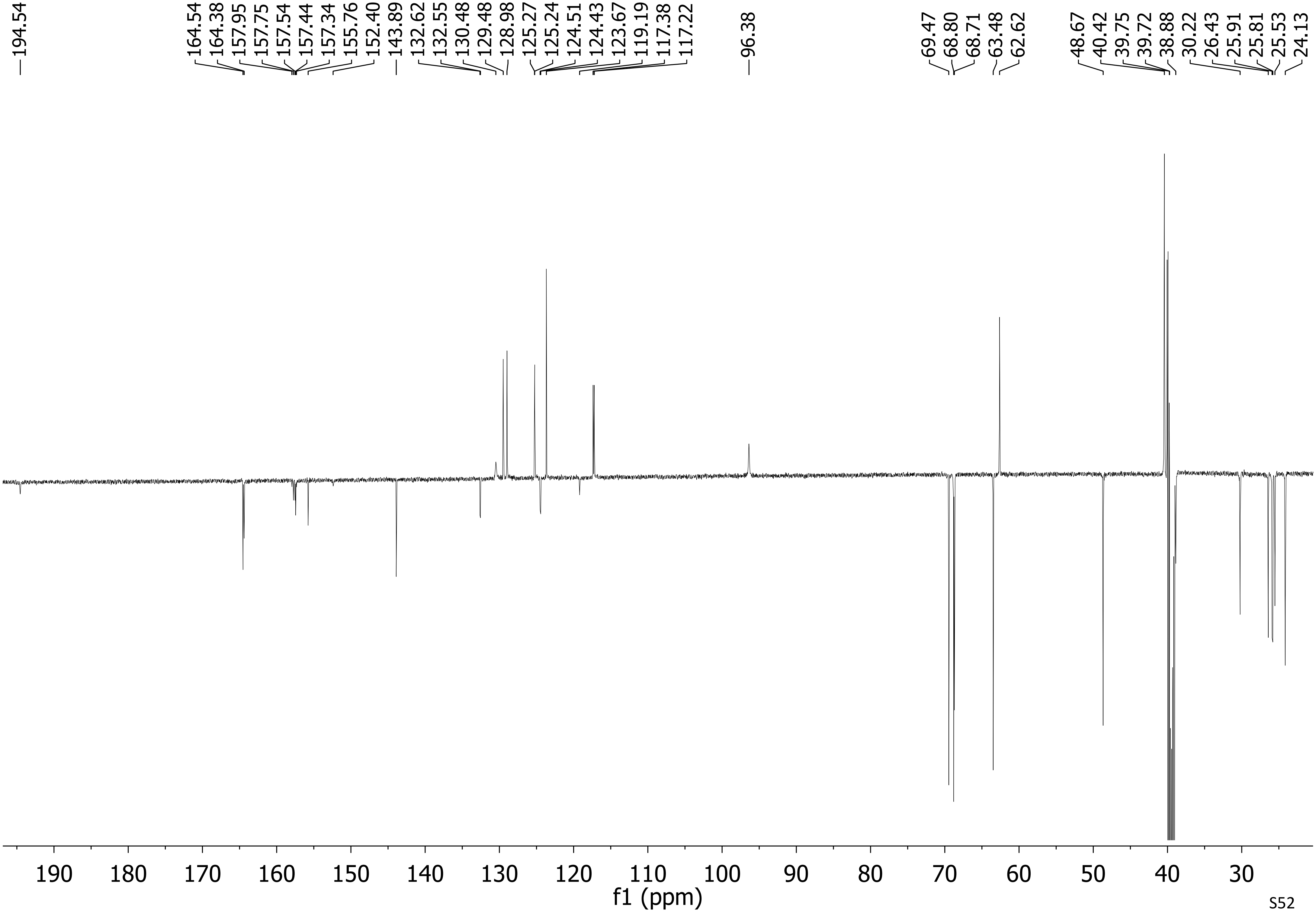

^{1}H NMR (600 MHz, DMSO-d_6) for compound **12**

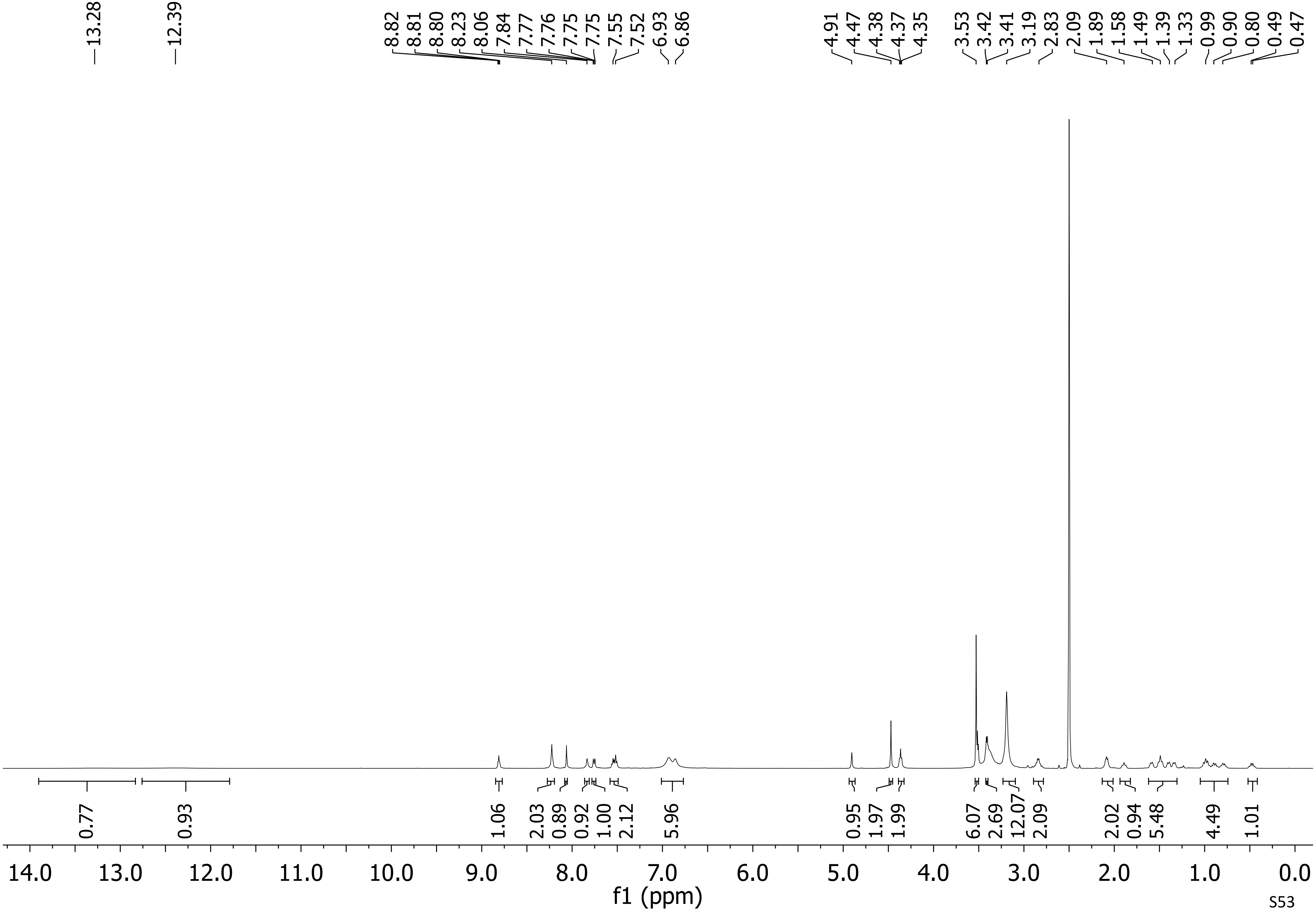

^{1}H NMR (600 MHz, DMSO-d_6) for compound **12** (zoomed-in view)

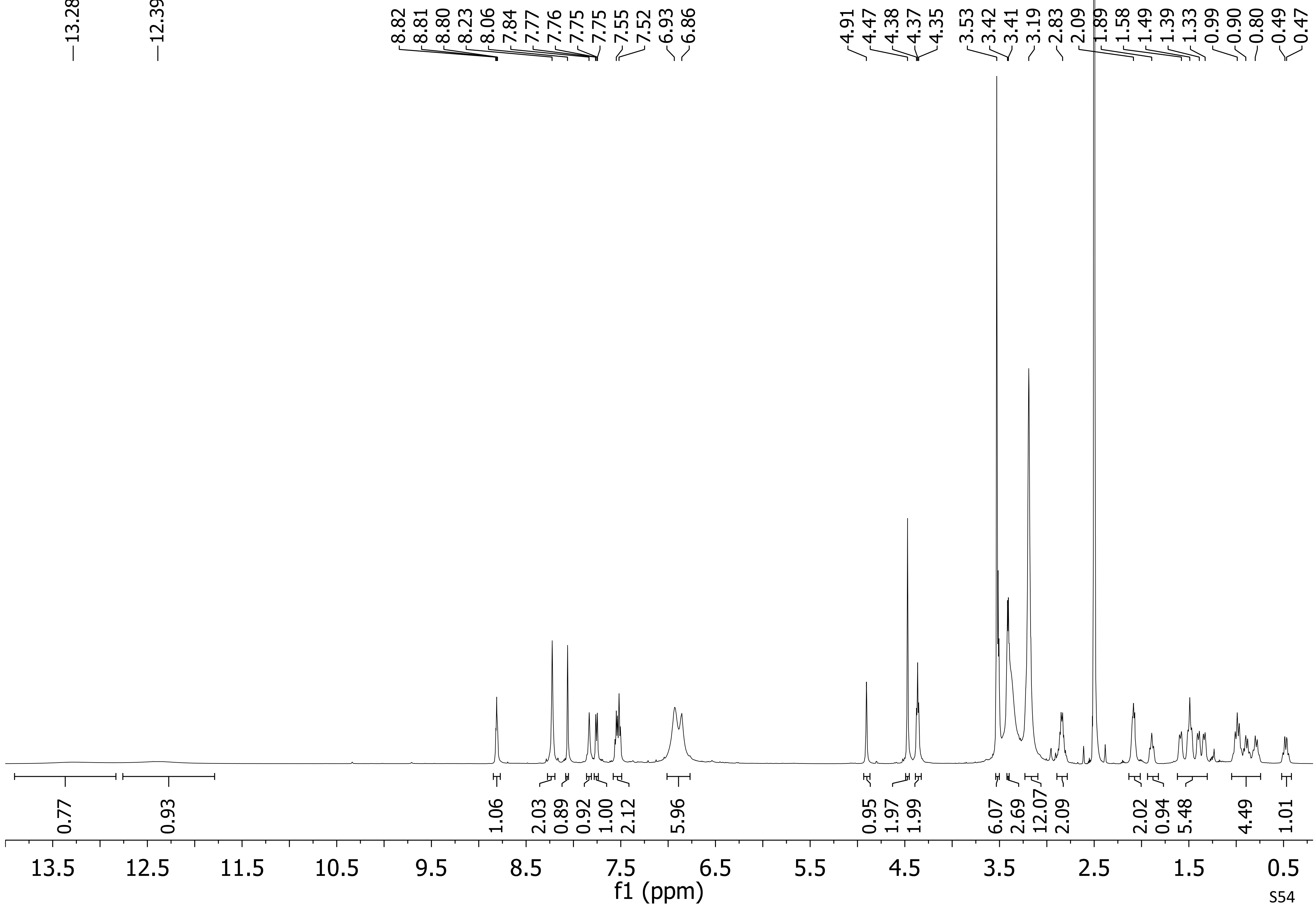

DEPTQ ^{13}C NMR (151 MHz, DMSO-d_6) for compound **12**

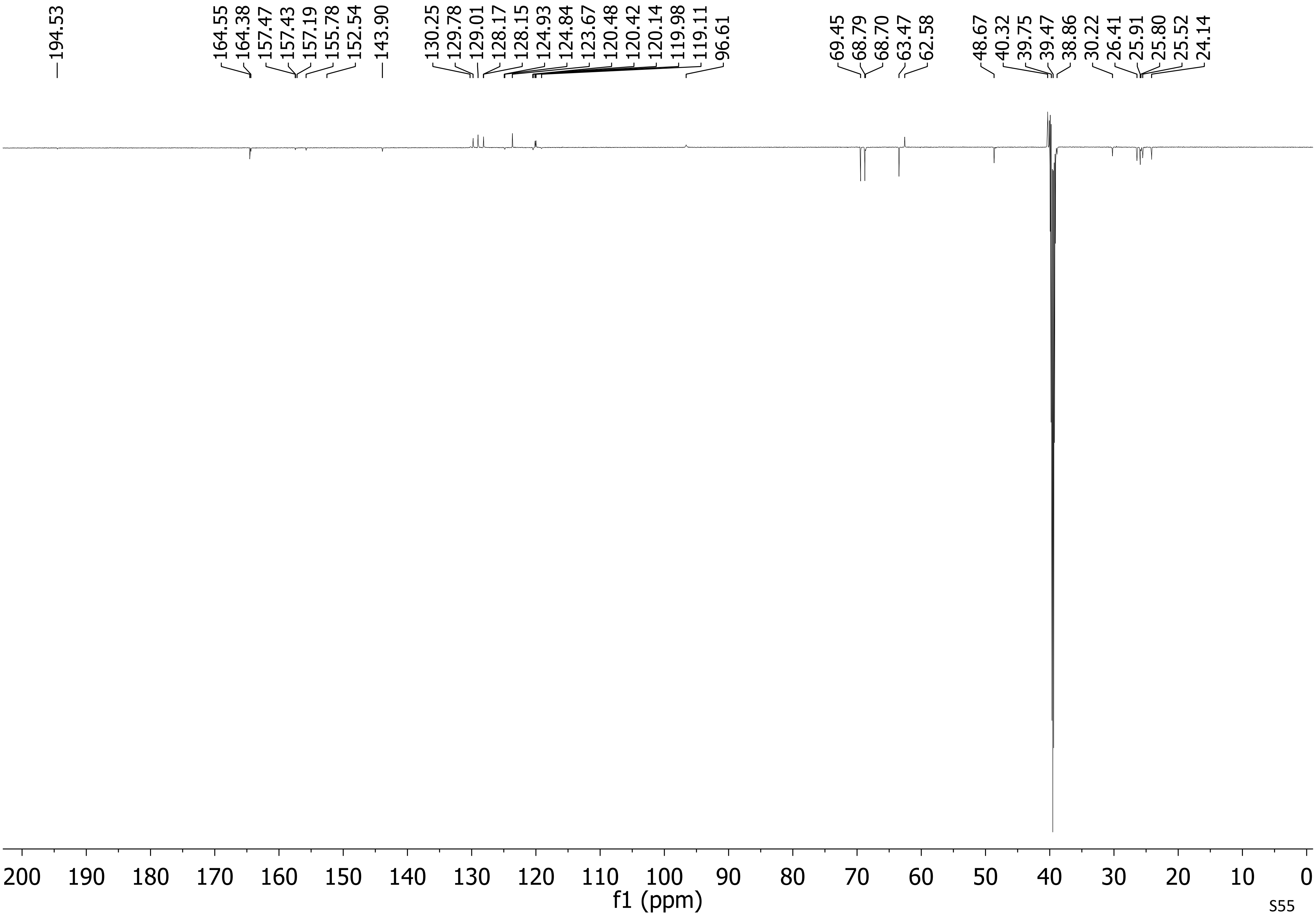

DEPTQ ^{13}C NMR (151 MHz, DMSO-d_6) for compound **12** (zoomed-in view)

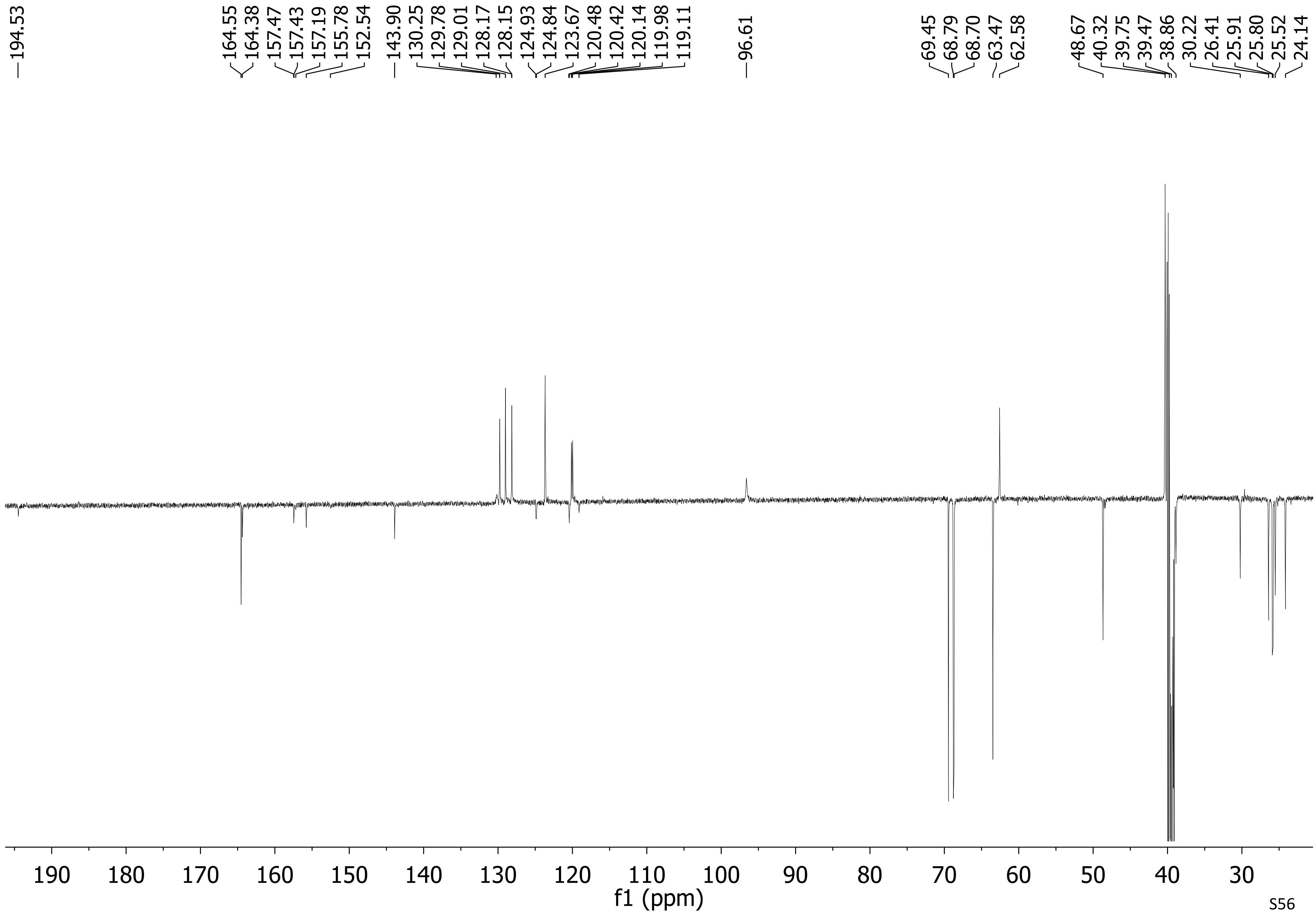

^{1}H NMR (400 MHz, DMSO-d_6) for compound **15**

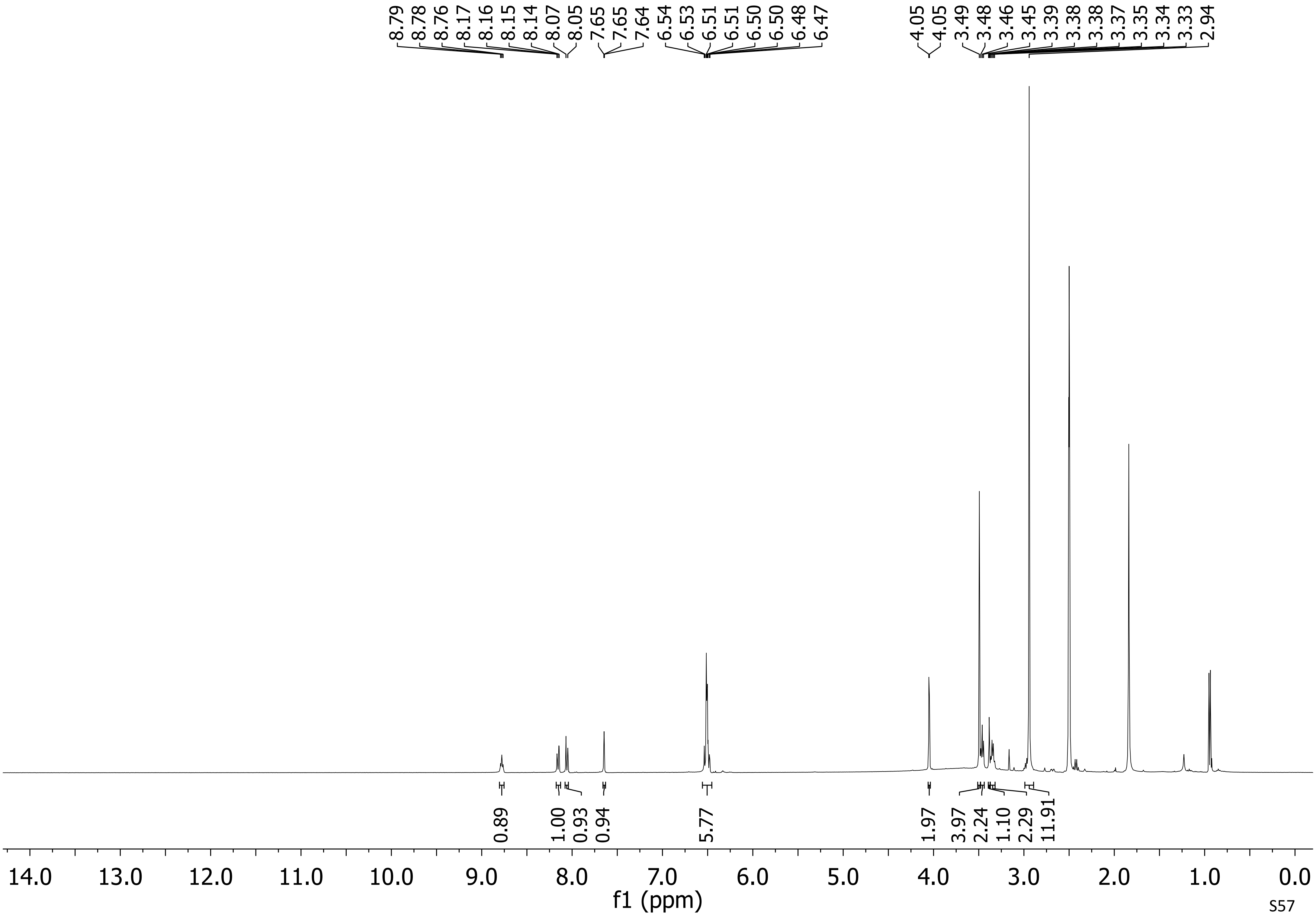

DEPTQ ^{13}C NMR (151 MHz, DMSO-d_6) for compound **15**

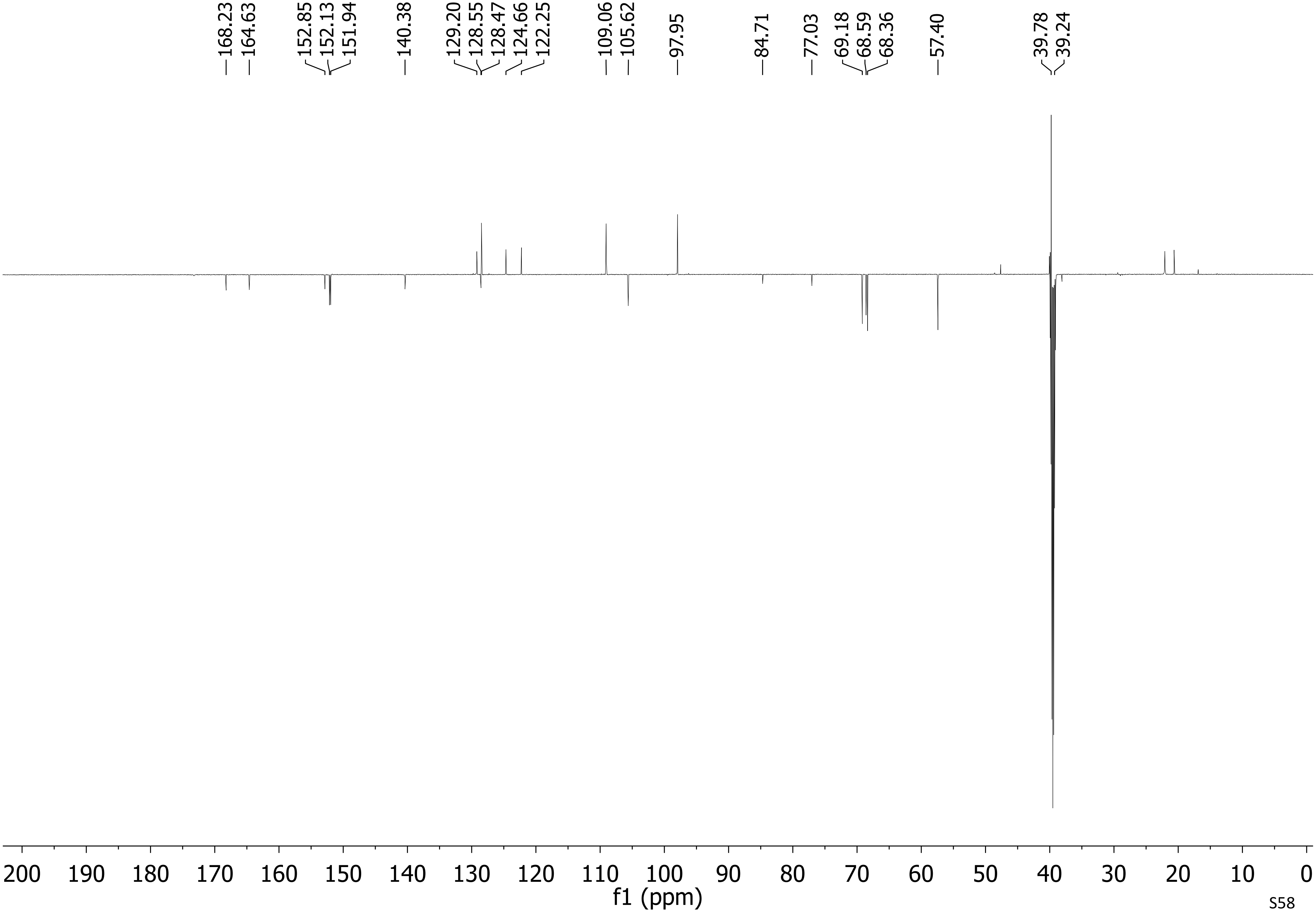

^{1}H NMR (400 MHz, DMSO-d_6) for compound **23**

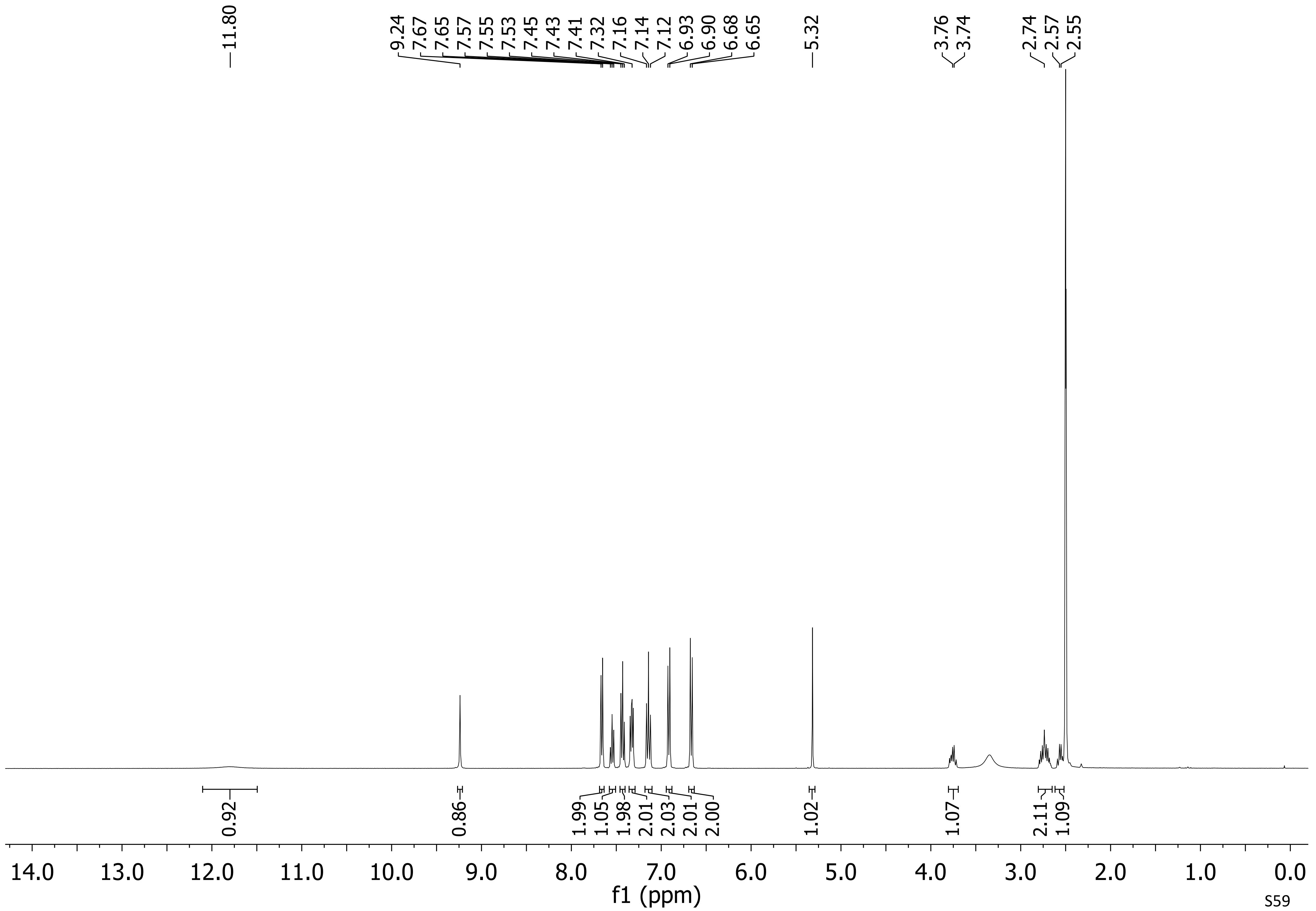

DEPTQ ^{13}C NMR (101 MHz, DMSO-d_6) for compound **23**

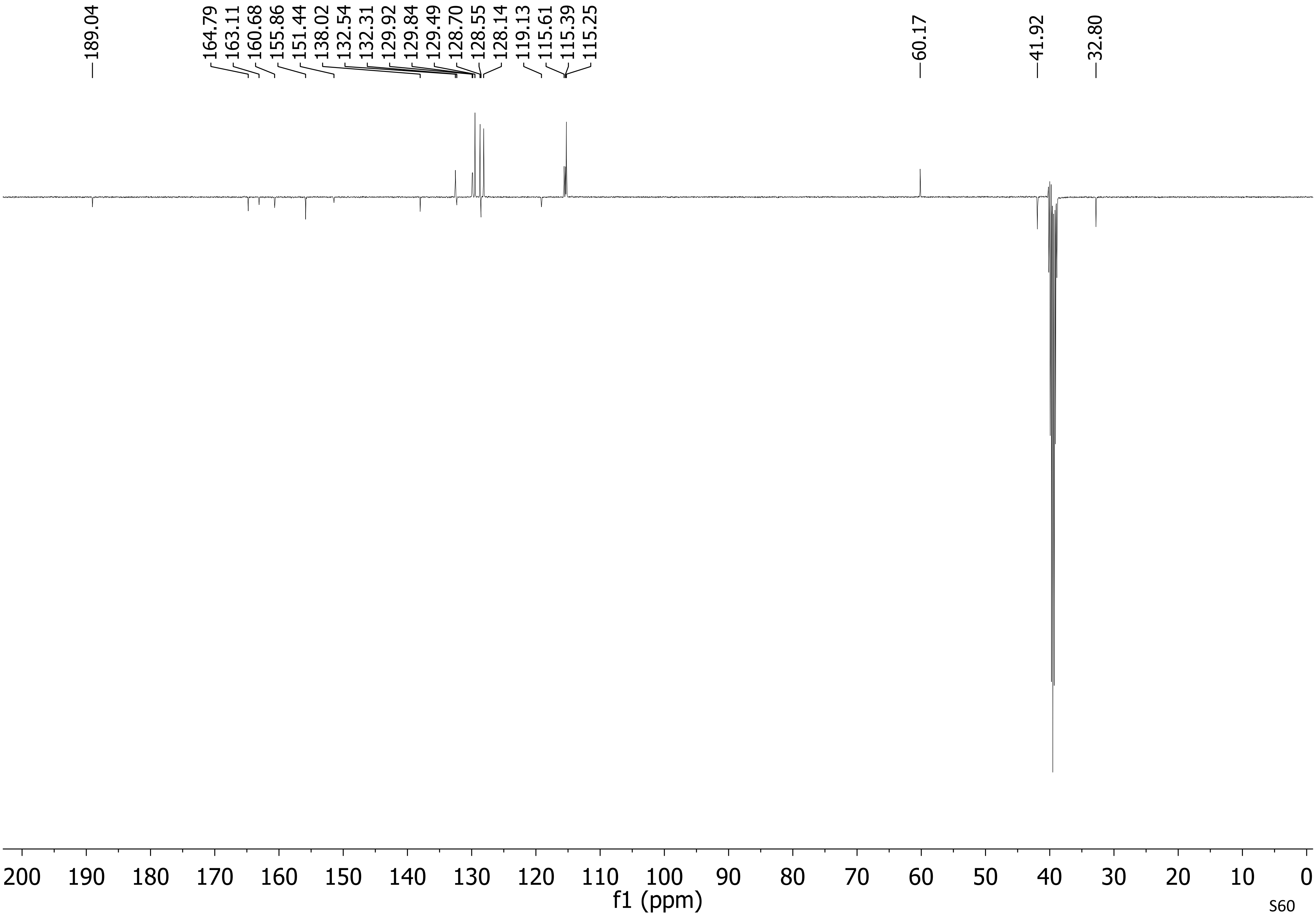

^{1}H NMR (400 MHz, DMSO-d_6) for compound **24**

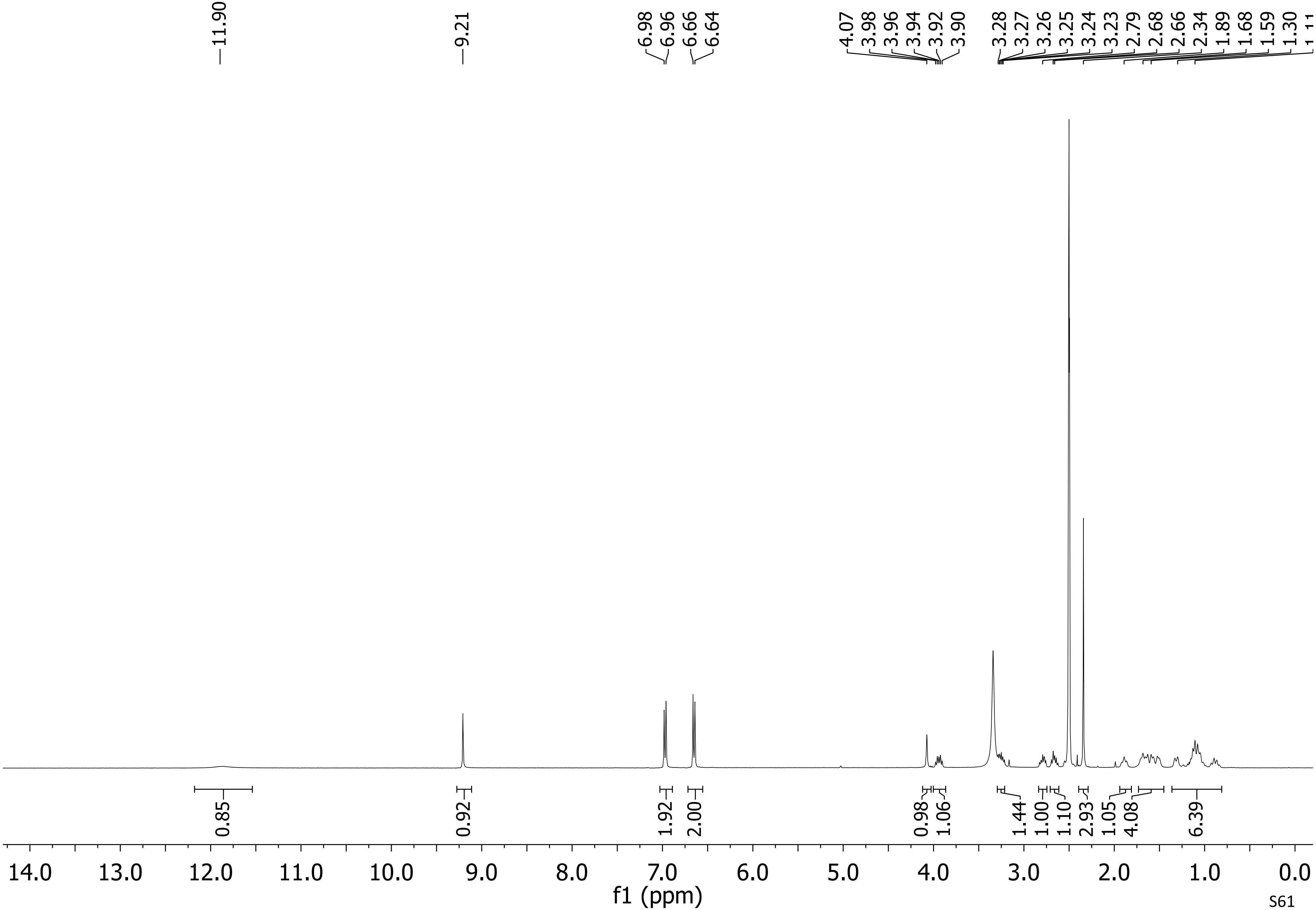

DEPTQ ^{13}C NMR (101 MHz, DMSO-d_6) for compound **24**

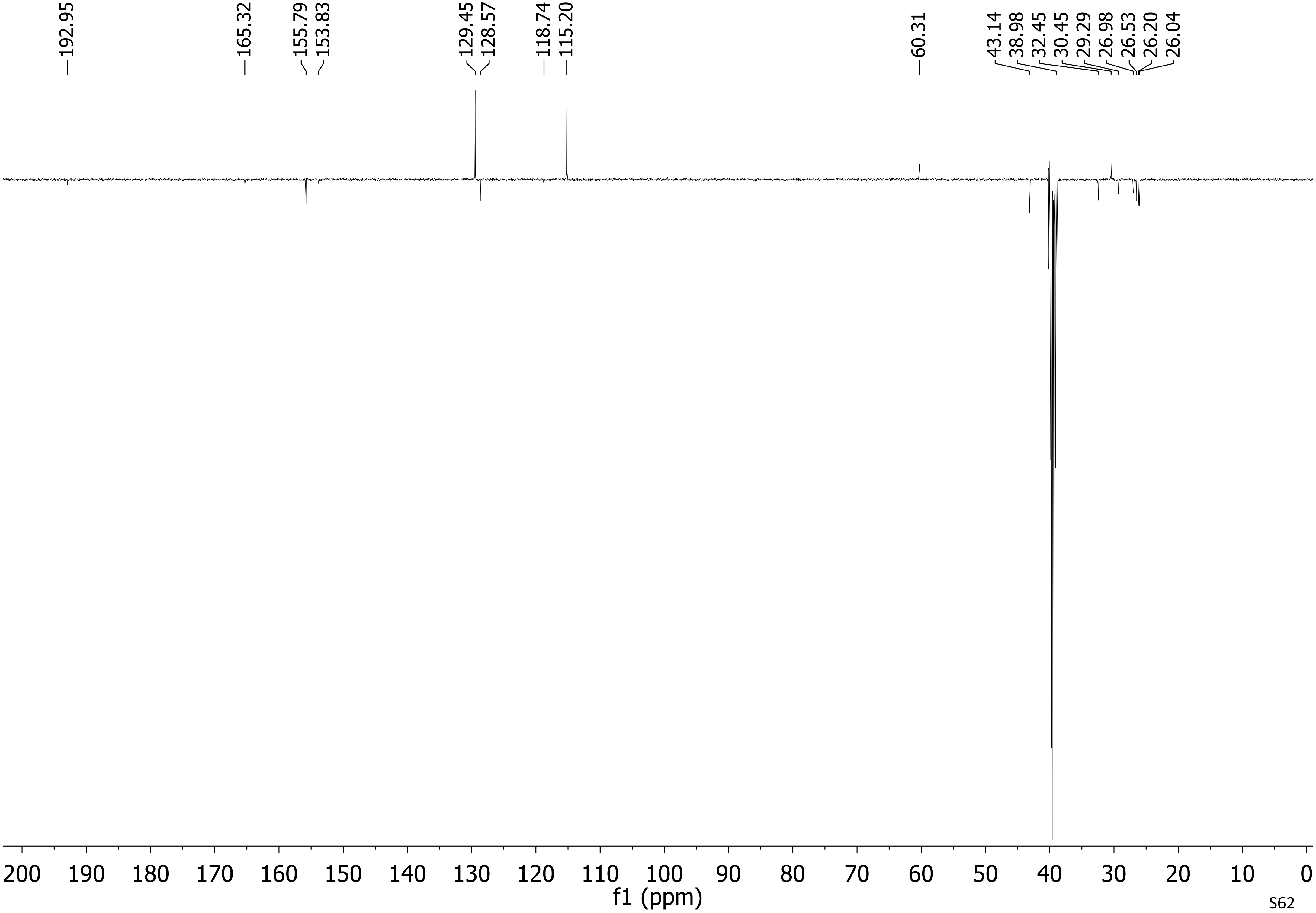

^{1}H NMR (400 MHz, DMSO-d_6) for compound **25**

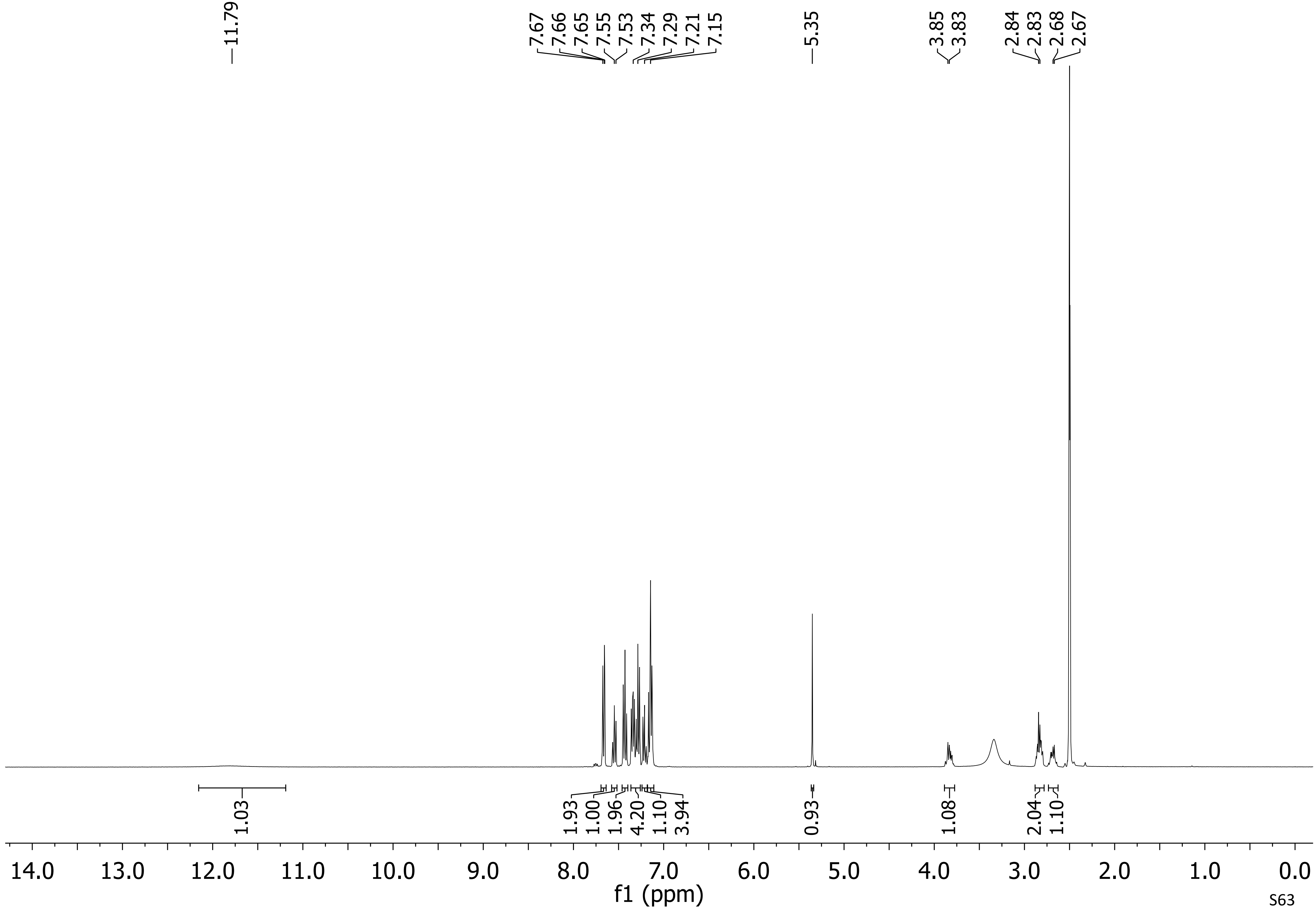

DEPTQ ^{13}C NMR (101 MHz, DMSO-d_6) for compound **25**

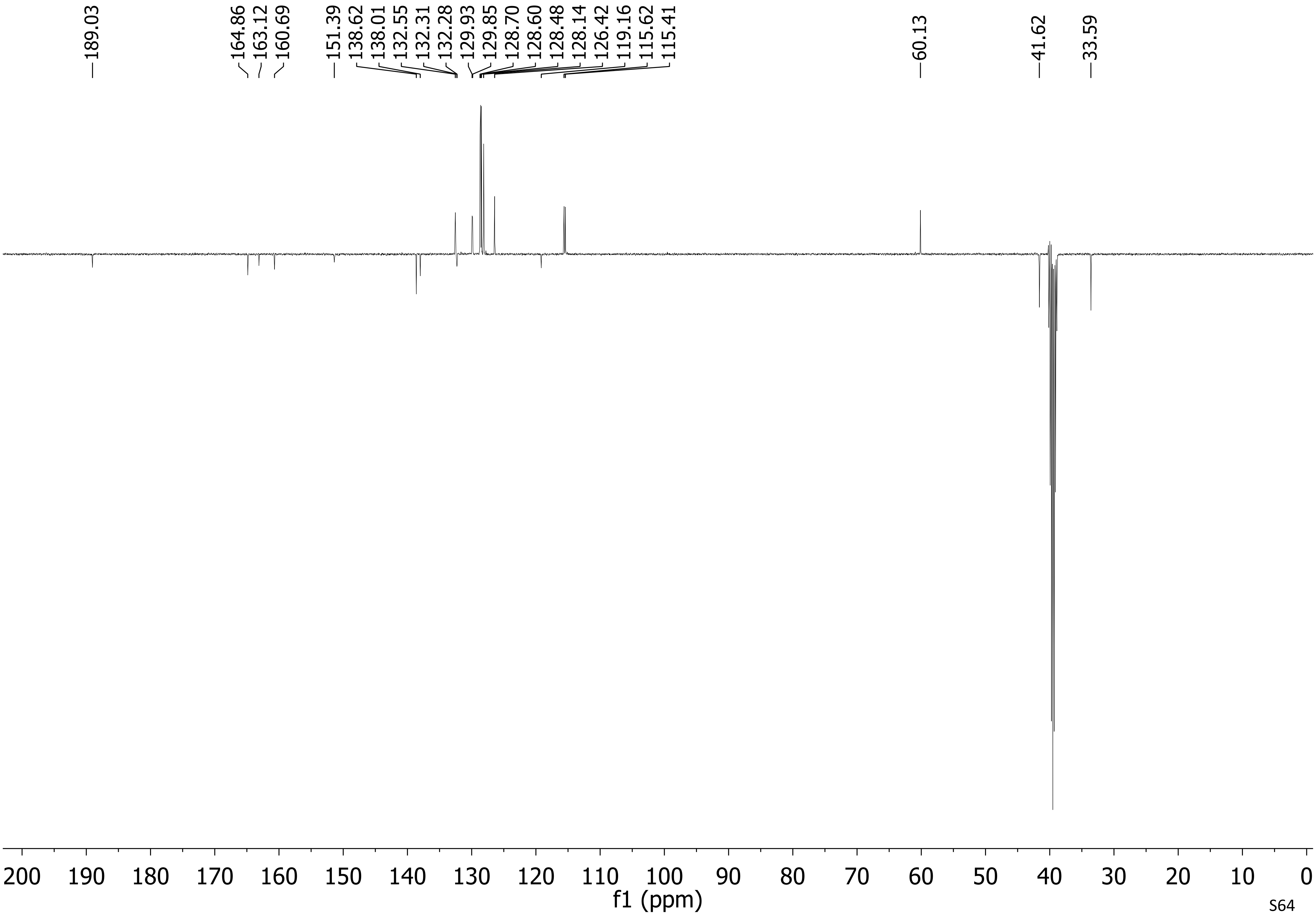

^{1}H NMR (400 MHz, DMSO-d_6) for compound **26**

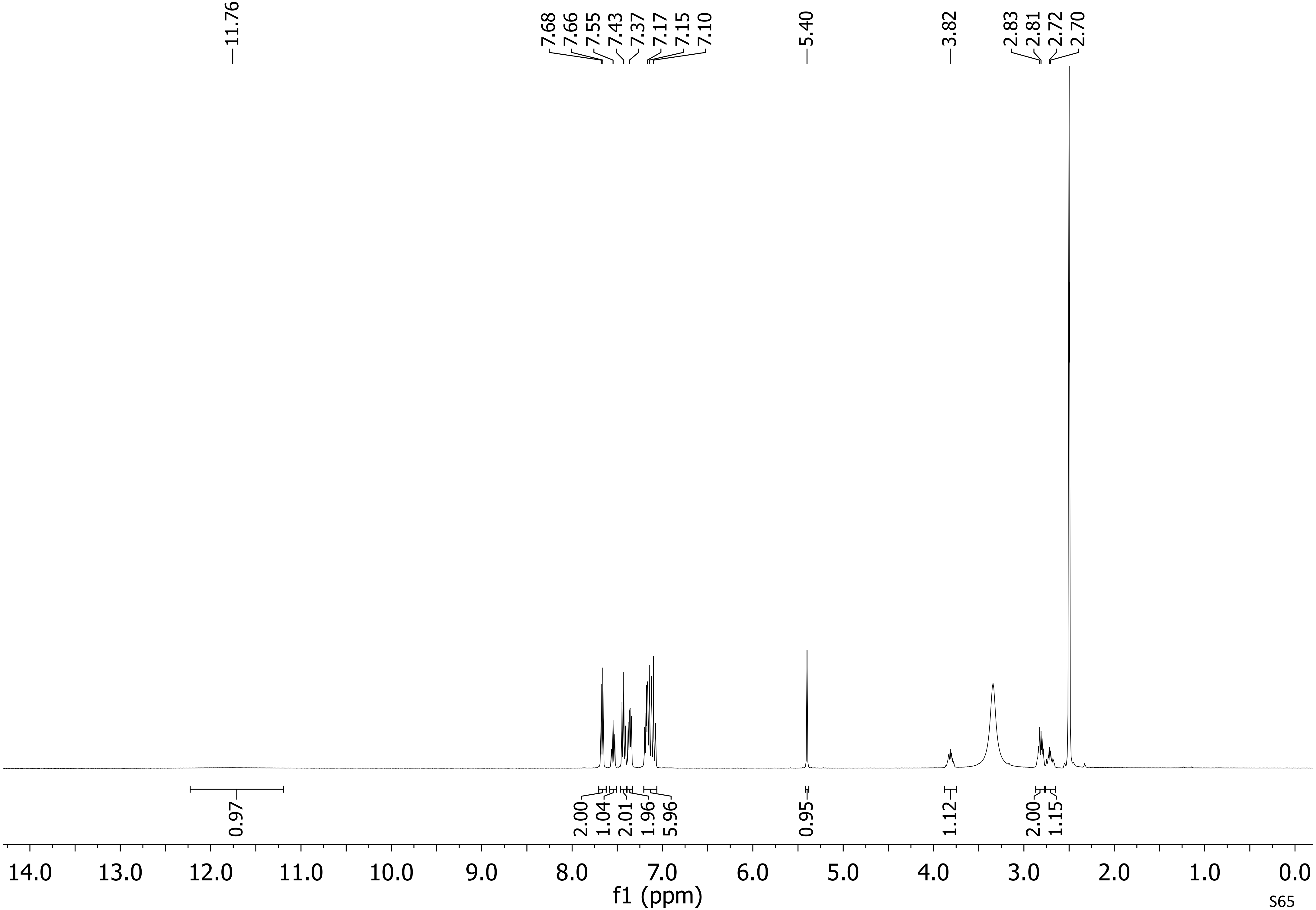

DEPTQ ^{13}C NMR (151 MHz, DMSO-d_6) for compound **26**

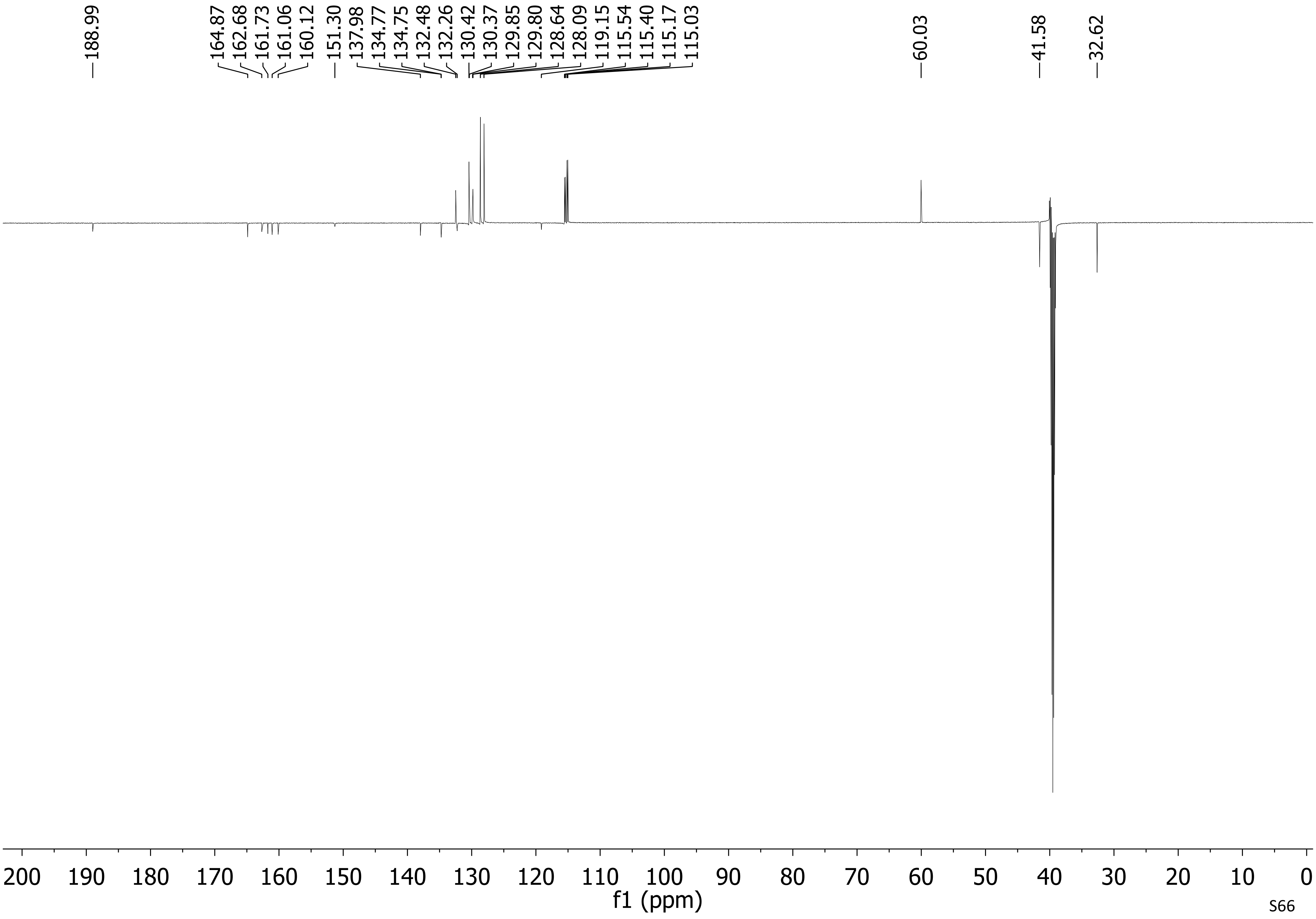

^{1}H NMR (400 MHz, DMSO-d_6) for compound **27**

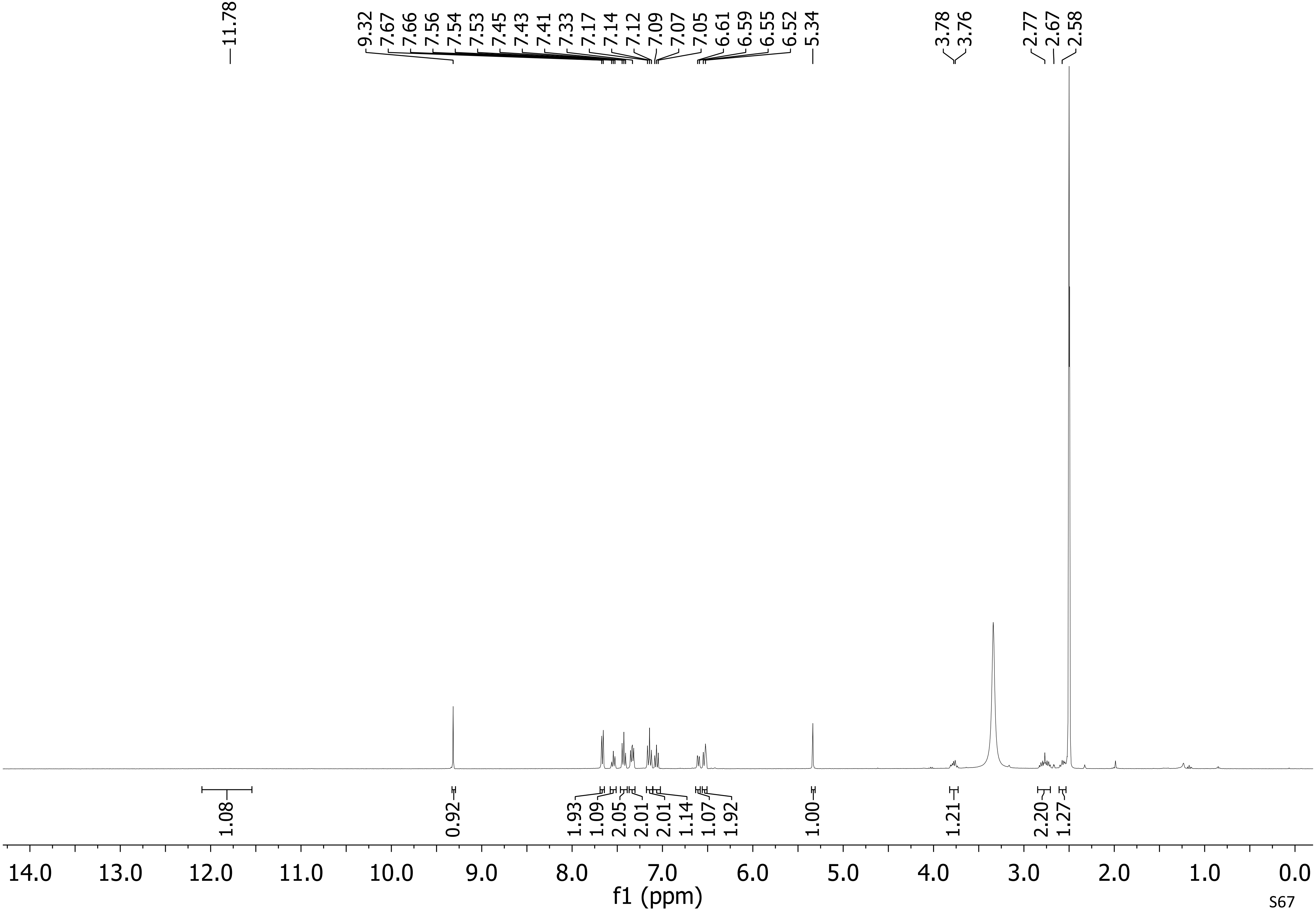

DEPTQ ^{13}C NMR (101 MHz, DMSO-d_6) for compound **27**

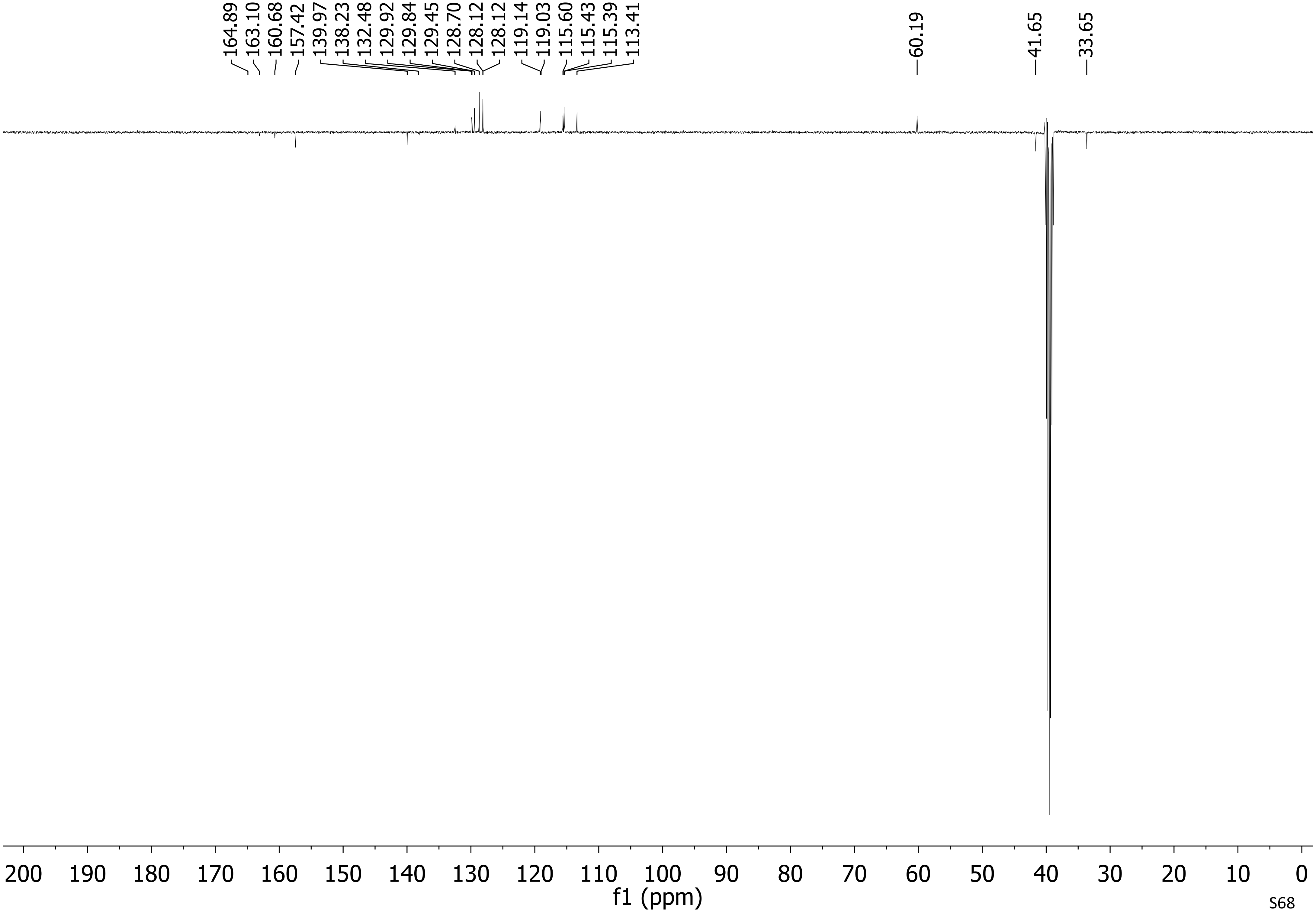

DEPTQ ^{13}C NMR (101 MHz, DMSO-d_6) for compound **27** (zoomed-in view)

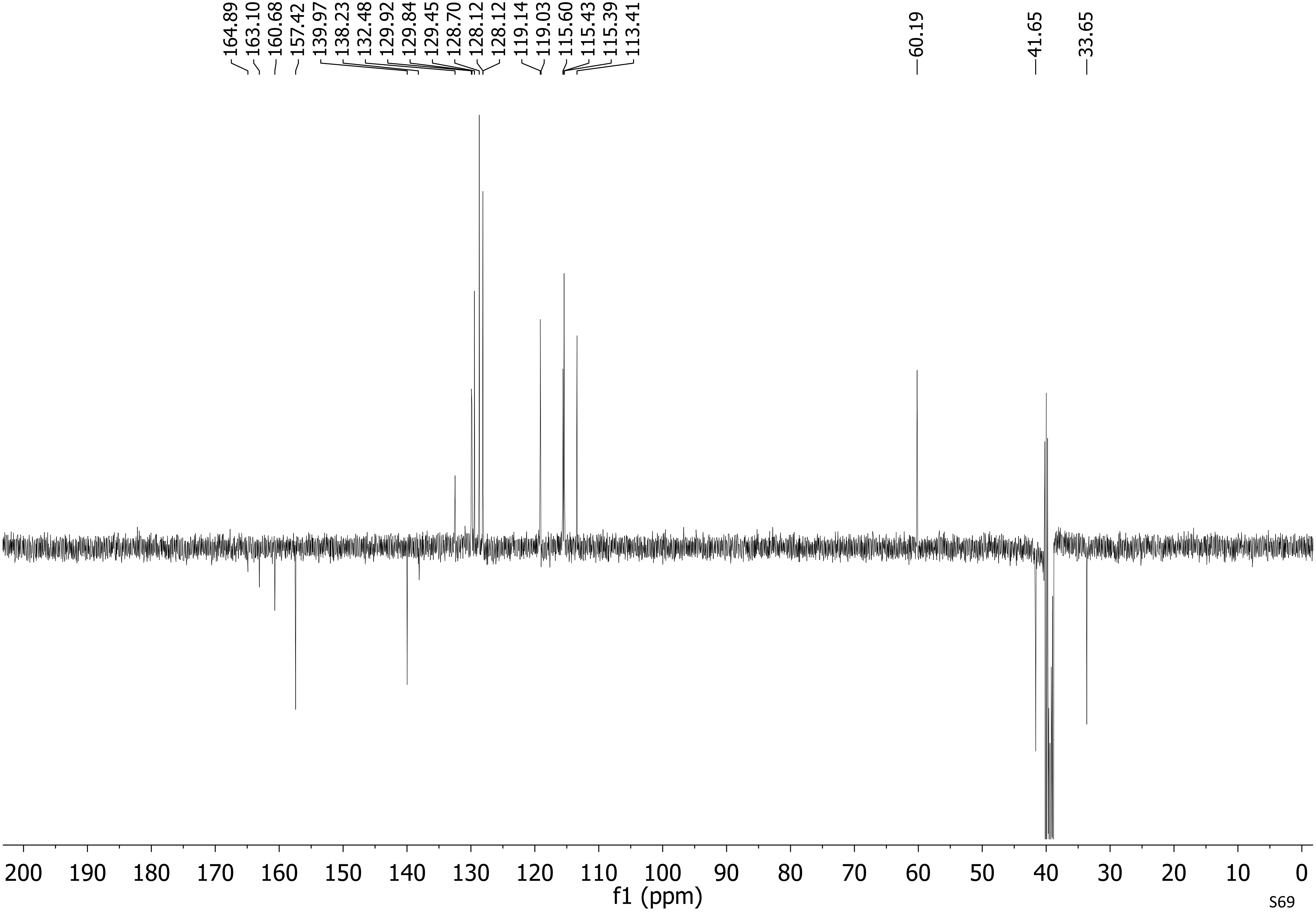

^{1}H NMR (500 MHz, DMSO-d_6) for compound **28**

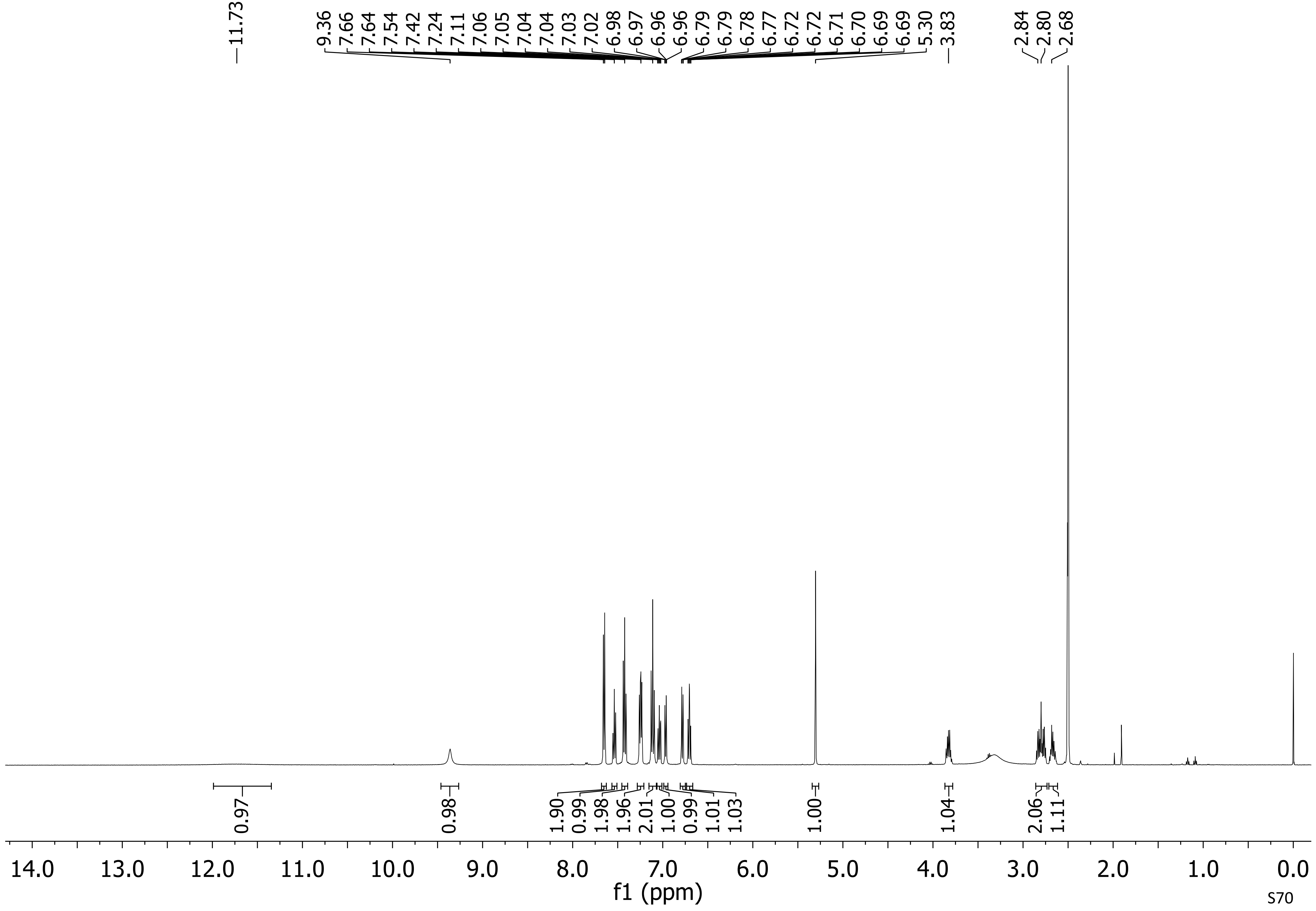

^{13}C NMR (126 MHz, DMSO-d_6) for compound **28**

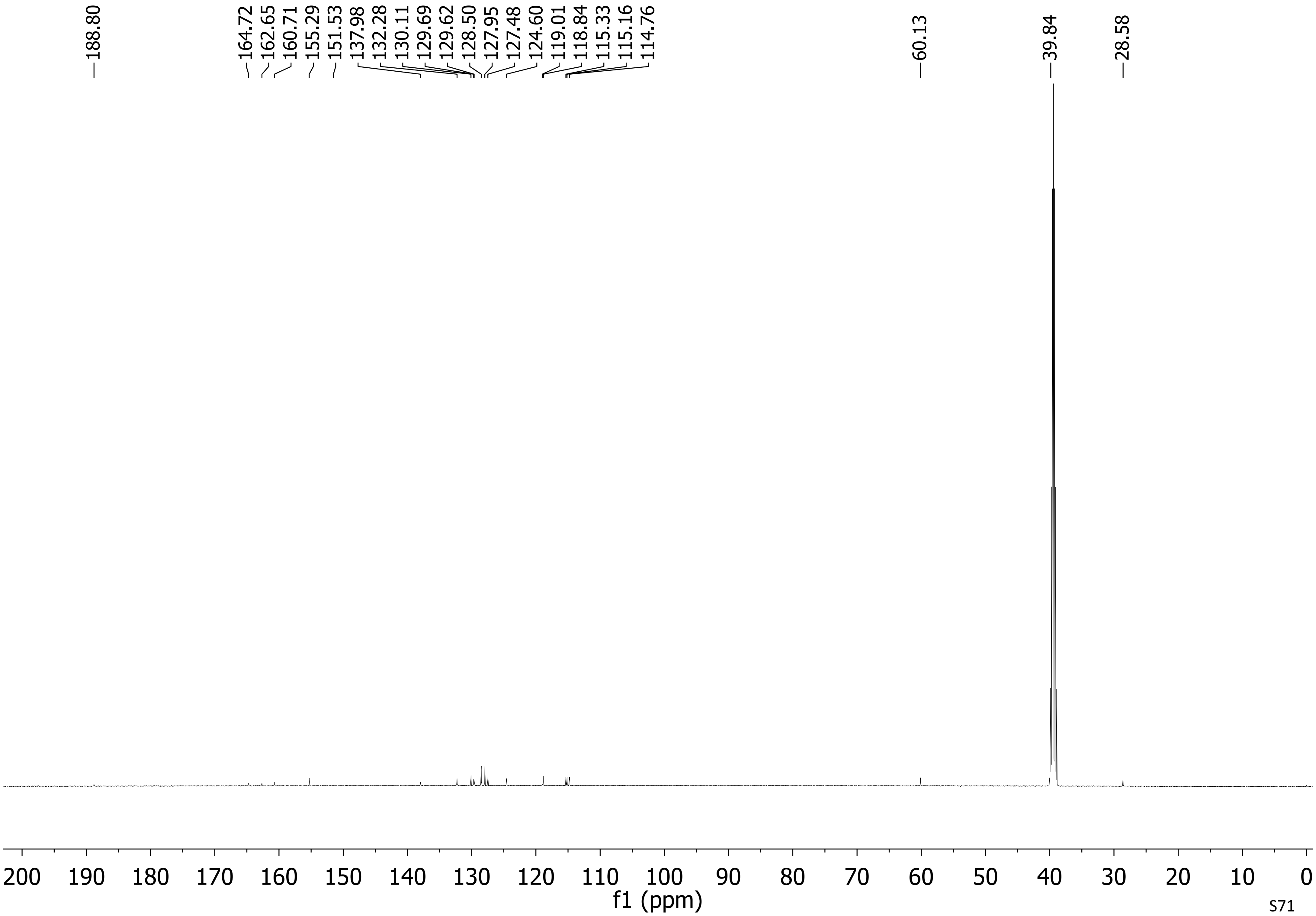

^{13}C NMR (126 MHz, DMSO-d_6) for compound **28** (zoomed-in view)

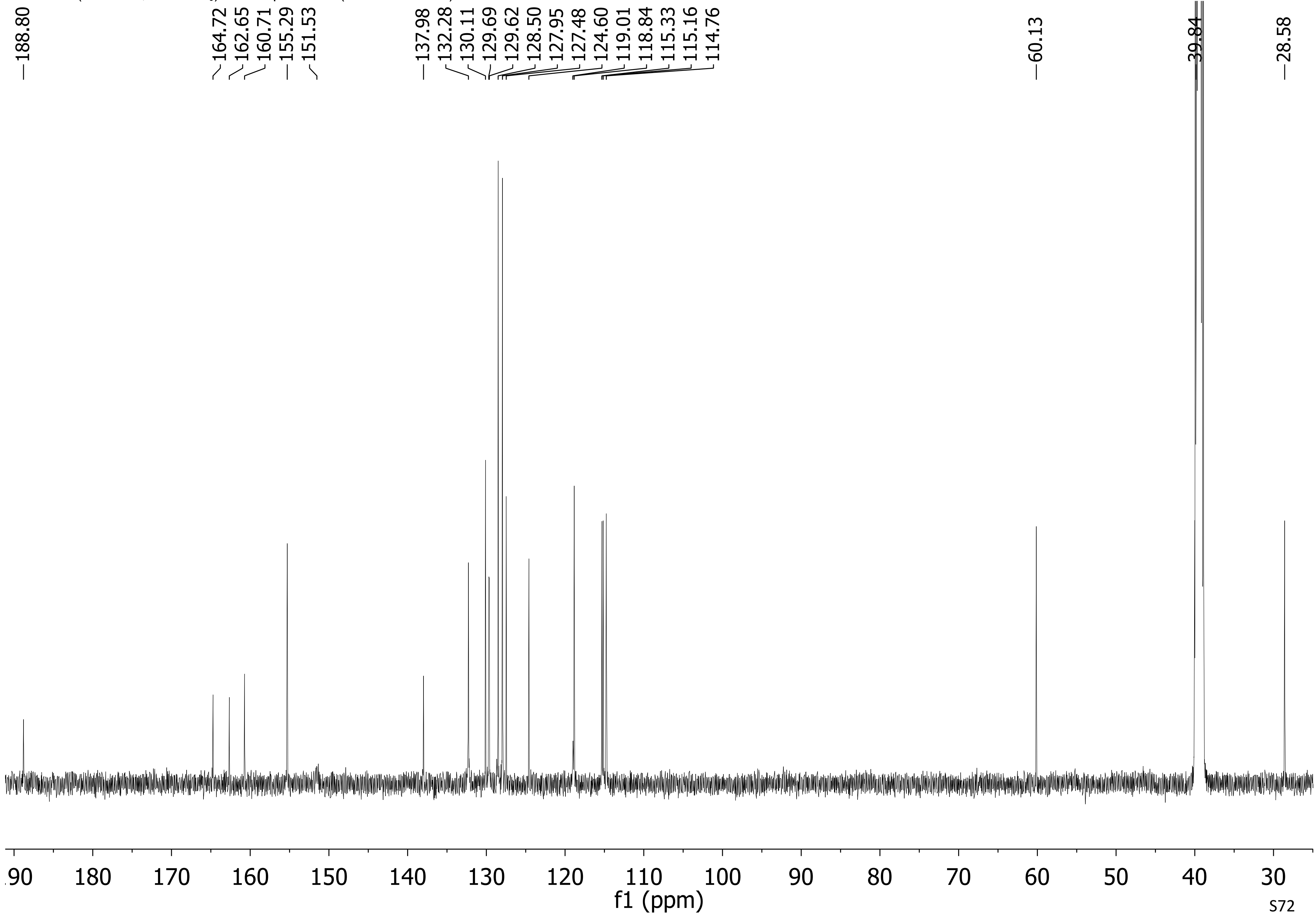

^{1}H NMR (400 MHz, DMSO-d_6) for compound **29**

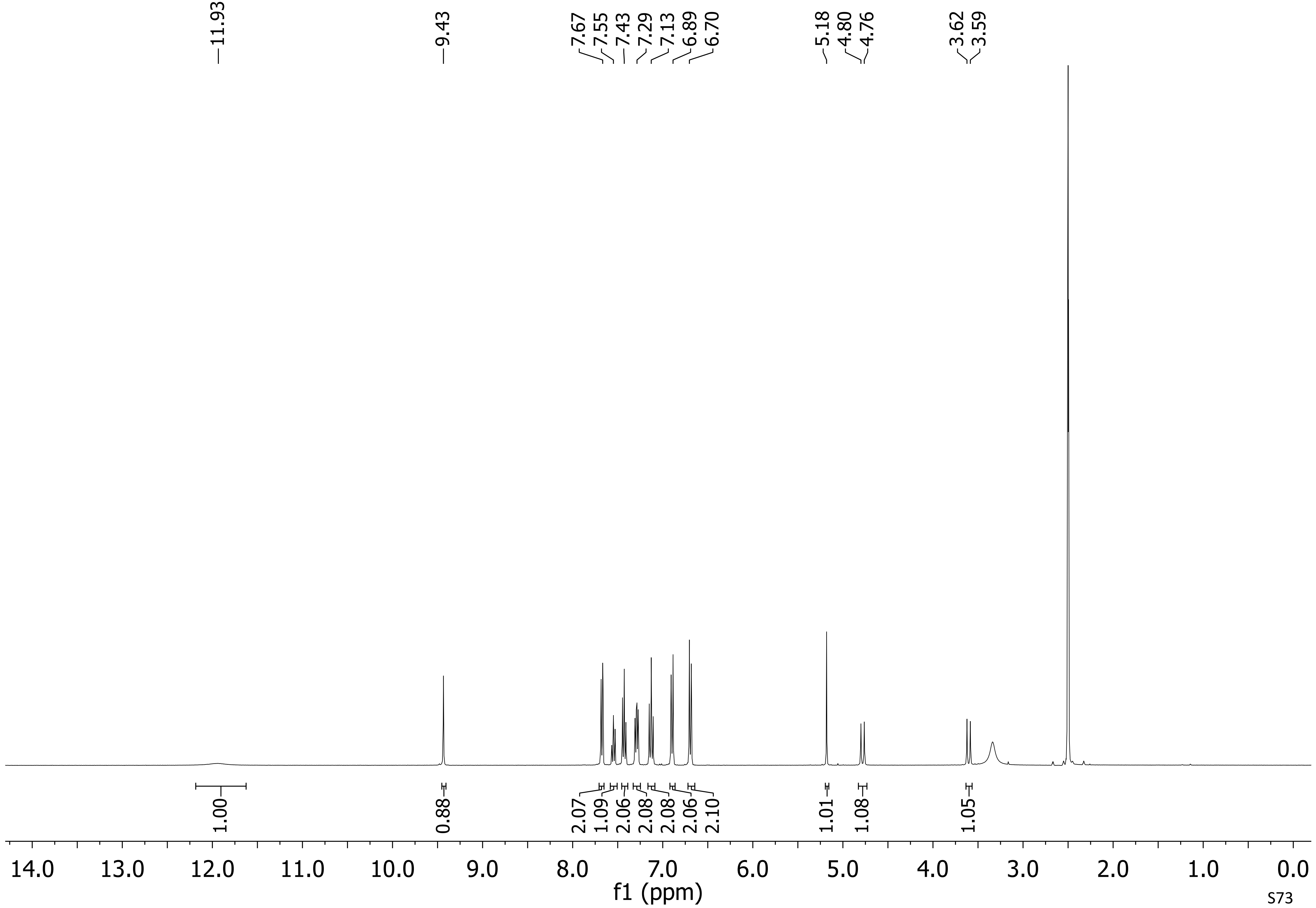

DEPTQ ^{13}C NMR (151 MHz, DMSO-d_6) for compound **29**

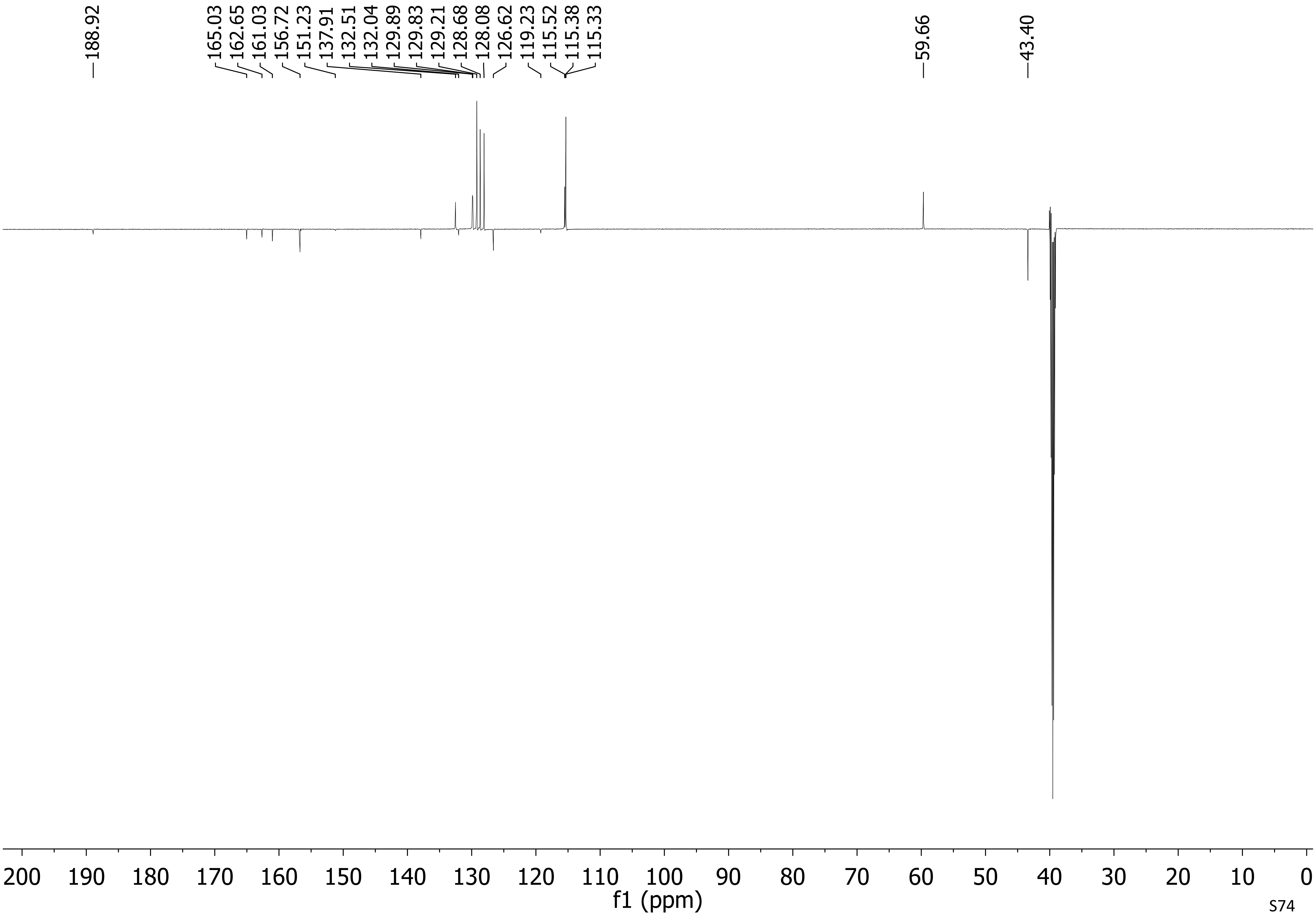

^{1}H NMR (400 MHz, DMSO-d_6) for compound **30**

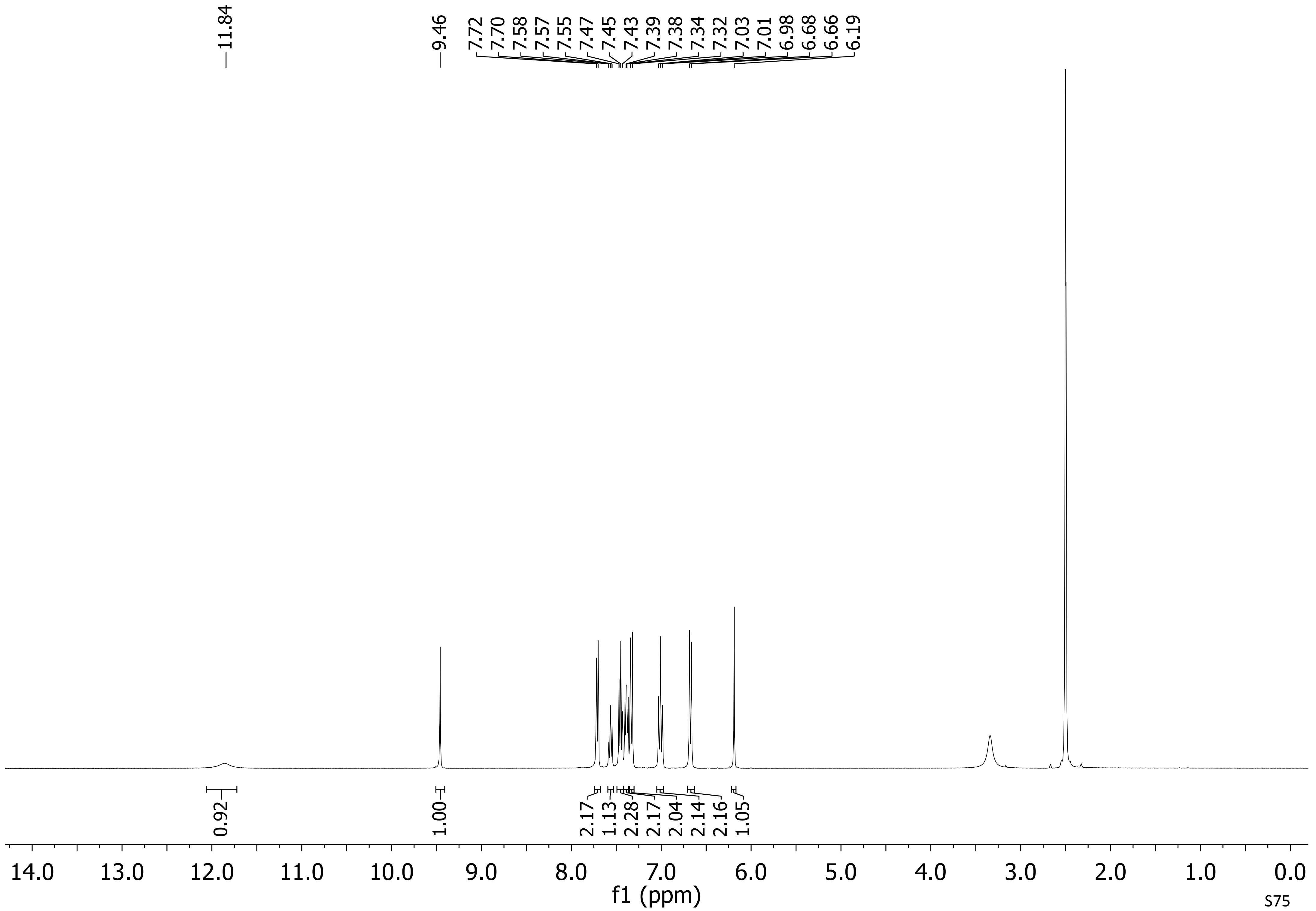

DEPTQ ^{13}C NMR (101 MHz, DMSO-d_6) for compound **30**

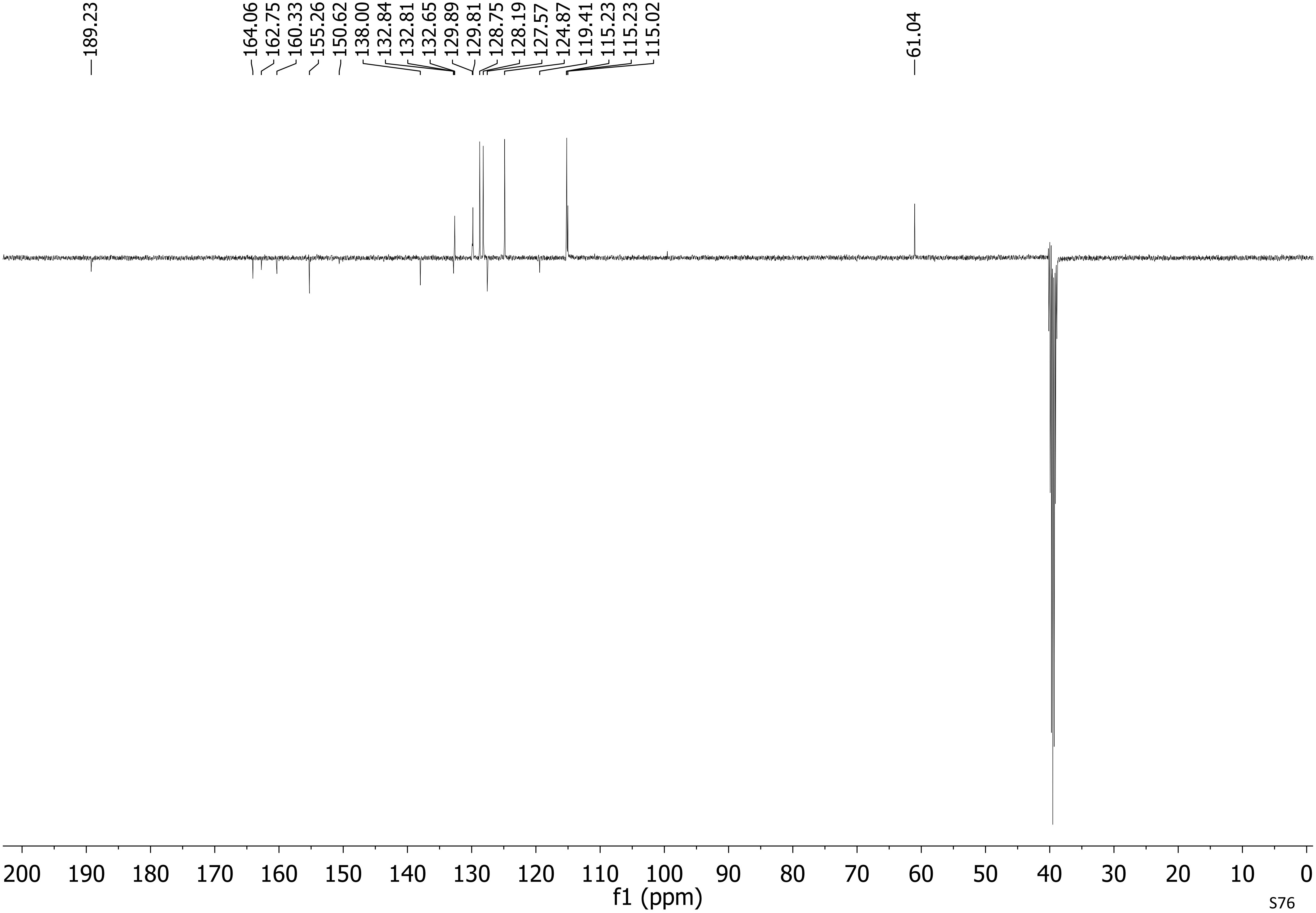

^{1}H NMR (400 MHz, DMSO-d_6) for compound **31**

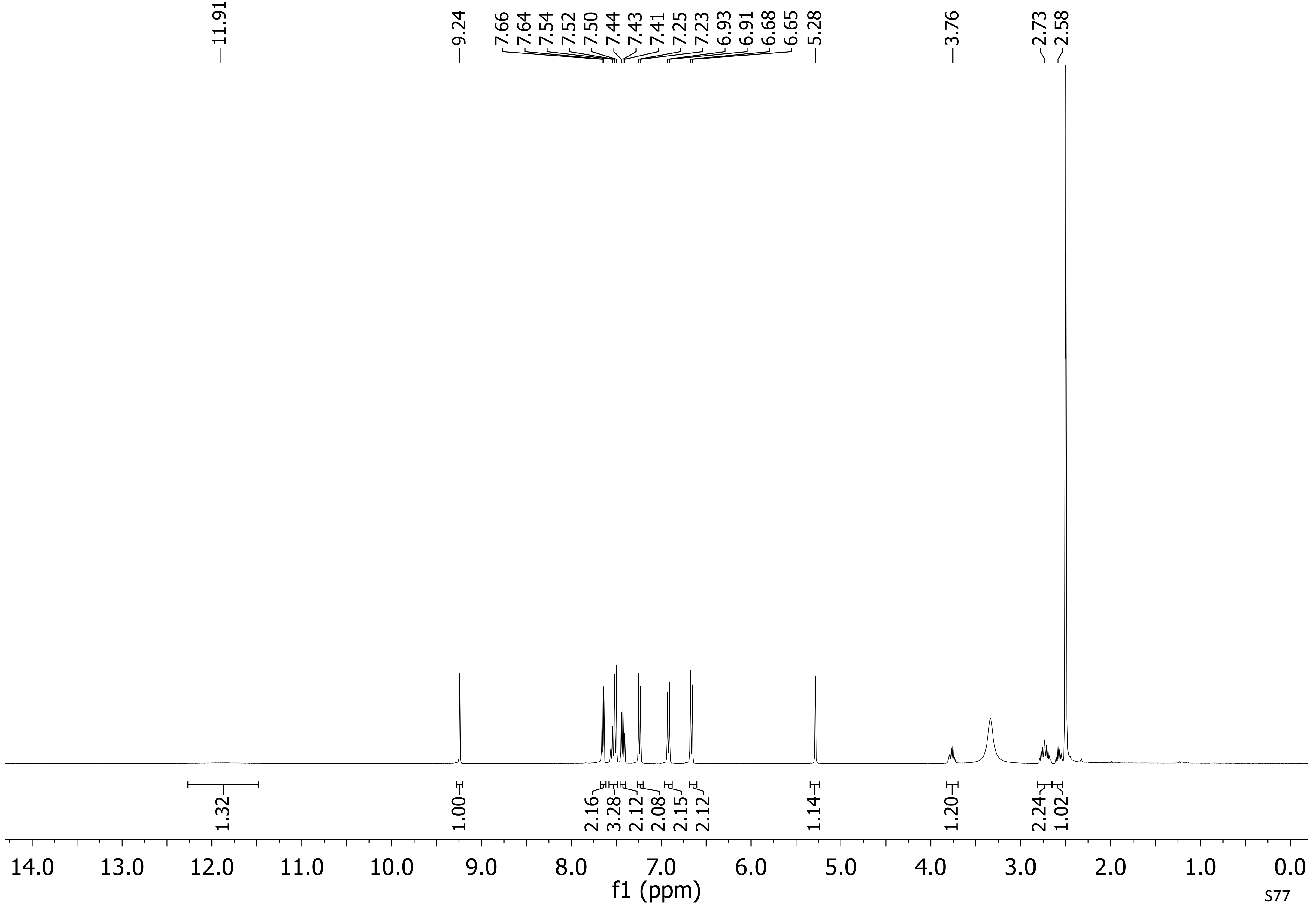

DEPTQ ^{13}C NMR (151 MHz, DMSO-d_6) for compound **31**

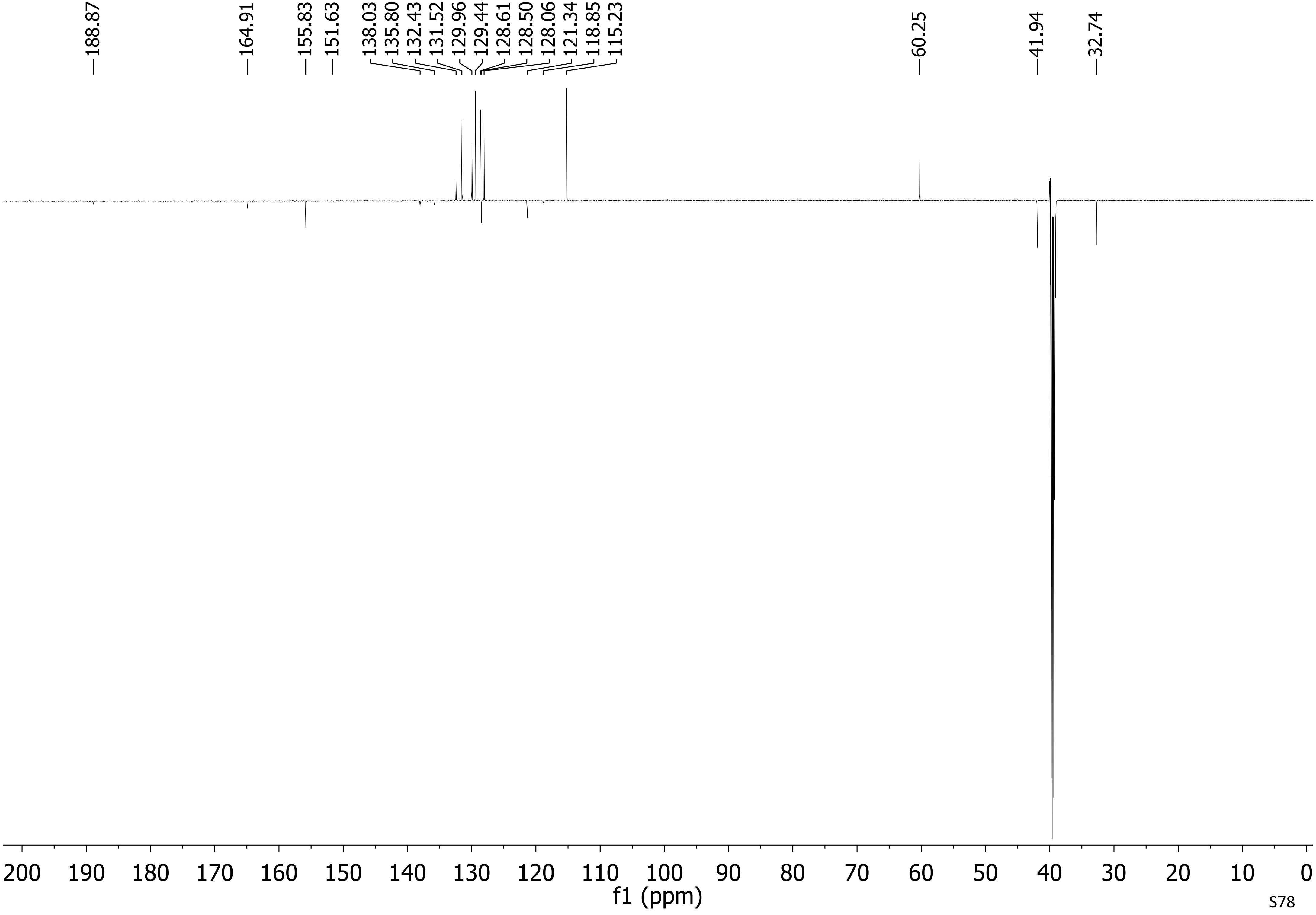

^1H NMR (400 MHz, DMSO-d_6) for compound **32**

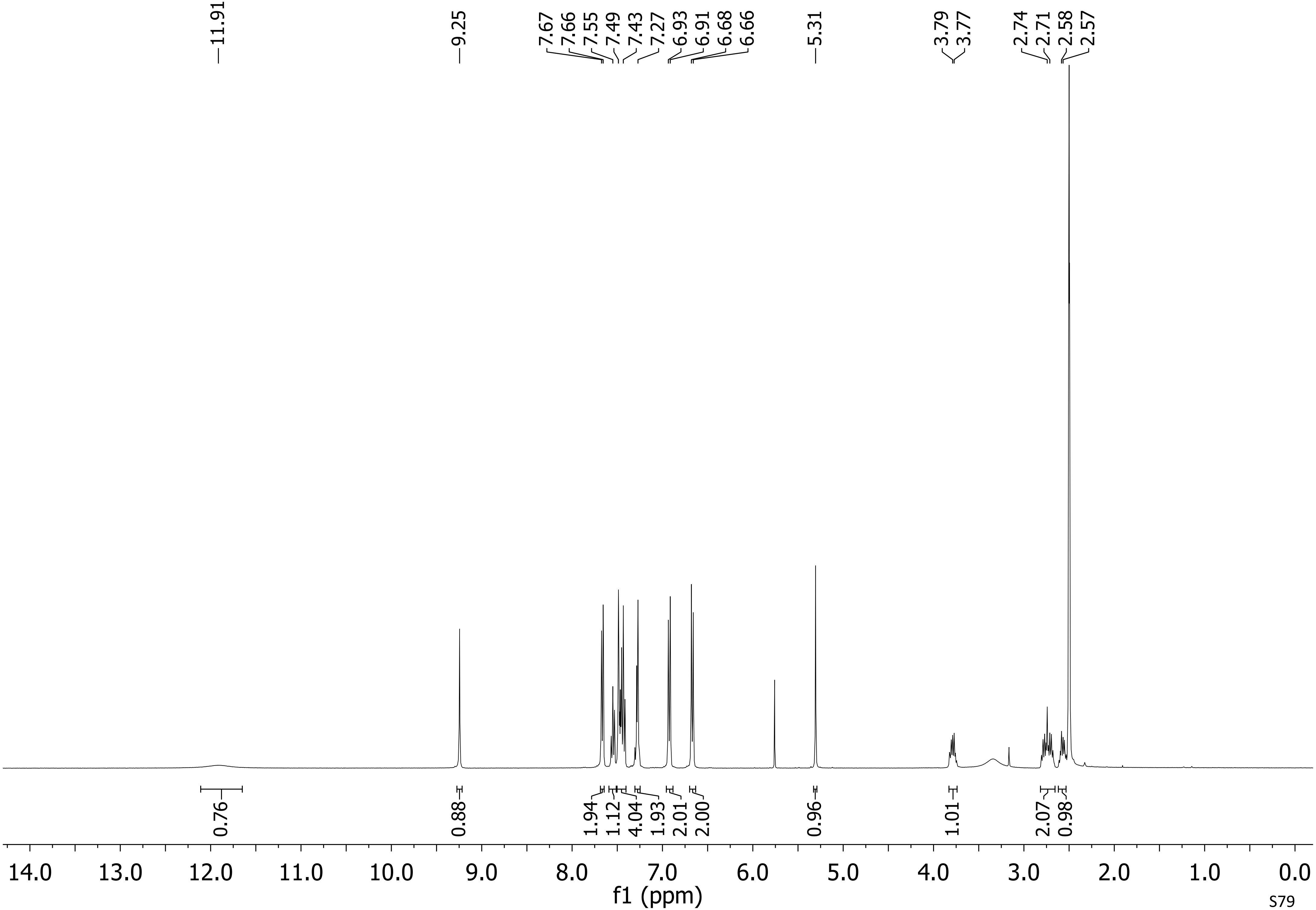

DEPTQ ^{13}C NMR (101 MHz, DMSO-d_6) for compound **32**

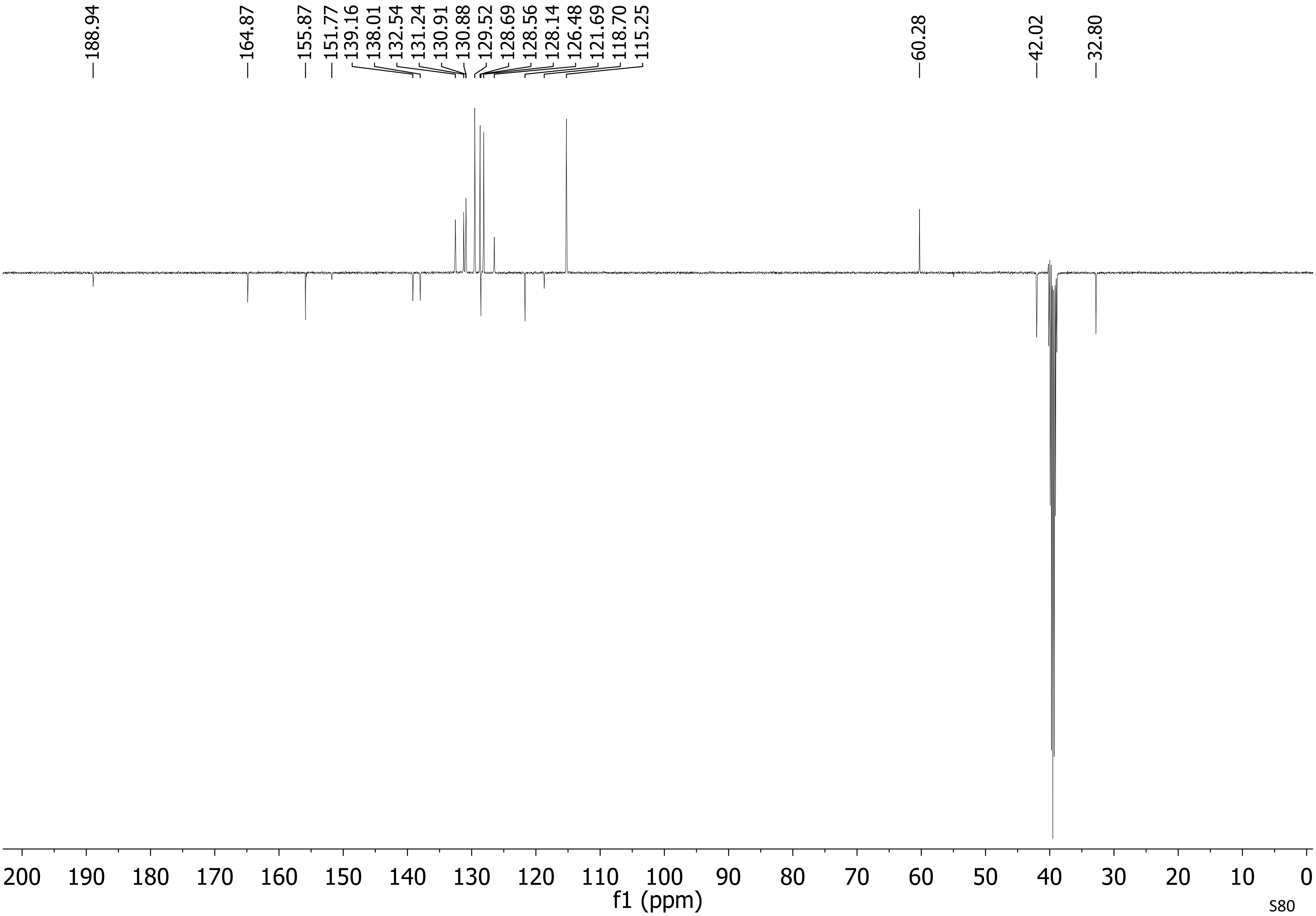

^{1}H NMR (400 MHz, DMSO-d_6) for compound **33**

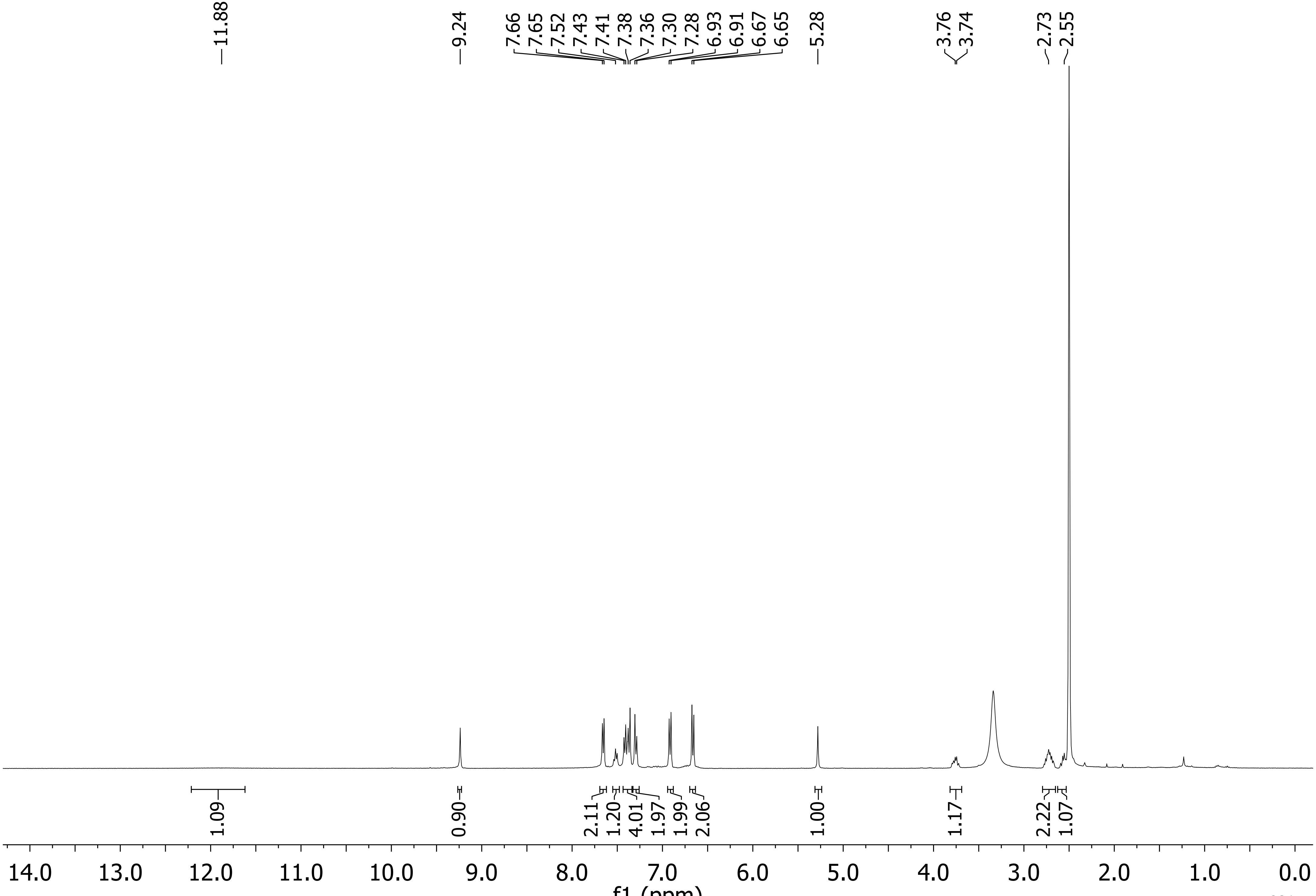

^{13}C NMR (151 MHz, DMSO-d_6) for compound **33**

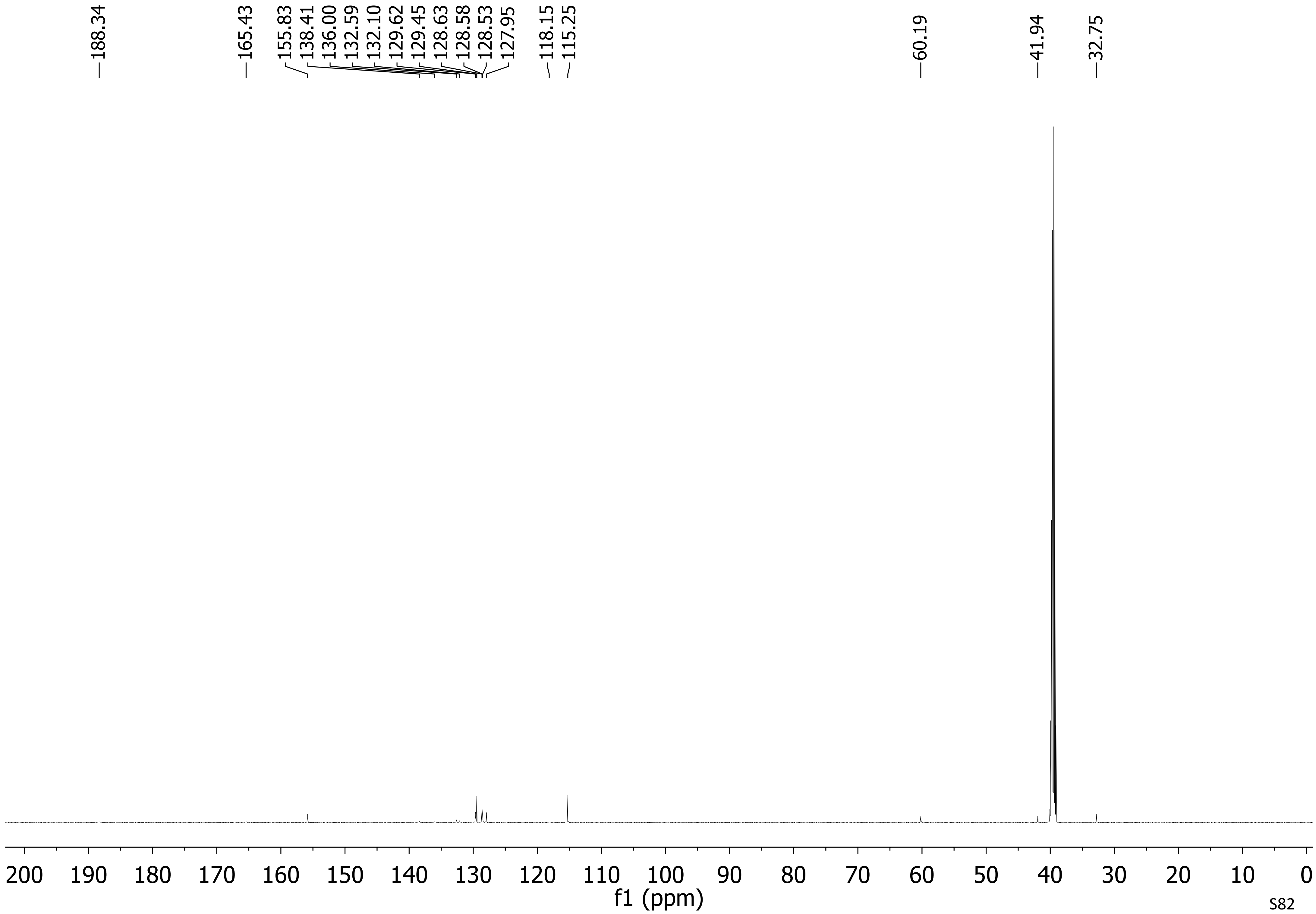

^{13}C NMR (151 MHz, DMSO-d_6) for compound **33** (zoomed-in view)

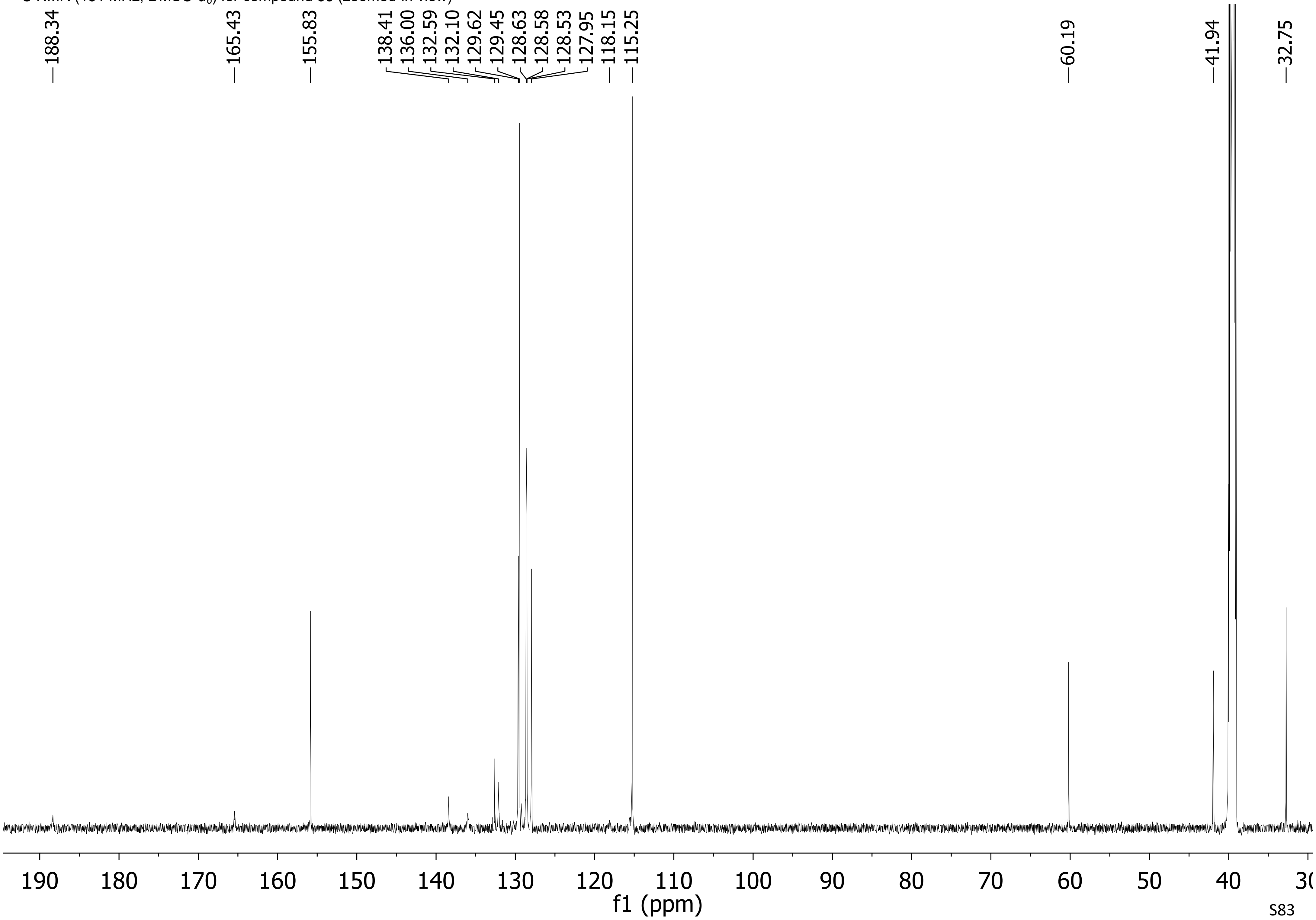

^{1}H NMR (400 MHz, DMSO-d_6) for compound **34**

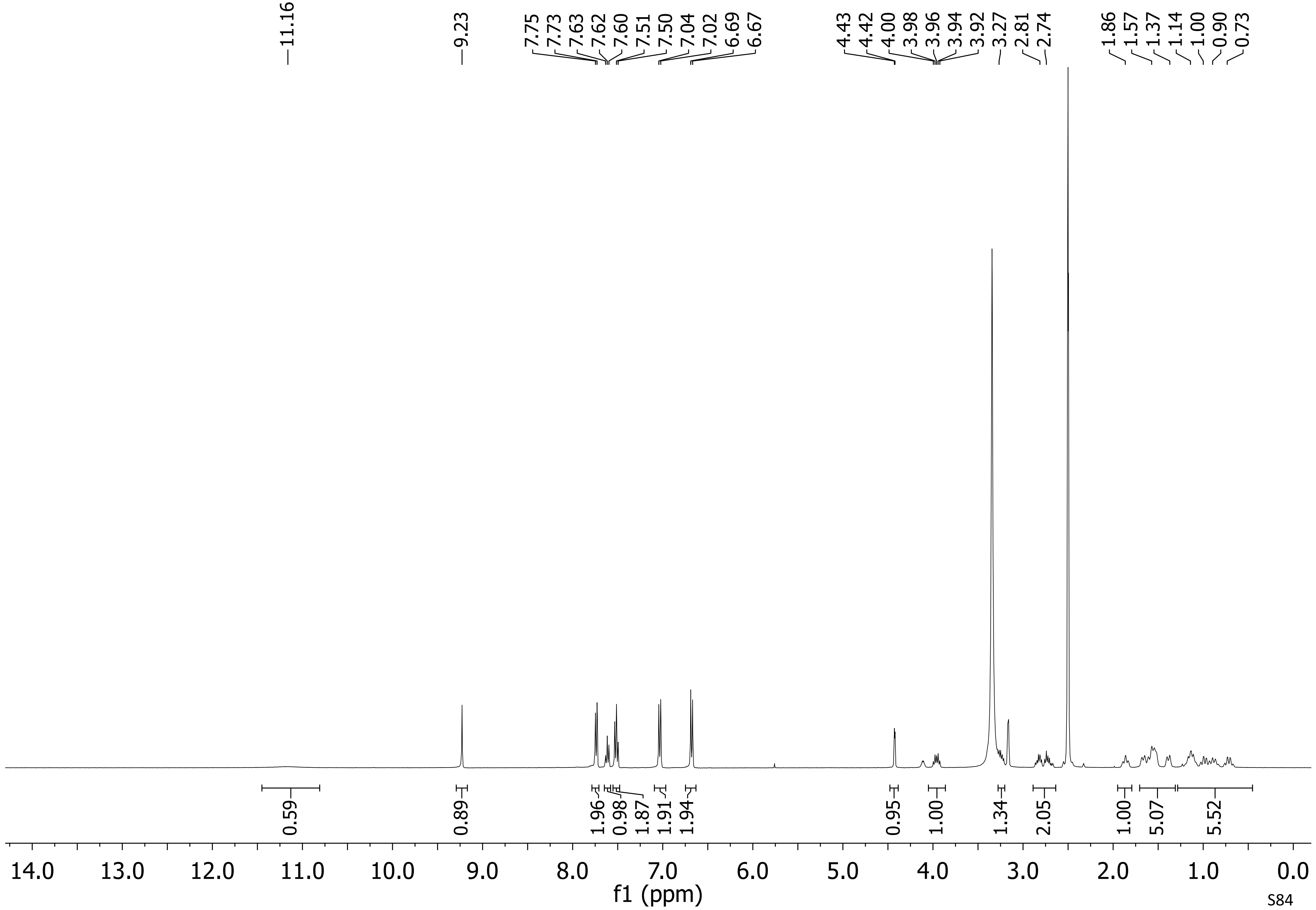

DEPTQ ^{13}C NMR (101 MHz, DMSO-d_6) for compound **34**

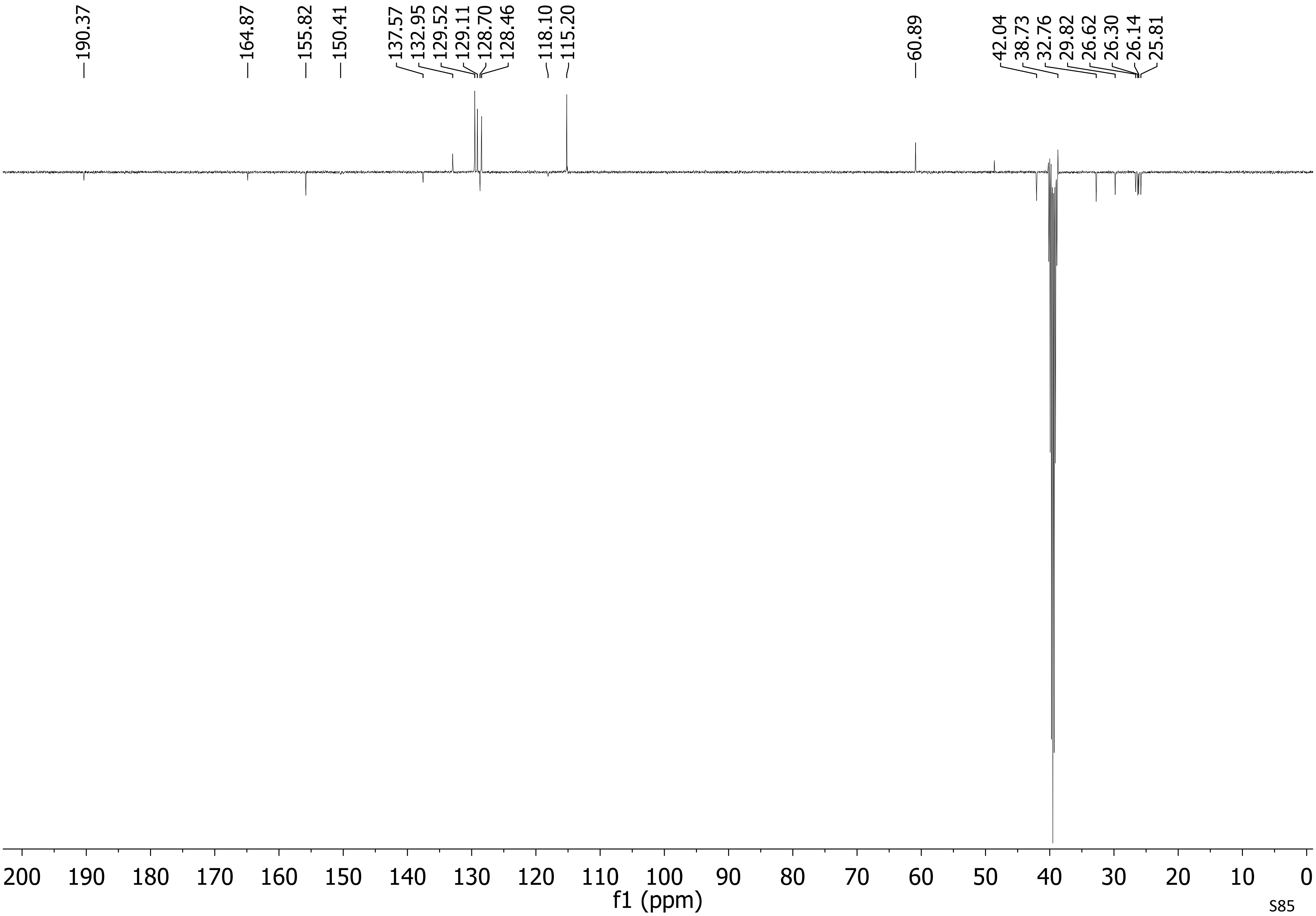

^{1}H NMR (400 MHz, DMSO-d_6) for compound **35**

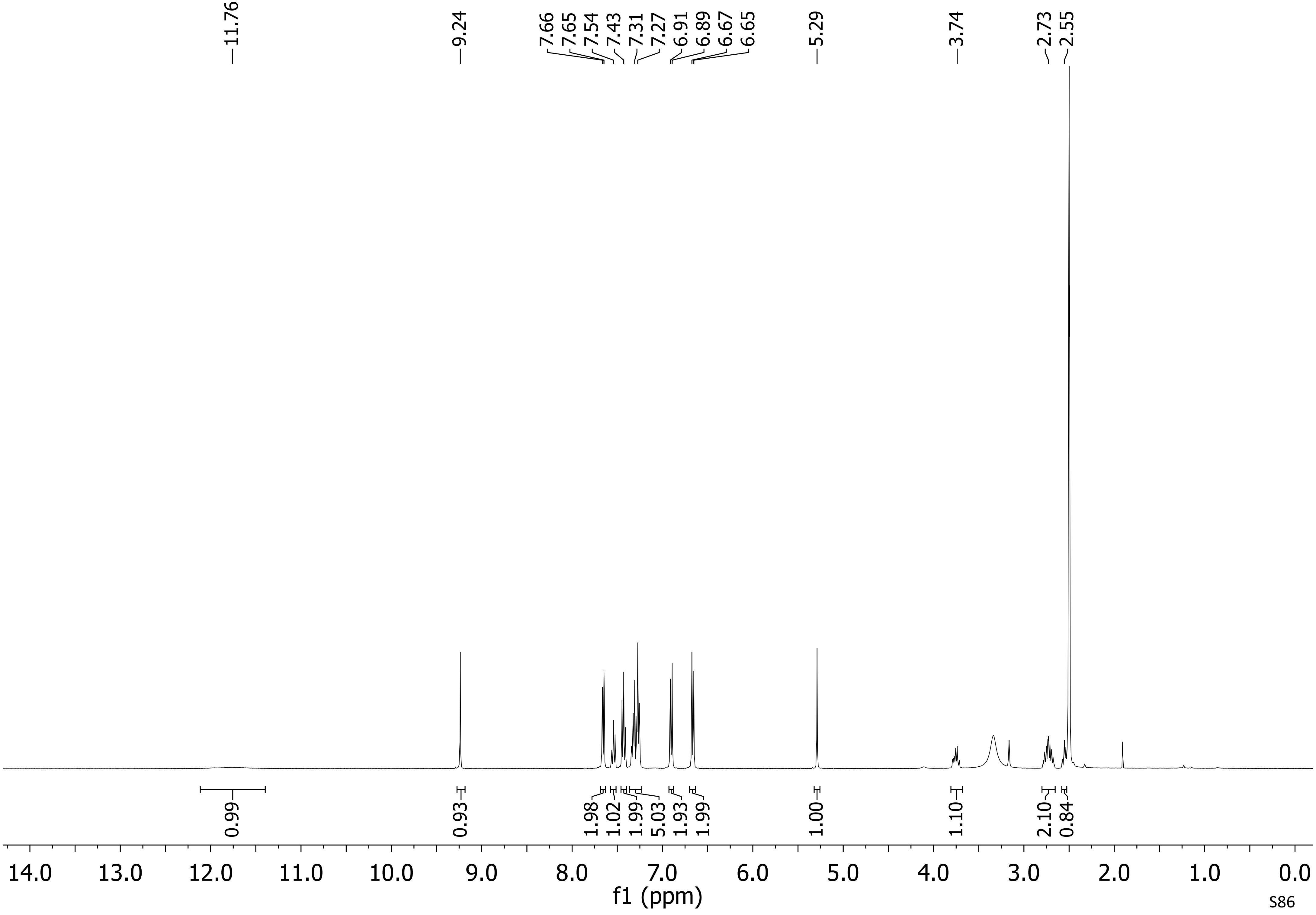

DEPTQ ^{13}C NMR (151 MHz, DMSO-d_6) for compound **35**

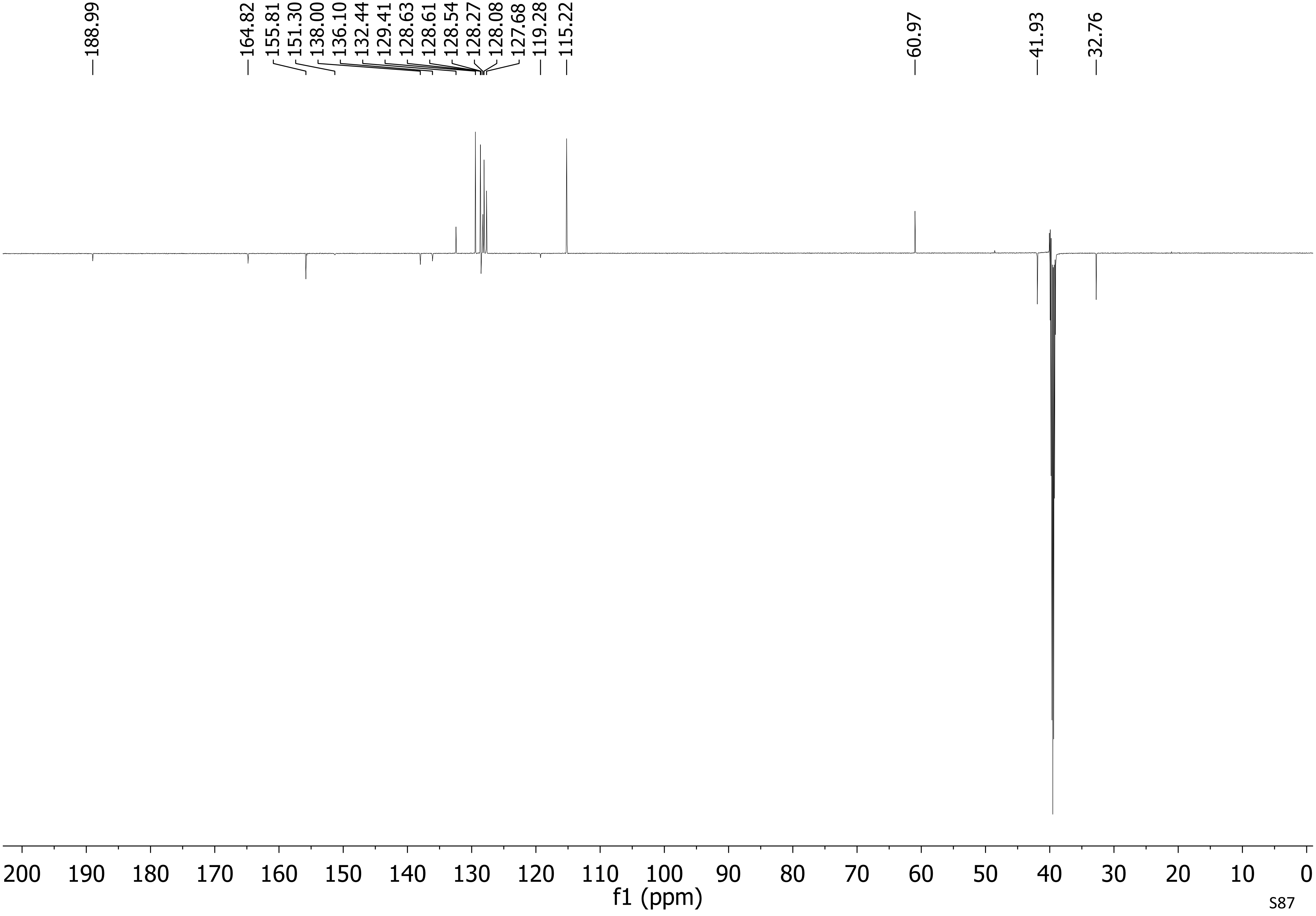

^1H NMR (400 MHz, DMSO-d_6) for compound **36**

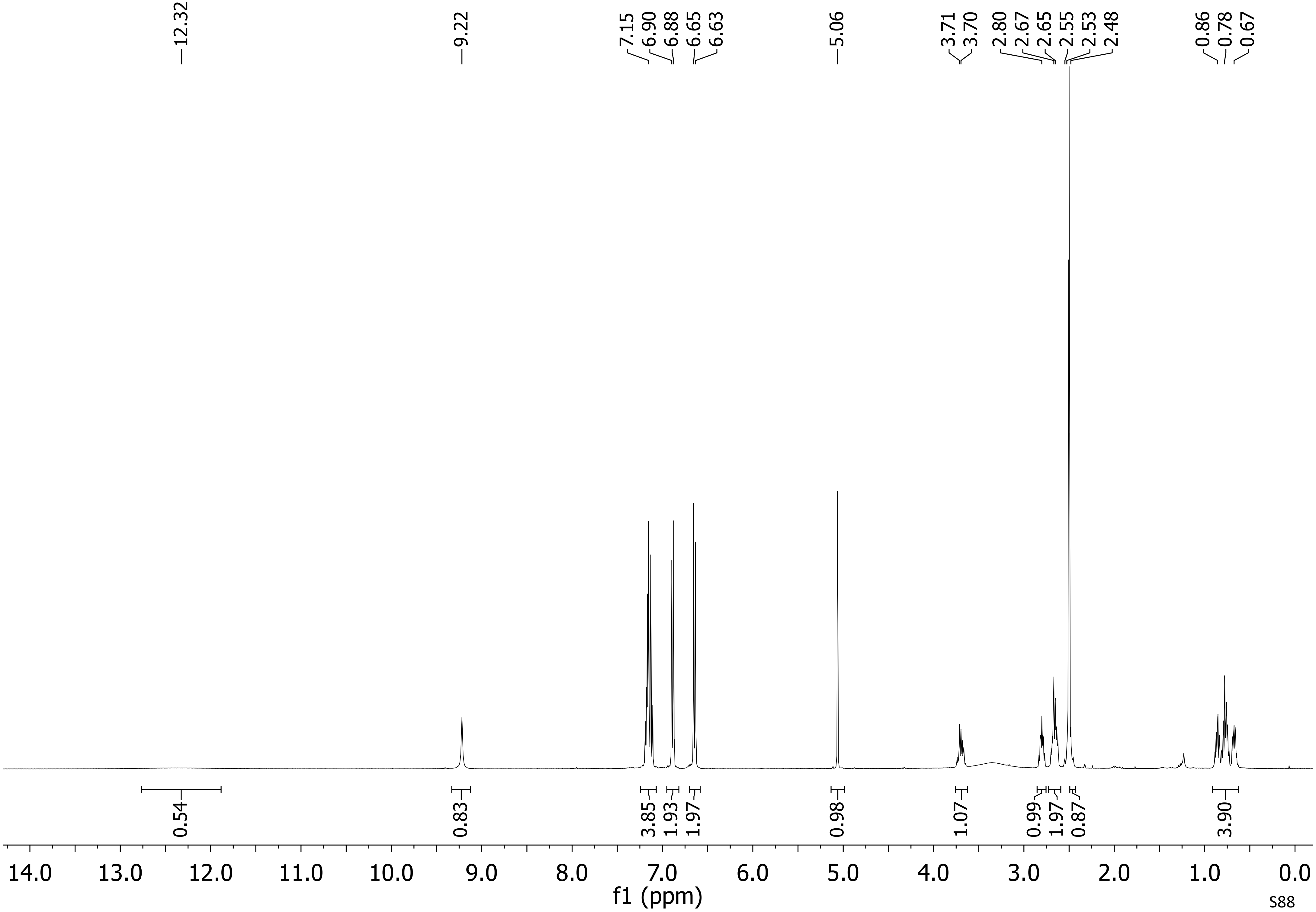

DEPTQ ^{13}C NMR (101 MHz, DMSO-d_6) for compound **36**

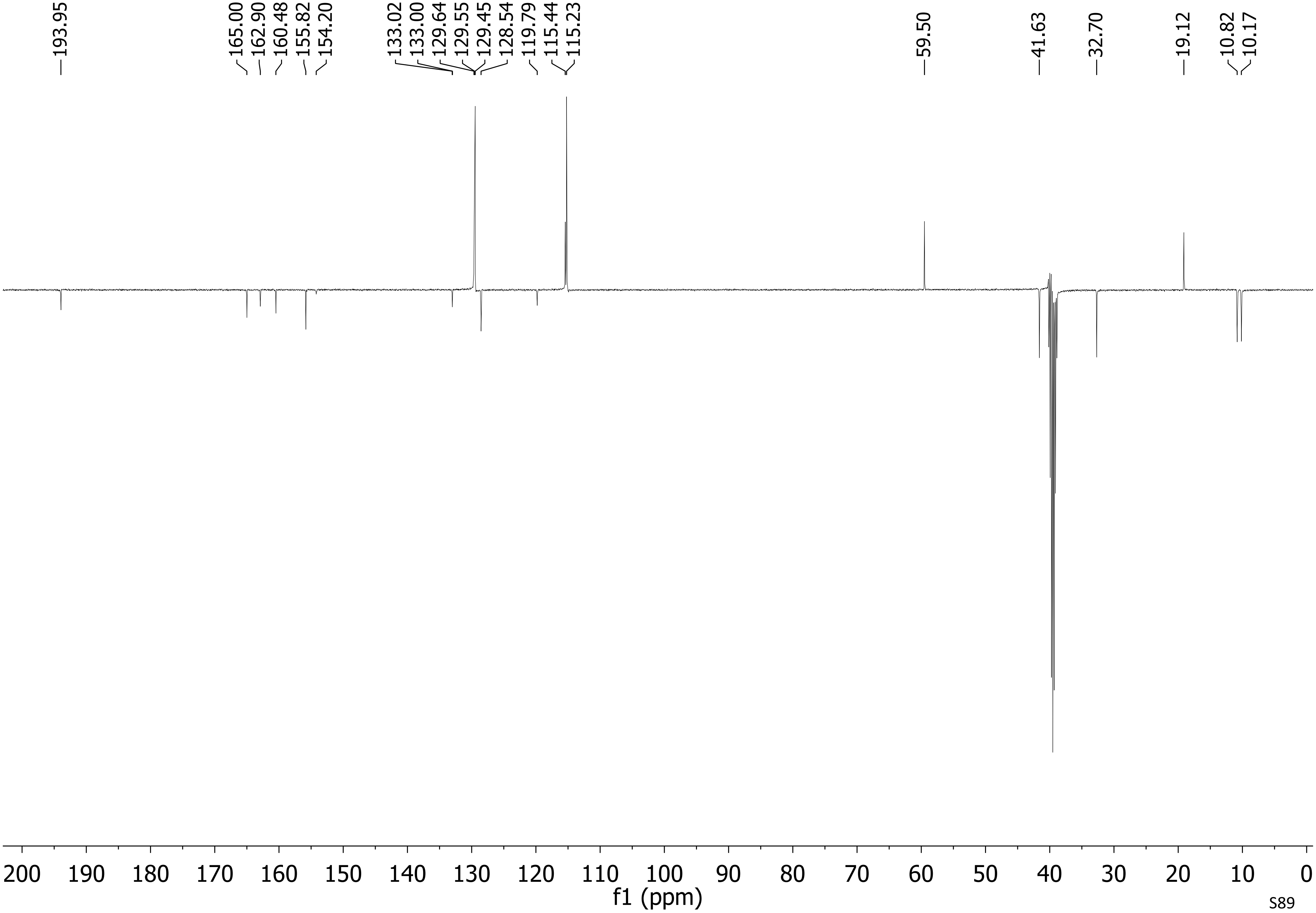

^{1}H NMR (400 MHz, DMSO-d_6) for compound **37**

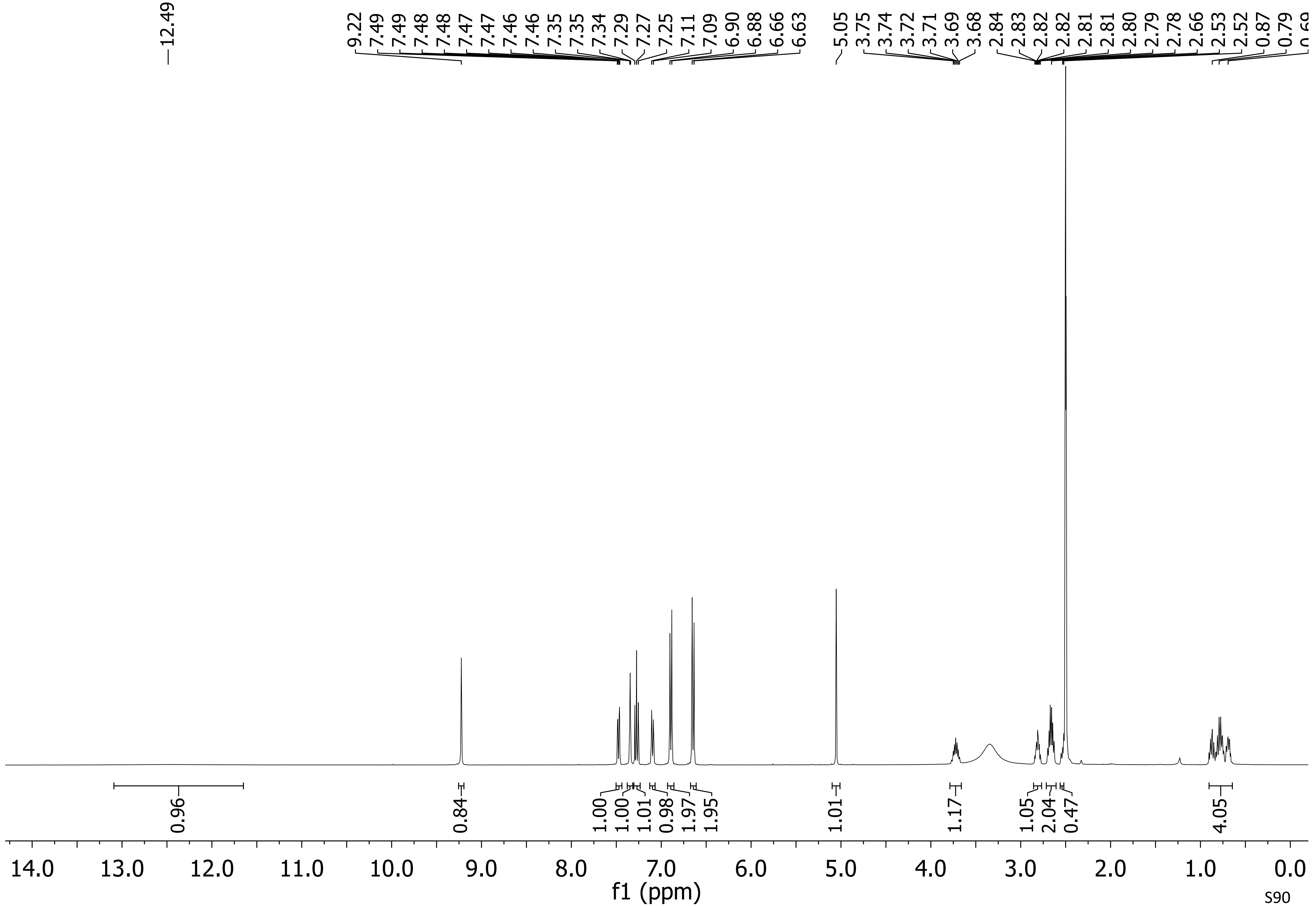

DEPTQ ^{13}C NMR (101 MHz, DMSO-d_6) for compound **37**

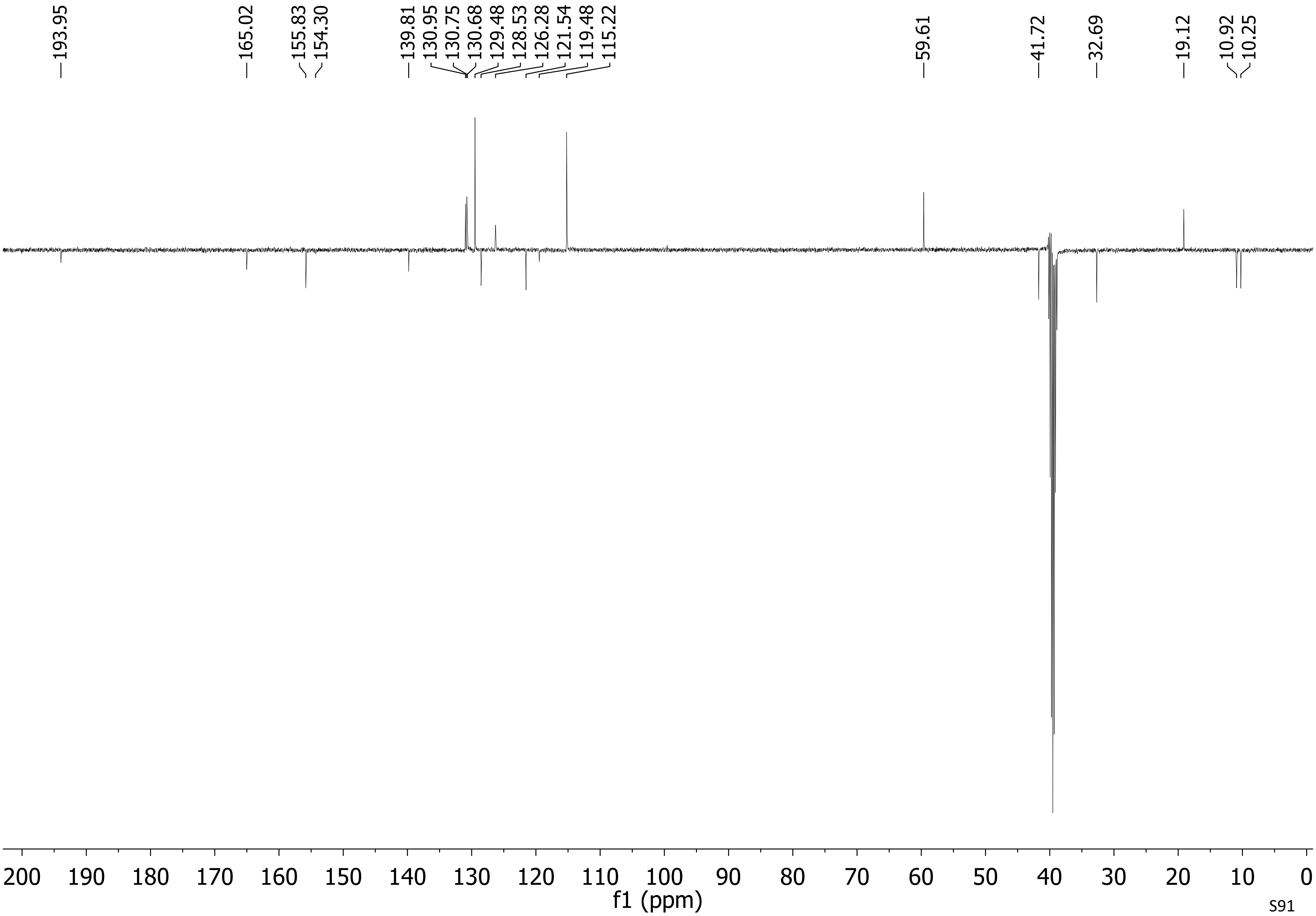

^{1}H NMR (500 MHz, DMSO-d_6) for compound **38**

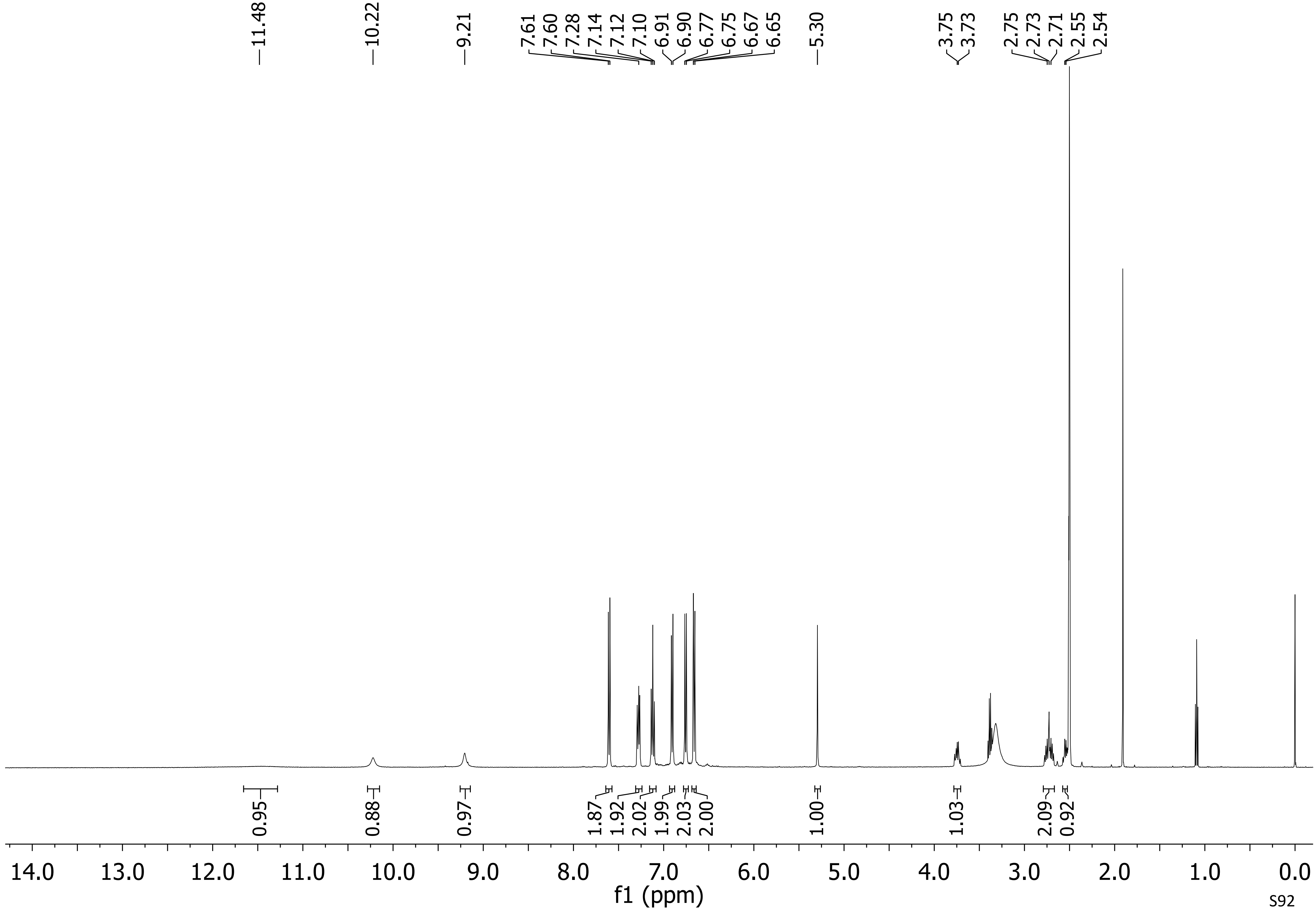

^{13}C NMR (126 MHz, DMSO-d_6) for compound **38**

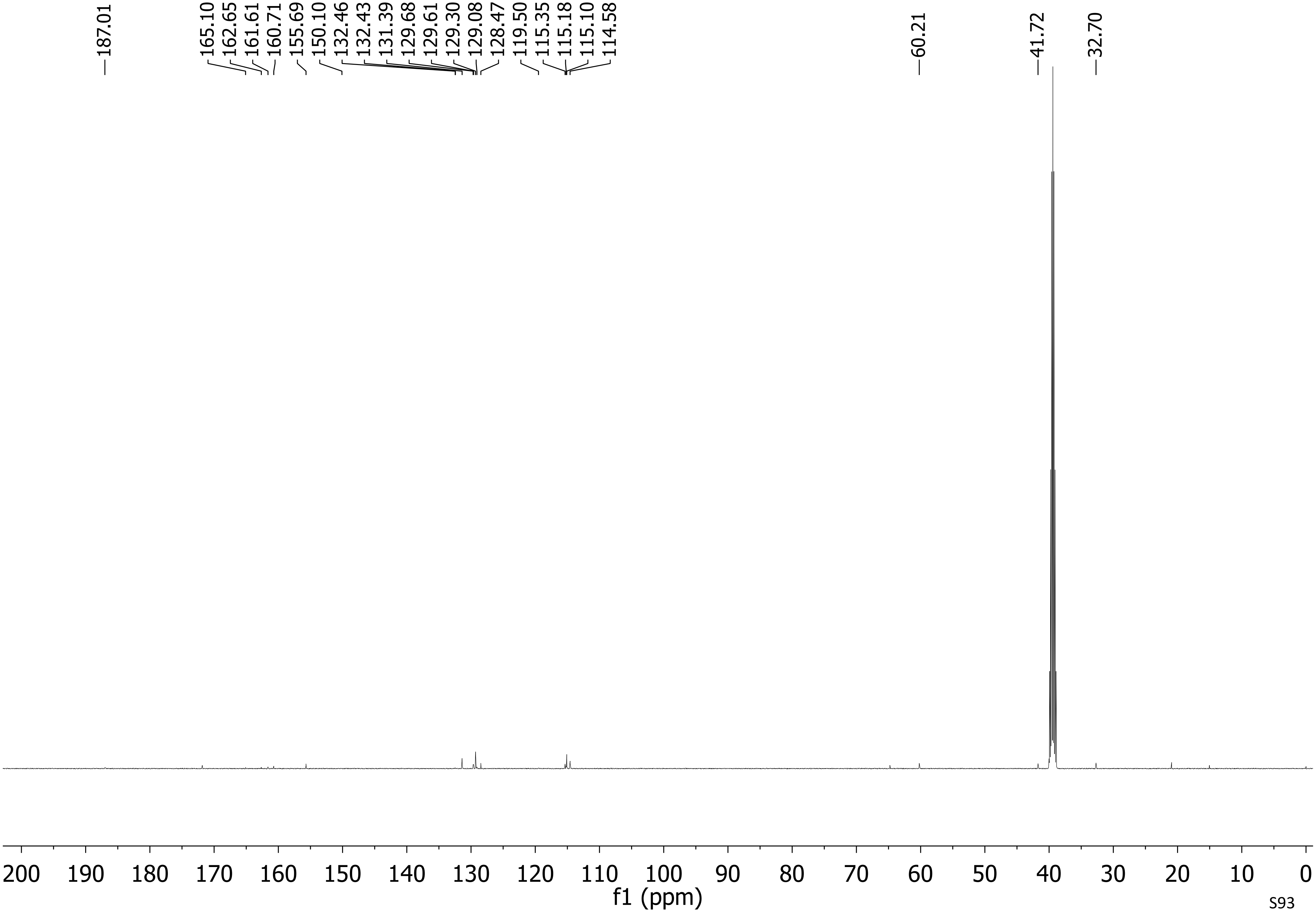

^{13}C NMR (126 MHz, DMSO-d_6) for compound **38** (zoomed-in view)

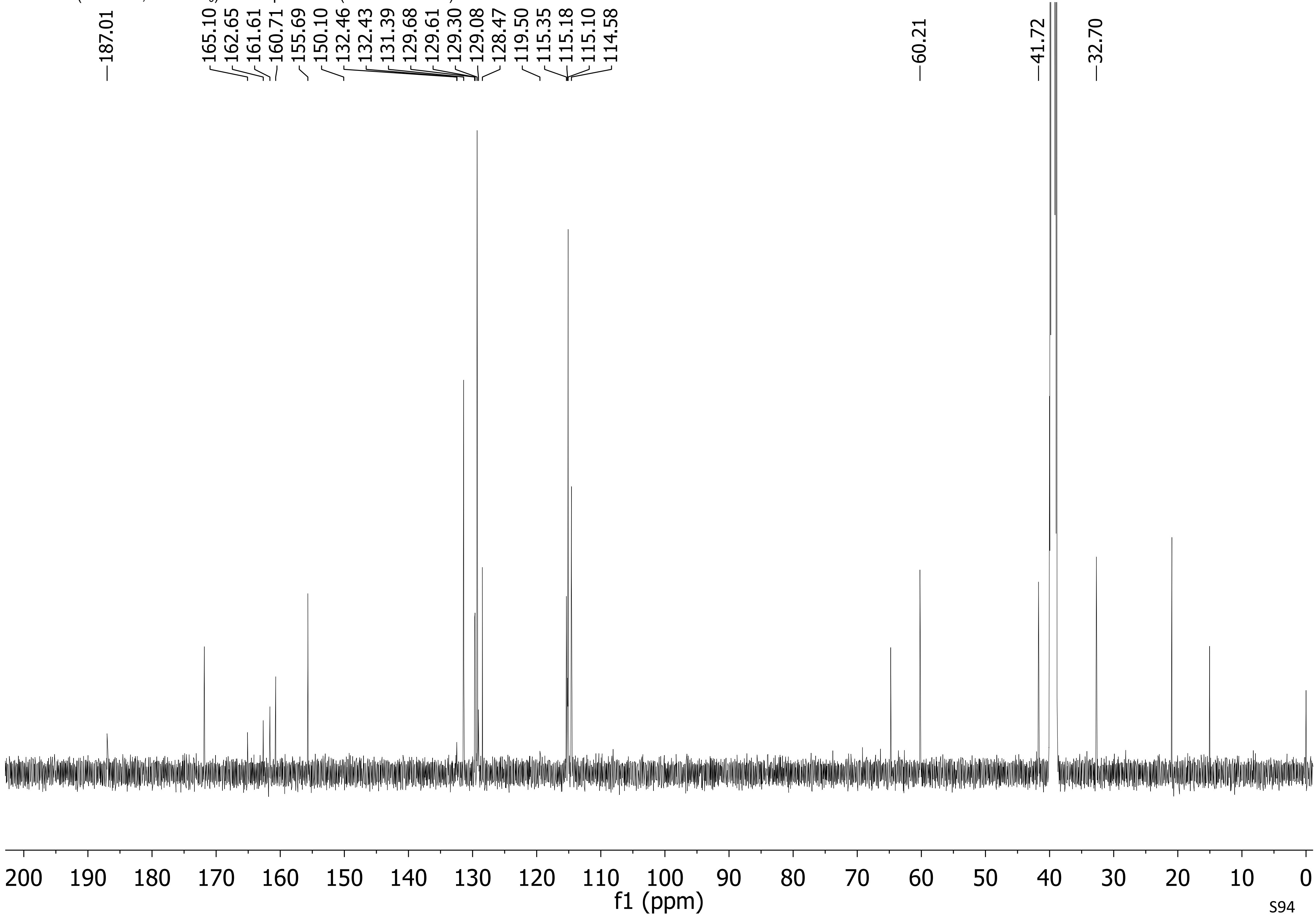

^1H NMR (600 MHz, DMSO-d_6) for compound **39**

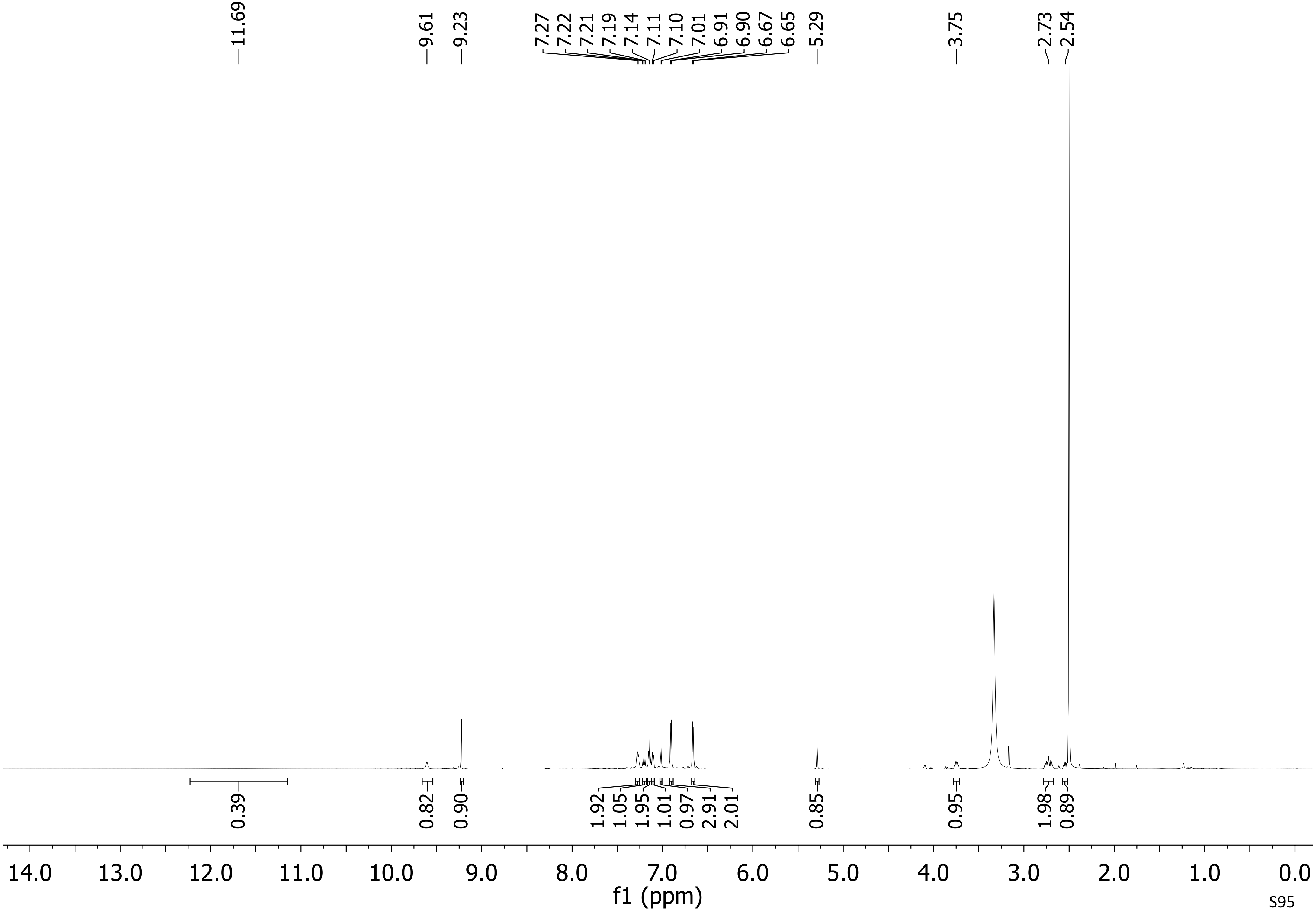

DEPTQ ^{13}C NMR (151 MHz, DMSO-d_6) for compound **39**

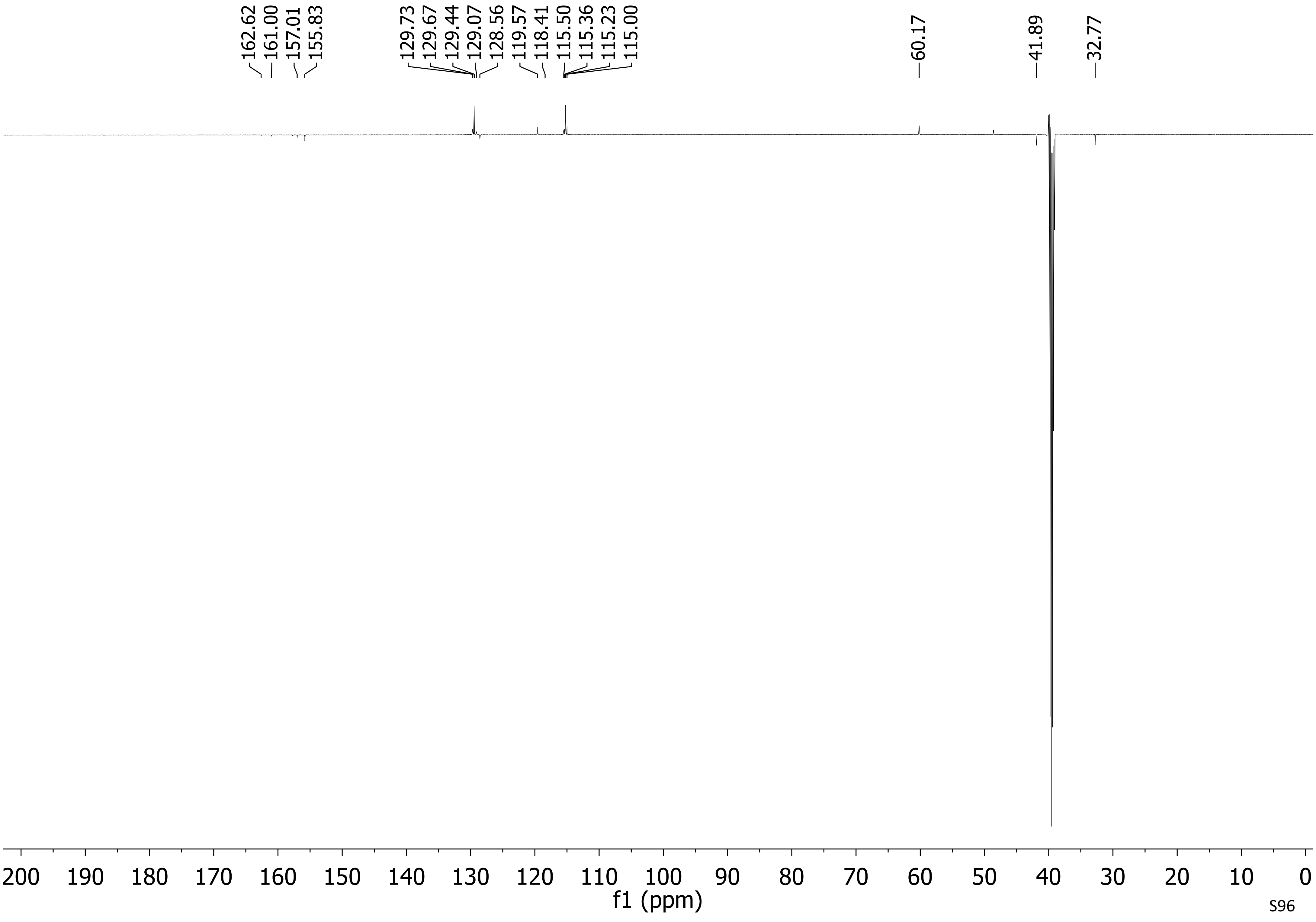

DEPTQ ^{13}C NMR (151 MHz, DMSO-d_6) for compound **39** (zoomed-in view)

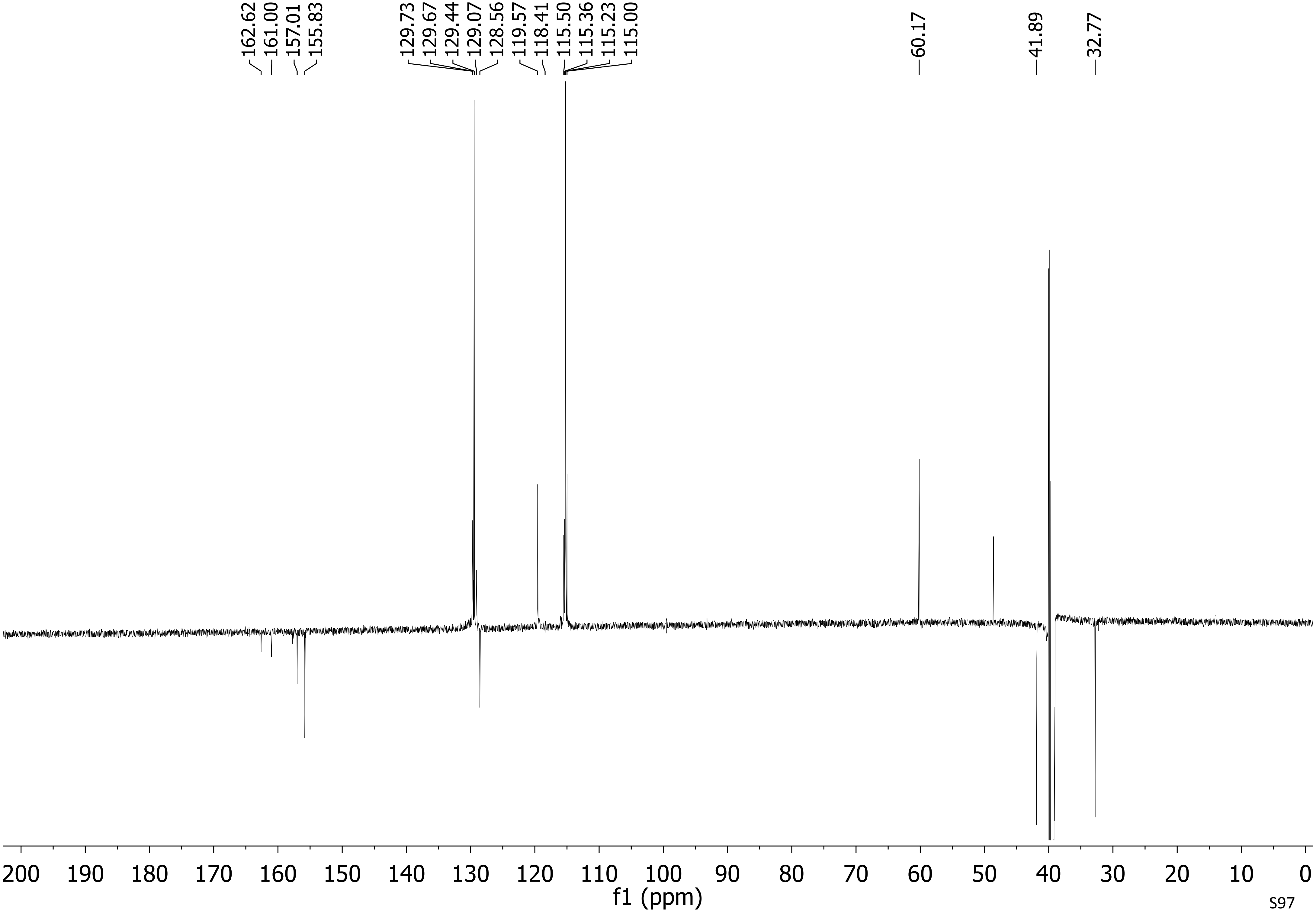

^{1}H NMR (400 MHz, DMSO-d_6) for compound **40**

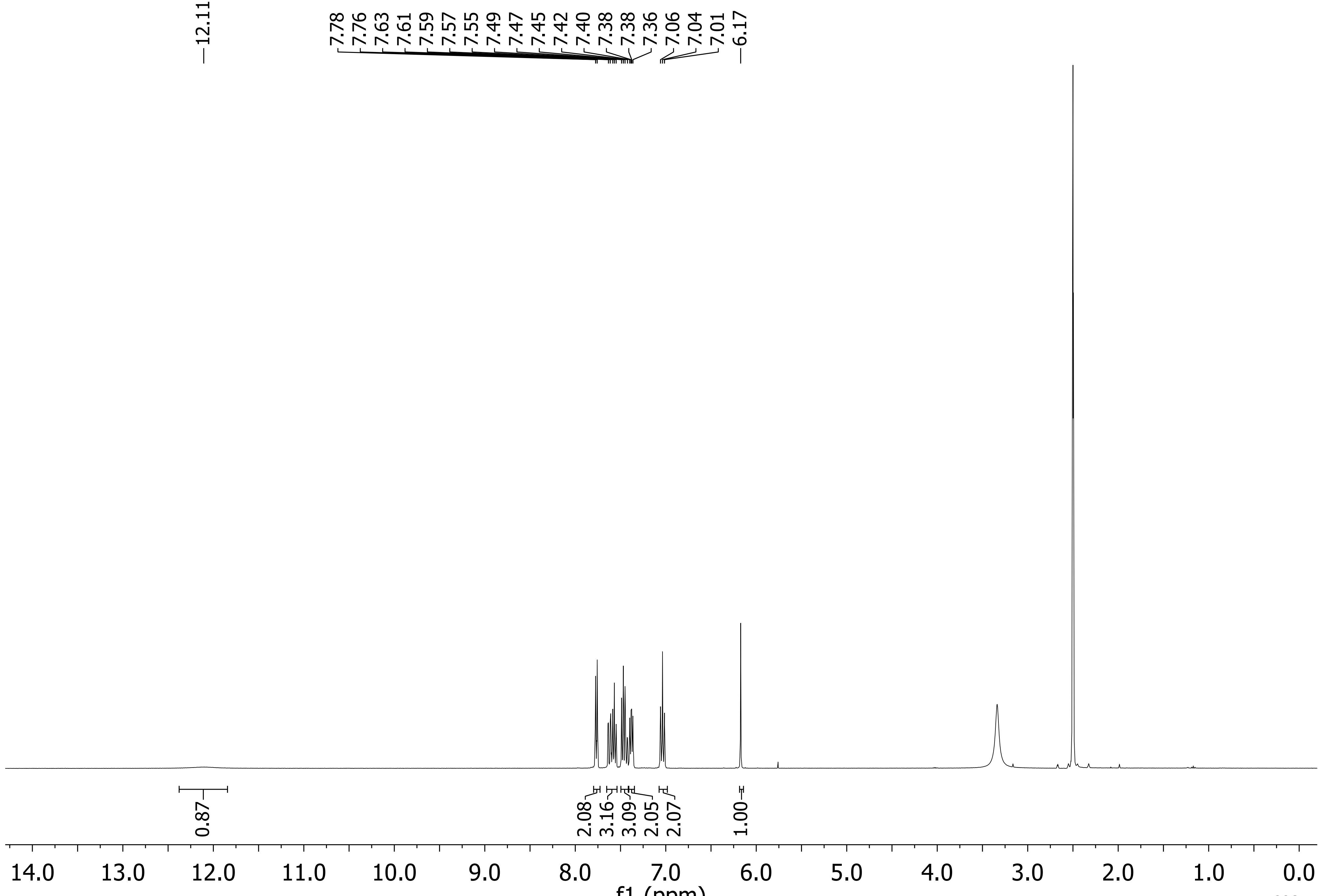

DEPTQ ^{13}C NMR (101 MHz, DMSO-d_6) for compound **40**

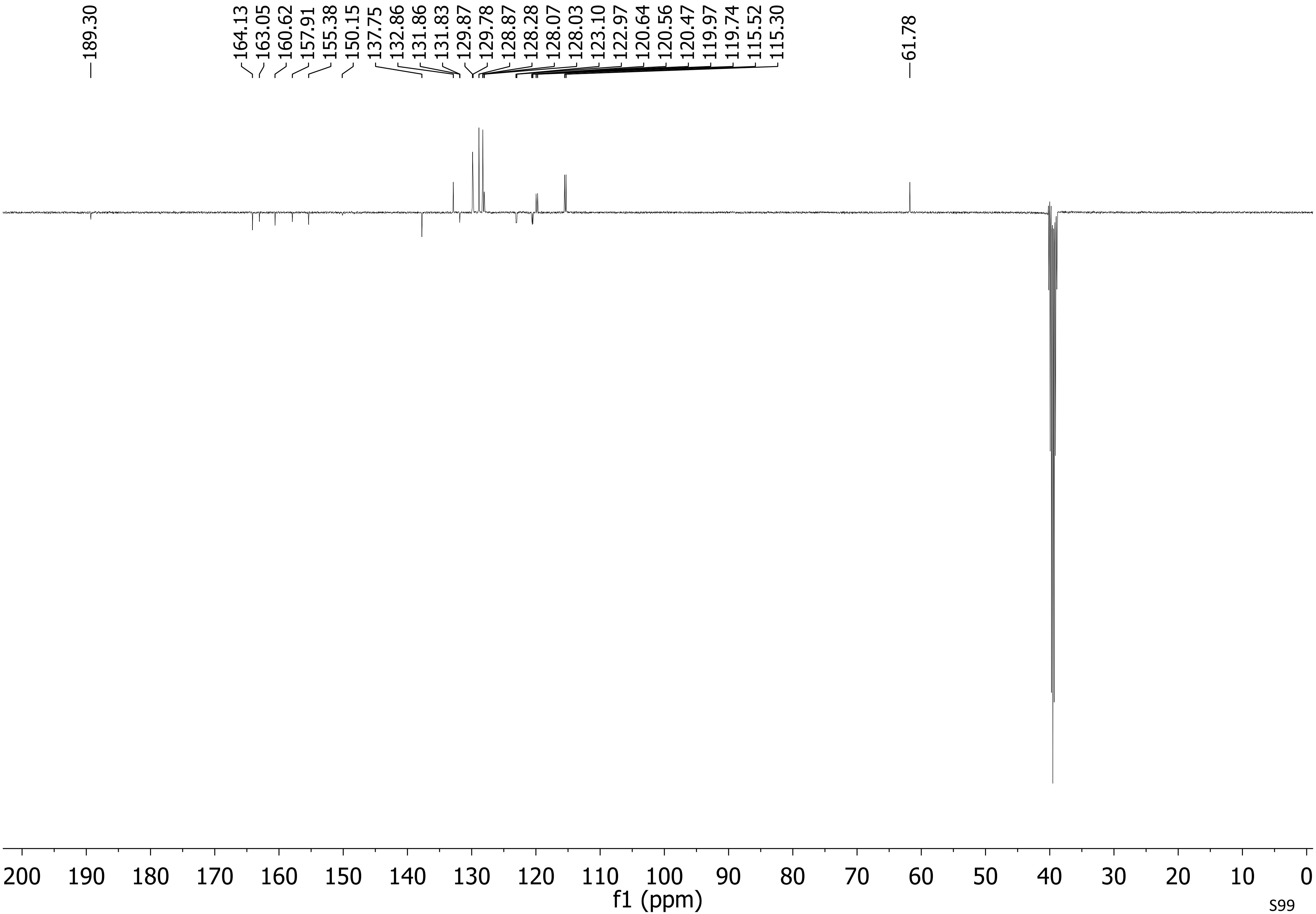

^{1}H NMR (400 MHz, DMSO-d_6) for compound **44**

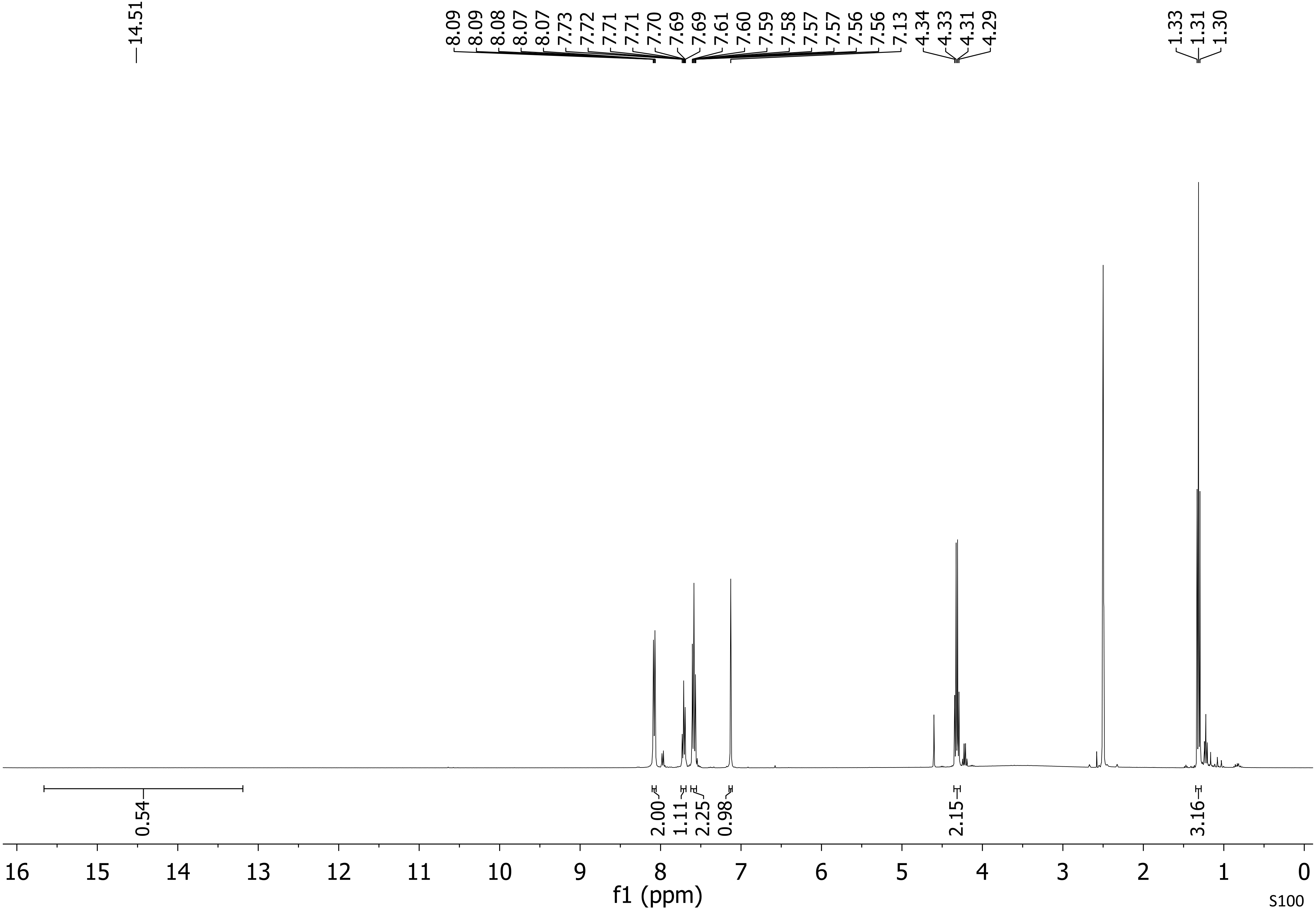

^{1}H NMR (400 MHz, DMSO-d_6) for compound **45**

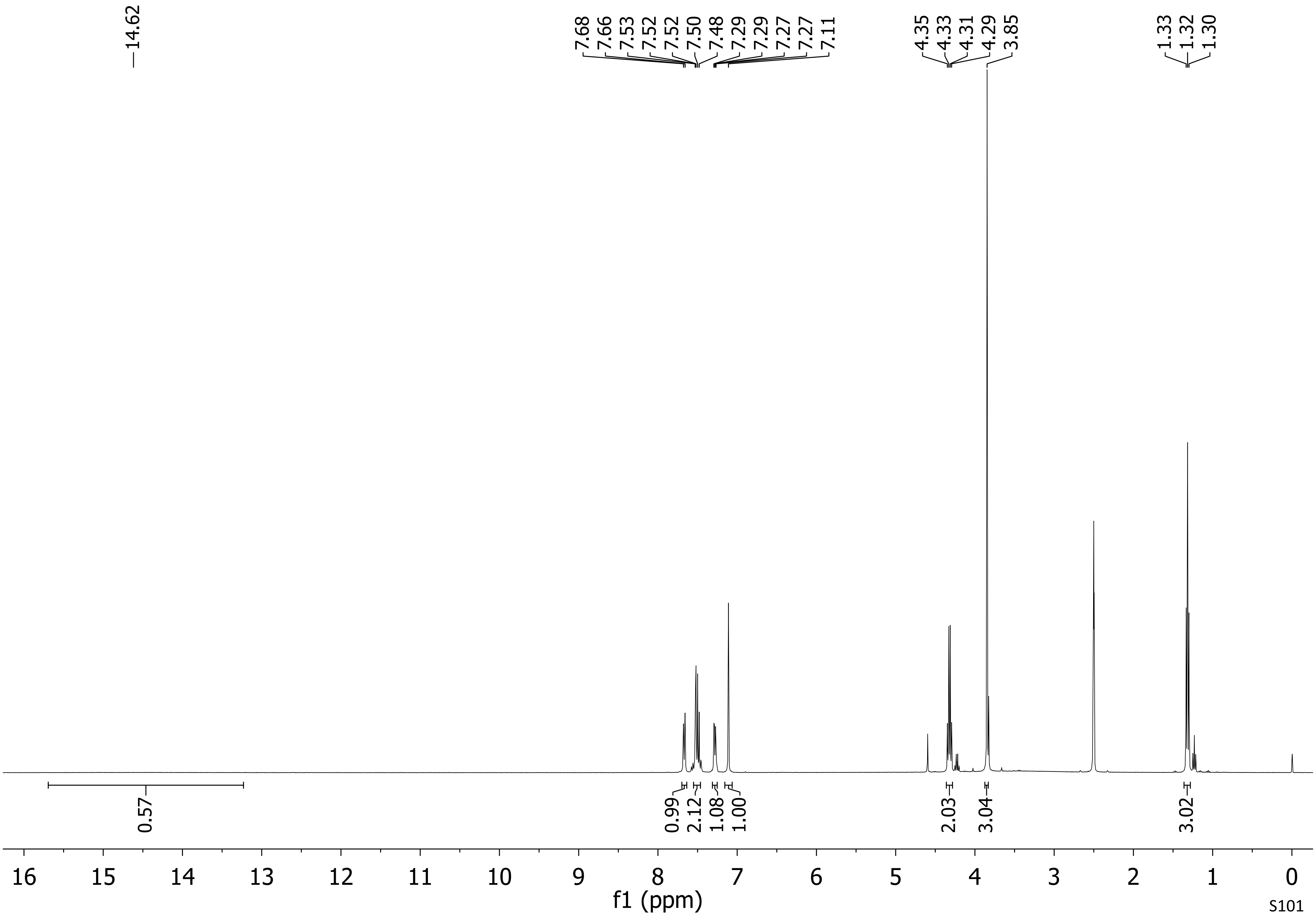

^{13}C NMR (101 MHz, DMSO-d_6) for compound **45**

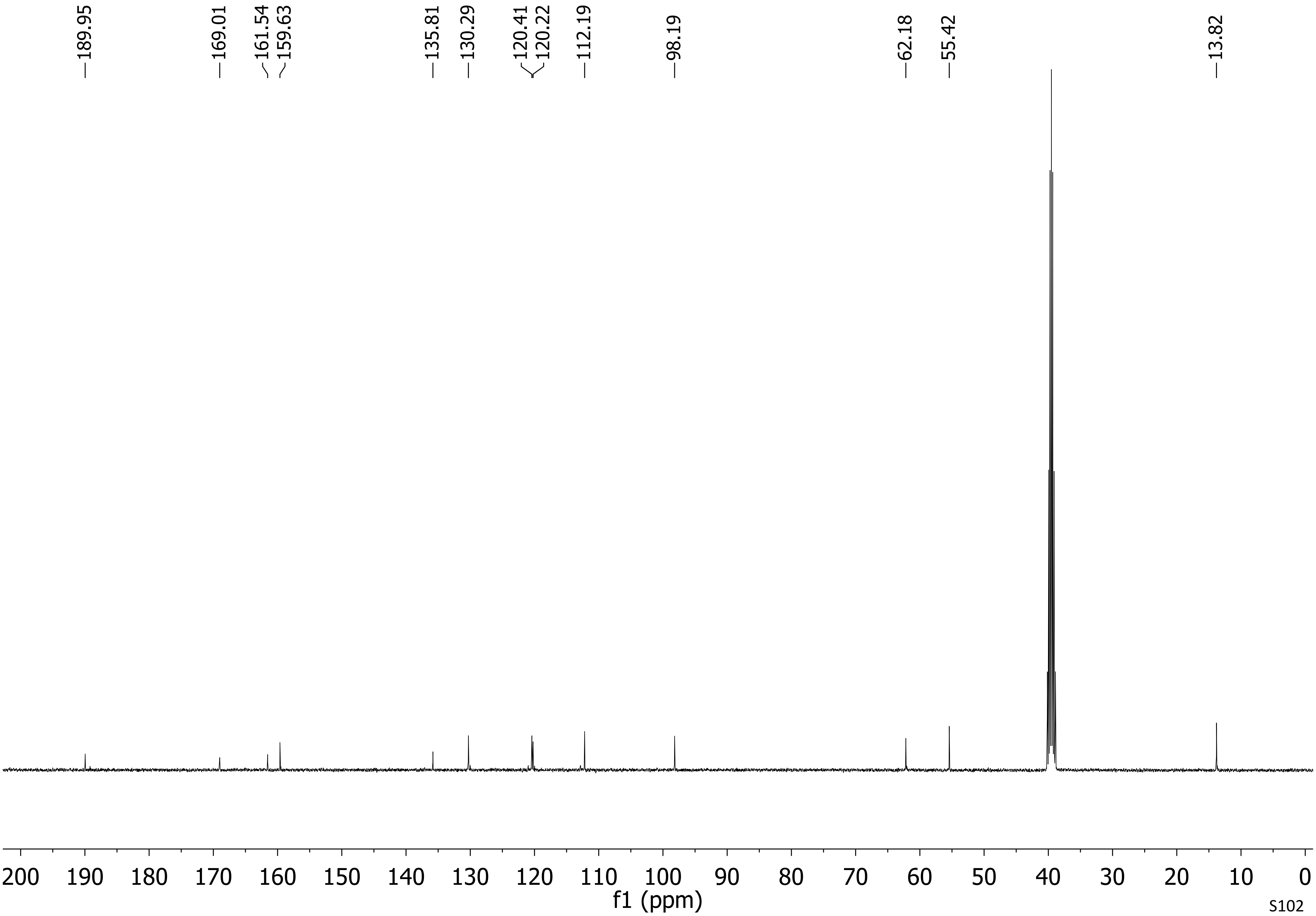

^{1}H NMR (500 MHz, DMSO-d_6) for compound **46**

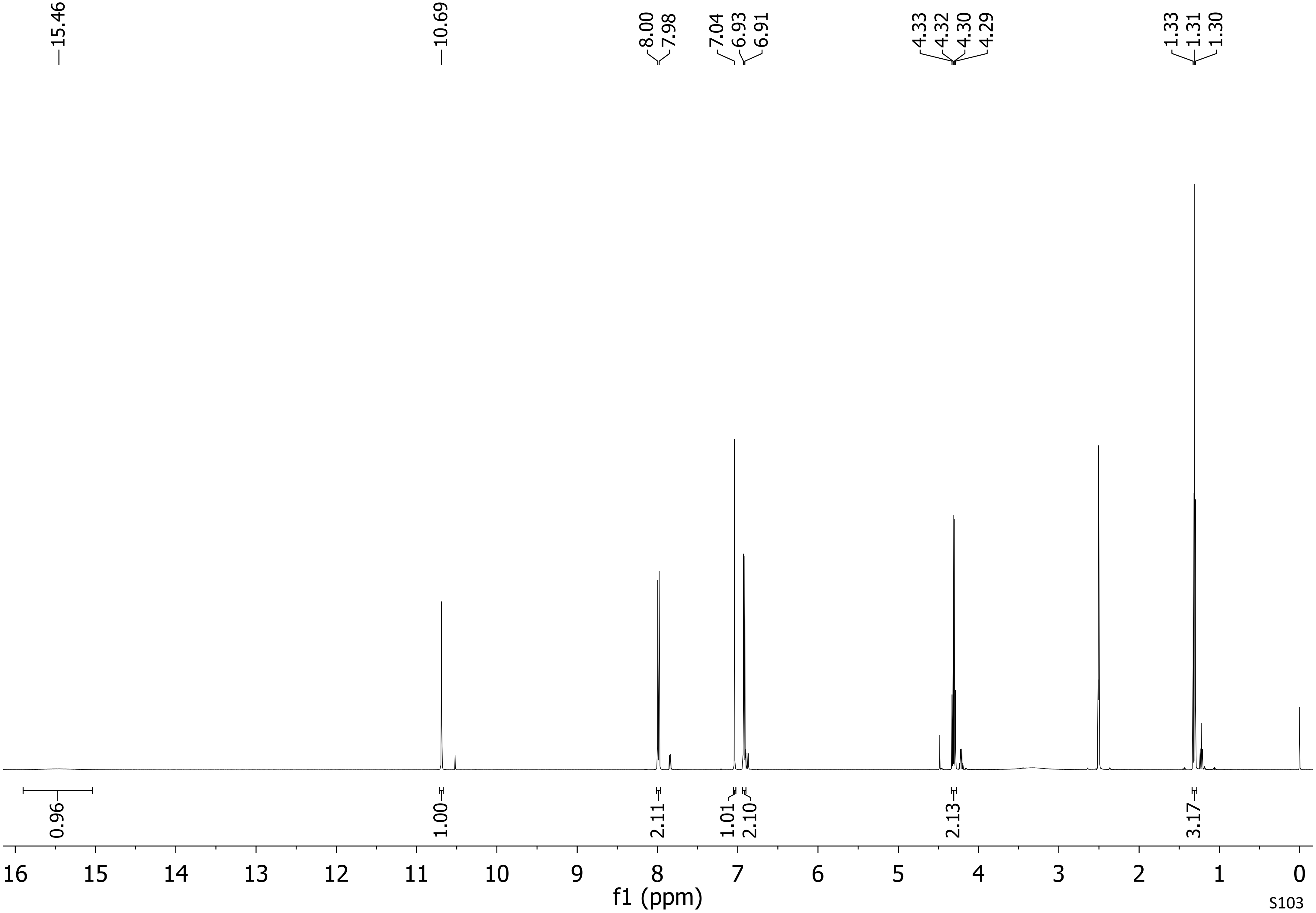

^{13}C NMR (126 MHz, DMSO-d_6) for compound **46**

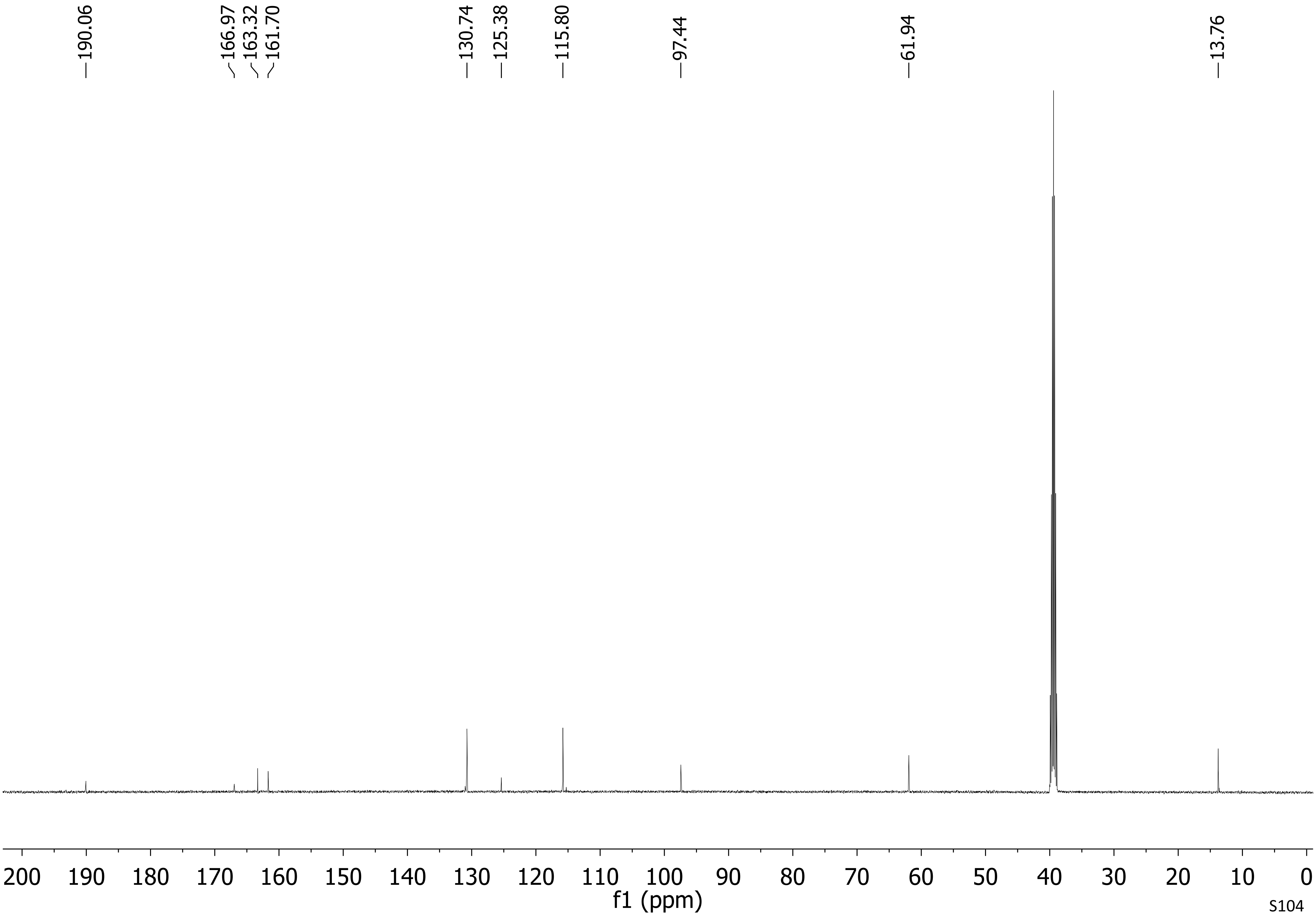

^{1}H NMR (500 MHz, DMSO-d_6) for compound **48**

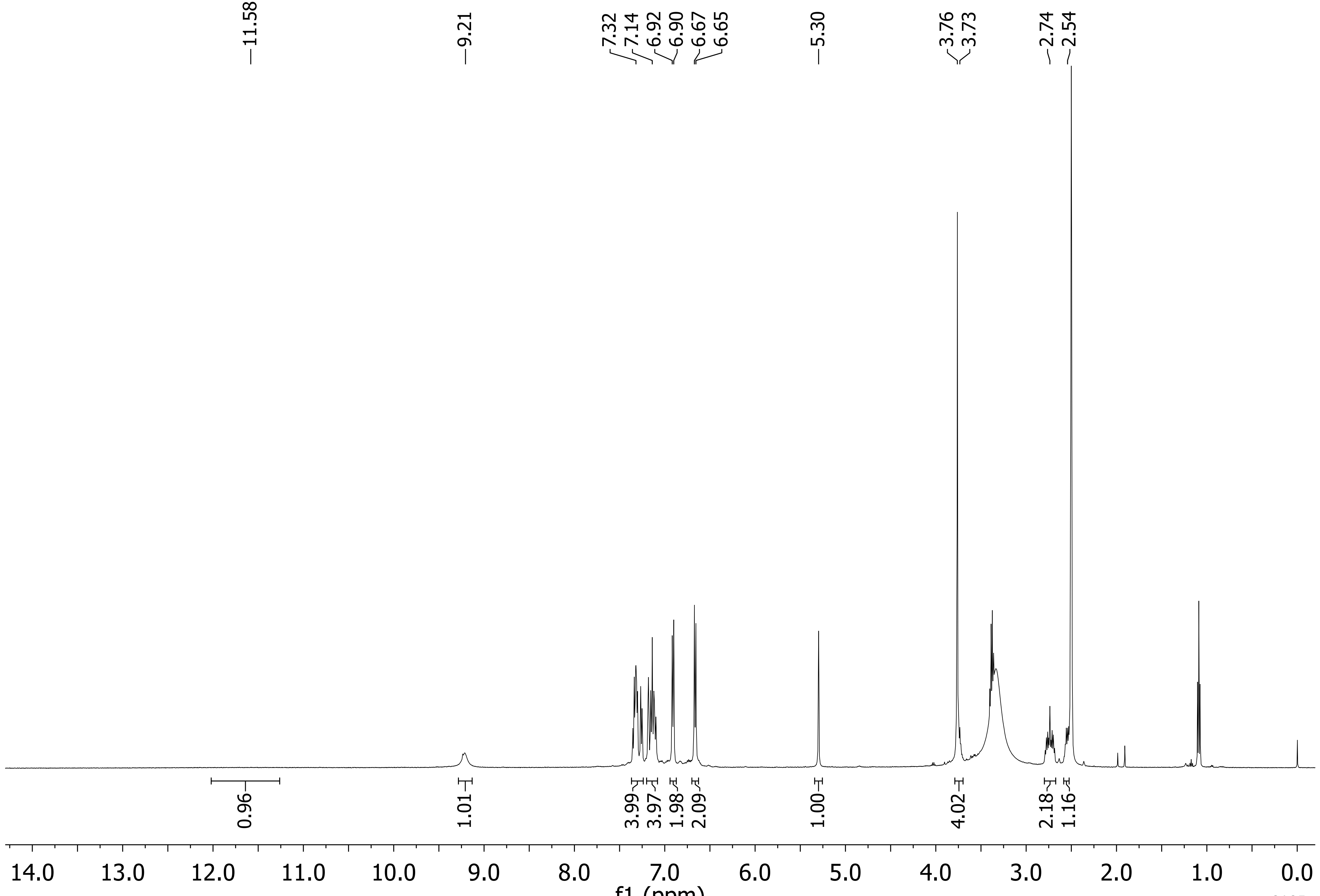

^{13}C NMR (126 MHz, DMSO-d_6) for compound **48**

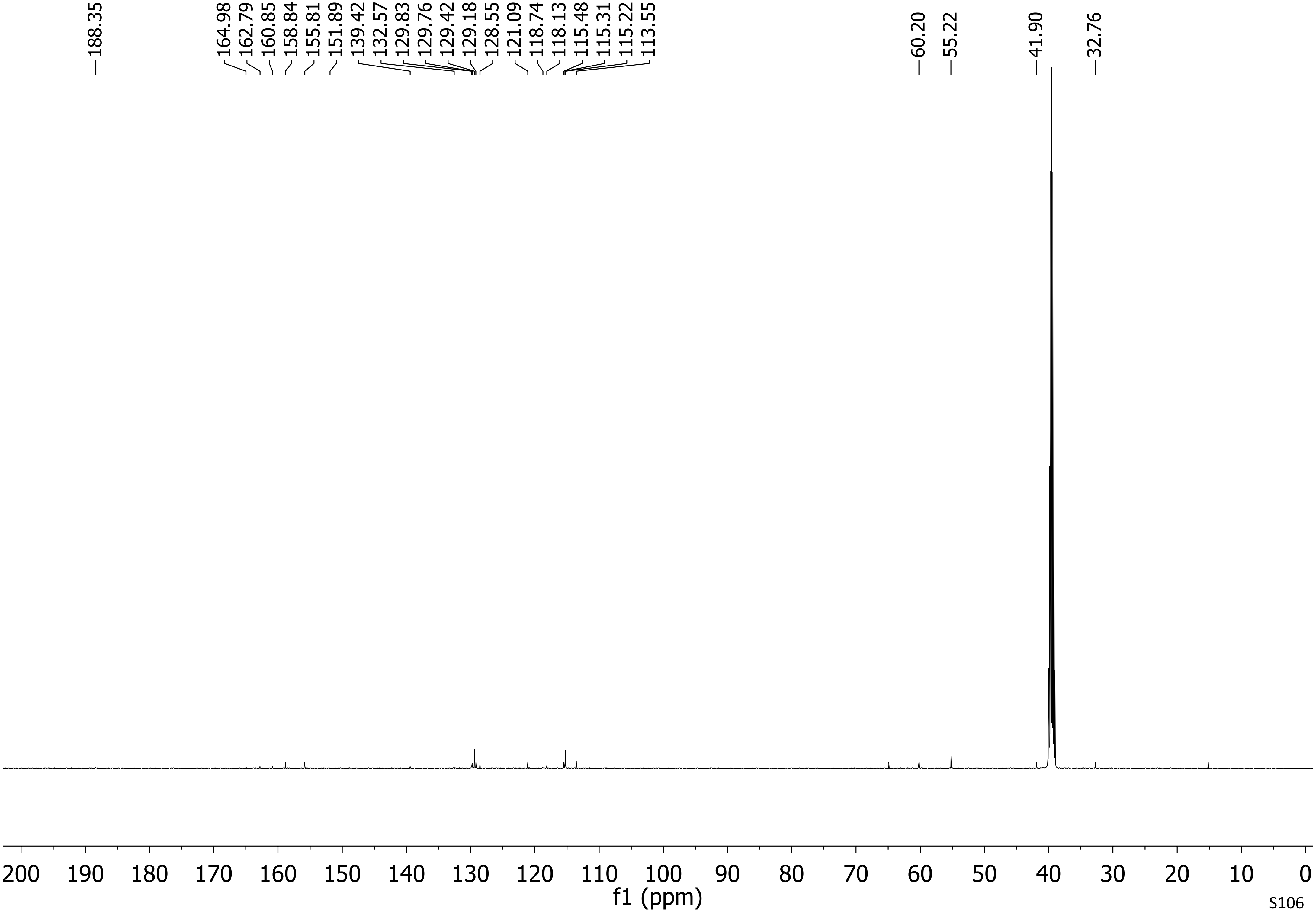

^{13}C NMR (126 MHz, DMSO-d_6) for compound **48** (zoomed-in view)

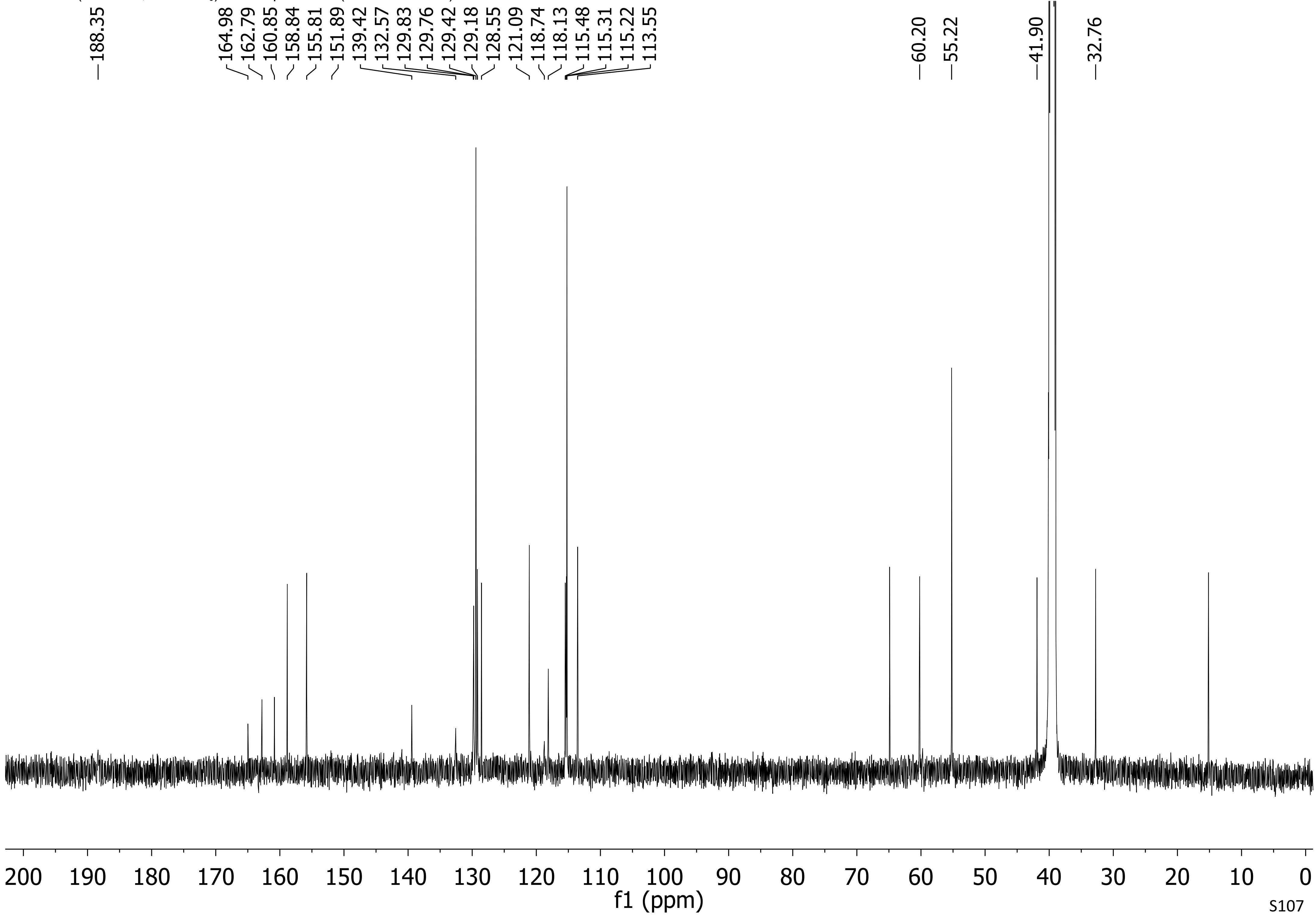

^{1}H NMR (400 MHz, DMSO-d_6) for compound **49**

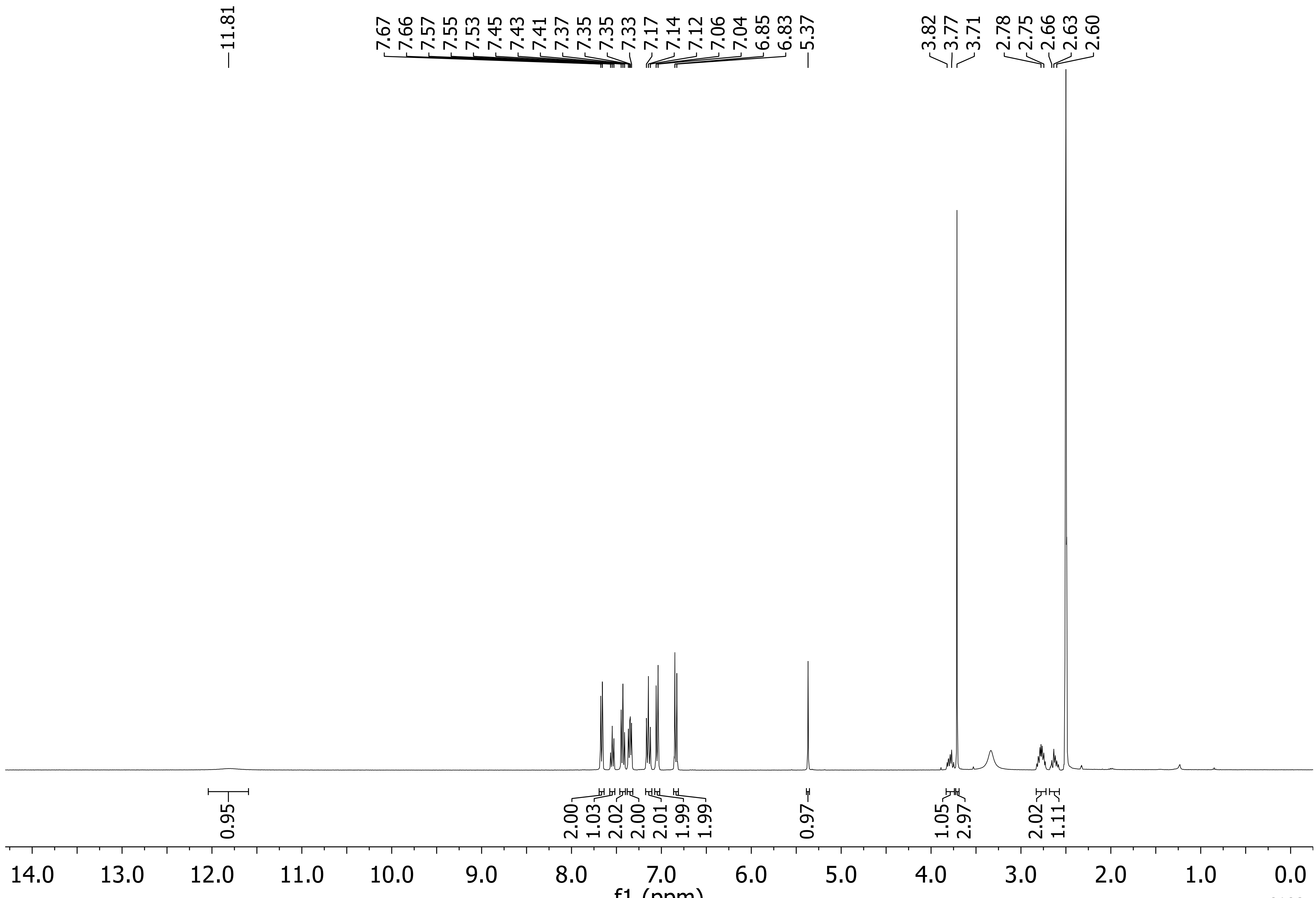

DEPTQ ^{13}C NMR (101 MHz, DMSO-d_6) for compound **49**

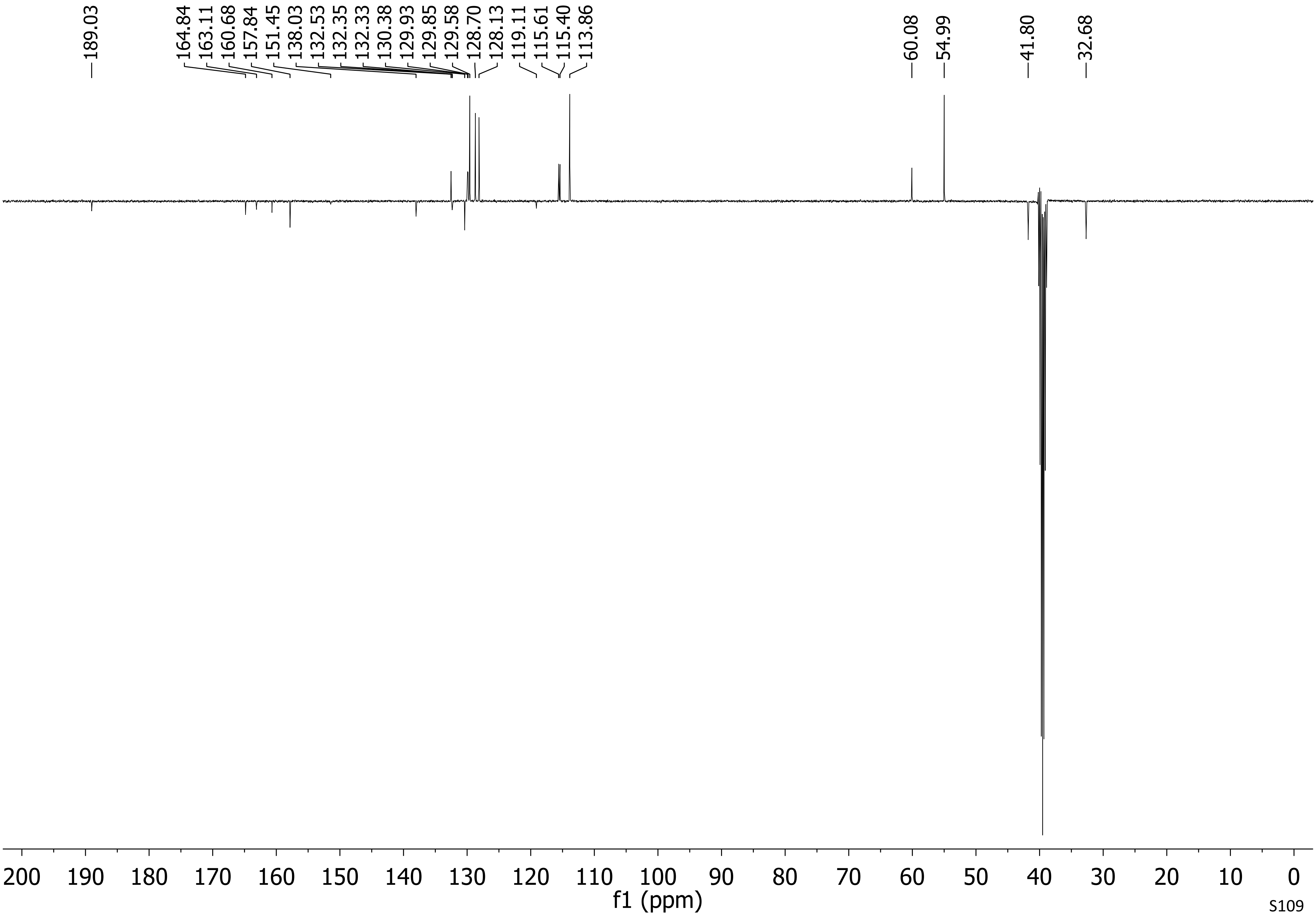

DEPTQ ^{13}C NMR (101 MHz, DMSO-d_6) for compound **49** (zoomed-in view)

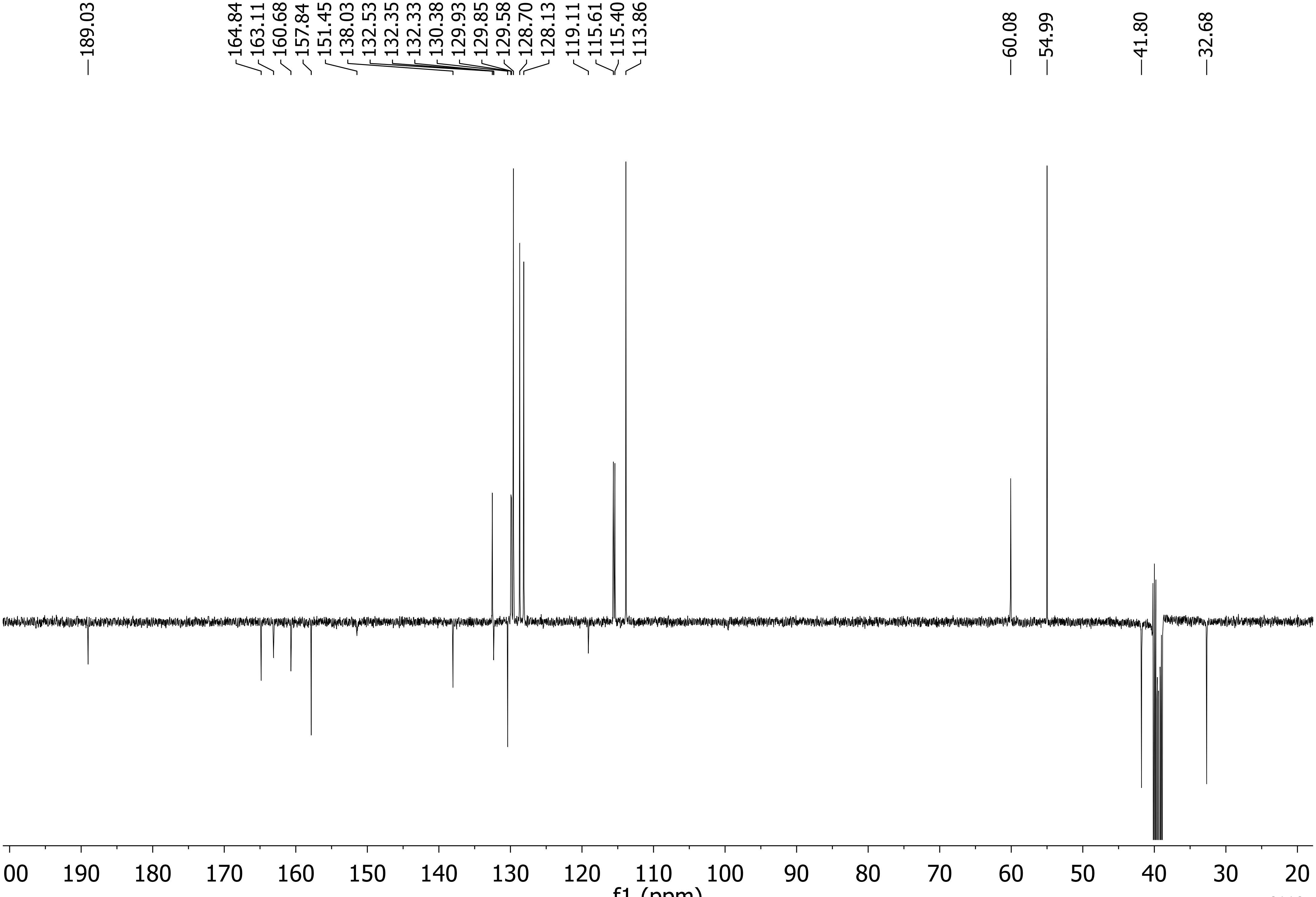

^{1}H NMR (400 MHz, DMSO-d_6) for compound **50a**

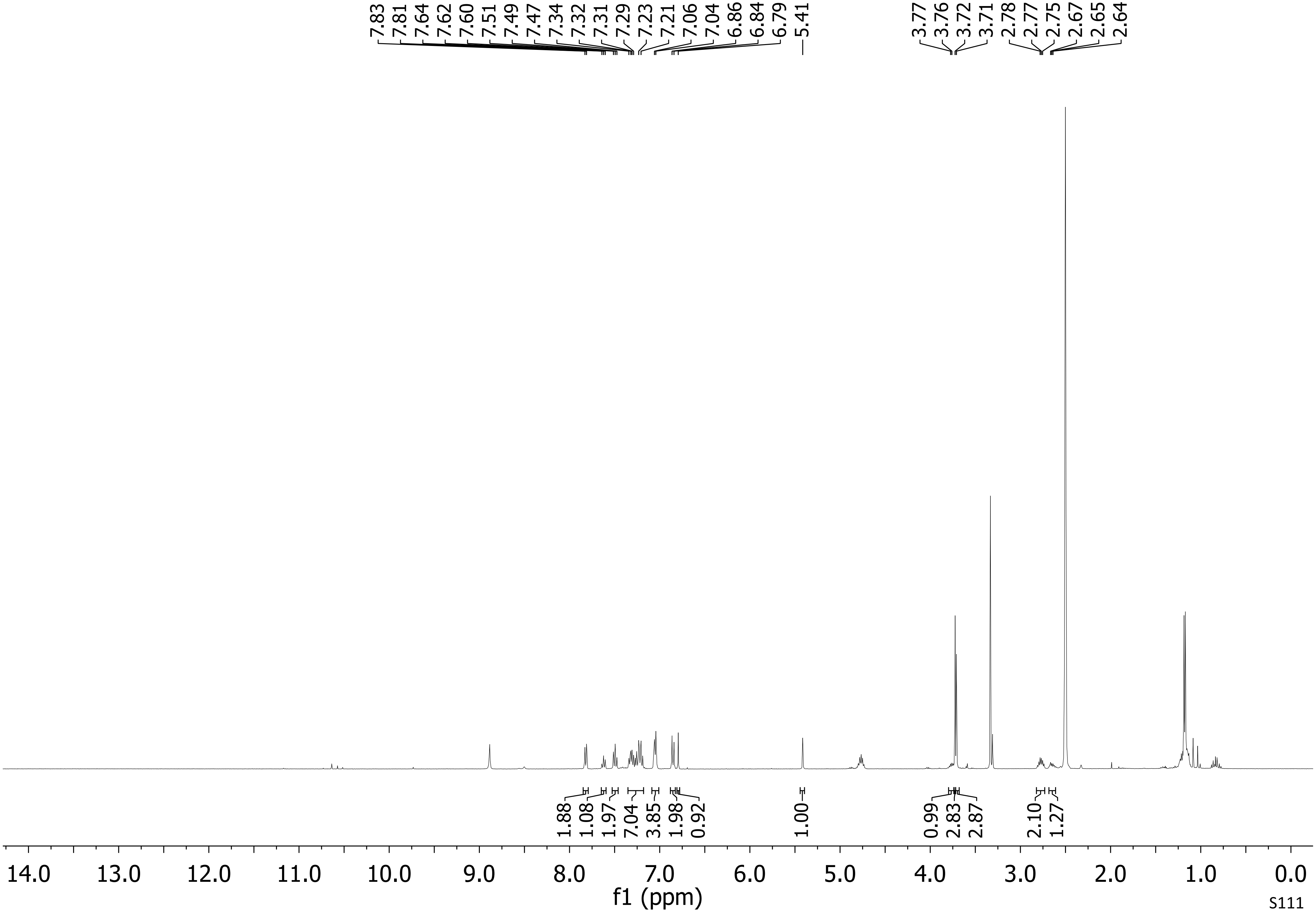

^{1}H NMR (400 MHz, DMSO-d_6) for compound **50a** (zoomed-in view)

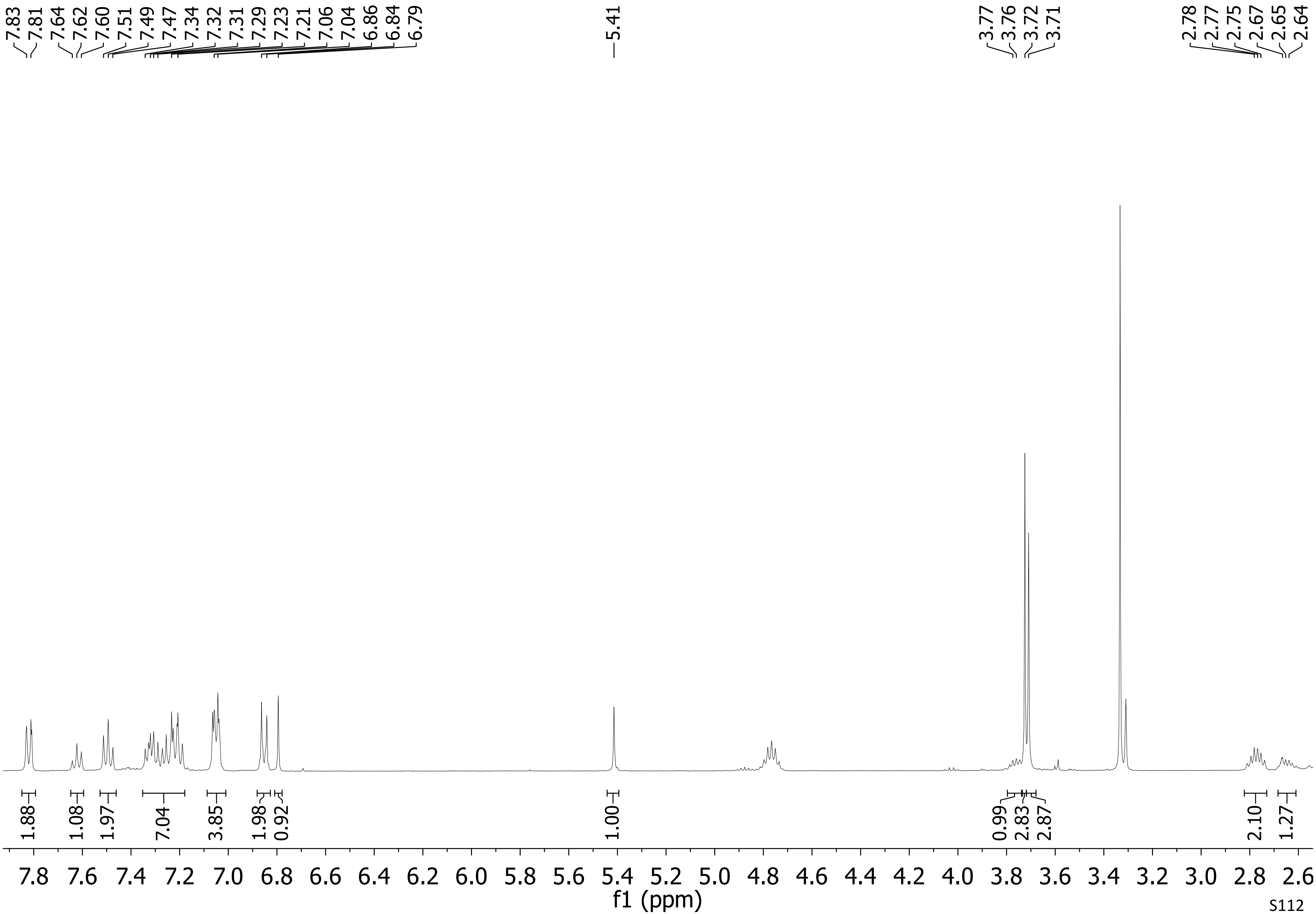

DEPTQ ^{13}C NMR (101 MHz, DMSO-d_6) for compound **50a**

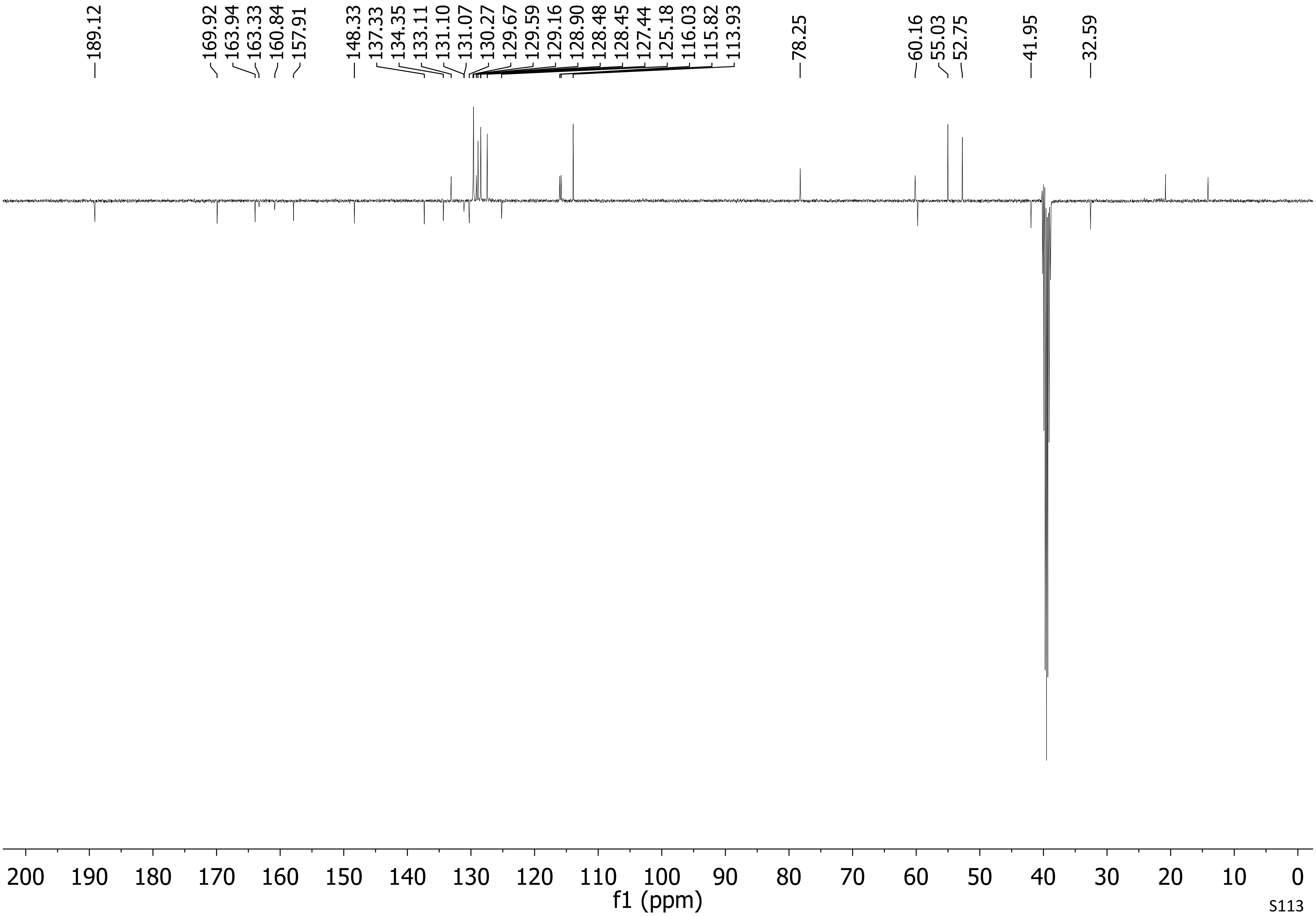

DEPTQ ^{13}C NMR (101 MHz, DMSO-d_6) for compound **50a** (zoomed-in view)

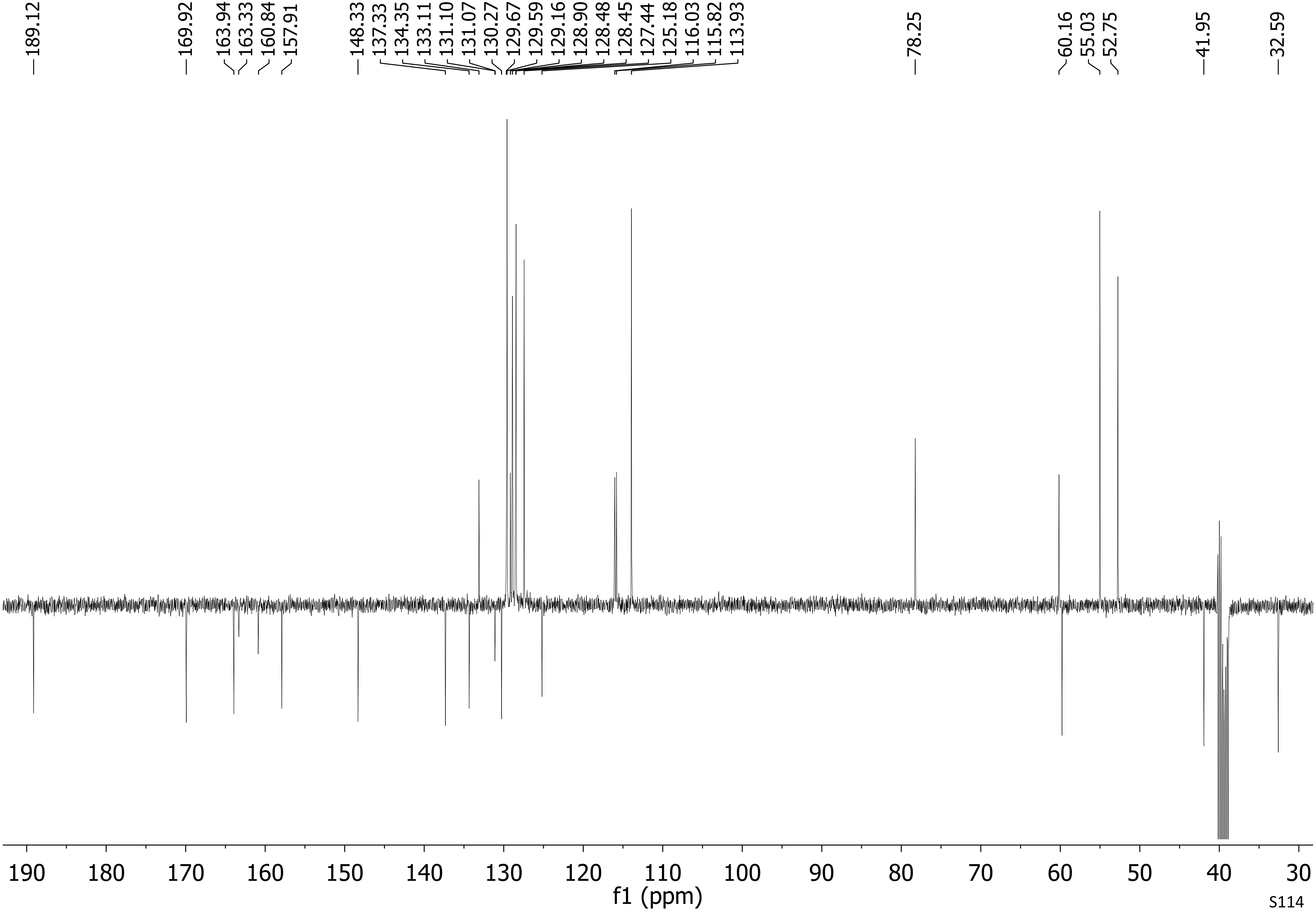

^{1}H NMR (400 MHz, DMSO-d_6) for compound **50b**

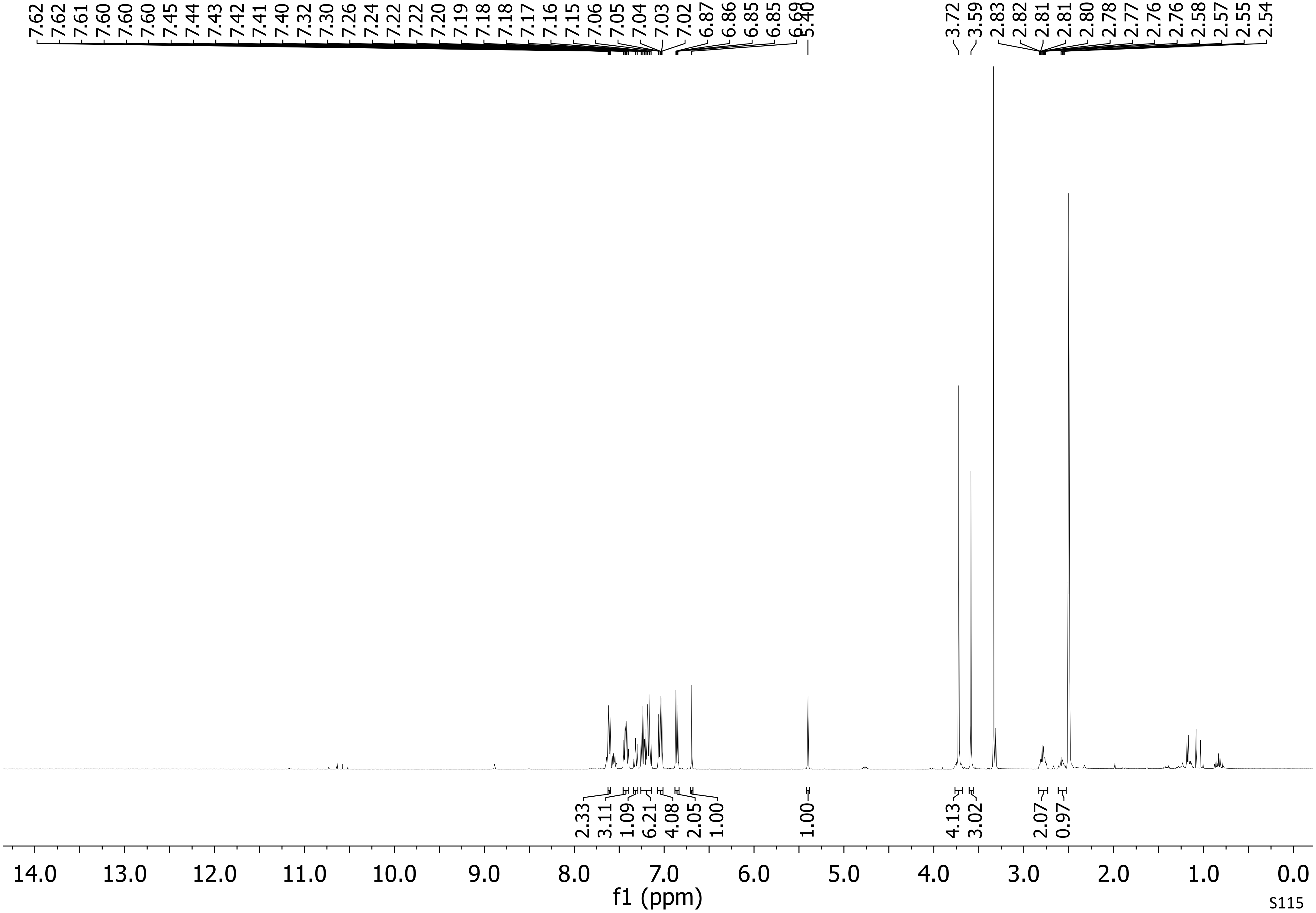

^{1}H NMR (400 MHz, DMSO-d_6) for compound **50b** (zoomed-in view)

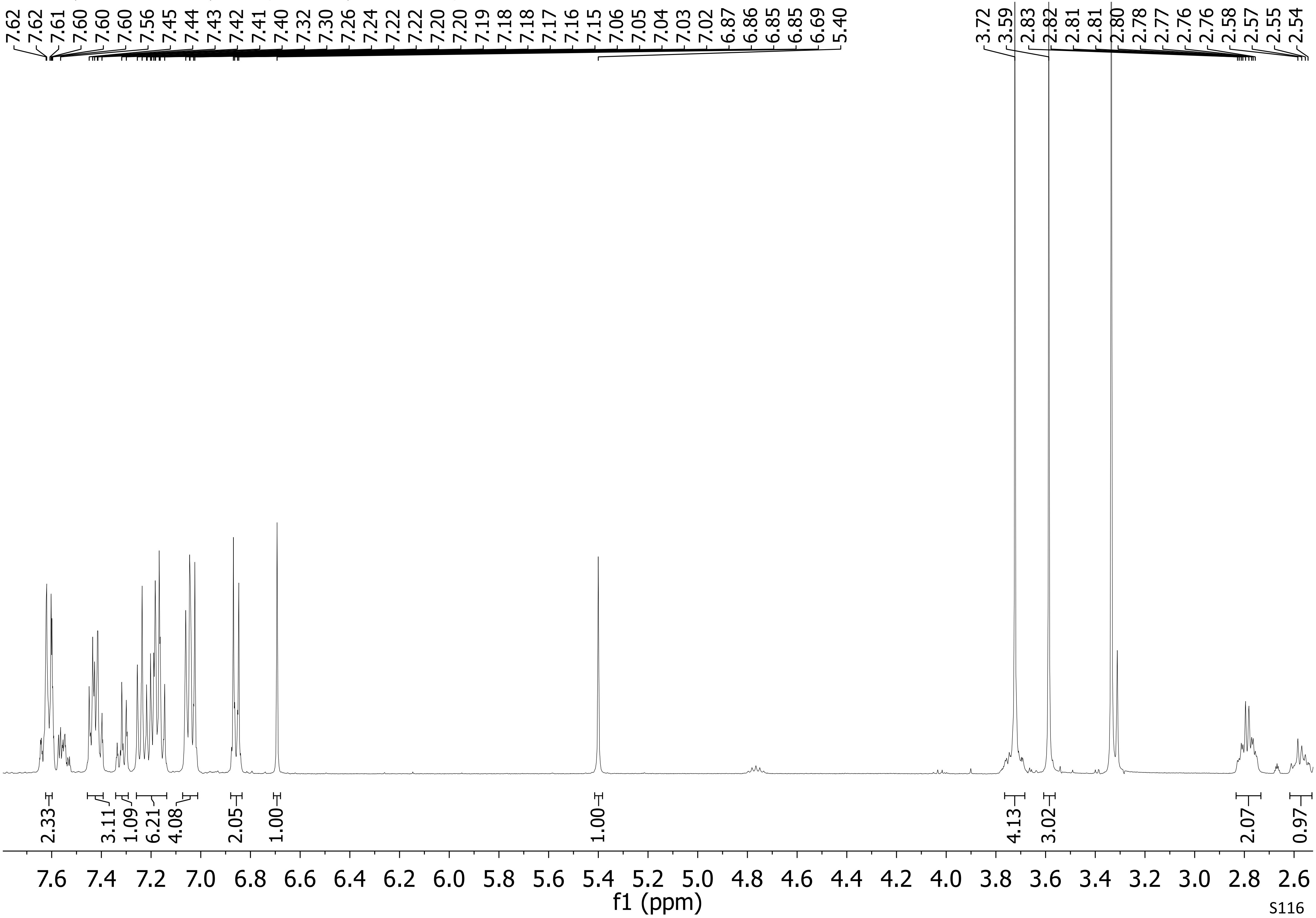

DEPTQ ^{13}C NMR (101 MHz, DMSO-d_6) for compound **50b**

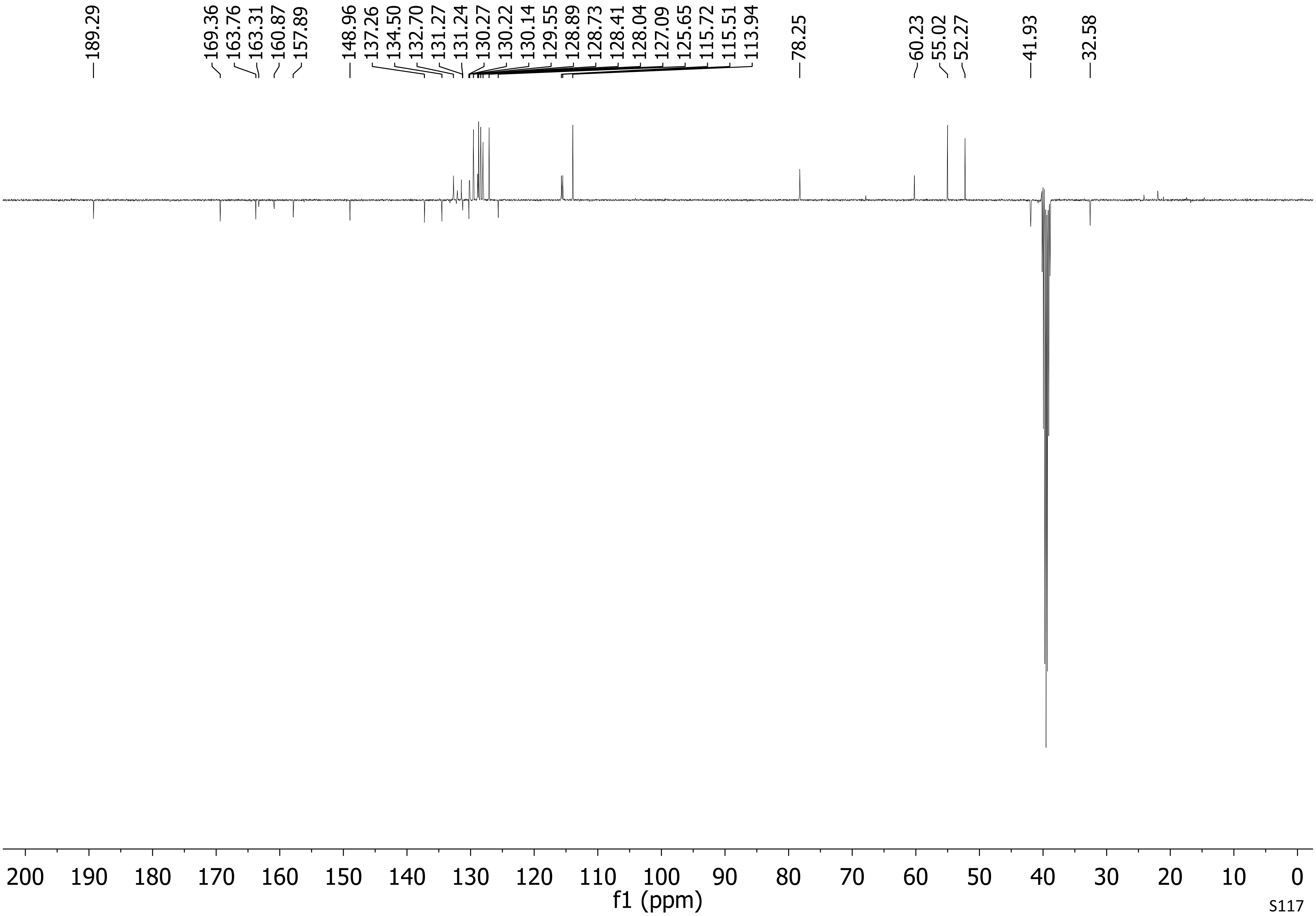

DEPTQ ^{13}C NMR (101 MHz, DMSO-d_6) for compound **50a** (zoomed-in view)

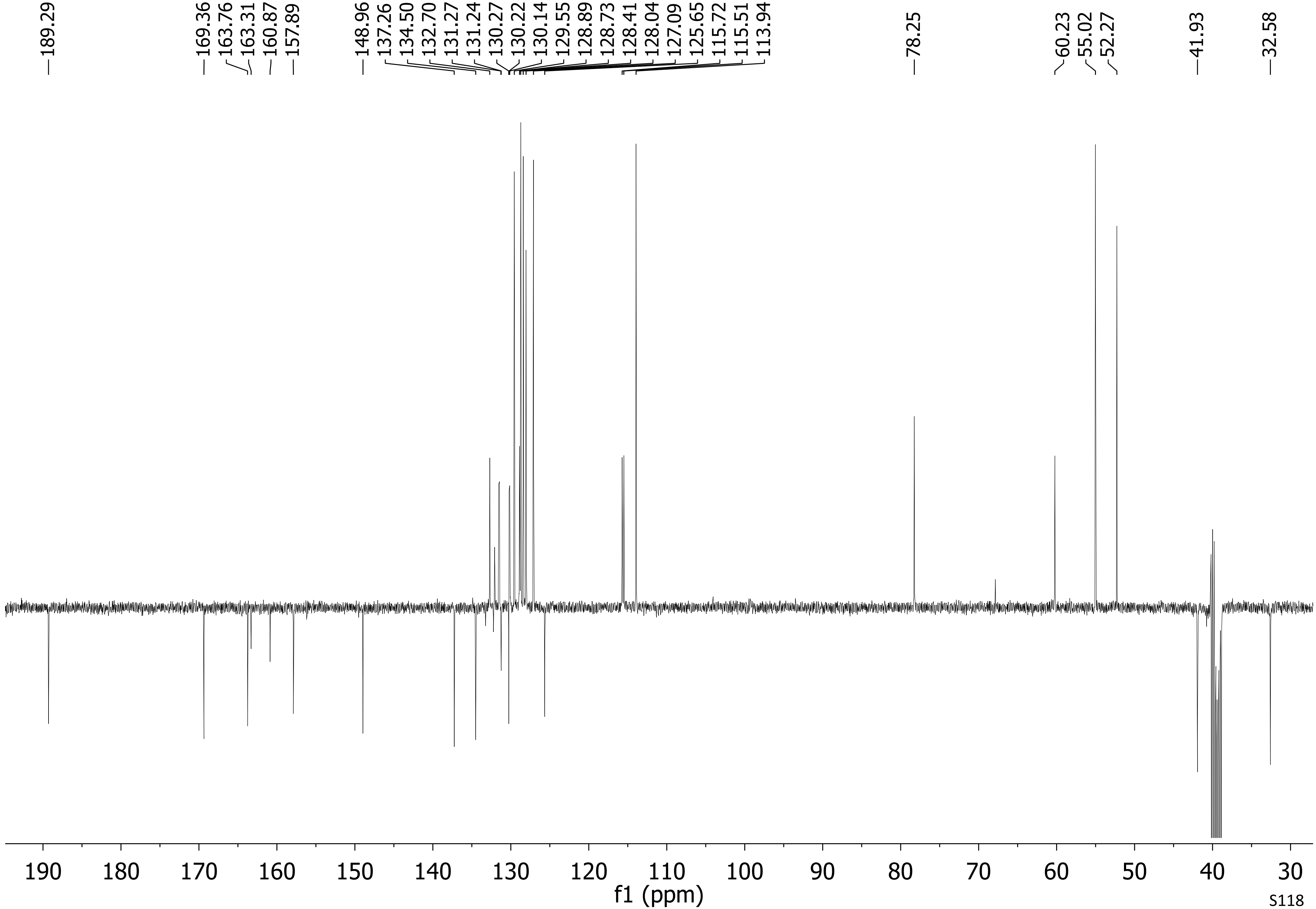

Supplementary HPLC Chromatograms

HPLC chromatogram of **10**:

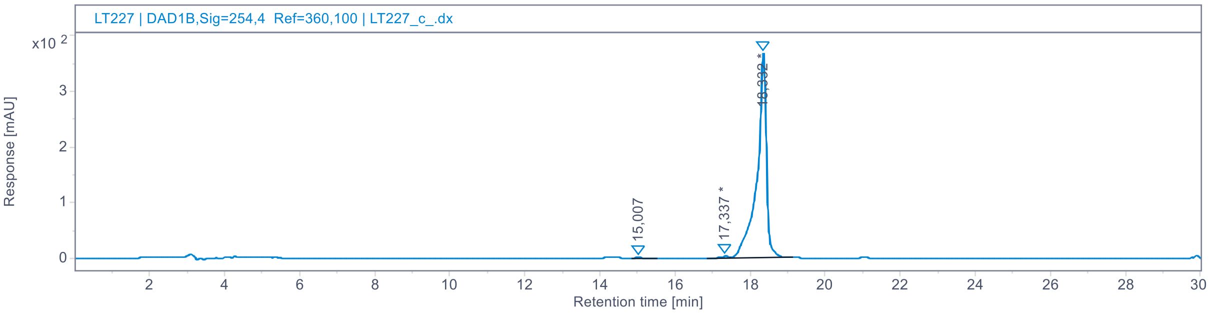

Signal: DAD1B,Sig=254,4 Ref=360,100

RT [min]	Type	Width [min]	Area	Height	Area%	Name
15.007	VB	0.67	24.25	2.19	0.37	
17.337	BM m	0.65	42.73	3.16	0.66	
18.332	MM m	1.66	6450.44	369.36	98.97	
		**Sum**	**6517.42**			

HPLC chromatogram of **11**:

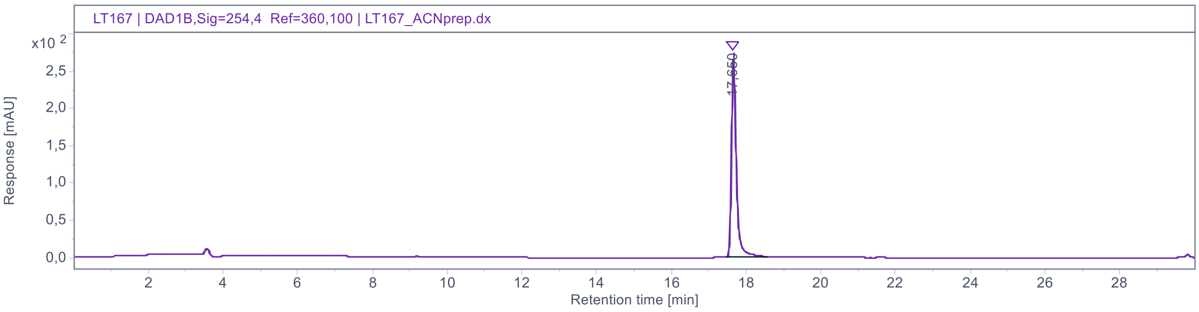

Signal: DAD1B,Sig=254,4 Ref=360,100

RT [min]	Type	Width [min]	Area	Height	Area%	Name
17.650	VB	1.12	2427.22	275.35	100.00	
		**Sum**	**2427.22**			

HPLC chromatogram of **12**:

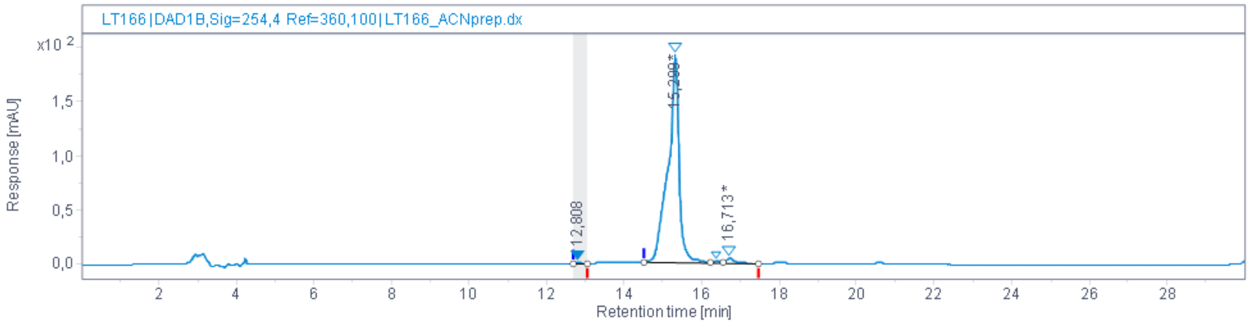

Signal: DAD1B,Sig=254,4 Ref=360,100

RT [min]	Type	Width [min]	Area	Height	Area%	Name
12.808	VB	0.37	18.21	1.95	0.47	
15.299	MM m	1.70	3772.15	192.61	97.46	
16.379	MM m	0.33	20.26	1.66	0.52	
16.713	MB m	0.91	59.98	4.27	1.55	
		**Sum**	**3870.60**			

HPLC chromatogram of **23**:

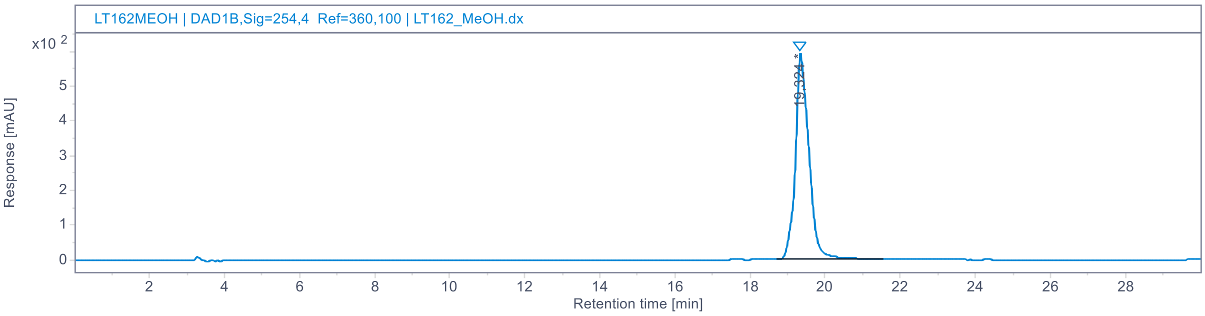

Signal: DAD1B,Sig=254,4 Ref=360,100

RT [min]	Type	Width [min]	Area	Height	Area%	Name
19.324	MV m	2.84	14900.32	594.71	100.00	
		**Sum**	**14900.32**			

HPLC chromatogram of (+)-**23**:

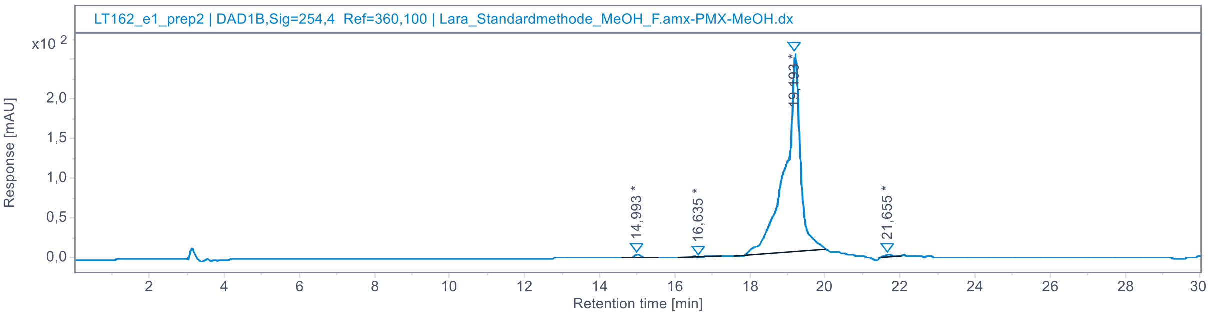

Signal: DAD1B,Sig=254,4 Ref=360,100

RT [min]	Type	Width [min]	Area	Height	Area%	Name
14.993	MM m	0.98	24.32	3.67	0.33	
16.635	MM n	1.17	6.35	0.90	0.09	
19.193	MM m	2.44	7237.19	250.96	99.29	
21.655	MM m	0.55	20.74	2.05	0.28	
		**Sum**	**7288.60**			

HPLC chromatogram of (-)-**23**:

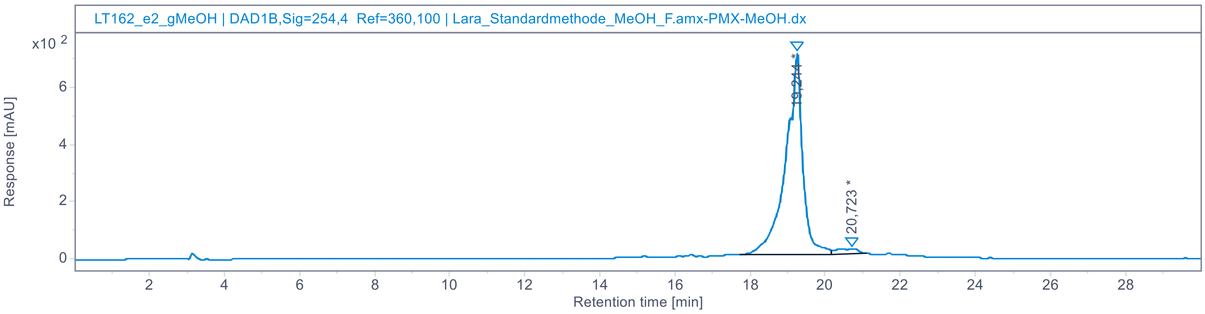

Signal: DAD1B,Sig=254,4 Ref=360,100

RT [min]	Type	Width [min]	Area	Height	Area%	Name
19.244	BV m	2.46	22444.44	708.73	96.73	
20.723	MM m	0.93	757.65	18.21	3.27	
		**Sum**	**23202.09**			

HPLC chromatogram of **24**:

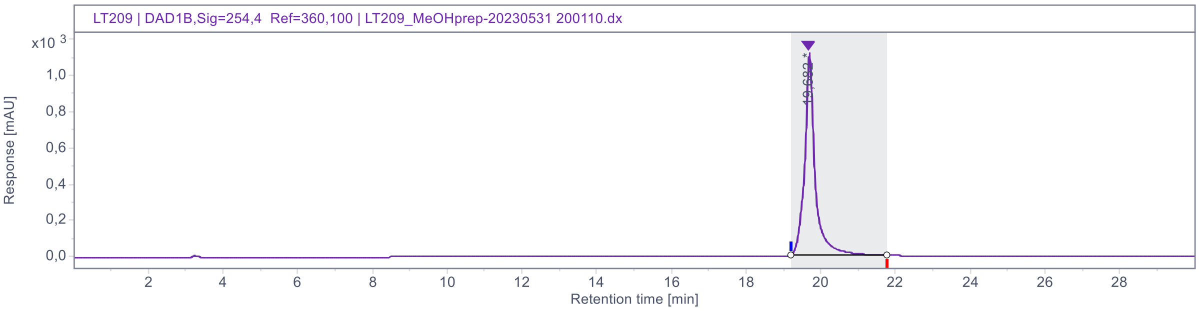

Signal: DAD1B,Sig=254,4 Ref=360,100

RT [min]	Type	Width [min]	Area	Height	Area%	Name
19.682	MM m	2.57	21639.74	1120.10	100.00	
		**Sum**	**21639.74**			

HPLC chromatogram of **25**:

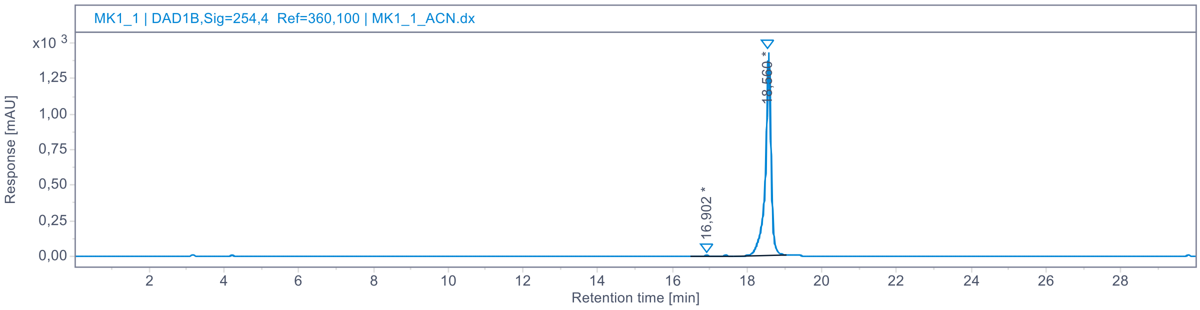

Signal:	DAD1B,Sig=254,4 Ref=360,100					
RT [min]	Type	Width [min]	Area	Height	Area%	Name
16.902	MM m	1.16	72.03	3.19	0.47	
18.560	MM m	1.41	15301.30	1435.52	99.53	
		**Sum**	**15373.34**			

HPLC chromatogram of **26**:

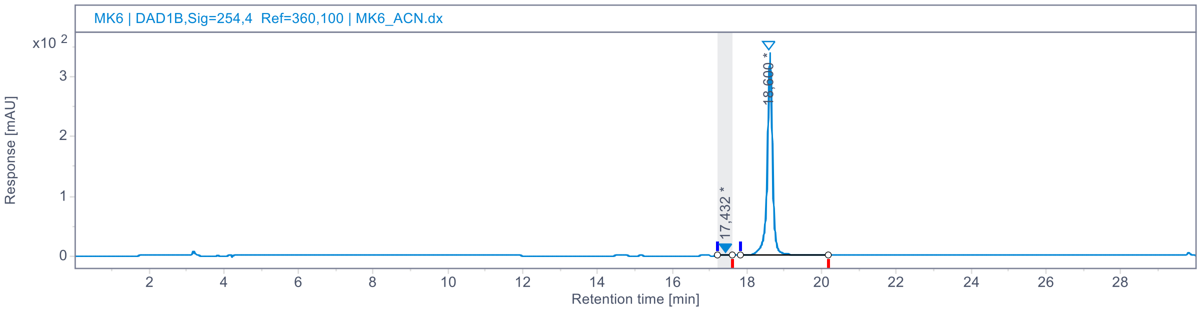

Signal: DAD1B,Sig=254,4 Ref=360,100

RT [min]	Type	Width [min]	Area	Height	Area%	Name
17.432	MM m	0.39	14.31	1.51	0.42	
18.600	MM m	2.32	3369.65	341.47	99.58	
		**Sum**	**3383.96**			

HPLC chromatogram of **27**:

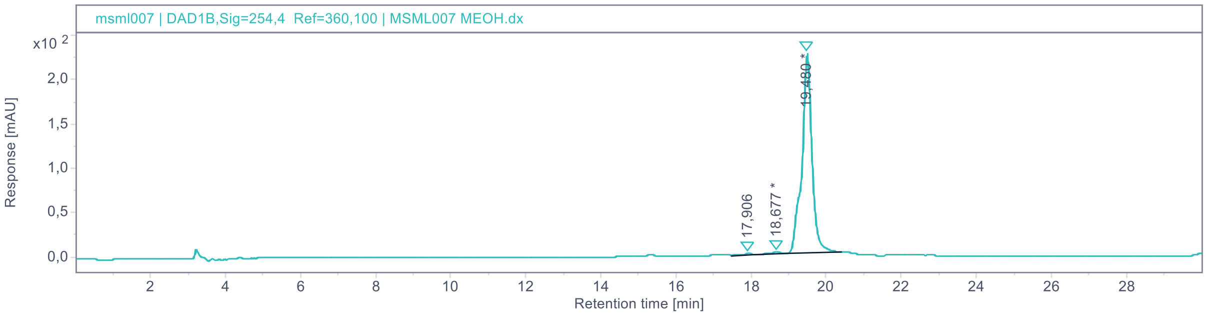

Signal: DAD1B,Sig=254,4 Ref=360,100

RT [min]	Type	Width [min]	Area	Height	Area%	Name
17.906	BB	0.58	24.07	2.17	0.58	
18.677	BM m	0.94	26.07	2.40	0.63	
19.480	MM m	1.13	4068.96	224.45	98.78	
		**Sum**	**4119.09**			

HPLC chromatogram of **28**:

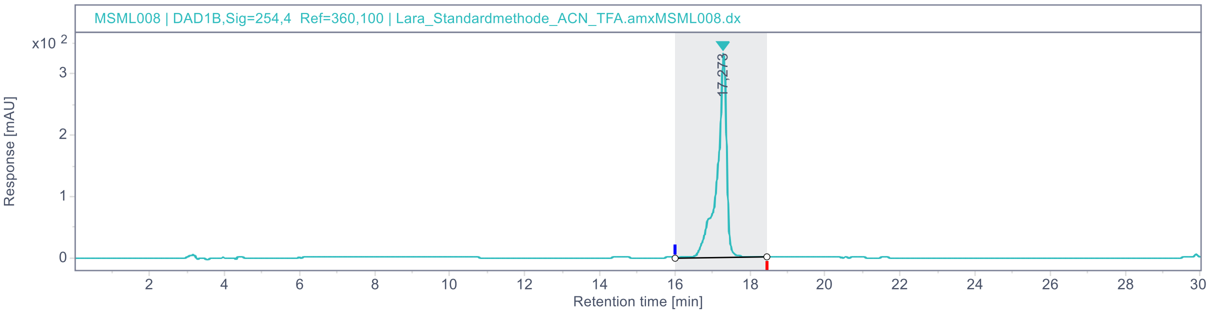

Signal:	DAD1B,Sig=254,4 Ref=360,100					
**RT [min]**	**Type**	**Width [min]**	**Area**	**Height**	**Area%**	**Name**
17.273	BB	2.47	5956.68	334.58	100.00	
		**Sum**	**5956.68**			

HPLC chromatogram of **29**:

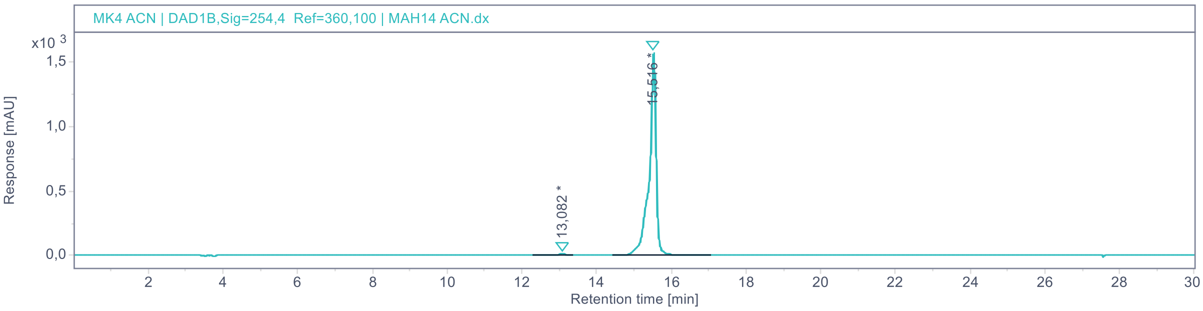

Signal: DAD1B,Sig=254,4 Ref=360,100

RT [min]	Type	Width [min]	Area	Height	Area%	Name
13.082	MM m	1.08	260.54	17.33	1.27	
15.516	MM m	2.67	20259.38	1578.04	98.73	
		**Sum**	**20519.91**			

HPLC chromatogram of **30**:

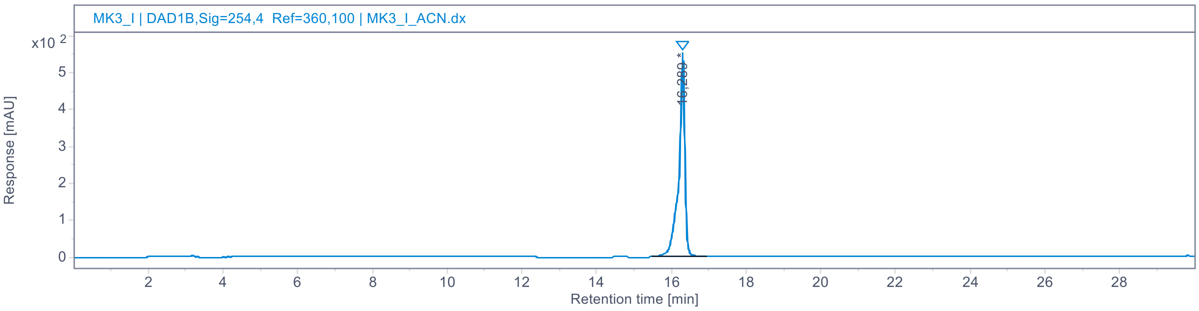

Signal: DAD1B,Sig=254,4 Ref=360,100

RT [min]	Type	Width [min]	Area	Height	Area%	Name
16.289	MM m	1.48	6222.01	555.22	100.00	
		**Sum**	**6222.01**			

HPLC chromatogram of **31**:

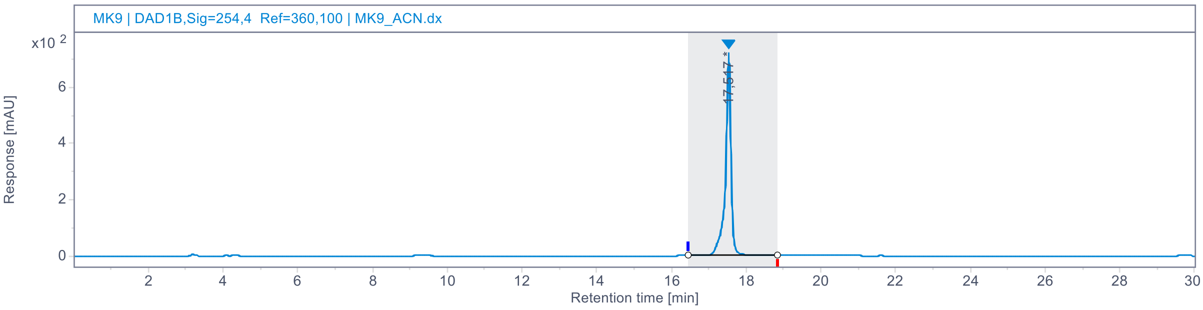

Signal: DAD1B,Sig=254,4 Ref=360,100

RT [min]	Type	Width [min]	Area	Height	Area%	Name
17.517	MM m	2.39	7286.11	724.81	100.00	
		**Sum**	**7286.11**			

HPLC chromatogram of **32**:

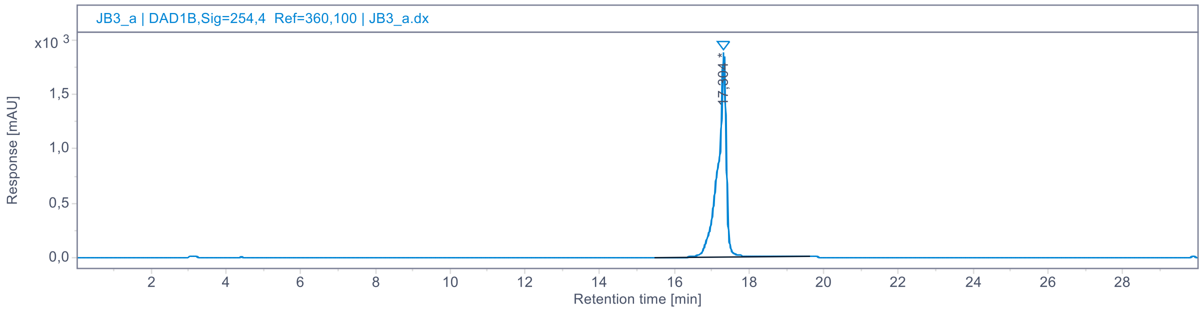

Signal: DAD1B,Sig=254,4 Ref=360,100

RT [min]	Type	Width [min]	Area	Height	Area%	Name
17.304	MM m	4.16	30101.92	1892.52	100.00	
		**Sum**	**30101.92**			

HPLC chromatogram of **33**:

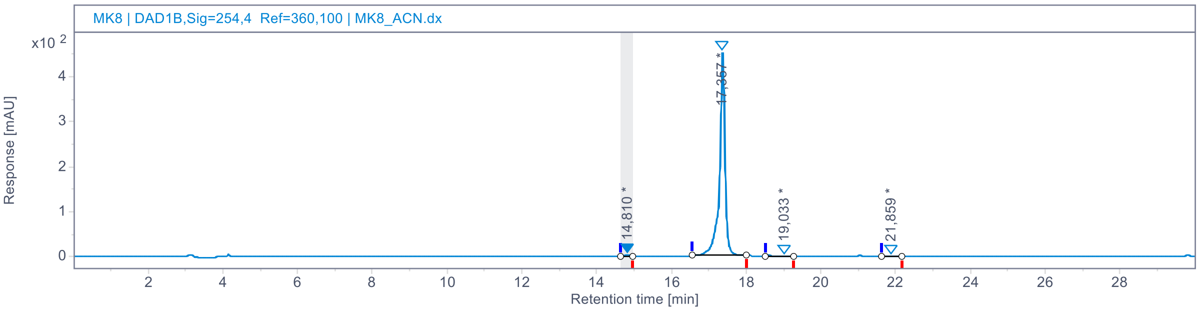

Signal: DAD1B,Sig=254,4 Ref=360,100

RT [min]	Type	Width [min]	Area	Height	Area%	Name
14.810	MM m	0.33	30.98	4.36	0.66	
17.357	MM m	1.45	4638.35	451.39	99.17	
19.033	MM n	0.76	5.83	0.51	0.12	
21.859	MM n	0.54	1.89	0.17	0.04	
		**Sum**	**4677.05**			

HPLC chromatogram of **34**:

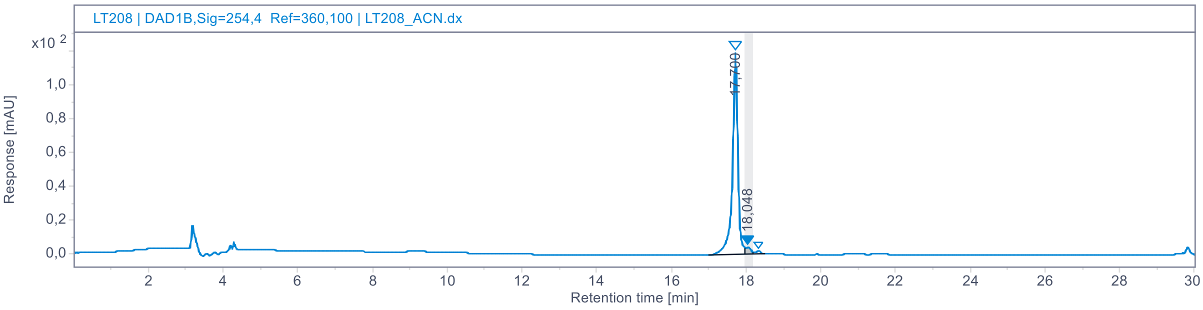

Signal: DAD1B,Sig=254,4 Ref=360,100

RT [min]	Type	Width [min]	Area	Height	Area%	Name
17.700	BV	1.01	1205.57	119.42	95.67	
18.048	VV	0.21	35.52	4.05	2.82	
18.318	VB	0.31	19.00	2.10	1.51	
		**Sum**	**1260.08**			

HPLC chromatogram of **35**:

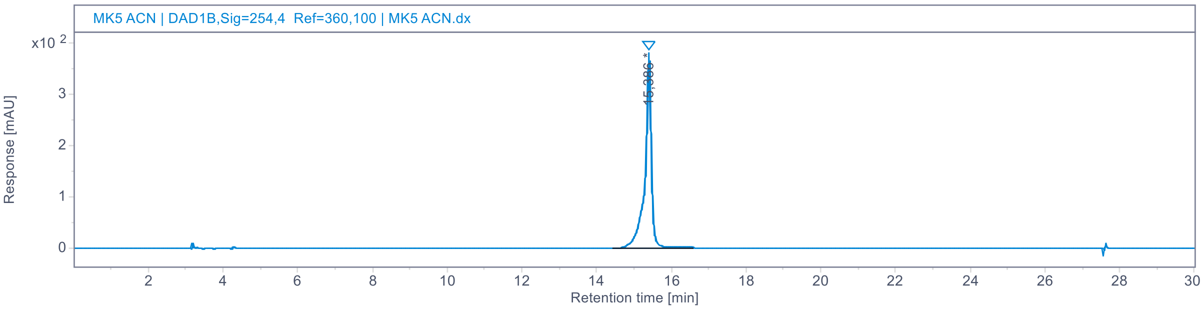

Signal: DAD1B,Sig=254,4 Ref=360,100

RT [min]	Type	Width [min]	Area	Height	Area%	Name
15.386	MM m	2.18	4457.20	382.49	100.00	
		**Sum**	**4457.20**			

HPLC chromatogram of **36**:

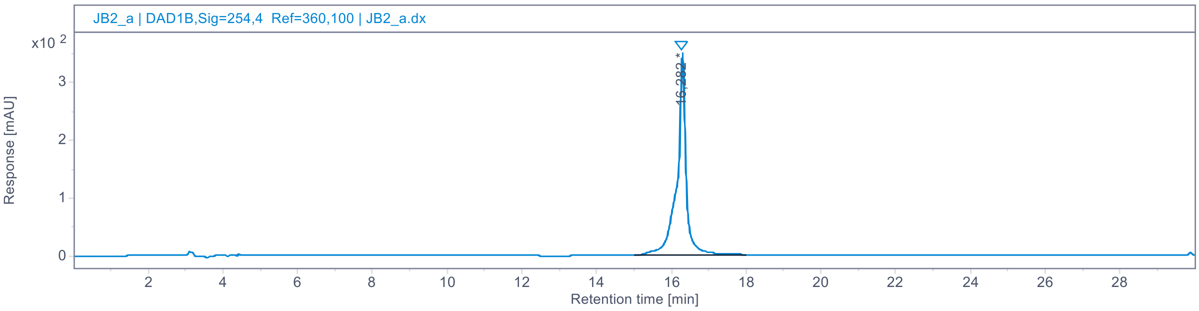

Signal:	DAD1B,Sig=254,4 Ref=360,100					
RT [min]	Type	Width [min]	Area	Height	Area%	Name
16.282	MM m	3.00	5959.17	352.19	100.00	
		**Sum**	**5959.17**			

HPLC chromatogram of **37**:

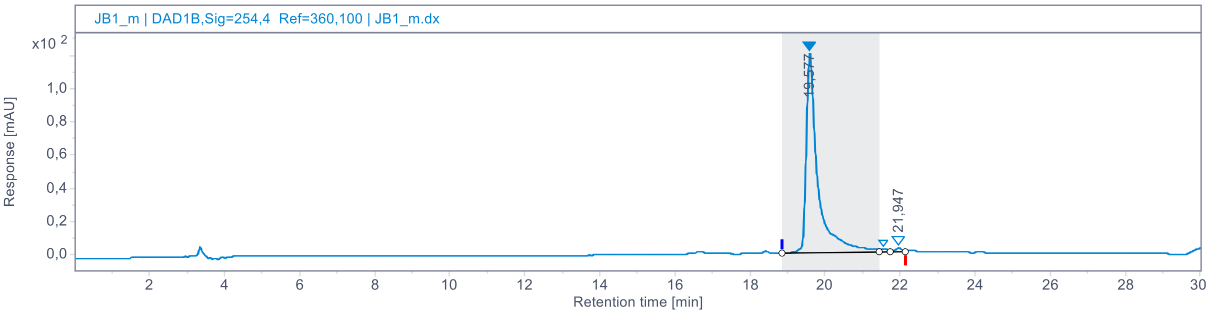

Signal: DAD1B,Sig=254,4 Ref=360,100

RT [min]	Type	Width [min]	Area	Height	Area%	Name
19.577	BV	2.60	2590.84	120.57	98.77	
21.947	VV	0.40	32.21	2.22	1.23	
		**Sum**	**2623.04**			

HPLC chromatogram of **38**:

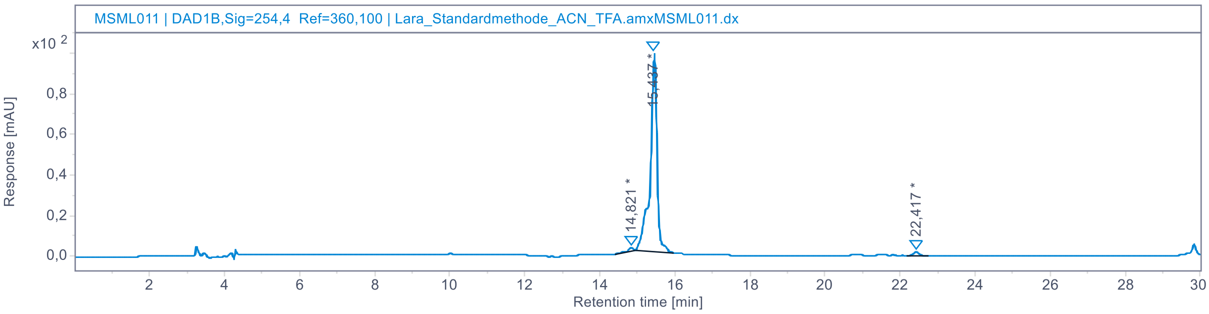

Signal: DAD1B,Sig=254,4 Ref=360,100

RT [min]	Type	Width [min]	Area	Height	Area%	Name
14.821	MM m	0.55	13.08	1.78	1.04	
15.437	MM m	1.02	1224.69	97.87	97.79	
22.417	MM m	0.58	14.65	1.58	1.17	
		Sum	1252.41			

HPLC chromatogram of **39**:

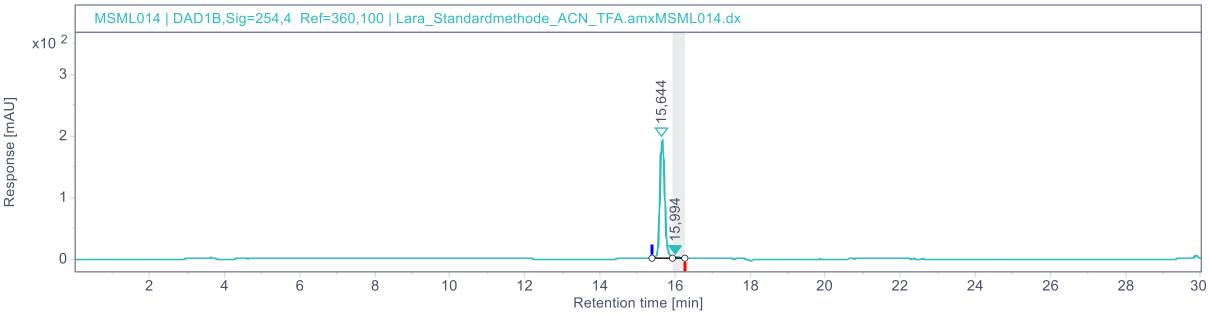

Signal: DAD1B,Sig=254,4 Ref=360,100

RT [min]	Type	Width [min]	Area	Height	Area%	Name
15.644	VV	0.56	1756.89	193.24	98.13	
15.994	VB	0.33	33.51	3.34	1.87	
		**Sum**	**1790.40**			

HPLC chromatogram of **40**:

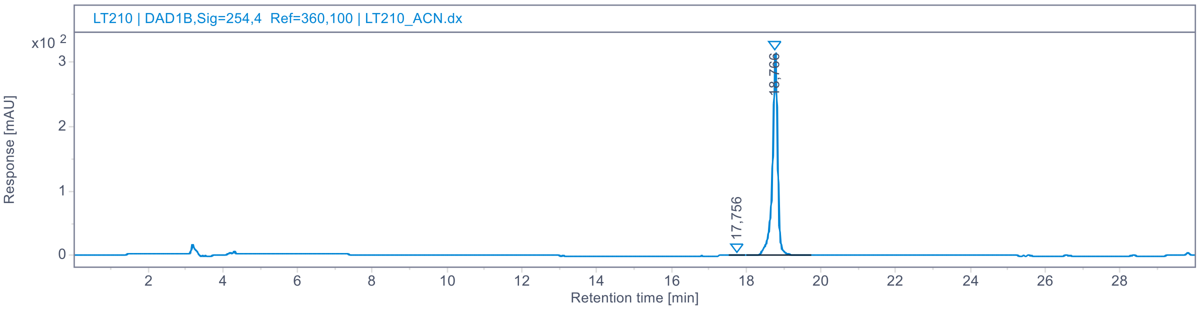

Signal: DAD1B,Sig=254,4 Ref=360,100

RT [min]	Type	Width [min]	Area	Height	Area%	Name
17.756	BB	0.44	15.14	1.77	0.49	
18.766	BB	1.76	3068.53	316.18	99.51	
		**Sum**	**3083.67**			

HPLC chromatogram of **49**:

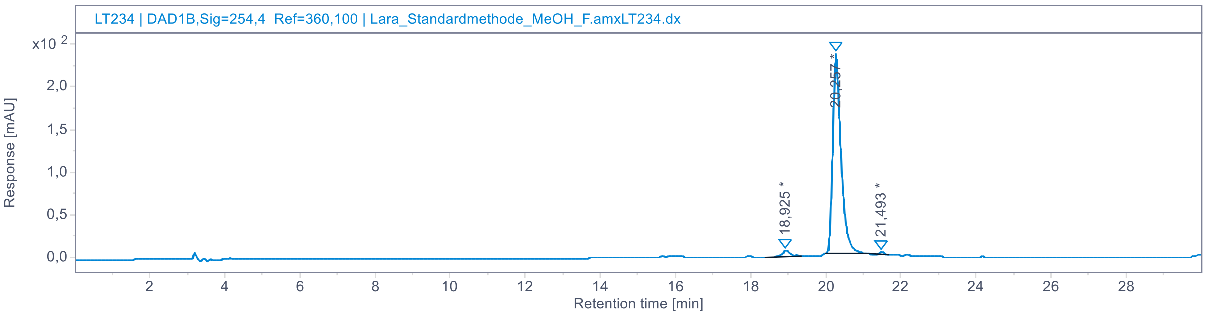

Signal: DAD1B,Sig=254,4 Ref=360,100

RT [min]	Type	Width [min]	Area	Height	Area%	Name
18.925	MM m	0.98	102.70	7.30	2.80	
20.257	MM m	1.09	3557.63	234.45	96.94	
21.493	MM m	0.62	9.58	2.53	0.26	
		**Sum**	**3669.91**			

HPLC chromatogram of **50a**:

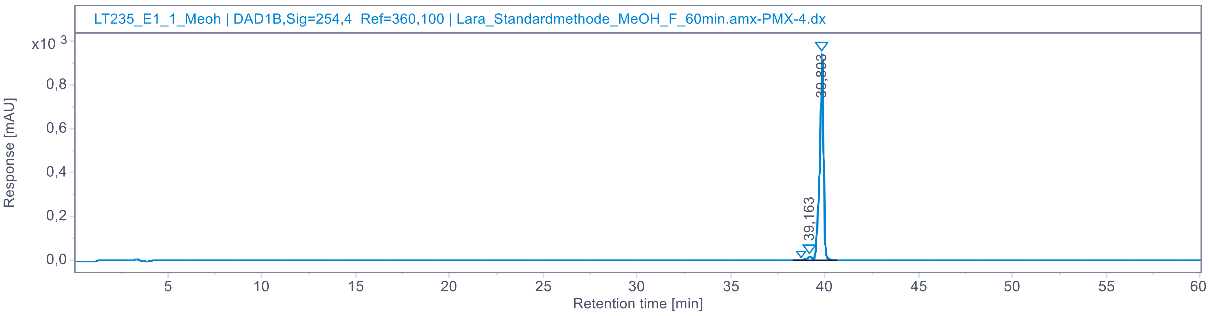

Signal: DAD1B,Sig=254,4 Ref=360,100

RT [min]	Type	Width [min]	Area	Height	Area%	Name
38.723	BV	0.46	27.10	2.66	0.20	
39.163	VV	0.57	325.84	21.18	2.40	
39.803	VV	1.28	13232.09	944.25	97.40	
		**Sum**	**13585.03**			

HPLC chromatogram of **50b**:

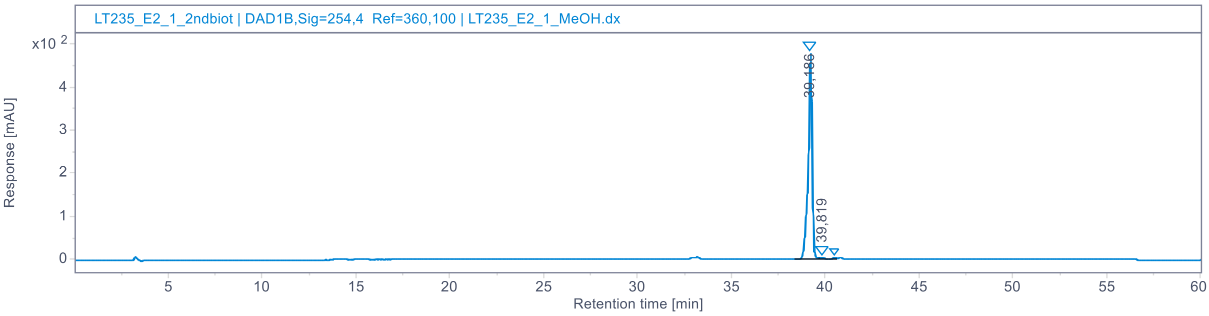

Signal: DAD1B,Sig=254,4 Ref=360,100

RT [min]	Type	Width [min]	Area	Height	Area%	Name
39.186	BV	1.26	6702.73	478.21	98.81	
39.819	VB	0.54	51.59	3.78	0.76	
40.481	BV	0.43	29.39	2.22	0.43	
		**Sum**	**6783.71**			

Supplementary References

1. Toy, L.; Huber, M. E.; Schmidt, M. F.; Weikert, D.; Schiedel, M., Fluorescent ligands targeting the intracellular allosteric binding site of the chemokine receptor CCR2. *ACS Chem. Biol.* **2022,** *17*, 2142-2152. DOI: 10.1021/acschembio.2c00263.
2. Mudd, G.; Pi, I. P.; Fethers, N.; Dodd, P. G.; Barbeau, O. R.; Auer, M., A general synthetic route to isomerically pure functionalized rhodamine dyes. *Methods Appl. Fluores.* **2015,** *3*, 045002. DOI: 10.1088/2050-6120/3/4/045002.
3. Huber, M. E.; Toy, L.; Schmidt, M. F.; Vogt, H.; Budzinski, J.; Wiefhoff, M. F. J.; Merten, N.; Kostenis, E.; Weikert, D.; Schiedel, M., A chemical biology toolbox targeting the intracellular binding site of CCR9: fluorescent ligands, new drug leads and PROTACs. *Angew. Chem. Int. Ed.* **2022,** *61*, e202116782. DOI: 10.1002/anie.202116782.
4. Yangthara, B.; Mills, A.; Chatsudthipong, V.; Tradtrantip, L.; Verkman, A. S., Small-molecule vasopressin-2 receptor antagonist identified by a g-protein coupled receptor "pathway" screen. *Mol. Pharmacol.* **2007,** *72*, 86-94. DOI: 10.1124/mol.107.034496.
5. Braga, R.; Hecquet, L.; Blonski, C., Slow-binding inhibition of 2-keto-3-deoxy-6-phosphogluconate (KDPG) aldolase. *Bioorg. Med. Chem.* **2004,** *12*, 2965-2972. DOI: 10.1016/j.bmc.2004.03.039.
6. Nagarapu, L.; Gaikwad, H. K.; Sarikonda, K.; Mateti, J.; Bantu, R.; Raghu, P. S.; Manda, K. M.; Kalvendi, S. V., Synthesis and cytotoxicity evaluation of 1-[3-(9H-carbazol-4-yloxy)-2-hydroxypropyl]-3-aryl-1H-pyrazole-5-carboxylic acid derivatives. *Eur. J. Med. Chem.* **2010,** *45*, 4720-4725. DOI: 10.1016/j.ejmech.2010.07.004.
7. Tabrizi, M. A.; Baraldi, P. G.; Ruggiero, E.; Saponaro, G.; Baraldi, S.; Romagnoli, R.; Martinelli, A.; Tuccinardi, T., Pyrazole phenylcyclohexylcarbamates as inhibitors of human fatty acid amide hydrolases (FAAH). *Eur. J. Med. Chem.* **2015,** *97*, 289-305. DOI: 10.1016/j.ejmech.2015.04.064.
8. Huber, M. E.; Wurnig, S.; Toy, L.; Weiler, C.; Merten, N.; Kostenis, E.; Hansen, F. K.; Schiedel, M., Fluorescent ligands enable target engagement studies for the intracellular allosteric binding site of the chemokine receptor CXCR2. *J. Med. Chem.* **2023,** *66*, 9916-9933. DOI: 10.1021/acs.jmedchem.3c00769.
9. Lowry, O. H.; Rosebrough, N. J.; Farr, A. L.; Randall, R. J., Protein measurement with the Folin phenol reagent. *J. Biol. Chem.* **1951,** *193*, 265-275.
10. Gibson, D. G.; Young, L.; Chuang, R. Y.; Venter, J. C.; Hutchison, C. A., 3rd; Smith, H. O., Enzymatic assembly of DNA molecules up to several hundred kilobases. *Nat. Methods* **2009,** *6*, 343-345. DOI: 10.1038/nmeth.1318.
11. Hall, M. P.; Unch, J.; Binkowski, B. F.; Valley, M. P.; Butler, B. L.; Wood, M. G.; Otto, P.; Zimmerman, K.; Vidugiris, G.; Machleidt, T.; Robers, M. B.; Benink, H. A.; Eggers, C. T.; Slater, M. R.; Meisenheimer, P. L.; Klaubert, D. H.; Fan, F.; Encell, L. P.; Wood, K. V., Engineered luciferase reporter from a deep sea shrimp utilizing a novel imidazopyrazinone substrate. *ACS Chem. Biol.* **2012,** *7*, 1848-1857. DOI: 10.1021/cb3002478.
12. Allikalt, A.; Purkayastha, N.; Flad, K.; Schmidt, M. F.; Tabor, A.; Gmeiner, P.; Hübner, H.; Weikert, D., Fluorescent ligands for dopamine D2/D3 receptors. *Sci. Rep.* **2020,** *10*, 21842. DOI: 10.1038/s41598-020-78827-9.
13. Ortiz Zacarias, N. V.; van Veldhoven, J. P. D.; Portner, L.; van Spronsen, E.; Ullo, S.; Veenhuizen, M.; van der Velden, W. J. C.; Zweemer, A. J. M.; Kreekel, R. M.; Oenema, K.; Lenselink, E. B.; Heitman, L. H.; AP, I. J., Pyrrolone derivatives as intracellular allosteric modulators for chemokine receptors: selective and dual-targeting inhibitors of CC chemokine receptors 1 and 2. *J. Med. Chem.* **2018,** *61*, 9146-9161. DOI: 10.1021/acs.jmedchem.8b00605.
14. Walters, M. J.; Wang, Y.; Lai, N.; Baumgart, T.; Zhao, B. N.; Dairaghi, D. J.; Bekker, P.; Ertl, L. S.; Penfold, M. E.; Jaen, J. C.; Keshav, S.; Wendt, E.; Pennell, A.; Ungashe, S.; Wei, Z.; Wright, J. J.; Schall, T. J., Characterization of CCX282-B, an orally bioavailable antagonist of the CCR9 chemokine receptor, for treatment of inflammatory bowel disease. *J. Pharmacol. Exp. Ther.* **2010,** *335*, 61-69. DOI: 10.1124/jpet.110.169714.

15. Oswald, C.; Rappas, M.; Kean, J.; Dore, A. S.; Errey, J. C.; Bennett, K.; Deflorian, F.; Christopher, J. A.; Jazayeri, A.; Mason, J. S.; Congreve, M.; Cooke, R. M.; Marshall, F. H., Intracellular allosteric antagonism of the CCR9 receptor. *Nature* **2016,** *540*, 462-465. DOI: 10.1038/nature20606.
16. Dwyer, M. P.; Yu, Y.; Chao, J.; Aki, C.; Chao, J.; Biju, P.; Girijavallabhan, V.; Rindgen, D.; Bond, R.; Mayer-Ezel, R.; Jakway, J.; Hipkin, R. W.; Fossetta, J.; Gonsiorek, W.; Bian, H.; Fan, X.; Terminelli, C.; Fine, J.; Lundell, D.; Merritt, J. R.; Rokosz, L. L.; Kaiser, B.; Li, G.; Wang, W.; Stauffer, T.; Ozgur, L.; Baldwin, J.; Taveras, A. G., Discovery of 2-hydroxy-N,N-dimethyl-3-{2-[[(R)-1-(5-methylfuran-2-yl)propyl]amino]-3,4-dioxocyclobut-1-enylamino}benzamide (SCH 527123): a potent, orally bioavailable CXCR2/CXCR1 receptor antagonist. *J. Med. Chem.* **2006,** *49*, 7603-7606. DOI: 10.1021/jm0609622.
17. Luis, R.; D'Uonnolo, G.; Palmer, C. B.; Meyrath, M.; Uchanski, T.; Wantz, M.; Rogister, B.; Janji, B.; Chevigne, A.; Szpakowska, M., Nanoluciferase-based methods to monitor activation, modulation and trafficking of atypical chemokine receptors. *Methods Cell Biol.* **2022,** *169*, 279-294. DOI: 10.1016/bs.mcb.2022.03.002.
18. Meyrath, M.; Szpakowska, M.; Zeiner, J.; Massotte, L.; Merz, M. P.; Benkel, T.; Simon, K.; Ohnmacht, J.; Turner, J. D.; Kruger, R.; Seutin, V.; Ollert, M.; Kostenis, E.; Chevigne, A., The atypical chemokine receptor ACKR3/CXCR7 is a broad-spectrum scavenger for opioid peptides. *Nat. Commun.* **2020,** *11*, 3033. DOI: 10.1038/s41467-020-16664-0.
19. Dixon, A. S.; Schwinn, M. K.; Hall, M. P.; Zimmerman, K.; Otto, P.; Lubben, T. H.; Butler, B. L.; Binkowski, B. F.; Machleidt, T.; Kirkland, T. A.; Wood, M. G.; Eggers, C. T.; Encell, L. P.; Wood, K. V., NanoLuc complementation reporter optimized for accurate measurement of protein interactions in cells. *ACS Chem. Biol.* **2016,** *11*, 400-408. DOI: 10.1021/acschembio.5b00753.
20. Nehme, R.; Carpenter, B.; Singhal, A.; Strege, A.; Edwards, P. C.; White, C. F.; Du, H.; Grisshammer, R.; Tate, C. G., Mini-G proteins: Novel tools for studying GPCRs in their active conformation. *PLoS One* **2017,** *12*, e0175642. DOI: 10.1371/journal.pone.0175642.
21. Zheng, Y.; Qin, L.; Zacarias, N. V.; de Vries, H.; Han, G. W.; Gustavsson, M.; Dabros, M.; Zhao, C.; Cherney, R. J.; Carter, P.; Stamos, D.; Abagyan, R.; Cherezov, V.; Stevens, R. C.; AP, I. J.; Heitman, L. H.; Tebben, A.; Kufareva, I.; Handel, T. M., Structure of CC chemokine receptor 2 with orthosteric and allosteric antagonists. *Nature* **2016,** *540*, 458-461. DOI: 10.1038/nature20605.
22. El-Zohairy, M. A.; Zlotos, D. P.; Berger, M. R.; Adwan, H. H.; Mandour, Y. M., Discovery of novel CCR5 ligands as anticolorectal cancer agents by sequential virtual screening. *ACS Omega* **2021,** *6*, 10921-10935. DOI: 10.1021/acsomega.1c00681.
23. http://www.specs.net, (accessed September 3, 2021).
24. *Molecular Operating Environment (MOE)*, 2019.01; Chemical Computing Group ULC, 910-1010 Sherbrooke St. W., Montreal, QC H3A 2R7, Canada: 2019.
25. Jones, G.; Willett, P.; Glen, R. C., Molecular recognition of receptor sites using a genetic algorithm with a description of desolvation. *J. Mol. Biol.* **1995,** *245*, 43-53. DOI: 10.1016/s0022-2836(95)80037-9.
26. Jones, G.; Willett, P.; Glen, R. C.; Leach, A. R.; Taylor, R., Development and validation of a genetic algorithm for flexible docking. *J. Mol. Biol.* **1997,** *267*, 727-748. DOI: 10.1006/jmbi.1996.0897.
27. Kooistra, A. J.; Mordalski, S.; Pandy-Szekeres, G.; Esguerra, M.; Mamyrbekov, A.; Munk, C.; Keseru, G. M.; Gloriam, D. E., GPCRdb in 2021: integrating GPCR sequence, structure and function. *Nucleic Acids Res.* **2021,** *49*, D335-D343. DOI: 10.1093/nar/gkaa1080.
28. Isberg, V.; Mordalski, S.; Munk, C.; Rataj, K.; Harpsoe, K.; Hauser, A. S.; Vroling, B.; Bojarski, A. J.; Vriend, G.; Gloriam, D. E., GPCRdb: an information system for G protein-coupled receptors. *Nucleic Acids Res.* **2016,** *44*, D356-364. DOI: 10.1093/nar/gkv1178.
29. Hanwell, M. D.; Curtis, D. E.; Lonie, D. C.; Vandermeersch, T.; Zurek, E.; Hutchison, G. R., Avogadro: an advanced semantic chemical editor, visualization, and analysis platform. *J. Cheminform.* **2012,** *4*, 17. DOI: 10.1186/1758-2946-4-17.
30. Eberhardt, J.; Santos-Martins, D.; Tillack, A. F.; Forli, S., AutoDock Vina 1.2.0: new docking methods, expanded force field, and python bindings. *J. Chem. Inf. Model.* **2021,** *61*, 3891-3898. DOI: 10.1021/acs.jcim.1c00203.

31. Trott, O.; Olson, A. J., AutoDock Vina: improving the speed and accuracy of docking with a new scoring function, efficient optimization, and multithreading. *J. Comput. Chem.* **2010,** *31*, 455-461. DOI: 10.1002/jcc.21334.
32. Jakalian, A.; Bush, B. L.; Jack, D. B.; Bayly, C. I., Fast, efficient generation of high‐quality atomic charges. AM1‐BCC model: I. Method. *J. Comput. Chem.* **2002,** *21*, 132-146. DOI: 10.1002/jcc.10128.
33. Darden, T.; York, D.; Pedersen, L., Particle Mesh Ewald - an N log(N) method for Ewald sums in large systems. *J. Chem. Phys.* **1993,** *98*, 10089-10092. DOI: 10.1063/1.464397.
34. Case, D. A.; Aktulga, H. M.; Belfon, K.; Ben-Shalom, I. Y.; Berryman, J. T.; Brozell, S. R.; Cerutti, D. S.; Cheatham, I., T.E. ; Cisneros, G. A.; Cruzeiro, V. W. D.; Darden, T. A.; Forouzesh, N.; Giambaşu, G.; Giese, T.; Gilson, M. K.; Gohlke, H.; Goetz, A. W.; Harris, J.; Izadi, S.; Izmailov, S. A.; Kasavajhala, K.; Kaymak, M. C.; King, E.; Kovalenko, A.; Kurtzman, T.; Lee, T. S.; Li, P.; Lin, C.; Liu, J.; Luchko, T.; Luo, R.; Machado, M.; Man, V.; Manathunga, M.; Merz, K. M.; Miao, Y.; Mikhailovskii, O.; Monard, G.; Nguyen, H.; O'Hearn, K. A.; Onufriev, A.; Pan, F.; Pantano, S.; Qi, R.; Rahnamoun, A.; Roe, D. R.; Roitberg, A.; Sagui, C.; Schott-Verdugo, S.; Shajan, A.; Shen, J.; Simmerling, C. L.; Skrynnikov, N. R.; Smith, J.; Swails, J.; Walker, R. C.; Wang, J.; Wang, J.; Wei, H.; Wu, X.; Wu, Y.; Xiong, Y.; Xue, Y.; York, D. M.; Zhao, S.; Zhu, Q.; Kollman, P. A. *Amber 2023*, University of California, San Francisco: 2023.
35. Roe, D. R.; Cheatham, T. E., 3rd, PTRAJ and CPPTRAJ: Software for processing and analysis of molecular dynamics trajectory data. *J. Chem. Theory Comput.* **2013,** *9*, 3084-3095. DOI: 10.1021/ct400341p.
36. Turner, P. J. *XMGRACE*, Version 5.1.25; Center for Coastal and Land-Margin Research, Oregon Graduate Institute of Science and Technology, Beaverton, Oregon: 2005.
37. Humphrey, W.; Dalke, A.; Schulten, K., VMD: visual molecular dynamics. *J. Mol. Graph.* **1996,** *14*, 33-38. DOI: 10.1016/0263-7855(96)00018-5.
38. Liang, M.; Mallari, C.; Rosser, M.; Ng, H. P.; May, K.; Monahan, S.; Bauman, J. G.; Islam, I.; Ghannam, A.; Buckman, B.; Shaw, K.; Wei, G. P.; Xu, W.; Zhao, Z.; Ho, E.; Shen, J.; Oanh, H.; Subramanyam, B.; Vergona, R.; Taub, D.; Dunning, L.; Harvey, S.; Snider, R. M.; Hesselgesser, J.; Morrissey, M. M.; Perez, H. D., Identification and characterization of a potent, selective, and orally active antagonist of the CC chemokine receptor-1. *J. Biol. Chem.* **2000,** *275*, 19000-19008. DOI: 10.1074/jbc.M001222200.
39. Harcken, C.; Kuzmich, D.; Cook, B.; Mao, C.; Disalvo, D.; Razavi, H.; Swinamer, A.; Liu, P.; Zhang, Q.; Kukulka, A.; Skow, D.; Patel, M.; Patel, M.; Fletcher, K.; Sherry, T.; Joseph, D.; Smith, D.; Canfield, M.; Souza, D.; Bogdanffy, M.; Berg, K.; Brown, M., Identification of novel azaindazole CCR1 antagonist clinical candidates. *Bioorg. Med. Chem. Lett.* **2019,** *29*, 441-448. DOI: 10.1016/j.bmcl.2018.12.024.
40. Karlström, S.; Nordvall, G.; Sohn, D.; Hettman, A.; Turek, D.; Ahlin, K.; Kers, A.; Claesson, M.; Slivo, C.; Lo-Alfredsson, Y.; Petersson, C.; Bessidskaia, G.; Svensson, P. H.; Rein, T.; Jerning, E.; Malmberg, A.; Ahlgen, C.; Ray, C.; Vares, L.; Ivanov, V.; Johansson, R., Substituted 7-amino-5-thio-thiazolo[4,5-d]pyrimidines as potent and selective antagonists of the fractalkine receptor (CX3CR1). *J. Med. Chem.* **2013,** *56*, 3177-3190. DOI: 10.1021/jm3012273.
41. *The PyMOL Molecular Graphics System*, Version 2.5.4; Schrödinger LLC: 2015.
